# Metabolomic and Metagenomic Analysis of Two Crude Oil Production Pipelines Experiencing Differential Rates of Corrosion

**DOI:** 10.3389/fmicb.2017.00099

**Published:** 2017-01-31

**Authors:** Vincent Bonifay, Boris Wawrik, Jan Sunner, Emily C. Snodgrass, Egemen Aydin, Kathleen E. Duncan, Amy V. Callaghan, Athenia Oldham, Turid Liengen, Iwona Beech

**Affiliations:** ^1^Department of Microbiology and Plant Biology, University of Oklahoma, NormanOK, USA; ^2^Institute for Energy and the Environment, University of Oklahoma, NormanOK, USA; ^3^Department of Biology, University of Texas of the Permian Basin, OdessaTX, USA; ^4^Research Centre Porsgrunn, Statoil ASA, Herøya IndustriparkPorsgrunn, Norway

**Keywords:** microbially influenced corrosion (MIC), reservoir microbiology, metabolomics, mass spectrometry, hydrocarbon degradation, anaerobic microbiology

## Abstract

Corrosion processes in two North Sea oil production pipelines were studied by analyzing pig envelope samples via metagenomic and metabolomic techniques. Both production systems have similar physico-chemical properties and injection waters are treated with nitrate, but one pipeline experiences severe corrosion and the other does not. Early and late pigging material was collected to gain insight into the potential causes for differential corrosion rates. Metabolites were extracted and analyzed via ultra-high performance liquid chromatography/high-resolution mass spectrometry with electrospray ionization (ESI) in both positive and negative ion modes. Metabolites were analyzed by comparison with standards indicative of aerobic and anaerobic hydrocarbon metabolism and by comparison to predicted masses for KEGG metabolites. Microbial community structure was analyzed via 16S rRNA gene qPCR, sequencing of 16S PCR products, and MySeq Illumina shotgun sequencing of community DNA. Metagenomic data were used to reconstruct the full length 16S rRNA genes and genomes of dominant microorganisms. Sequence data were also interrogated via KEGG annotation and for the presence of genes related to terminal electron accepting (TEA) processes as well as aerobic and anaerobic hydrocarbon degradation. Significant and distinct differences were observed when comparing the ‘high corrosion’ (HC) and the ‘low corrosion’ (LC) pipeline systems, especially with respect to the TEA utilization potential. The HC samples were dominated by sulfate-reducing bacteria (SRB) and archaea known for their ability to utilize simple carbon substrates, whereas LC samples were dominated by pseudomonads with the genetic potential for denitrification and aerobic hydrocarbon degradation. The frequency of aerobic hydrocarbon degradation genes was low in the HC system, and anaerobic hydrocarbon degradation genes were not detected in either pipeline. This is in contrast with metabolite analysis, which demonstrated the presence of several succinic acids in HC samples that are diagnostic of anaerobic hydrocarbon metabolism. Identifiable aerobic metabolites were confined to the LC samples, consistent with the metagenomic data. Overall, these data suggest that corrosion management might benefit from a more refined understanding of microbial community resilience in the face of disturbances such as nitrate treatment or pigging, which frequently prove insufficient to alter community structure toward a stable, less-corrosive assemblage.

## Introduction

Corrosion of ferrous alloys in oilfield environments is of considerable concern to the oil and gas industry worldwide. In industrialized economies, the cost of corrosion-related damage to industrial infrastructure has been estimated to be as high as 4% of GNP (Heitz, 1992). In the U.S. alone, the National Association of Corrosion Engineers (NACE) estimated that the direct cost of deterioration of metallic materials was $276 billion in 1998, or 3.1% of GNP, with incurred indirect monetary loss being equally high (Koch et al., 2002). Whether in natural or man-made environments, including oilfields, corrosion of metallic materials results from physico-chemical interactions between the metal and its surroundings. These interactions are often strongly influenced by the presence of complex microbial communities thriving as biofilms on internal or external surfaces of metals (Geesey et al., 2000; Beech et al., 2014). It is often assumed that the biofilm-driven corrosion process, referred to as microbiologically influenced corrosion (MIC) or biocorrosion, is initiated, enhanced, or facilitated by the metabolic activity of sessile microbial populations (Beech et al., 2005). In the absence of biofilms, abiotically formed corrosion products adhere to surfaces of deteriorating metals, thus providing a diffusion barrier to reactants and slowing the corrosion reaction (Beech and Sunner, 2004). In the presence of biofilms, however, the metabolic activity of organisms within the biofilm community can considerably alter interfacial electrochemistry between the biofilm matrix and the colonized metallic substrata, either enhancing or inhibiting corrosion reactions (Little and Ray, 2002; Ornek et al., 2002).

The activities of biofilm-associated prokaryotes, such as sulfide-producing prokaryotes (SPP), which in particular include sulfate-reducing bacteria (SRB; Hamilton, 1985; Lee et al., 1995), iron oxidizing/reducing bacteria, manganese-oxidizing bacteria, and bacteria that secrete extracellular polymeric substances (EPS) and organic acids (Beech and Gaylarde, 1999), are associated with metal deterioration and corrosion failures. In oilfield installations, SPPs are frequently invoked as a main cause of MIC (Gu, 2012). Despite extensive efforts to elucidate the roles that biofilms play in MIC and the mechanisms by which a single species or a complex microbial community can mediate corrosion processes, the mechanisms are still disputed. Notably, the relationship between biofilm community dynamics and MIC is poorly understood. Cultivation-based approaches are typically of limited utility, given that the vast majority of microbes cannot be brought into culture using standard cultivation techniques (Amann et al., 1995). As a consequence, nucleic acid based techniques have emerged as the standard approach for profiling of microbial community composition *in situ*. To date, several studies have characterized planktonic microbial communities implicated in MIC via molecular techniques (Dahle et al., 2008; Duncan et al., 2009; Gittel et al., 2009; Stevenson et al., 2011; Suflita et al., 2012). For example, 16S rRNA gene surveys of produced water from the North Sea Troll formation indicated the presence of bacteria belonging to *Firmicutes*, *Bacteroidetes*, and δ-proteobacteria, as well as Archaea related to the genera *Methanococcus* and *Methanolobus* (Dahle et al., 2008). Similar taxonomic groupings have been detected at other production facilities and oil reservoir produced waters, although considerable variability among sites have also been observed, reflecting variations in *in situ* environmental parameters (e.g., reservoir temperature, facility production practices, and availability of oxygen, nitrate, and sulfate; Stevenson et al., 2011). Importantly, when comparing bacterial community structure in production waters from two adjacent North Sea oil reservoirs, which differed solely in the method of controlling sulfide production, it was revealed that nitrate treatment was associated with lower microbial diversity yet higher cell densities, as well as bacterial taxa known to have the capability of nitrate reduction (Gittel et al., 2009).

Although insightful, requisite studies thus far have lacked a broader perspective of *in situ* microbial metabolic capabilities and, importantly, activities. In addition, reports on biofilm community structure and their potential function and potential contribution to corrosion are scarce (Stevenson et al., 2011; Lenhart et al., 2014). Recent advances in DNA sequencing methods (Buermans and den Dunnen, 2014) and rapid progress in techniques of mass spectrometry, in particular metabolomics (Patti et al., 2012; Monteiro et al., 2013), have greatly enhanced our capacity to interrogate both the metabolic potential and the actual metabolic activity of biofilm communities implicated in corrosion (Bonifay et al., 2013; Sunner and Beech, 2015).

Here, we report a combination of metabolomic and metagenomic measurements conducted on material recovered from pigging of two North Sea production pipelines. The aim was to gain insight into the contribution of prokaryotic biofilms to corrosion of these structures, given that they experienced very different levels of deterioration.

## Materials and Methods

### Field Sample Collection

Samples comprising pigging debris were collected from the production pipelines of two adjacent oil-fields, both located in the Norwegian sector of the North Sea. At the time of sampling, the pipeline of one of the fields had been experiencing on-going and severe corrosion, whereas the corrosion status in the other pipeline was moderate and of no concern. The pipelines will be designated herein as “HC” (high corrosion) and “LC” (low corrosion), respectively. Pigging debris from sequential pigging runs was collected during routine cleaning operations. For each pipeline, early and late pigging material was recovered by sampling from the third and eleventh pig run, respectively. The samples are here identified as LC3, HC3, LC11, and HC11. Pigging material was collected into sterile glass bottles that had been filled with nitrogen gas. Bottles were filled to exclude head space, placed on ice, and delivered to a laboratory, where approximately 100 g of each sample was removed, acidified, and subsequently frozen and stored at -70°C.

### Metabolite Analysis

For each of the four samples, metabolic profiling was performed in triplicate, using ultra-high performance liquid chromatography/high-resolution mass spectrometry (UPLC/HRMS) with electrospray ionization (ESI) in both positive and negative ion modes. For each sample, 1 g was removed and further acidified by the addition of 5 mL of 4 M HCl (ACS grade, EMD Millipore). Five milliliters of a 50/50 (v/v) mixture of ethyl acetate and dichloromethane (ACS grade) were added; the mixture was vortexed for 2 min; and sonicated in a water bath for 15 min. The phases were allowed to separate, and the aqueous layer was removed. The remaining organic solution was evaporated to dryness under a stream of N_2_ gas, and the residue was reconstituted in 100 μL of isopropyl alcohol (HPLC grade, EMD Millipore). Five microliters of each solution were injected on an Agilent 1290 Infinity UPLC system, coupled to an Agilent 6538 quadrupole time-of-flight mass spectrometer (QTOF-MS; Agilent, Santa Clara, CA, USA) equipped with a dual-spray ESI source. For negative ion mode, an Acquity UPLC^®^ HSS C18 SB column (1.8 μm, 2.1 mm × 100 mm; Waters Inc., USA) was used for separation with a flow rate of 0.4 mL min^-1^. The initial concentration of 23.5% of HPLC grade acetonitrile (EMD Millipore) in HPLC grade water (EMD Millipore) was maintained for 5 min, and this was followed by a linear gradient from 23.5 to 95.5% over 35 min, and by 95.5% maintained for an additional 5 min. For positive ion mode, a SeQuant^®^ ZIC^®^-HILIC column (5 μm, 150 mm × 4.6 mm, The Nest Group, Inc., Southborough, MA, USA) was used at a flow rate of 0.30 mL min^-1^. An initial concentration of 80% HPLC grade acetonitrile in HPLC grade water was maintained for 5 min; followed by a linear gradient from 80 to 20% over 30 min; and isocratic operation at 5% acetonitrile for an additional 8 min. In positive ion mode only, HPLC solvent mixtures contained 0.1% HPLC grade formic acid (Sigma Aldrich) to improve ionization efficiency. In both positive and negative ion modes, MS parameters were as follows: gas temperature, 325°C; capillary voltage, 3,500 V; fragmentor voltage, 160 V; m/z range, 50–1100; detector signal acquisition rate, 4 GHz; and spectrum storage rate, 1 s^-1^.

Raw MS data were processed under IDEOM (version 19; Creek et al., 2012) workflow control. IDEOM uses XCMS Centwave (Tautenhahn et al., 2008) for peak detection and mzMatch.R (Scheltema et al., 2011) for alignment of peaks, filtering, and storage of data in peakML files. XCMS (Smith et al., 2006) and mzMatch (Scheltema et al., 2011) are scripts in the R environment. Abundance values are here reported in instrument-specific units (i.u.), and an abundance of 1,000 i.u. corresponds to the detection of about 80 ions. An abundance threshold of three times the noise level, as determined by XCMS, was used for peak-picking. For alignment of features between triplicates, samples and ionization modes at a maximum mass difference of 6 ppm and a retention time difference of 0.25 min were allowed. The parameter settings used for peak-picking and alignment are listed in **Supplementary Table S1**.

### Putative Identification of Metabolites

Detected features were matched against metabolites in IDEOM’s version of the KEGG [Kyoto Encyclopedia of Genes and Genomes database (Kanehisa et al., 2014)], as well as the Metacyc (Caspi et al., 2014), Lipidmaps (Fahy et al., 2009), and HMDB (Wishart et al., 2013) databases using the criterion that the experimentally determined exact mass should be within 6 ppm of the theoretical mass of the metabolite. For features detected in positive ion mode (about 72% of the total), identification was also based on comparing experimental and calculated retention times. Creek et al. (2011) described improved metabolite identification using retention time prediction for HILIC chromatography, and the method is implemented in IDEOM. The retention time calculator is based on a quantitative structure-retention relationship model that incorporates six physico-chemical variables in a multiple-linear regression based on 120 authentic standard metabolites. For the present work, 33 metabolite standards were used to calibrate the retention time predictor (**Supplementary Table S2**). Putative metabolites, whose experimentally observed retention time was more than 45% off from that calculated by the model, were rejected. Metabolites matching eight of the standards were observed in the pigging samples.

With the use of an RT calculator, an identification level of 2 can be claimed (Sumner et al., 2007; Salek et al., 2013), but given the level of uncertainty associated with identification, detected metabolites herein are referred to as “putative.” The eight metabolites matching standards, however, were identified with a higher degree of certainty. The IDEOM software assigns a level of confidence to each identification using a numerical value that ranges from 1 to 10, where 10 reflects the highest level of confidence. The confidence value assigned is based on the magnitude of the mass error, on the difference between experimental and expected retention times (for features detected in positive ion mode), and on a database priority order internal to IDEOM. For any feature, the metabolite with the highest confidence score is selected.

After the putative identification of extracted features with metabolites and other compounds in the KEGG database, the (putatively) identified metabolites were matched with the Pathos (Leader et al., 2011) metabolic pathway database to identify ‘active’ pathways. The grouping of metabolites into named pathways in Pathos is identical to that used in the KEGG database. Finally, targeted analysis was performed for alkylsuccinates and alkylbenzylsuccinates, known anaerobic degradation products of alkanes and alkylated aromatics, respectively (Agrawal and Gieg, 2013; Rabus et al., 2016). Positive identification required that compounds were observed with the correct mass (± 2 ppm) and retention time (± 10%). Two standard alkylsuccinates, hexylsuccinate, and decylsuccinate, were used. The retention times of other alkylsuccinates were determined using samples from anaerobic alkane degradation cultures known to produce prominent series of alkylsuccinate peaks. For identification of benzylsuccinates, the standards ethylbenzylsuccinate and benzylsuccinate were used.

### DNA Extraction and Metagenome Sequencing

DNA was extracted from 3 g of pigging sample using the Maxwell 16 Tissue LEV Total RNA Purification Kit with the automated Maxwell 16 Research System (Promega, Madison, WI, USA). Briefly, 1.5 mL of 10 mM Tris:50 mM EDTA, 500 μL of 10% SDS, and 50 μL of proteinase K (>600 U mL^-1^ solution; QIAGEN, Valencia, CA, USA) were added to the 3 g of sample in a 50-mL Falcon tube. To facilitate cell lysis, the sample mixture was vortexed for 30 s and incubated at room temperature (∼21°C) for 25 min, followed by 5 min at 65°C. To each sample, one tube of lysing matrix E beads (MP Biomedicals, Solon, OH, USA) was added, and samples were bead-beaten on the high setting for 2 min using a vortex adapter (MOBIO Laboratories, Carlsbad, DA, USA). Samples were centrifuged at 6,000 × *g* for 2 min, and the supernatants were transferred to four pre-dispensed reagent cartridges with the addition of 250 μL of RNA lysis buffer (RLA) and 250 μL of RNA dilution buffer (RDB). Samples were processed using the pre-programmed RNA isolation mode with the DNA removal steps omitted. Post-extraction, DNA extracts were centrifuged for 1 min on a low setting to pellet particulate carryover from the extraction procedure. The extracts were pooled and quantified using the Qubit dsDNA BR Assay kit with the Qubit 2.0 fluorometer (Life Technologies, Carlsbad, CA, USA) following the manufacturer’s instructions. Metagenome sequencing was conducted by generating libraries with 400 bp fragment sizes by first fragmenting DNA with a Covaris Focused-ultrasonicator (Covaris). Separate Illumina libraries with unique index tags were generated for each sample. DNA was then pooled and sequenced using the Illumina MiSeq PE250 platform at the Oklahoma Medical Research Foundation sequencing core.

### qPCR for 16S Bacteria and Archaea

16S rRNA gene copy numbers were determined by quantitative PCR (qPCR), as previously described (Wawrik et al., 2012). In brief, amplification was conducted using bacterial 16S rRNA gene qPCR forward primer 27F (5′-AGA GTT TGA TCM TGG CTC AG-3′) and reverse primer 519R (5′-GWA TTA CCG CKG CTG-3′), as well as archaeal-specific qPCR forward primer 8AF (5′-TCC GGT TGA TCC TGC C-3′) and reverse primer A344R (5′-TCG CGC CTG CTC CIC CCC GT-3′). The qPCR reactions were performed in 30 μL of sample using *Power* SYBR^®^ Green PCR Master Mix (Applied Biosystems) with 250 nM of each primer and 2 μL of template DNA in an Applied Biosystems ABI 7300 Real Time PCR System. PCR conditions were as follows: 50°C for 2 min and 95°C for 8 min, followed by 40 cycles of 95°C for 30 s, 55°C for 30 s, and 72°C for 30 s. Genomic DNA of *Roseobacter denitrificans* Och114 was used as a standard for Bacteria, while a plasmid (pCRII-TOPO^®^, Invitrogen) containing the complete 16S rRNA gene of *Methanospirillum hungatei* JF-1 served as a standard for Archaea.

### 16S rRNA Gene-Based Community Analysis

DNA was amplified by targeting partial 16S rRNA genes with the universal (Bacteria and Archaea) primers S-D-Arch-0519-a-S-15 (5′-CAG CMG CCG CGG TAA-3′) and S-D-Bact-785-a-A-21 (5′-GAC TAC HVG GGT ATC TAA TCC-3′) as previously described (Klindworth et al., 2013). Based on *in silico* analysis, these primers exclude eukaryotes, but cover 86.5 and 87.1% of bacterial and archaeal phyla, respectively. If one mismatch is allowed, only candidate divisions WS6, TM7, and OP11, as well as phylum Nanoarchaeota are undetectable with these primers (Klindworth et al., 2013). Forward primers were 5′-M13 tagged to allow the addition of Illumina primers and barcodes (Wawrik et al., 2012). PCR reactions were carried with 2X PCR Master Mix (Fermentas/Thermo Scientific), and products were checked by gel electrophoresis to confirm that a single band was produced. PCR products were cleaned using a QIAquick^®^ PCR Purification Kit (Qiagen), and amplicons were tagged for MiSeq Illumina sequencing by including a unique 8 bp barcode into each amplicon and mixing products in equimolar ratios (Wawrik et al., 2012). MiSeq Illumina sequencing was performed as previously described (Caporaso et al., 2010b) with the modification of an added CC between the adapter and the barcode.

### 16S rRNA Gene Sequence Classification

For Illumina sequence libraries of 16S rRNA gene PCR products, adapter sequences were removed from raw Illumina reads using Cutadapt (Martin, 2011). Sequences were trimmed using a quality score of 30, and paired-end reads were joined. All unpaired reads were removed from further analysis. Sequences were clustered and assigned taxonomy using the QIIME pipeline (Caporaso et al., 2010b). After de-multiplexing, sequences were clustered into Operational Taxonomic Units (OTUs) using UCLUST at the 95% identity level and screened for the presence of chimeric sequences. A randomly selected set of representative set of sequences were then aligned to the SILVA small subunit rRNA reference alignment^1^ using the PyNAST algorithm (Caporaso et al., 2010a). For the analysis of 16S rRNA genes contained in metagenomic datasets, full length 16S sequences were extracted using EMIRGE (Miller, 2013). Adapter and quality trimmed metagenomic reads were utilized (see below). Forty iterations were conducted using the SILVA small subunit rRNA reference alignment with otherwise default parameters. Full length sequences were exported to the Ribosomal RNA Database Project for classification. The most closely related type strains were aligned with metagenomic 16S sequences, and Neighbor-Joining, Parsimony, Maximum Likelihood phylogenetic trees were generated using MEGA 6 (Tamura et al., 2013).

### Metagenome Analysis

Metagenomic sequence reads were trimmed to remove Illumina index tags and bar codes using Cutadapt (Martin, 2011). Reads were subsequently quality trimmed with a minimum quality score cutoff of 30. Sequences shorter than 50 bp were discarded. Metagenomes were analyzed as both unassembled and assembled datasets. Assembly of paired reads was conducted using Ray (Boisvert et al., 2010) with a K-mer of 31. Open reading frames (ORF) were predicted using the Prokaryotic Dynamic Programming Gene Finding Algorithm (MetaProdigal; Hyatt et al., 2010, 2012). ORF frequency read counts were generated via mapping trimmed reads onto ORFs using Bowtie 2 (Langmead and Salzberg, 2012). All predicted ORFs were compared to the NCBI NR, COG (clusters of orthologous groups), and KEGG databases using the blastN and blastX functions of CAMERA (Sun et al., 2011). ORFs that were assigned to COGs related to S and N cycling, as well as methanogenesis, were compared to the NR database via blastX. Taxonomic identifiers were retrieved from each best hit and then analyzed via MEGAN (Huson et al., 2007) to evaluate the phylogenetic breakdown of each COG in the dataset (**Supplementary Table S3**). Genome scaffolds of dominant microorganisms were reconstructed via contig and tetranucleotide frequencies as described by Albertsen (Albertsen et al., 2013). Scaffolds were uploaded to Rapid Annotation using Subsystem Technology (RAST^2^) for annotation and genome recruitment analysis. Unassembled reads were exported to MG-RAST for annotation, PCoA and rarefaction analysis, heatmap generation, and KEGG map analysis.

## Results

### Site Characteristics

The two production pipelines investigated in this study were selected because one of them was experiencing on-going and severe corrosion (“HC”), whereas the other was experiencing low levels of corrosion (“LC”). This difference in corrosion was interesting because the pipelines were associated with adjacent fields in the North Sea, and their physico-chemical environments were very similar to each other (see **Table 1**). Specifically, the temperature (≈80°C), oil:water ratios, acetate, and propionate concentrations were similar in both pipelines; however, the sulfate concentration was approximately eightfold higher in water obtained from the LC compared to the HC pipe. At the time of sampling, injection water at both fields had been treated with nitrate over a period of several years. Sea water injection at HC started in 1999, while nitrate treatment did not commence until 2001 after significant souring and corrosion problems were first noticed. Production in the LC system started in 1997. Biocide treatment with glutaraldehyde of the injection water (sea water) occurred between 1997 and 2007, after which nitrate treatment was started and biocide treatment ended.

**Table 1:** Site characteristics.

Site	LC pipeline	HC pipeline
*In situ* corrosion rate^∗^	<0.1 mm y^-1^	0.5–5.3 mm y^-1^
Nitrate treatment	From inception	Started after aggressive corrosion was observed
Pressure support	Nitrate treated SW	Nitrate treated SW
*In situ* biomass	High	Low
Pipeline	12 km, 12″ carbon steel (X65)	10 km, 10″ carbon steel (0.5% Cr)
Temperature	> 80°C	80°C
Oil:water ratio	1:3	2:5
Sulfate (mg L^-1^)	980	121
Phosphate (mg L^-1^)	<0.5	<0.5
Acetate (mg L^-1^)	330	311
Propionate (mg L^-1^)	30	31
Pigs analyzed	3 and 11	3 and 11
# Bases sequenced	2.20 × 10^9^ and 2.11 × 10^9^	2.20 × 10^9^ and 7.54 × 10^8^


*^∗^As determined by coupon weight loss (*n* = 19).*

### 16S rRNA Gene Surveys

The microbial community composition was assessed via sequencing of 16S rRNA gene PCR products (**Supplementary Figure S1**). A total of 1,126 OTU’s were detected in bacterial PCR-based 16S rRNA gene sequence data across all samples. Of these, 15 OTUs accounted for >80% of all read data, indicating a high degree of enrichment for a small number of lineages. The LC pipeline was overwhelmingly dominated by Gammaproteobacteria in both the early (74% in LC3) and the late pigging material (72% in LC11; **Supplementary Figure S1B**). In contrast, Gammaproteobacteria only accounted for 3.2 and 2.7% of reads in the HC3 and HC11 pigging material, respectively. HC sequence libraries contained comparatively greater proportions of sequences classified within the Thermotogae, Synergistes, and Firmicutes. Sequences classified as methanogenic archaeal lineages fell within several methanomicrobial OTUs as well as a single OTU within the Methanococci. Methanomicrobial lineages collectively accounted for less than 0.04% of methanococcal reads in each of the four libraries. The methanococcal OTU accounted for less than 0.2% of the reads in LC samples but for 3 and 21% of reads in the HC3 and HC11 samples, respectively. A significant portion of reads were classified within marine epipelagic taxa, accounting for 12% of LC3 and LC11 reads, and for 4.3 and 4.8% of HC3 and HC11 16S RNA sequences observed (**Supplementary Figure S2**). Among the detected epipelagic marine lineages were *Synechococcus*, *Prochlorococcus*, Marine Group I Crenarchaeota, *Pelagibacter ubique* (a.k.a. Sar11), *Oceanospirillum*, and a diverse group of 16S gene sequences originating from marine chlorophytes and chromalveolates including heterokonts, haptophytes, and dinoflagellates. This suggests that the LC pipeline contained a greater proportion of recently introduced surface seawater than the HC pipeline.

### Metagenomic Surveys

**FIGURE 1:**
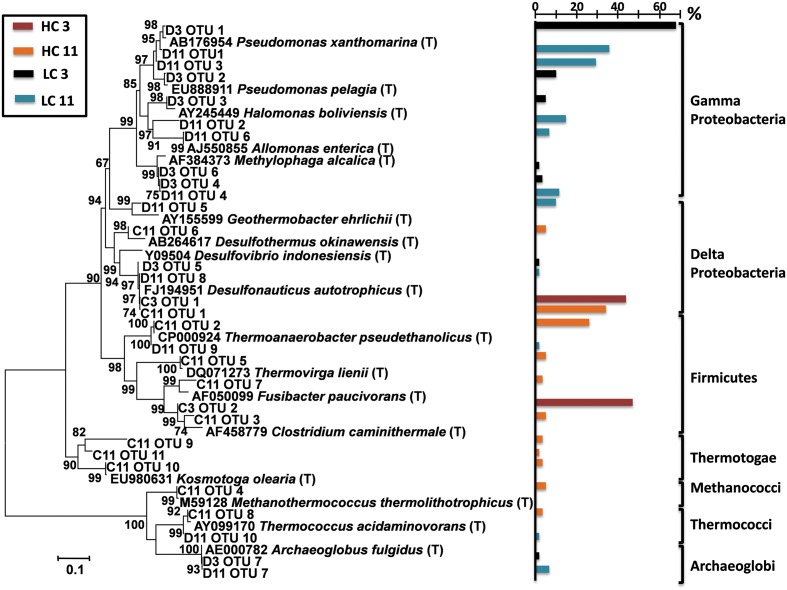
**Phylogenetic analysis of full length 16S rRNA genes extracted from metagenomic data using EMIRGE (Miller, 2013).** Operational Taxonomic Units (OTUs) were compared to the RDP database, and the most closely related 16S rRNA genes from type strains (designated with a T) were used for phylogenetic analysis of metagenomic OTUs. A bootstrapped (5,000 replicates) Neighbor-Joining tree is shown. The colored bars indicated the proportion of 16S reads that were assembled into respective full length 16S sequences for each of the detected phylotypes (percentage scale shown in scale bar above). Bacterial and archaeal divisions/families are indicated and color coded. D3 = LC3; D11 = LC11; C3 = HC3; and C11 = HC11.

To generate a deeper classification of dominant microbes, full length 16S rRNA gene sequences were reconstructed from metagenomic data using EMIRGE (Miller, 2013) (**Figure 1**). This analysis produced a total of 30 full length bacterial and archaeal 16S rRNA sequences. With respect to the dominant microbial populations, this analysis mirrored results from PCR-based 16S rRNA gene sequencing. Gammaproteobacterial lineages that dominated in the LC samples were most closely related to marine pseudomonads and to halo- and alkali-tolerant halomonads and *Methylophaga* spp. Early pigging material at the HC site (HC3) was dominated by bacteria most closely related to thiosulfate-reducing Firmicutes within the genus *Clostridium* and deltaproteobacteria closely related to sulfate/thiosulfate-reducing *Desulfonauticus* spp. Bacteria related to *Desulfonauticus* spp. were also most abundant in the 16S sequences from late HC pigging debris (HC11), which also contained a dominant population of bacteria most closely related to thermophilic fermentative *Thermoanaerobacter* spp. Emirge analysis also reconstructed 16S rRNA genes for lineages closely related to *Clostridium, Methanothermococcus, Thermovirga, Desulfothermus, Fusibacter, Kosmotoga*, and *Thermococcus*.

**FIGURE 2:**
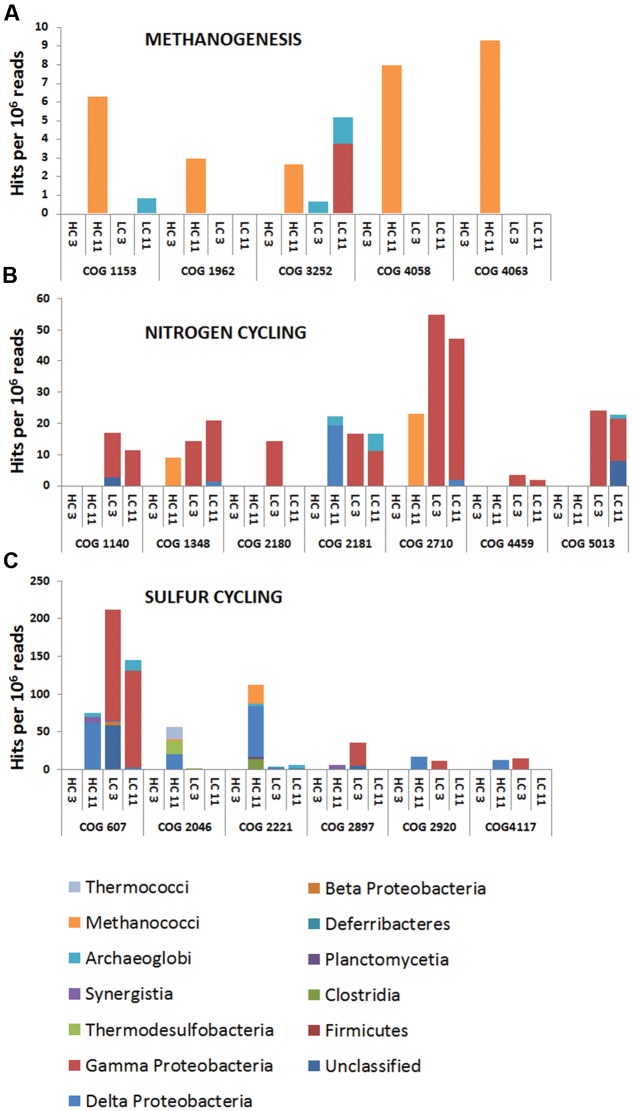
**Cluster of Orthologous Groups (COG) analysis of metagenomic data from high corrosion (HC) and low corrosion (LC) pigging samples.** Open reading frames (ORF) were predicted from assembled, paired-end read data. Predicted ORFs were compared to the COG database in CAMERA (Sun et al., 2011) and are shown as hit frequencies per million reads (*y*-axis) where the frequencies were calculated by mapping read data onto predicted ORFs and normalizing to the respective metagenome size. Frequencies for COGs involved in **(A)** methanogenesis, **(B)** nitrogen cycling, and **(C)** sulfur cycling.

To gain insight into the metabolic capabilities of these microbial populations, several analyses were performed. First, all metagenomic reads were classified into COGs via CAMERA. Reads associated with individual COGs for denitrification, methanogenesis, and sulfate reduction (**Supplementary Table S3**) were blasted against NCBI’s non-redundant nucleotide database, and the identities of their best matches were used to extract phylogenetic information. The phylogenetic breakdown of a subset of these COGs is shown in **Figure 2**. COG analysis indicated that methanogens were limited to the HC11 sample and belonged to the class of Methanococci (**Figure 2A**). Nitrogen cycling genes were primarily associated with Gammaproteobacteria found mostly in the LC samples. In the HC11 sample, Archaeoglobi appear to contain genes for nitrogen fixation (COGs 1348 and 2710; **Figure 2B**). Sulfate reduction COGs were more diverse (**Figure 2C**) and suggested the presence of sulfate-reducing Clostridia, Deltaproteobacteria, and Archaeoglobi in the HC samples. In LC samples, these COGs yielded a number of hits to Gammaproteobacteria. However, the latter are not known to reduce sulfate, and these hits are more likely the result of homology among domain structures of enzymes with unrelated functions.

**FIGURE 3:**
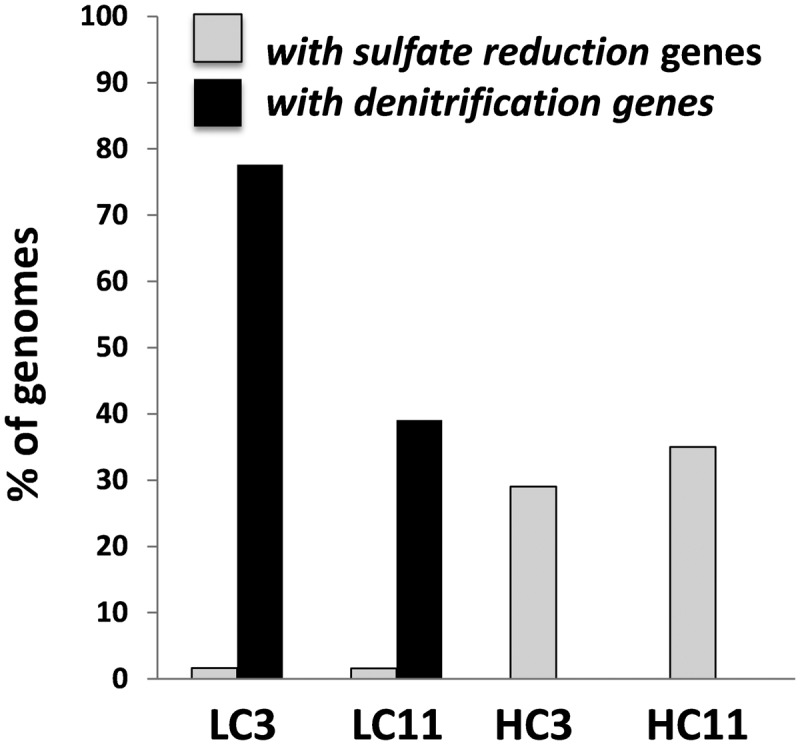
**Estimated relative abundance of cells in each of the pigging debris samples carrying sulfate reduction and denitrification pathway genes.** The estimates were generated by considering the proportion of all metagenomic reads that assembled into individual genome scaffolds reconstructed from metagenomic data using multi-metagenome (Albertsen et al., 2013) (**Table 2**; **Supplementary Figure S3**). Each genome scaffold was then annotated via Rapid Annotation using Subsystem Technology (RAST) to identify the presence of denitrification and sulfate reduction pathways (**Supplementary Tables S3**–**S5**).

For more targeted analysis, genome scaffolds for dominant microorganisms were reconstructed using multi-metagenomic analysis (Albertsen et al., 2013) by clustering assembled contigs based on coverage, %GC, phylogenetic affiliation of housekeeping genes, and tetranucleotide frequencies (**Supplementary Figure S3**). This analysis allowed the reconstruction of 15 binned genomes (referred to as genome ‘scaffolds’; **Table 2**), which were annotated and assigned to their closest relative based on recruitment analysis of predicted genes via RAST (Overbeek et al., 2014). Annotations were interrogated for the presence of key N and S cycling genes, revealing that denitrification genes were exclusively present in two LC scaffolds most closely related to *Pseudomonas stutzeri* (**Supplementary Table S4**). Sulfate reduction genes, in contrast, were detected in both LC and HC scaffolds and were associated with the reconstructed genomes of the Deltaproteobacteria *Desulfonatronospira thiodismutans* and *Desulfohalobium retbaense*, as well as the archaeon *Archaeoglobus fulgidis* (**Supplementary Table S5**). These data suggest that the LC and HC communities differ in their potential to utilize sulfate and nitrate as terminal electron acceptors. It can be estimated from the size of the metagenomes and the coverage of the reconstructed genomes that 29 and 34% of cells in the HC3 and HC11 but only 1.5 and 1.6% in the LC3 and LC11 pigging communities, respectively, are capable of sulfate reduction (**Figure 3**). Conversely, it is estimated that 78 and 39% of cells in LC3 and LC11 samples contain genes for denitrification, while such genes were absent from any of the HC genome scaffolds. Similar results with respect to denitrification potential are obtained when read data are considered directly via MG-RAST analysis (**Table 3A**). The frequency of reads annotated as nitrogen cycling genes in LC samples was consistent with the interpretation that the majority of cells contained the capability to reduce nitrate. In contrast, N-cycling genes were almost absent from HC metagenomes. MG-RAST based blast analysis of S cycling genes produced hits in all four metagenomes (**Table 3B**), a result that was consistent with the analysis of full length gene sequences generated by assembly and genome binning (**Supplementary Table S5**).

**Table 2:** Genome recruitment of genome scaffolds extracted from metagenomic data.

Site	Contigs	Total length	N50	% G+C	Coverage pig #3	Coverage pig #11	# of Open reading frames (ORFS)	Best recruitment	Recruitment hits
Low Corrosion (LC)	123	4749114	58352	61.42	161.5	19.1	4720	*Pseudomonas stutzeri*	2279
	21	3244921	436384	44.66	72.3	21.1	3087	*Methylophaga thiooxidans*	1301
	118	4521009	56215	52.31	52.3	28.6	4075	*Shewanella amazonensis*	436
	43	4853824	189984	62.79	37.2	175.5	2942	*Pseudomonas stutzeri*	2467
	67	2261788	43799	48.08	28.6	6.5	2571	*Archaeoglobus fulgidis*	2196
	126	1895321	28962	51.91	13.7	7.5	2070	*Thermococcus kodakarensis*	646
	116	2136066	33623	37.97	8.5	13.4	2185	*Desulfonatronospira thiodismutans*	319
High Corrosion (HC)	51	2132562	63261	37.94	220	111	2144	*Desulfonatronospira thiodismutans*	342
	40	2131658	85501	51.87	191.3	1.9	2350	*Thermococcus kodakarensis*	1121
	84	2335091	38988	34.24	89.1	7.9	2421	*Thermoanaerobacter pseudethanolicus*	703
	21	1256481	81700	32.1	38.9	0.9	1255	*Methanothermococcus okinawensis*	284
	36	2640115	123629	34.43	20.5	9.7	2638	*Desulfohalobium retbaense*	164
	85	1907033	30617	41.69	19.2	3.8	1838	*Kosmotoga olearia*	1645
	142	3318928	33498	33.18	12.9	33.1	3198	*Alkaliphilus metalliredigens*	370
	138	1633891	13445	30.53	10.6	9.5	1611	*Thermosipho africanus*	1482


**Table 3A:** Frequency of N-cycling genes in metagenomic data as determined by MG-RAST annotation.

NITROGEN CYCLING											

**Sample**	***amoA***	***narB***	***nasA***	***nifH***	***ureC***	***nirS***	***nirK***	***nosZ***	***norB***	***narG***	***napA***
LC 3	200.5	0.0	17.2	116.3	9.3	4.0	26.5	331.5	506.8	798.2	393.6
LC 11	175.3	0.0	11.7	133.6	35.8	22.3	12.3	173.9	503.7	709.3	104.0
HC 3	0.0	0.0	0.0	0.0	0.0	0.0	0.0	1.6	0.0	1.6	0.8
HC 11	0.0	10.5	0.0	0.8	0.3	0.0	0.0	0.0	1.0	0.5	0.0


*# hits/10^6^ reads. The numbers of reads annotated with respective gene identifiers (columns) per million metagenomic reads are shown.*

**Table 3B:** Frequency of S-cycling genes in metagenomic data as determined by MG-RAST annotation.

SULFATE REDUCTION										
	
Sample	*dsrA*	*dsrB*	*dsrk*	*dsrJ*	*dsrP*	*dsrM*	*dsrE*	*dsrF*	*dsrH*
LC 3	9.6	8.2	8.2	0.0	0.0	3.1	67.5	54.8	27.4	
LC 11	47.6	25.6	4.1	0.0	0.9	0.9	56.7	72.8	36.7	
HC 3	0.0	8.8	132.8	0.0	4.8	58.4	49.6	1.6	1.6	
HC 11	20.1	22.2	64.7	0.5	7.4	54.5	58.4	12.0	0.8	

**SULFUR OXIDATION**						**THIOSULFATE REDUCTION**				
	
**Sample**	***soxA***	***soxB***	***soxX***	***soxY***	***soxZ***	**Rhodanese domain**		**Thiosulfate sulfotransferase**		**Thiosulfate reductase**	

LC 3	15.2	0.3	2.8	3.4	42.6	987.4		314.2		87.0	
LC 11	42.6	0.0	0.0	0.0	138.9	828.8		282.0		19.7	
HC 3	3.2	0.0	0.0	0.0	0.0	547.2		80.0		32.8	
HC 11	18.4	0.0	0.0	0.0	2.5	71.6		23.5		7.9	


*# hits/10^6^ reads. The numbers of reads annotated with respective gene identifiers (columns) per million metagenomic reads are shown.*

Given the presence of crude oil in the HC and LC pipelines, pigging envelope metagenomes were further analyzed for the presence of genes associated with aerobic and anaerobic hydrocarbon degradation by evaluating the MG-RAST annotations. A search for reads similar to aerobic hydrocarbon degradation genes revealed large differences in the frequencies of oxygen-dependent, hydrocarbon activation enzymes (i.e., mono- and di-oxygenases) among samples. The LC metagenomes contained, on average, >250-fold more monooxygenase >4- to 8-fold more dioxygenase genes per million reads than the corresponding HC metagenomes. An analysis of the dominant types of mono- and di-oxygenases indicated high potential for the degradation of cyclic ketones, mono-cyclic aromatic compounds, and alkanes by LC-, but not HC-, associated communities (**Table 4A**). Analysis of reconstructed genome scaffolds painted a similar picture, with few genes annotated as mono- and di-oxygenases in the HC scaffolds. LC scaffolds contained 6.8-fold more hits to mono- and di-oxygenases than HC scaffolds. (**Supplementary Table S6**). In particular, one LC scaffold which is most similar to *Pseudomonas stutzeri*, as per recruitment analysis, stands out for containing a range of genes annotated as dioxygenases involved in aromatic hydrocarbon degradation (**Supplementary Table S6B**).

**Table 4:** Frequency of hydrocarbon degradation genes in metagenomic data as determined by MG-RAST.

(A) Aerobic hydrocarbon degradation							
**Sample**	**(Total) monooxygenases**	**Alkane monooxygenase**	**Cyclopentanone monooxygenase**	**Cyclohexanone monooxygenase**	**Cyclodecanone monooxygenase**	**Cyclododecanone monooxygenase**	**Phenol monooxygenase**

LC 3	1331.8	136.7	0.3	79.6	0.0	6.2	3.1
LC 11	1453.0	0.6	0.0	227.0	0.0	0.0	0.0
HC 3	3.2	0.0	0.0	0.0	0.0	0.0	0.0
HC 11	7.6	0.0	0.0	0.8	0.0	0.0	0.0

**Sample**	**Phenylacetone monooxygenase**	**Xylene monooxygenase**	**Cytochrome P450 monooxygenase**	**Alkane hydroxylase**	**(Other) monooxygenase**		

LC 3	29.6	16.7	0.3	19.5	1039.9		
LC 11	37.0	0.0	0.0	0.0	1188.3		
HC 3	0.0	0.0	0.0	0.0	3.2		
HC 11	0.3	0.0	0.0	0.0	6.6		

**Sample**	**(Total) dioxygenases**	**Ethylbenzene dioxygenase**	**Nitropropane dioxygenase**	**Isopropylcatechol dioxygenase**	**Hydroxyphenylpyruvate dioxygenase**	**Phthalate dioxygenase**	**Benzoate dioxygenase**

LC 3	4262.0	250.7	440.2	140.0	557.3	11.6	147.1
LC 11	2401.7	59.0	382.4	28.8	347.5	0.0	19.4
HC 3	572.0	0.0	215.2	0.0	0.0	0.0	0.0
HC 11	523.6	0.0	202.4	0.3	0.0	0.0	0.0

**Sample**	**Ring opening dioxygenases**	**Catechol dioxygenase**	**Homogentisate dioxygenase**	**Isopropylbenzene dioxygenase**	**Protocatechuate dioxygenase**	**Toluate dioxygenase**	**(Other) dioxygenase**

LC 3	181.3	399.8	246.2	325.3	32.2	331.2	1199.1
LC 11	90.8	91.0	286.1	96.3	7.9	71.1	921.4
HC 3	0.0	0.0	0.0	0.0	0.0	0.0	356.8
HC 11	0.8	1.0	0.3	0.0	0.0	0.5	318.4

**(B) Anaerobic hydrocarbon degradation**						

**Sample**	**Alkylsuccinate synthase**	**Benzylsuccinate synthase**	**Ethylbenzene dehydrogenase**	**Phenylphosphate carboxylase**	**Benzene carboxylase**	**Acetophenone carboxylase**

LC 3	0.0	0.0	0.0	0.0	0.0	3.4
LC 11	0.3	0.0	0.0	0.0	0.0	31.1
HC 3	1.6	0.0	0.0	0.0	0.0	0.8
HC 11	0.3	0.0	0.0	0.0	0.0	0.0


*# hits/10^6^ reads. The numbers of reads annotated with respective gene identifiers (columns) per million metagenomic reads are shown.*

Genes associated with known anaerobic hydrocarbon degradation pathways were essentially absent from both the HC and the LC metagenomes as determined by MG-RAST analysis. To confirm this observation, blastP searches of predicted proteins from each genome scaffold were conducted against a custom database of known proteins involved in anaerobic hydrocarbon degradation (Callaghan and Wawrik, 2016) revealing no significant hits. In total, only four reads (three in the HC and one in the LC samples; **Table 4B**) with significant blast hits against the catalytic subunit of naphthylmethylsuccinate synthase (NmsA) in the Deltaproteobacterium NaphS6 (Annweiler et al., 2000) were observed, but further analysis (data not shown) did not support a phylogeny that would classify these reads as proteins that catalyze hydrocarbon addition to fumarate, but rather placed them in association with known pyruvate formate lyases. Similarly, a number of reads produced blast hits to acetophenone carboxylase, but closer analysis suggested that these sequences contained a motif common to many types of carboxylases that are not related to hydrocarbon degradation.

### Metabolomic Surveys

Pigging debris from oil pipelines contain a large diversity of chemicals, including microbial metabolites, crude oil constituents, material introduced with the seawater, industrial chemicals such as corrosion inhibitors, as well as metal ions from pipeline corrosion and their complexes. The main objective of the metabolomic analysis was to identify groups of features that potentially distinguish two systems experiencing differential corrosion rates. Total ion chromatograms for the four samples are shown in **Supplementary Figure S4**. In all, 9,824 aligned features were detected, with 5,917 features in positive ion mode and 3,964 features in negative ion mode. Included in both numbers are 57 features that were found in both ionization modes. Each feature is associated with 12 abundance values (i.e., three for each set of triplicate analyses of the four pipeline samples). Features with an abundance below <1,000 i.u. in all four samples were removed from further analysis. Below this abundance value, the probability of detection decreases substantially, as determined from a log/log plot of the number of detected features as a function of the peak abundance (Suflita et al., 2016). The remaining 5,465 features, of which 3,041 were detected in all four samples, ranged in abundance by more than four orders of magnitude among the samples. The reproducibility of the abundance measurements in this set of features was excellent as demonstrated by the observation that for ∼90% of the triplicate measurements the distribution of relative standard deviations was in agreement with what is expected based on ion counting shot noise alone (Suflita et al., 2016; data not shown).

When comparing the HC with the LC system, the sum of the abundances of Pigs 3 and 11 were used. The ratio of the abundances in the two systems ranged over more than six orders of magnitude; however, for more than 50% of the features, the abundances in the two systems were similar, differing by a ratio smaller than a factor of 3 (**Supplementary Figure S5**). However, more than 2,000 features were over- or under-represented in one or other of the systems by a factor of three or more. It is thus apparent that the metabolic activities in the two systems are very different.

Of particular interpretive value are those features that both have high abundance and exhibit large abundance differences among samples. Using the criterion of a minimum abundance (>10,000 i.u.) in at least one of the pipelines and a minimum abundance ratio of 5, about 400 features were found to be more abundant in the HC system, versus about 220 features that were more abundant in the LC system.

**FIGURE 4:**
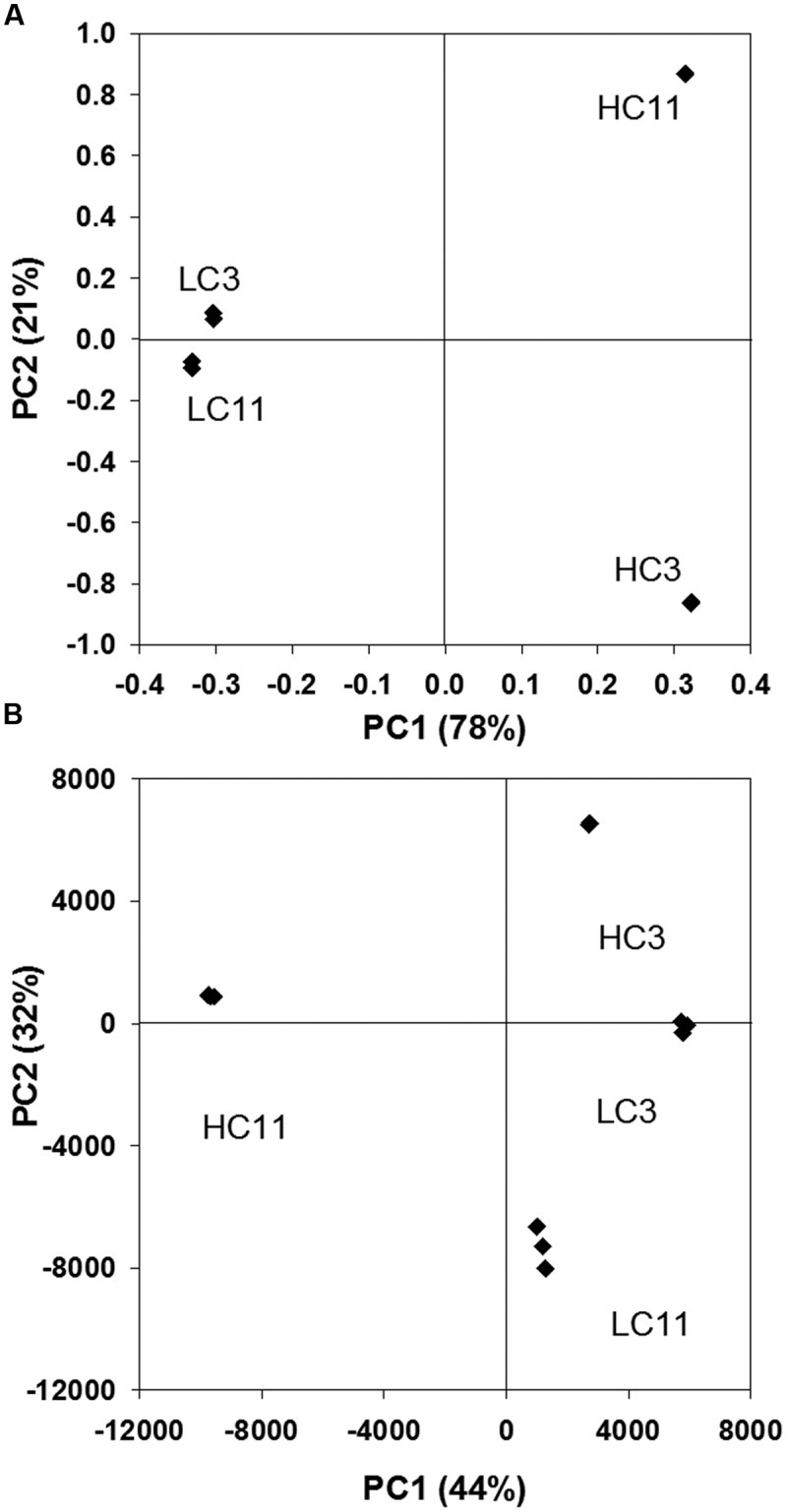
**Principal component analysis results (PCA) showing principal components 1 and 2 for** **(A)** metagenomic DNA sequence reads as determined via MG-RAST; and for **(B)** metabolomic data from each triplicate analysis of each of the four samples. Only features with an abundance of at least 10,000 in one of the four samples were included.

The relative similarities and differences between the metabolomes of the four samples are illustrated by the result of a principal component analysis (PCA; **Figure 4B**). The figure shows that all four metabolomes were distinctly different, though LC3 and LC11 are the pair of samples most similar to each other. The PCA pattern in **Figure 4B** was very robust. For example, while only abundant features (>10,000 i.u. in at least one sample) were used for this analysis, a very similar graph resulted when including all 5,465 features or whether a linear or logarithmic abundance scale was used. The notable exception was that the relative similarity of the HC3 and LC3 metabolomes was shown to be largely due to the presence of high-abundance compounds. Most of these were not identified in the KEGG database and, presumably, they were not of microbial origin, but likely derived from injected seawater, extracted petroleum, and/or industrial chemicals added to the systems.

Of the 5,465 features retained for analysis, 2,352 were putatively matched with metabolites in the KEGG database (**Supplementary Table S7**). The percentage of features that was putatively identified with known metabolites was 43% overall, but it was substantially different in the four samples. Thus, for the 200 most abundant features in each respective sample, the ratios of identified to non-identified features were 1.47, 0.87, 0.80, and 0.35 for HC11, HC3, LC11, and LC3, respectively. Among the 2,353 putative metabolites, peptides and lipids were the numerically dominating compound groups, comprising 671 di-, tri-, and tetra-peptides and 590 lipids (sterol lipids, sphingo-lipids, prenols, polyketides, glycerophospholipids, and fatty acyls). While peptides were more numerous, lipids contributed ∼4X as much as the peptides to the total metabolite abundance. A large group of putatively identified metabolites (247) were mapped to “housekeeping” processes (**Supplementary Table S8**), including metabolism of amino acids (168) and carbohydrates (37). Secondary metabolism was represented by 110 putative compounds, and a total of 735 putatively identified metabolites were not associated with any specific metabolic pathway in the KEGG database. The “richness” of the HC metabolome was largely due to a wide variety of high-abundance lipids. Other putative metabolites preferentially present in the HC system were involved in biosynthesis and/or metabolism of steroid lipids, phenylpropanoids, porphyrins, arachidonic acid, unsaturated fatty acids, taurine, and isoprenoids. Further, iron ions were detected at a high abundance in both HC samples, and they were four times more abundant in HC11 than in HC3.

Given the dominating presence of petroleum in the pipelines, targeted searches were also performed for compounds that are indicative of known aerobic and anaerobic hydrocarbon degradation pathways (**Supplementary Table S8**). A number of both aerobic and anaerobic degradation products were detected. The metabolites with abundances above 5 × 10^3^ are listed in **Table 5**. Alkylsuccinates and benzylalkylsuccinates are of particular importance as they are strongly indicative of anaerobic degradation of alkanes and aromatics via addition to fumarate (Agrawal and Gieg, 2013; Rabus et al., 2016) respectively, and these compounds were identified using standards. A wide range of both alkylsuccinates and alkylbenzylsuccinates were detected in all samples. The corresponding extracted ion chromatograms can be found in **Supplementary Figure S6**. Signatures consistent with carboxylated phenanthrene were also observed suggesting potential for anaerobic aromatic ring carboxylation, primarily in the HC samples.

**Table 5:** Selected hydrocarbon degradation products detected in the LC and HC systems (EIC of the corresponding ions are reproduced in **Supplementary Figure S6**).

Degradation product	Pathway/substrate^1^		Formula	Experimental mass (Da)	Mass error (mDa)	Retention time (min)	High abundance in samples	Abundance/i.u.
Ethylsuccinic acid	AN	Ethane	C_6_H_10_O_4_	146.0579	0.1	1.20	HC11	162000
Propylsuccinic acid	AN	Propane	C_7_H_12_O_4_	160.0735	0.2	1.67	HC11	39000
Butylsuccinic acid	AN	Butane	C_8_H_14_O_4_	174.0892	0.3	2.51	HC11	11500
Pentylsuccinic acid	AN	Pentane	C_9_H_16_O_4_	188.1045	0.3	4.12	HC11	42000
Hexylsuccinic acid	AN	Hexane	C_10_H_18_O_4_	202.1205	0.4	5.98	HC11	15400
1-Phenylethyl-succinates or methyl benzylsuccinate	AN	Ethylbenzene or xylene	C_12_H_14_O_4_	222.0892	0.0	4.6	HC11	1080000
Methylbenzylalcohol	A	Xylene	C_8_H_10_O	122.0732	-0.5	15.17	LC11	175000
Dimethylcatechol or isomers	A	Ethylbenzene	C_8_H_10_O_2_	138.0681	1.1	5.99	LC11	9000
		or					LC3	6600
		xylene					HC3	7100


*^1^ AN, anaerobic and A, aerobic degradation pathway.*

## Discussion

The problem addressed in this study is the observation that different rates of corrosion occur in two very similar oil pipeline systems in adjacent fields in the North Sea, and the ultimate objective is to approach an explanation for this observation. Both metagenomic and metabolomic analyses point to strong microbial activity in both systems. This is consistent with aggressive MIC observed in the HC system, but does not explain the relatively low rates of corrosion in the LC pipeline. The physico-chemical characteristics of produced waters in both systems are very similar (**Table 1**) suggesting that abiotic processes may not be the differentiating factor, but rather that differences in corrosion rates are rooted in the biological activity occurring in the two systems.

### Community Structure

Data described here reveal fundamental differences in the microbial community structure and metabolism associated with LC and HC pig envelopes. With respect to the metabolome, chemicals detected in oil-field systems have both microbial and non-microbial origins. The latter group (referred to as “exochemicals” here), originate from seawater, petroleum, and industrial equipment or are added industrial chemicals (e.g., as corrosion inhibitors). Using the ratio of metabolomics features identified via comparison with catalogs of biological molecules (i.e., KEGG) to non-identified features might thus be a useful measure of the relative importance of microbial metabolites versus exochemicals. This analysis indicates that a significantly larger fraction of the features detected in the HC samples (55%) were putatively identified as known metabolites than in the LC samples (32%). For the 200 most abundant features in each respective sample, the ratios of identified to non-identified features were 1.47, 0.87, 0.80, and 0.35 for HC11, HC3, LC11, and LC3, respectively. In addition to indicating a more dominant microbial signal in the HC system, biological contributions also appear more prominent in later pigging samples (i.e., pig run 11 vs. 3), perhaps reflecting the physical structure and densities of microbial biofilms associated with the pipeline walls.

With respect to the metagenomic data, a sharp differentiation between LC and HC samples is reflected by apparent differences in the potential for terminal electron acceptor utilization by dominant community members. This is despite that fact that nitrate addition to injection water had occurred in both systems for several years. Many different bacterial groups have been associated with MIC, including SRB, iron oxidizers, and manganese-oxidizing bacteria (Little and Lee, 2007), though sulfate reducers in particular are often the focus of corrosion management due to the corrosivity of sulfides. In the HC samples, >40% of genome equivalents included the capacity to respire sulfate (**Figure 3**), and the dominant binned genome contained the genes for dissimilatory sulfate reduction. Overall, the features encoded by this genome bin were most similar to the genome of *Desulfonatronospira thiodismutans* (**Table 2**), which is known for high sulfidogenesis under haloalkaline conditions (Sorokin et al., 2008). The type strain of *D. thiodismutans* can perform sulfite/thiosulfate dismutation to sulfide. It can also grow both autotrophically with formate or hydrogen, and utilize short chain organic molecules such as lactate, pyruvate, ethanol, and acetate, which are present in high concentrations in HC production waters (**Table 1**). The second most abundant taxon in the HC metagenomic data was a genome bin most similar to the archaeon *Thermococcus kodakarensis*, which is known for sulfidogenic, heterotrophic growth on organic substrates in the presence of elemental sulfur (Atomi et al., 2004). We note that neither aerobic nor anaerobic hydrocarbon degradation genes were detected in the HC genome scaffolds. HC metagenomic and physiochemical data are therefore consistent with sulfur cycling that is dependent on short chain organic acids, but not hydrocarbons. Conversely, only a small population of sulfate reducers (<5% of cells) was present the LC samples, and by far the most abundant bacterial population was most closely related to *Pseudomonas stutzeri. P. stutzeri* is well known for its ability to reduce nitrate, and requisite genome bins contained the necessary genes for the dissimilatory nitrate reduction machinery (**Supplementary Table S4**). Also present in the LC *P. stutzeri* genome bins are a large diversity of mono- and di-oxygenases related to hydrocarbon utilization. Similarly, this is a feature common to *P. stutzeri* strains, which have been shown to be involved in the utilization of a range of hydrocarbons (Lalucat et al., 2006). LC microbial communities therefore appear to be supported by dissimilatory nitrate reduction, potentially coupled to the utilization of oil hydrocarbons.

Principal component analysis analysis (**Figure 4**) indicates that the patterns of overall differences in the metagenomes and the metabolomes of the four samples analyzed here are quite similar, with the exception that the LC3 and LC11 metagenomes are relatively more similar than the two corresponding metabolomes. This does not necessarily mean that the metabolism of the LC microbial community changes with increasing depth into the deposits, but may simply reflect that the concentrations of oilfield- and other exo-chemicals decrease with increasing depth into the deposits.

### Metabolomic Analysis of Hydrocarbon Degradation Pathways

A targeted search of more than 200 hydrocarbon degradation products reported in the literature was conducted in an effort to identify degradation pathways and to determine whether degradation occurred under aerobic and/or anaerobic conditions. Although, fewer positive identifications were made than expected, meaningful results were obtained where standards were available (**Table 5**). Particularly interesting in this context was the presence of several alkylsuccinates and benzylsuccinates, which are diagnostic for anaerobic hydrocarbon degradation (Agrawal and Gieg, 2013; Rabus et al., 2016). Requisite succinates were more than an order of magnitude more abundant in HC11 than in any other sample. Although this points to anaerobic microbial hydrocarbon degradation in HC, the necessary genetic signatures (i.e., alkyl- and benzyl-succinate synthase genes), which are diagnostic of hydrocarbon degradation via addition to fumarate (Callaghan et al., 2010), were not observed in the metagenomes (**Table 4B**). This discrepancy might potentially be explained by several properties of the system. Anaerobic hydrocarbon degraders may be present in the HC sample, but their abundance may have been too low to be detectable in metagenomic data given the sequencing depth applied here. Alternatively, anaerobic degradation metabolites may reflect processes in the source formation but not the pipeline. It is also possible that HC bacterial communities contain succinate-forming metabolic pathways for which the genetic signatures are unknown. More likely, however, the data are consistent with a relatively minor contribution of anaerobic hydrocarbon degradation to the overall microbial metabolism in the HC samples.

Detectable aerobic degradation products included hydroxylated derivatives of xylene, ethylbenzene, naphthalene, and phenanthrene. These were limited to the LC system and their relative abundance was greater in the later (LC11) vs. the earlier (LC3) pig sample (**Table 5**), which is consistent with the observed distribution of aerobic hydrocarbon degradation genes (see above). Thus, hydrocarbon degradation may have preferentially occurred deeper into the wall deposits. In the outer layer of the deposits, substrate requirements may well be met by diffusion from the water phase of partially oxidized compounds such as volatile fatty acids, which are preferentially used for oxidation. The role that biofilms play in the degradation of hydrocarbons in pipeline systems is, however, still poorly understood. It is, for example, unclear how requisite deposits were structured. Outer layers of the biofilm may have been dominated by abiotic products and the patterns observed here may simply reflect the distribution of biomass.

### Organonitrogen Metabolism

Denitrification, as a strictly inorganic process, is not directly targeted by conventional metabolomic analysis. Nitrate (NO_3_^-^; m/z 62) is detectable in the negative ion mode, but wasn’t found in any of the samples. Quite apart from the inorganic compounds involved, the biochemical denitrification process is likely associated with unique metabolic signatures (Kampschreur et al., 2012). The related signals, however, must be characterized using laboratory cultures before they can be reliable identified in metabolic data of complex communities. Our ability to extract requisite signatures related to denitrification or sulfate reduction from the LC and HC metabolomes, respectively, are therefore limited. With high-resolution mass spectrometry, it is, however, possible to study both patterns and abundance of nitrogen-containing organic compounds without the need for identifications. Organic compounds that contain an odd number of nitrogen atoms are unusual in that they have an odd integral mass (McLafferty and Turecek, 1993). The relative abundance of odd molecular masses in the metabolome, RN (Equation 1: RN = $\frac{\Sigma \,\text{Abundances of features derived from odd-mass molecules}}{\Sigma \text{Abundances of all features}}),$ might therefore be used as a measure of the importance of organonitrogen compounds, and presumably their metabolism, in the metabolome. While this assumption is hypothetical, a striking pattern is indeed observed: While RN values are similar in LC3 and HC3 at ≈40%, the value increases substantially with depth of deposits in the LC system to 55% in LC11, but decreases in the HC system to 30% in HC11. Even more strikingly, the RN value for those features that were given putative identifications was only 24% in HC11, while it was 62% in LC11. These observations are consistent with a more dominant role for the metabolism of nitrogen-containing organic compounds and the accumulation of such compounds in the LC deposit. What role, if any, denitrifying organisms such as *P. stutzeri* play in this remains unclear from our data. The observation may nonetheless present future opportunities for the development of predictive capacity with respect to corrosion, by correlating metabolomic profiles to microbial community structure and their corrosivity.

### Microbial Community Resilience

A notable feature of the HC and LC systems is the observation that dramatically different microbial community organizations are maintained over many years, even though both the physico-chemical drivers and chemical field treatments are very similar. While it is possible that there are significant, but overlooked, environmental differences between the two systems, it is also possible that a biological mechanism dominates. We note that at the time of sampling nitrate treatment of injection water was occurring in both systems, but while seawater injection in HC was initiated in 1999, nitrate treatment did not start until 2001. This could suggests that the HC system retains a corrosive community structure due to its inherent resilience to disturbance via requisite nitrate treatments. Similarly, the LC system might contain persistent denitrifying community assemblages that resist invasion by more corrosive SRBs. Ecological resilience theory indicates that communities can often assemble in many different stable states (Botton et al., 2006; Shade et al., 2012). Alternate community states often persist for no other reason than their historical context and resilience to disturbance. Whether nitrate treatment should be considered a severe, potentially community-transforming, “disturbance” for the established sulfate-reducing HC community is not at all obvious with the data at hand.

Survival of sulfate reduction metabolism in the HC system may seem surprising in view of the long-term nitrate treatment. For example, it has previously been reported that the addition of nitrate not only inhibits sulfate reduction but also leads to the reduction of sulfide already present in a biofilm (Halim et al., 2012). Similarly, nitrate reducing bacteria (NRB) appear to outcompete SRB for access to degradable oil organics (Hubert and Voordouw, 2007). Using a packed-bed bioreactor, the same study found that souring control in a reactor that received 12.5 mM lactate required the addition of 10 mM nitrate, irrespective of the sulfate concentration. Thus, if the (usable) oxidative capacity of organics, such as lactate or VFAs, is larger than the reductive capacity of nitrate added to the injection water (da Silva et al., 2014), most of the organics will remain in the water phase by the time the denitrification process is completed, creating ideal conditions for sulfate reduction. This is not inconsistent with observations from the LC and HC systems. Acetic acid concentrations were as high as 5.2 mM (**Table 1**), while nitrate additions were ca. 2.5 mM, potentially stoichiometrically insufficient to allow complete VFA depletion.

One may also consider that NRB are known to suppress SRB activity, not only by competition for electron donors, but also by producing nitrite, which inhibits sulfite reduction by binding to the dissimilatory sulfate reductase (DsrAB) enzyme (Haveman et al., 2004). In thin biofilms, nitrate and nitrite may penetrate by diffusion, offering a possible explanation for observations in experiments that demonstrate nitrate inhibition, even when nitrate levels would not allow the stoichiometric depletion of available carbon sources. In thicker, more stable biofilms, however, such as the ones frequently found in oil pipeline systems, requisite inhibition might not be observed, and the exact mechanism of SRB inhibition by NRB is likely to be more complicated. Other factors that might influence this interplay are the history of the system, the rates of transport, and the size and geometry of a system. Inoculation with nitrate reducing-sulfur oxidizing bacteria are effective in suppressing souring in experiments performed in serum bottles (Nemati et al., 2001) but might not be effective in packed-reactors or in thick pipeline deposits.

### Corrosion Mechanisms

It is in the profound differences in metabolic activity and organization between the two microbial systems that the explanations for the differences in corrosion rates are likely rooted. Aspects of the metabolic conditions in the HC systems that would seem to be particularly relevant to aggressive corrosion include the accumulation of sulfides and/or H_2_S, greater biomass, the presence of fermentation products, and the generation of a unique chemical environment.

Sulfides and H_2_S are traditionally associated with MIC corrosion, yet 80 years after the cathodic depolarization theory was first articulated (von Wolzogen Kuhr and van der Vlugt, 1934) the corrosion mechanisms occurring in the field are still disputed (Beech and Sunner, 2007; Enning and Garrelfs, 2014). It is assumed that the presence of sulfides affects the stability of protective oxide layers. However, the sulfide layers that replace them are sometimes protective (Ning et al., 2015). Sulfide layers may also catalyze reduction reactions, detected as cathodic depolarization (King and Miller, 1977). The ratio of sulfide generation by reduction and iron ion generation by corrosion will determine the extent to which free H_2_S or HS^-^_aq_ is formed. The dominant role of sulfate reduction in the HC samples, and the fact that both samples had a very strong odor of H_2_S, support that free H_2_S is indeed formed in HC. Hydrogen sulfide is, independently of other sulfides, recognized as a corrosion agent in the oilfields (Zheng et al., 2015), and significant enhancements of both cathodic and anodic currents on (X65) pipeline steel can be observed in its presence (Zheng et al., 2014).

Lastly, anaerobic fermentation may have been an important contributor. Fermentation produces a range of low-molecular weight organic compounds, such as acetate, which was observed in high concentration in produced waters. Furthermore, the potential for fermentative processes is somewhat supported by the observation that two of the dominant genome bins were most similar to Firmicutes lineages, which are well known for their fermentative metabolism. The genomic content of the more abundant of these bins was most closely related to the genome of *Thermoanaerobacter pseudethanolicus*, which is known to ferment simple sugars, starch, and cellobiose (Onyenwoke et al., 2007). However, *T. pseudethanolicus* is also known to respire via the reduction of thiosulfate to H_2_S, and it may have therefore been a contributor to sulfidogenesis. This distinction is likely dependent on the availability of suitable electron acceptors, but cannot be directly assessed via metagenomic analysis. The other dominant Firmicute genome bin is most similar to the genome of *Alkaliphilus metalliredigens*, which is best known for its ability to reduce iron in the presence of organic substrates (Ye et al., 2004), rather than fermentative metabolism. Metagenomic data are therefore not necessarily supportive of the notion that fermentative metabolism was a driving force in the corrosion of HC.

## Conclusion

A combined metagenomic and metabolomic approach was used to investigate significant differences in the severity of corrosion observed in two, otherwise very similar, production pipelines. Distinct microbial communities with dramatically different metabolic and genomic signatures were observed, with notable differences in the complexity and abundance of organonitrogen metabolites, the potential to utilize nitrate or sulfate as terminal electron acceptors, and the ability to degrade hydrocarbons. These results are not easily reconciled when it is assumed that nitrate addition is the primary driver (or disturbance) of community assembly. Put another way, it is typically expected that the addition of nitrate will create a push disturbance that, due to changes in redox potential, allows less-corrosive NRBs to outcompete SRBs. Our data indicate, however, that nitrate addition might sometimes result in an insufficient push, and that a lasting alternate stable community structure is not always achieved in the face of nitrate treatments. The problem of corrosion management in pipelines like HC may therefore be as much about assessing stability and creating the right kind of disturbance as it is about changing the redox potential. It is likely that localized, if not systemically occurring, biofilms will grow to sufficient thickness between pigging operations to allow the creation of nitrite-free locales in which SRB activity will have opportunities to persist. This would explain why nitrate addition has been reported to be effective in cultures as well as reservoirs, but does not always produce the desired results in pipeline systems. At this point, it therefore remains unclear how it is possible to effectively suppress SRB activity in production systems in which significant biofilms and abiotic deposits form. We suggest that corrosion management approaches and attempts to understand corrosion in complex engineered systems might benefit from a more refined understanding of the forces related to community resilience and the impact of disturbances such as pigging and nitrate addition.

## Author Contributions

VB conducted MS analyses and assisted with data analysis and writing. BW was responsible for molecular data QC, bioinformatics analysis, and python code. BW took the lead in writing the manuscript and data analysis. JS took the lead in metabolome data analysis and contributed to the writing of the manuscript. ES was involved in the bioinformatics analysis presented in the paper. EA assisted in MS analyses. KD was involved in sample processing, DNA extraction, qPCR analysis, and 16S rRNA gene sequencing as well as editing the manuscript. AC contributed to metagenome analysis for aerobic and anaerobic hydrocarbon degradation genes by providing reference databases for gene searches. AC also guided the analysis of metabolome data with respect to microbial hydrocarbon degradation by providing search targets for metabolomic surveys. AO (Duncan Lab) was involved in sample processing, DNA extraction, qPCR analysis, and 16S rRNA gene sequencing as well as editing the manuscript. TL was responsible for sampling in the field and metadata collection. IB initiated the project, assembled the team, and contributed to the ideas and writing.

## Accession Numbers

Metagenomic reads can be found in NCBI’s Short Read Archive. BioProject: PRJNA356928; BioSamples: SAMN06130235 and SAMN06130234.

## Conflict of Interest Statement

The authors declare that the research was conducted in the absence of any commercial or financial relationships that could be construed as a potential conflict of interest.

## Footnotes

1 www.arb-silva.de

2 rast.nmpdr.org
