# Supplementary material for: Metabolomic and Metagenomic Analysis of Two Crude Oil Production Pipelines Experiencing Differential Rates of Corrosion
Source: Front Microbiol. 2017 Jan 31;8:99. doi: 10.3389/fmicb.2017.00099 (PMC5281625; doi:10.3389/fmicb.2017.00099)
Supplement: Supplementary file 1 [file Data_Sheet_1.PDF]

Figure S1.

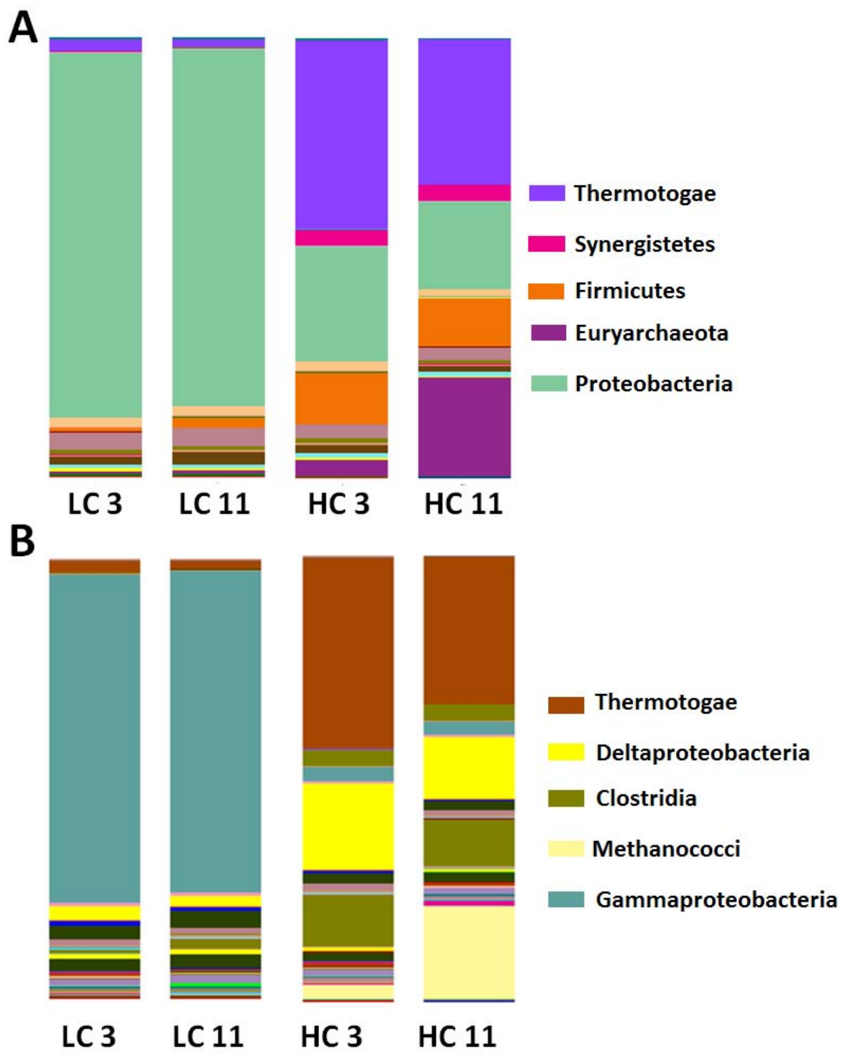

Figure S1. Phylogenetic breakdown of 16S rRNA gene sequences at the (A) phylum level and (B) class level. Partial 16S rRNA gene PCR products were sequenced after barcoding and multiplexing using an Illumina MiSeq. Data were then analyzed using the QIIME pipeline (Caporaso et al. 2010b). Operational Taxonomic Units (OTUs) were generated using UCLUST at the 95% identity level. Alignment to the SILVA small subunit rRNA reference alignment (www.arb-silva.de) was used for classification.

Figure S2.

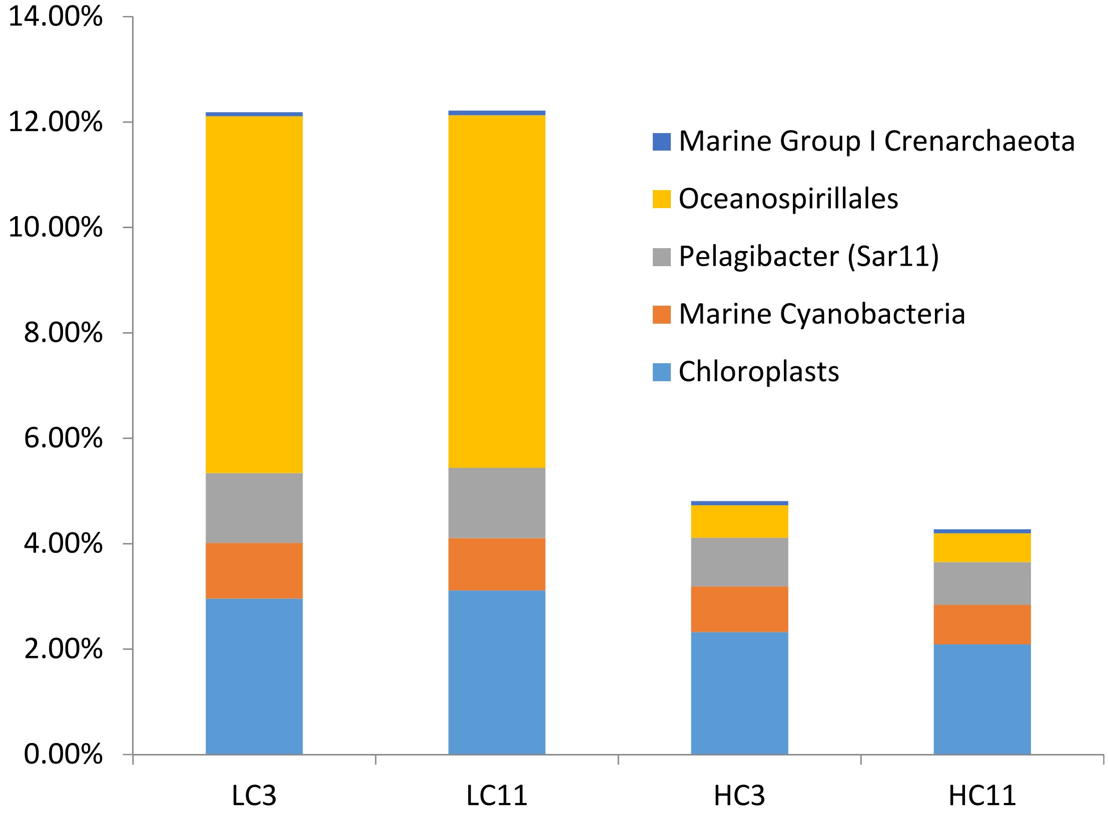

Figure S2. Frequency of diagnostic epipelagic marine taxa in 16S rRNA gene sequence data. Requisite taxa are common to marine surface waters and are not known to exist in subsurface environments or produced waters. The chloroplast signal originates from a range of Chlorophyte and Chromealveolate marine algal lineages including Heterokonts, Haptophytes and Dinoflagellates, which are similarly diagnostic of marine surface waters.

Figure S3

A

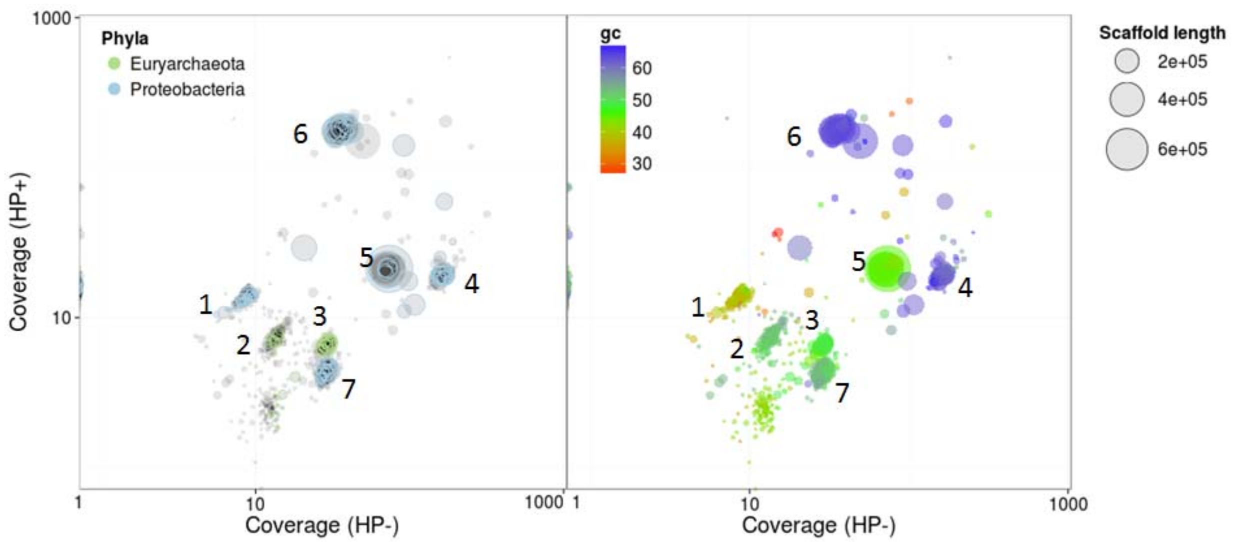

B

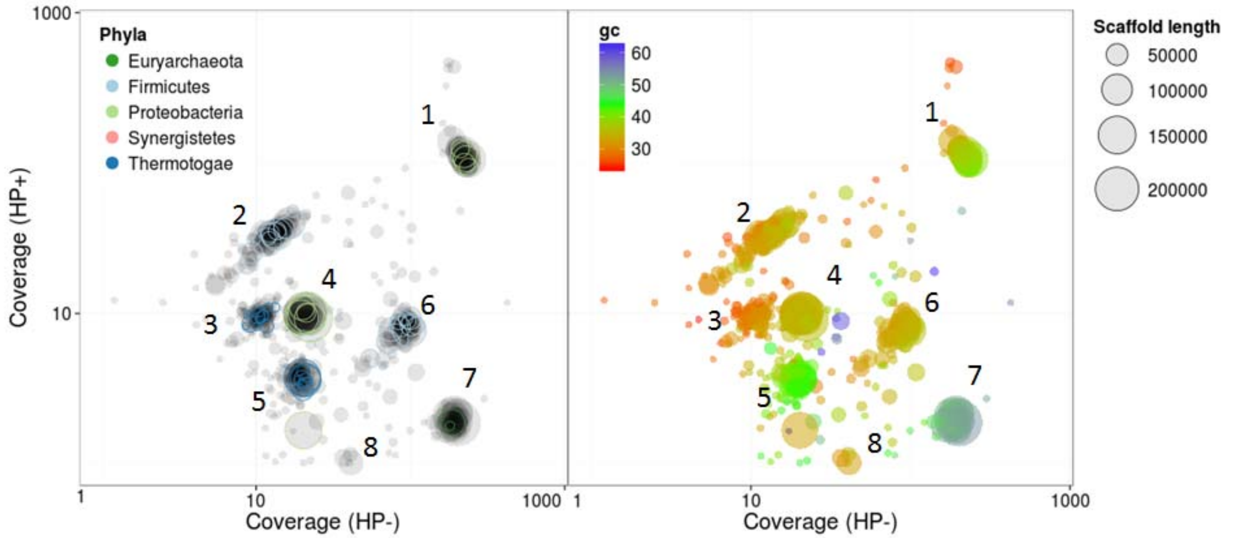

Figure S3. Graphical representation of metagenome data produced via Multimetagenome analysis (Albertsen et al. 2013). Two closely related metagneomes are analyzed in this analysis. Here, the LC3 vs LC11 (A) and HC3 vs HC11 (B) metagenomes were compared. Each contigs frequency is assessed via direct mapping of reads via Bowtie2 to determine coverage (x- and y-axes). HMM analysis of core housekeeping genes is used to determine the phylogenetic affiliation of individual contigs (left panels). Using this, as well as the GC content of contigs (right panels), groups of contigs are identified and extracted. Contig groups are further interrogated via PCA analysis of tetra-nucleotide frequencies to generate final bins (not shown).

Figure S4

a.

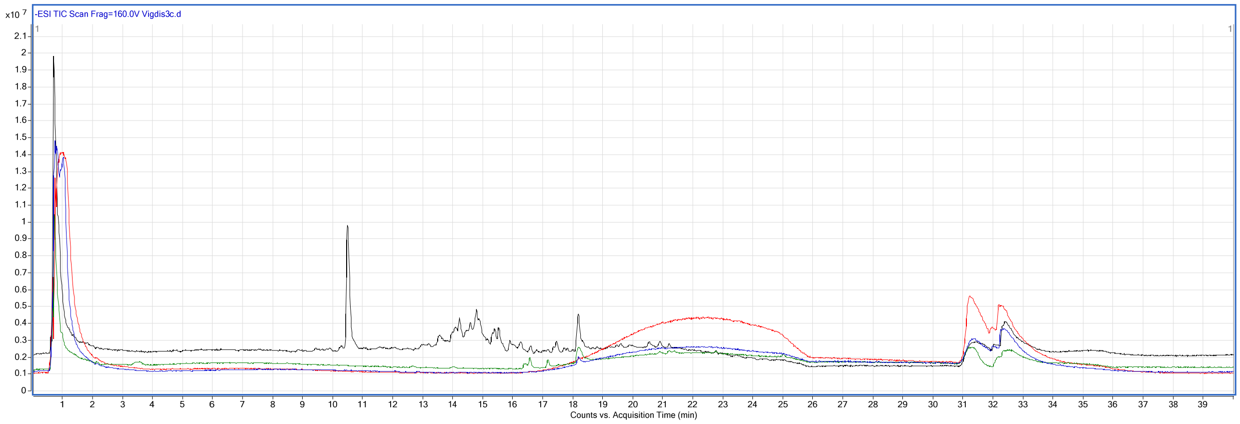

b.

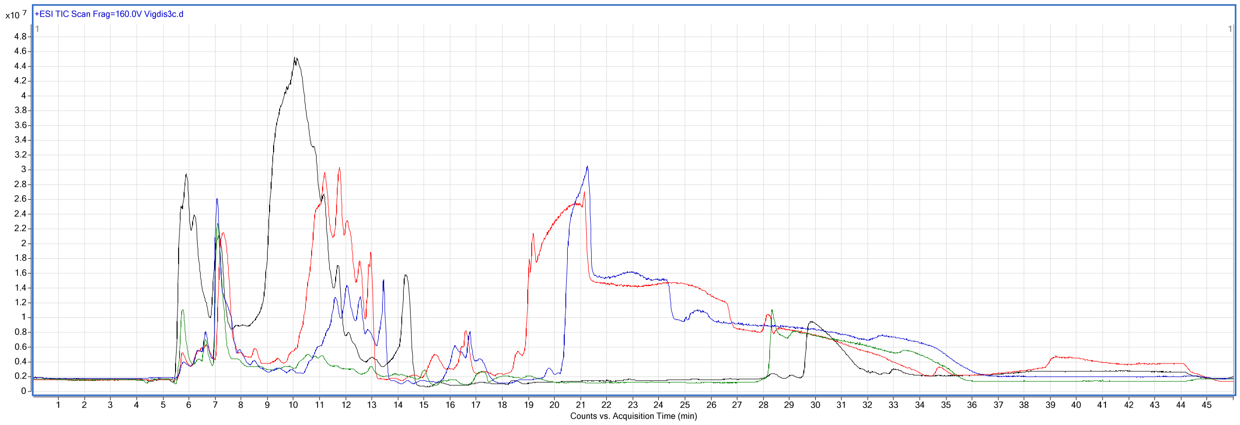

Figure S4. TIC of HC11 (black), HC3 (red), LC11(green) and LC3 (blue) from (a) from UPLC-HRMS ESI negative ionization mode and a C18 chromatographic column; (b) UPLC-MS positive ionization mode and ZIC-HILIC chromatographic column

Figure S5.

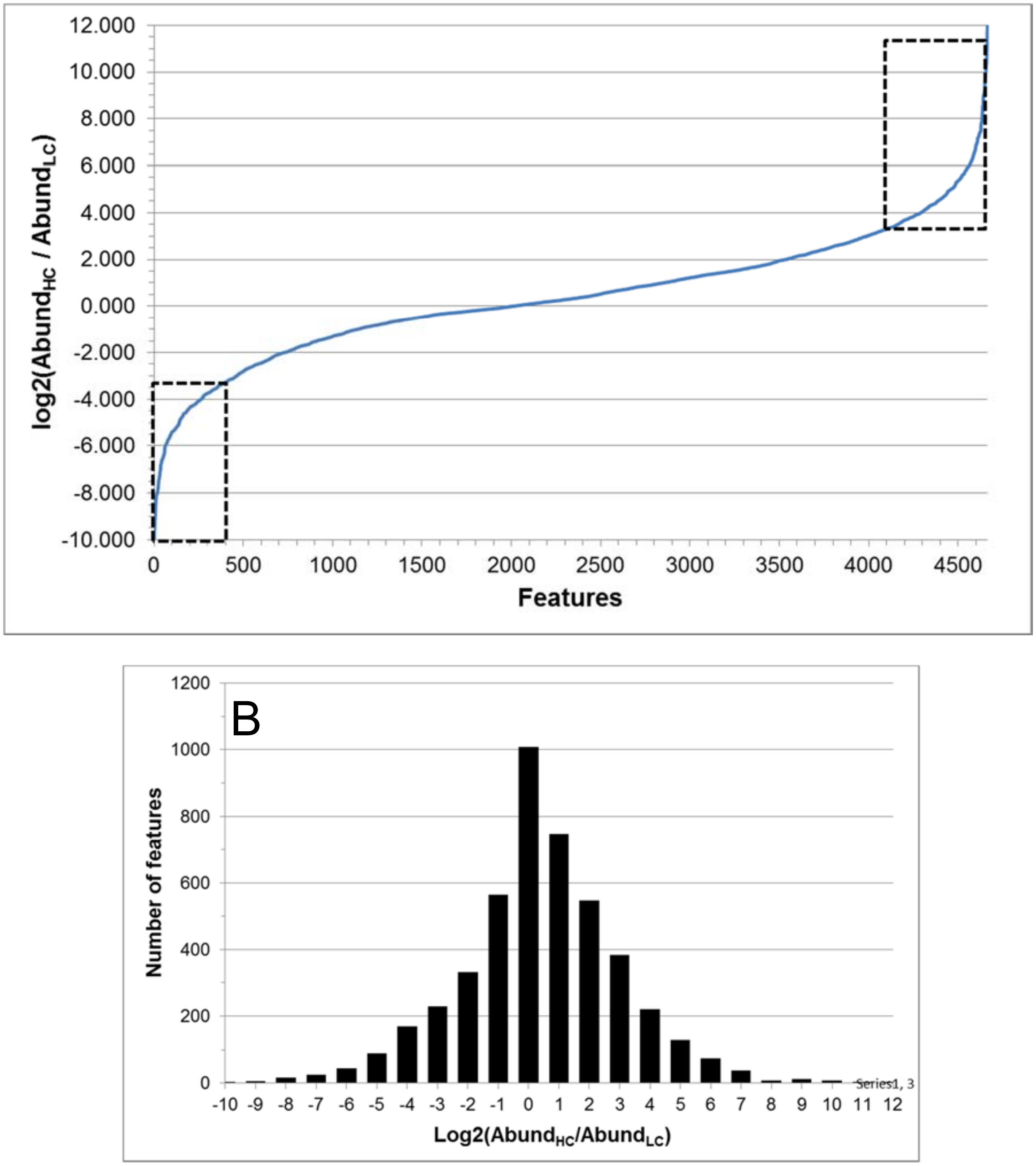

Figure S5. Distribution of log2 ratios of abundances of features in HC versus LC samples. A) The lower left rectangle encompass the 389 features that have a more than 10 times higher abundance in the combined LC samples than in the combined HC samples, and the upper right rectangle the 561 features that similarly are more than 10 times more abundant in the HC samples. B) The frequency distribution of log2 abundance ratios reveals a gradual distribution. For about 50% of the features, the abundance ratio is between 1/3 and 3/1.

Figures S6

a) $(M\text{-}H)^-$ ion of $C_6H_{10}O_4$ (ethylsuccinic acid)

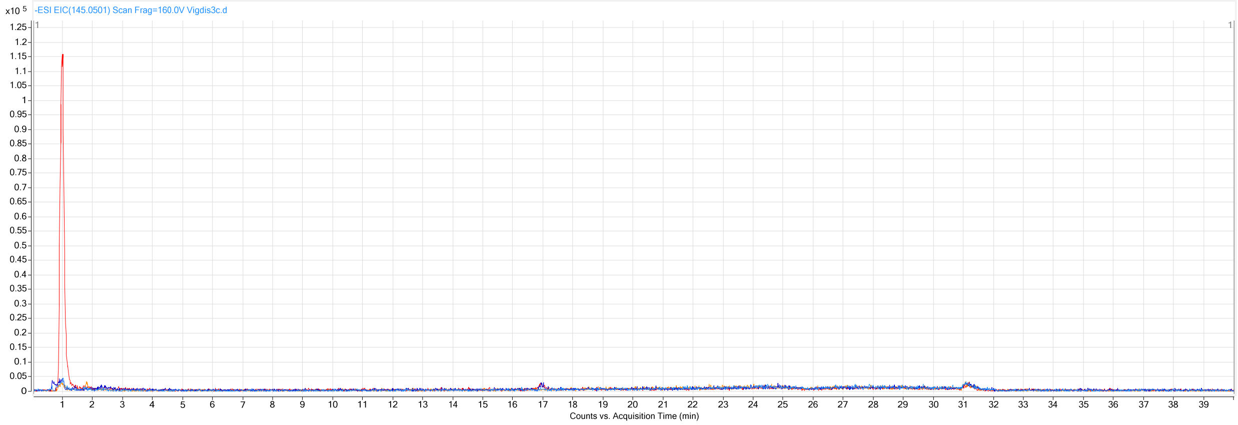

b) $(M\text{-}H)^-$ ion of $C_7H_{12}O_4$ (propylsuccinic acid)

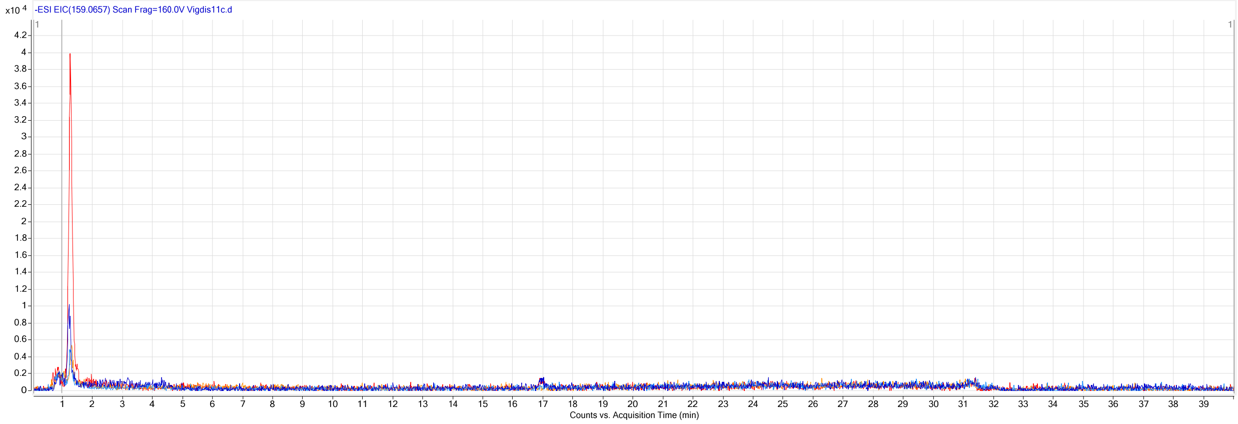

c) $(M\text{-}H)^-$ ion of $C_8H_{14}O_4$ (butylsuccinic acid)

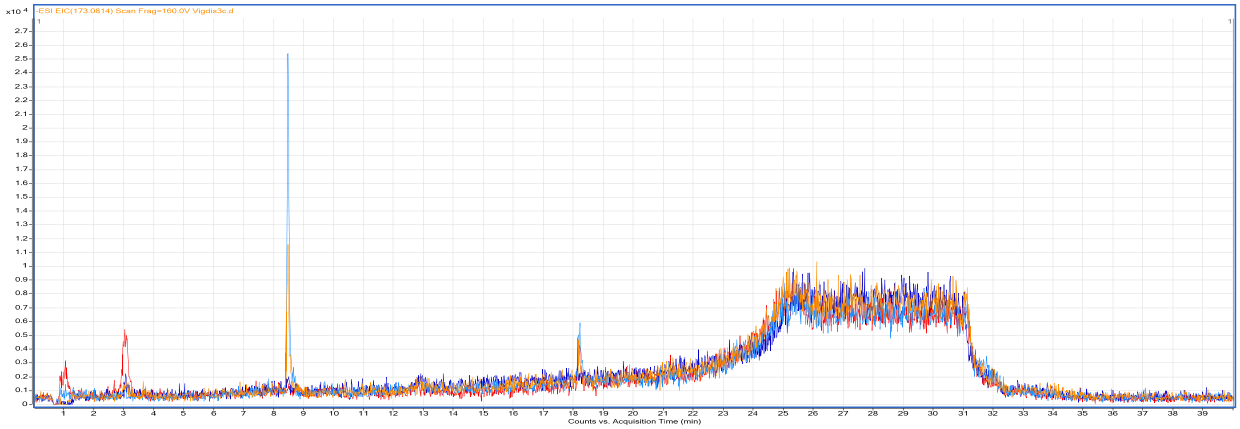

d) $(M\text{-}H)^-$ ion of $C_9H_{16}O_4$ (pentylsuccinic acid)

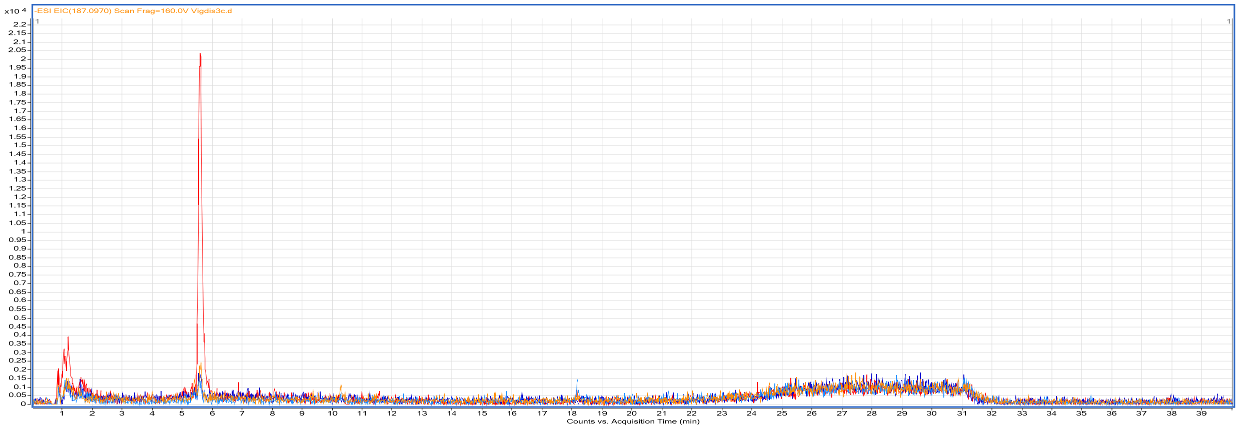

e) (M-H)$^-$ ion of $C_{10}H_{18}O_4$ (hexylsuccinic acid)

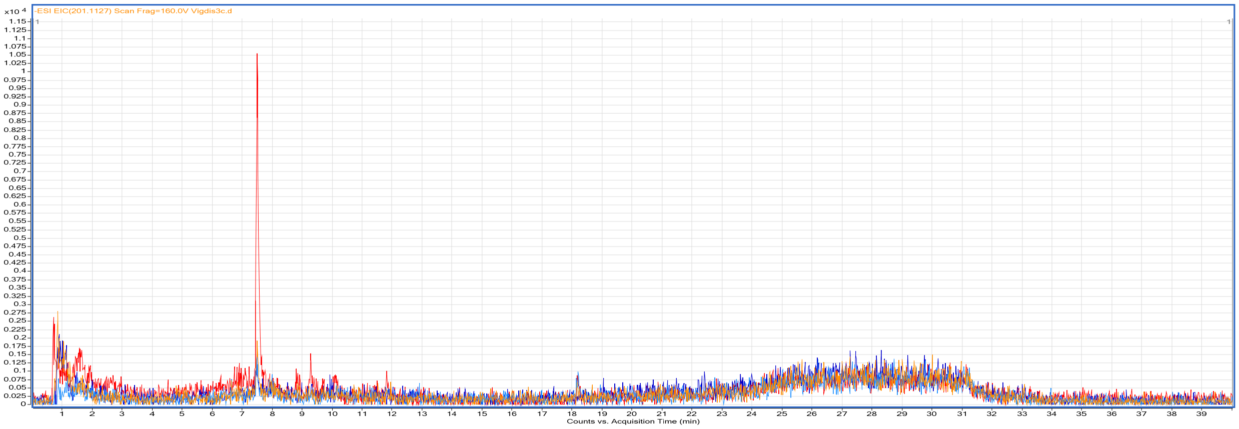

f) (M-H)$^-$ ion of $C_{12}H_{14}O_4$ (1-phenylethylsuccininc acid or methylbenzylsuccinic acid)

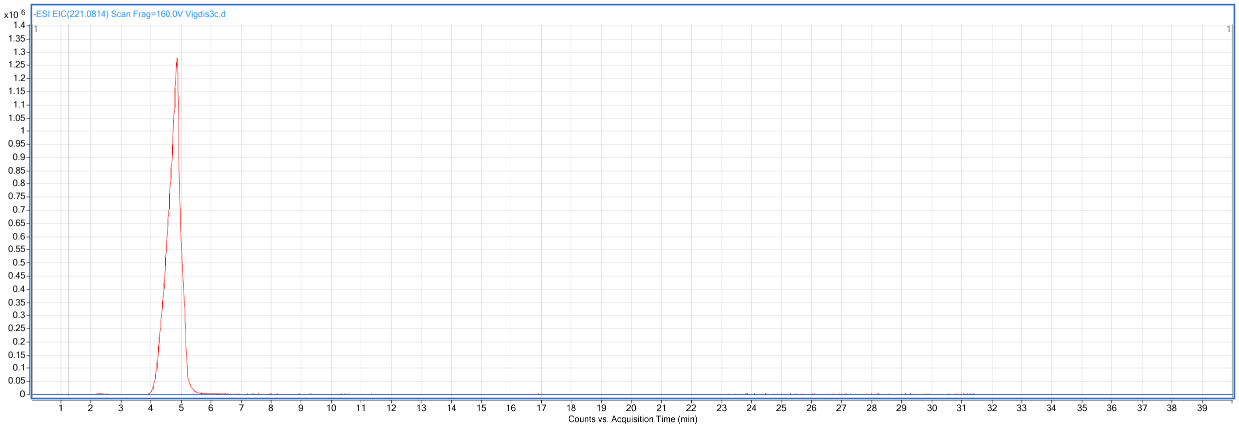

g) MH$^+$ ion of $C_8H_{10}O$ (methylbenzylalcohol)

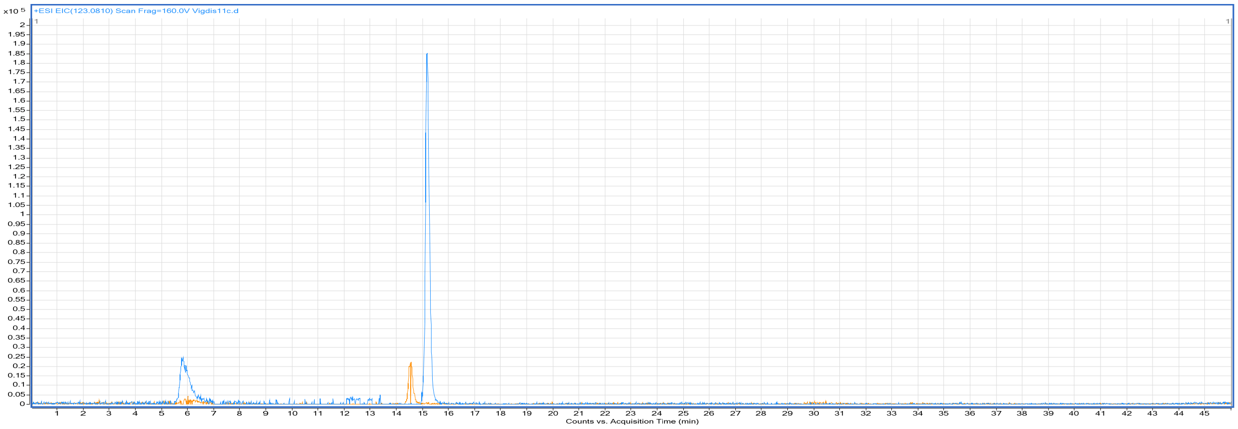

h) MH^+ ion of $C_8H_{10}O_2$ (dimethylcatechol)

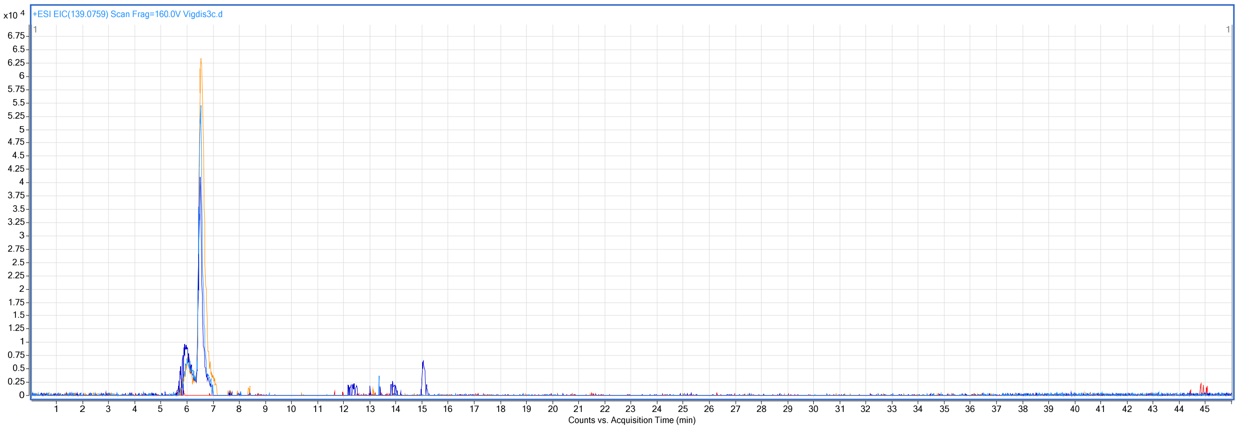

Figure S6. Extracted ion chromatograms (EIC) of $(M\text{-}H)^-$ and MH^+ ions of selected hydrocarbon degradation products for HC11 (red), HC3 (blue), LC 11 (light blue) and LC 3 (orange). For each EIC, all ions of the same mass will be detected, including those derived from the target compound and those derived from any structural isomers. The retention time range is 0 to 40 minutes.

Table S1. Parameter setting for XCMS, mzMatch and for putative identification with known metabolites

XCMS (Centwave)

Method (file type):	mzXML	
ppm:	2	
peak width (min):	5	seconds
peak width (max):	100	seconds
S/N threshold:	3	
Prefilter (# points):	3	
Prefilter (intensity):	1000	
Mzdiff:	0.001	

mzMatch

Mzmatch grouping RT window:	0.5	min
Mzmatch grouping m/z ppm:	5	ppm
**Relative Std Dev (RSD) filter:**	**1.00**	GENEROUS
Noise filter (codadw):	0.50	
**Intensity filter (LOQ):**	**1000**	
**Minimum detections #**	**3**	
RT window for related peaks:	0.20	min

IDEOM

**RT for id of authentic standards:**	**10.0**	**%**
**RT for id for calculated RT:**	**45.0**	**%**
**PPM for mass identification:**	**6.0**	**ppm**
Ignore related peaks before RT:	0.0	min
RT window for complex adducts:	0.50	min
RT window for Duplicatepeaks:	1.00	min
RT window for Shoulderpeaks:	2.0	min
Intensity ratio for Shoulderpeaks:	5	to 1
Intensity limit Duplicatepeaks:	1	%
r2 limit for duplicatepeaks	0.99	
**Preferred DB:**	**Central**	map

Table S2. List of authentic standard used for the calibration of retention time prediction model with ZIC-HILIC chromatography

Metabolites	RT
**Benzoate**	**5.68**
Fumarate	6.21
Pyruvate	6.73
Succinate	6.73
**Uracil**	**8.03**
L-Rhamnose	11.35
**L-Phenylalanine**	**11.84**
L-Tryptophan	12.26
**L-Leucine**	**12.63**
Guanosine	12.86
L-Methionine	13.11
L-Proline	13.96
L-Valine	14.02
D-Gluconic Acid	14.52
D-Glucose	14.97
L-Homocysteine	15.97
**L-Glutamate**	**16**
L-Tyrosine	16.03
L-Cysteate	16.08
Taurine	16.58
L-Threonine	16.66
**L-Aspartate**	**16.69**
L-Alanine	18.51
L-Glutamine	19.02
Creatinine	19.1
L-Asparagine	19.47
L-Serine	19.62
Glycine	19.62
L-Cystine	23.22
**L-Histidine**	**26.25**
**L-Arginine**	**27.29**
L-Lysine	27.67
Thiamin	33.43

Table S3. COGs used for phylogenetic classification of N & S cycling and methanogenesis

FUNCTION	COG	GENE
**Methanogenesis**	1029	Formylmethanofuran dehydrogenase subunit B
	1148	Heterodisulfide reductase, subunit A and related polyferredoxins
	1150	Heterodisulfide reductase, subunit C
	1153*	Formylmethanofuran dehydrogenase subunit D
	1229	Formylmethanofuran dehydrogenase subunit A
	1927	Tetrahydromethanopterin formyltransferase
	1962*	Tetrahydromethanopterin S-methyltransferase, subunit H
	2037	Formylmethanofuran
	2048	Heterodisulfide reductase, subunit B
	2141	Oxidoreductases
	2191	Formylmethanofuran dehydrogenase subunit E
	2218	Formylmethanofuran dehydrogenase subunit C
	3252*	Methenyltetrahydromethanopterin cyclohydrolase
	4054	Methyl coenzyme M reductase, beta subunit
	4055	Methyl coenzyme M reductase, subunit D
	4056	Methyl coenzyme M reductase, subunit C
	4057	Methyl coenzyme M reductase, gamma subunit
	4058*	Methyl coenzyme M reductase, alpha subunit
	4059	Tetrahydromethanopterin S-methyltransferase, subunit E
	4061	Tetrahydromethanopterin S-methyltransferase, subunit C
	4062	Tetrahydromethanopterin S-methyltransferase, subunit B
	4063*	Tetrahydromethanopterin S-methyltransferase, subunit A
**Nitrogen Metabolism**	1140*	Nitrate reductase beta subunit
	1348*	Nitrogenase subunit NifH (ATPase)
	2180*	Nitrate reductase delta subunit
	2181*	Nitrate reductase gamma subunit
	2710*	Nitrogenase molybdenum-iron protein, alpha and beta chains
	4459*	Periplasmic nitrate reductase system, NapE component
	5013*	Nitrate reductase alpha subunit
**Sulfur Respiration**	607*	Rhodanese-related sulfurtransferase
	2046*	ATP sulfurylase (sulfate adenylyltransferase)
	2221*	Dissimilatory sulfite reductase (desulfoviridin), alpha and beta subunits
	2897*	Rhodanese-related sulfurtransferase
	2920*	Dissimilatory sulfite reductase (desulfoviridin), gamma subunit
	4117*	Thiosulfate reductase cytochrome B subunit

*** used for the analysis in Figure 2**

Table S4. Denitrification genes annotated by RAST in genome scaffolds extracted from metagenome data generated via Multi-Metagenome analysis.

SITE	BEST RECRUITMENT	SCAFFOLD	*nirS*	*nirK*	*nosZ*	*norB*	*narG*
LC	*Desulfonatronospira thiodismutans*	1					
	Thermococcus kodakarensis	2					
	Archaeoglobus fulgidis	3					
	Pseudomonas stutzeri	4	√		√	√	√
	Methylophaga thiooxidans	5					
	Pseudomonas stutzeri	6	√	√	√	√	√
	Shewanella amazonensis	7			√		
HC	*Desulfonatronospira thiodismutans*	1					
	Alkaliphilus metalliredigens	2					
	Thermosipho africanus	3					
	Desulfohalobium retbaense	4					
	Kosmotoga olearia	5					
	Thermoanaerobacter pseudoethanolicus	6					
	Thermococcus kodakarensis	7					
	Methanothermococcus okinawensis	8					

√ = homolog detected

Table S5. Sulfate reduction genes annotated by RAST in genome scaffolds extracted from metagenome data generated via Multi-Metagenome analysis.

SITE	BEST RECRUITMENT	SCAFFOLD	*aprA*	*aprB*	*dsrA*	*dsrB*	*dsrk*	*dsrJ*	*dsrP*	*dsrM*	*dsrE*	*dsrF*	*dsrH*
LC	*Desulfonatronospira thiodismutans*	1	√	√			√	√	√	√			
	Thermococcus kodakarensis	2											
	Archaeoglobus fulgidis	3	√	√	√	√	√	√	√	√			
	Pseudomonas stutzeri	4											
	Methylophaga thiooxidans	5											
	Pseudomonas stutzeri	6									√	√	√
	Shewanella amazonensis	7									√	√	√
HC	*Desulfonatronospira thiodismutans*	1	√	√	√	√	√	√	√	√			
	Alkaliphilus metalliredigens	2	√	√									
	Thermosipho africanus	3											
	Desulfohalobium retbaense	4	√	√		√	√	√	√	√			
	Kosmotoga olearia	5											
	Thermoanaerobacter pseudoethanolicus	6											
	Thermococcus kodakarensis	7											
	Methanothermococcus okinawensis	8	√	√									

√ = homolog detected

Table S6. Number of aerobic hydrocarbon degradation genes detected by MG-RAST annotation in predicted genes of genome scaffolds extracted via Multi-Metagenome analysis.

(A) Monooxygenases / Hydroxylases

SITE	BEST RECRUITMENT	SCAFFOLD	Monooxygenase (total)	Alkane monooxygenase	Cyclopentanone monooxygenase	Cyclohexanone monooxygenase	Cyclodecanone monooxygenase	Cyclododecanone	Cytochrome P450	Phenol monooxygenase	Phehylacetone monooxygenase	Xylene monooxygenase	Alkane hydroxylase
**LC**	*Desulfonatronospira thiodismutans*	1											
	Thermococcus kodakarensis	2											
	Archaeoglobus fulgidis	3							1				
	Pseudomonas stutzeri	4	7			1							
	Methylophaga thiooxidans	5											
	Pseudomonas stutzeri	6	3										
	Shewanella amazonensis	7											
**HC**	*Desulfonatronospira thiodismutans*	1											
	Alkaliphilus metalliredigens	2											
	Thermosipho africanus	3											
	Desulfohalobium retbaense	4											
	Kosmotoga olearia	5											
	Thermoanaerobacter pseudoethanolicus	6											
	Thermococcus kodakarensis	7											
	Methanothermococcus okinawensis	8											

(B) ioxygenases (table shows a selection of detected dioxygenase genes).

SITE	BEST RECRUITMENT	SCAFFOLD	Dioxygenase (total)	Ethylbenzene dioxygenase	Nitropropane dioxygenase	Isopropylcatechol dioxygenase	hydroxyphenylpyruvate dioxygenase	Phthalate dioxygenase	Benzoate dioxygenase	LigB ring opening dioxygenase	Catechol dioxygenase	Homogentisate dioxygenase	Isopropylbenzene dioxygenase	Protocatechuate dioxygenase	Toluate dioxygenase
**LC**	*Desulfonatronospira thiodismutans*	1													
	Thermococcus kodakarensis	2	1												
	Archaeoglobus fulgidis	3	1		1										
	Pseudomonas stutzeri	4	9		2		1					1			
	Methylophaga thiooxidans	5	1												
	Pseudomonas stutzeri	6	17	1	1		1		1	2	3	1	1		1
	Shewanella amazonensis	7	13		2		1					1			
**HC**	*Desulfonatronospira thiodismutans*	1													
	Alkaliphilus metalliredigens	2	3		1										
	Thermosipho africanus	3	3		1										
	Desulfohalobium retbaense	4													
	Kosmotoga olearia	5													
	Thermoanaerobacter pseudoethanolicus	6	2		1										
	Thermococcus kodakarensis	7													
	Methanothermococcus okinawensis	8													

Table S7. List of putatively identified metabolites in the HC and LC pipeline pigging samples detected (A) in negative ion mode using reverse-phase and (B) in positive ion mode using ZIC-HILIC chromatography.

A						
Retention time (minutes)	Predicted RT (minutes)	m/z calculated	mass error ppm	Formula	Putatively identified metabolites (Level 1[1])	Samples
Negative ion mode - Reverse phase column						
0.30	N/A	116.9280	-0.22	CrH2O4	chromate	HC11,LC11
0.31	N/A	193.0345	-1.91	C6H10O7	D-Glucuronate	HC11,HC3,LC11,LC3
0.36	N/A	266.0887	-1.02	C10H13N5O4	Adenosine	HC11,HC3,LC11,LC3
0.47	N/A	154.0615	-1.47	C6H9N3O2	L-Histidine	HC11,HC3,LC11,LC3
0.56	N/A	339.0926	-0.38	C12H20O11	3-Ketosucrose	HC11,LC11
0.70	N/A	412.5869	1.41	C35H39O23	Malvidin 3,7-di-(6-malonylglucoside)	HC11,LC3
0.72	N/A	505.1408	-2.09	C18H26N4O13	Asp-Glu-Asp-Glu	LC11
0.73	N/A	641.3674	1.14	C30H59O12P	PI(21:0/0:0)	HC11,HC3
0.73	N/A	625.3940	-1.84	C34H58O10	6-O-(Glcb)-(25R)-5alpha-spirostan-3beta,6alpha,23S-triol	HC11,HC3
0.74	N/A	950.2318	-1.10	C42H47O25	Albireodelphin	LC11
0.79	N/A	330.6976	-0.62	C33H62NO10P	PS(12:0/15:1(9Z))	HC3,LC11,LC3
0.79	N/A	261.0721	-0.63	C9H14N2O7	Glu-Asp	LC11
0.80	N/A	852.6099	-2.33	C48H88NO9P	PS(O-20:0/22:4(7Z,10Z,13Z,16Z))	HC3,LC3
0.80	N/A	848.6169	-0.02	C49H88NO8P	PE(44:5)	LC3
0.81	N/A	134.0466	-0.60	C5H5N5	Adenine	HC11,HC3,LC11,LC3
0.82	N/A	150.0414	-1.49	C5H5N5O	Guanine	HC11,LC11,LC3
0.83	N/A	124.9908	-0.37	C2H6O4S	2-Hydroxyethanesulfonate	HC11,LC11
0.84	N/A	214.9987	-0.96	C12H8S2	5-(3-Buten-1-ynyl)-2,2,-bithienyl	LC11
0.86	N/A	89.0241	2.76	C3H6O3	(R)-Lactate	LC3
0.87	N/A	297.0702	1.45	C16H14N2O2S	Mefenacet	LC11
0.87	N/A	275.0877	-0.83	C10H16N2O7	GammaGlutamylglutamicacid	LC11
0.87	N/A	120.9959	-0.60	C3H6O3S	3-Mercaptolactate	HC11,LC11
0.88	N/A	117.0188	-0.21	C4H6O4	Succinate	HC11,HC3,LC11,LC3

0.88	N/A	244.1299	0.37	C10H19N3O4	Leu-Asn	HC11,LC11,LC3
0.89	N/A	487.1313	-1.80	C19H28N4O7S2	Cys-Thr-Cys-Tyr	LC11
0.89	N/A	237.0871	-2.03	C11H14N2O4	Gly-Tyr	HC3,LC3
0.89	N/A	404.1303	-0.60	C15H23N3O10	Glu-Glu-Glu	LC11
0.90	N/A	288.1561	0.29	C12H23N3O5	Leu-Ala-Ser	HC11,HC3,LC11,LC3
0.90	N/A	346.1612	-0.80	C14H25N3O7	Leu-Thr-Asp	HC11,HC3,LC11,LC3
0.90	N/A	119.0357	-0.66	C5H4N4	Purine	HC11,HC3,LC11,LC3
0.93	N/A	129.0186	-1.35	C5H6O4	Mesaconate	HC11,LC11,LC3
0.94	N/A	132.0296	-1.03	C4H7NO4	L-Aspartate	LC11,LC3
0.94	N/A	501.1463	1.39	C18H30O16	alpha-L-Rhamnopyranosyl-(1->2)-beta-D-galactopyranosyl-(1->2)-beta- D-glucuronopyranoside	HC11,LC11
0.94	N/A	473.0711	-1.96	C22H18O12	Chicoric acid	LC11
0.95	N/A	131.0455	-1.41	C4H8N2O3	L-Asparagine	HC11,LC11,LC3
0.95	N/A	473.1160	-1.01	C18H26N4O7S2	Cys-Cys-Ser-Tyr	LC11,LC3
0.95	N/A	125.0348	-2.41	C5H6N2O2	Thymine	HC11,HC3,LC11,LC3
0.96	N/A	217.1188	-0.47	C9H18N2O4	N2-(D-1-Carboxyethyl)-L-lysine	HC11,HC3,LC11,LC3
0.98	N/A	187.1079	-1.92	C8H16N2O3	N6-Acetyl-L-lysine	HC11,HC3,LC11,LC3
1.00	N/A	131.0344	-0.66	C5H8O4	2-Acetolactate	HC11,HC3,LC11,LC3
1.01	N/A	231.1344	-0.65	C10H20N2O4	Leu-Thr	HC11,HC3,LC11,LC3
1.02	N/A	893.2076	0.52	C28H49N8O17P3S	γ-butyrobetainyl-CoA	LC11
1.04	N/A	101.0238	-1.14	C4H6O3	2-Oxobutanoate	LC11
1.04	N/A	245.1135	-1.00	C10H18N2O5	Glu-Val	HC11,HC3,LC11,LC3
1.04	N/A	219.0502	-1.20	C8H12O7	dihomocitrate	LC11
1.05	N/A	376.0990	-0.59	C13H19N3O10	Glu-Asp-Asp	LC11
1.05	N/A	140.0348	0.14	C6H7NO3	2-Aminomuconate semialdehyde	LC11
1.08	N/A	110.9754	1.68	CH4O4S	Monomethyl sulfate	LC11
1.09	N/A	215.0189	-1.64	C8H8O7	5-Carboxy-2-oxohept-3-enedioate	LC11
1.11	N/A	259.1292	-0.73	C11H20N2O5	Glu-Leu	HC11,HC3,LC11,LC3
1.11	N/A	251.1031	-0.27	C12H16N2O4	Ala-Tyr	HC11,HC3,LC3
1.15	N/A	93.0343	2.40	C6H6O	Phenol	LC11
1.15	N/A	151.0399	2.42	C8H8O3	3,4-Dihydroxyphenylacetaldehyde	LC11

1.18	N/A	221.0924	-0.92	C11H14N2O3	Phe-Gly	HC11,HC3,LC11,LC3
1.19	N/A	265.1185	-1.31	C13H18N2O4	Phe-Thr	HC11,HC3,LC11,LC3
1.20	N/A	145.0499	-1.16	C6H10O4	(S)-2-Aceto-2-hydroxybutanoate	HC11,HC3,LC11,LC3
1.20	N/A	235.1081	-0.71	C12H16N2O3	Phe-Ala	HC11,HC3,LC3
1.22	N/A	240.0505	-1.27	C10H11NO6	N-(2,3-Dihydroxybenzoyl)-L-serine	HC11,LC11,LC3
1.22	N/A	227.1395	-0.51	C11H20N2O3	Leu-Pro	HC11,HC3,LC11,LC3
1.22	N/A	152.9855	-1.81	C3H6O5S	3-sulfopropanoate	HC11
1.23	N/A	215.0552	-1.69	C9H12O6	cis-(homo)3aconitate	HC11,LC11,LC3
1.23	N/A	172.9905	-2.16	C6H6O4S	Phenol sulfate	HC11,LC11,LC3
1.24	N/A	136.9907	-1.27	C3H6O4S	3-sulfopropanal	HC3,LC11,LC3
1.24	N/A	96.9597	1.00	H2O4S	Sulfate	HC11,LC3
1.25	N/A	138.9700	-0.90	C2H4O5S	Sulfoacetate	HC11
1.27	N/A	229.1551	-0.71	C11H22N2O3	Leu-Val	HC11,HC3,LC11,LC3
1.29	N/A	157.0499	-1.20	C7H10O4	2-Isopropylmaleate	HC11,HC3,LC11,LC3
1.30	N/A	123.0558	-0.80	C6H8N2O	Methylimidazole acetaldehyde	LC11
1.30	N/A	156.9956	-2.10	C6H6O3S	benzenesulfonate	HC11,LC11
1.31	N/A	112.9368	0.46	H2O3S2	H2S2O3	HC11,LC3
1.34	N/A	197.0452	0.92	C9H10O5	3-(3,4-Dihydroxyphenyl)lactate	HC11,HC3,LC3
1.35	N/A	130.0867	-0.88	C6H13NO2	L-Leucine	HC11,HC3,LC11,LC3
1.35	N/A	242.1388	-1.94	C12H21NO4	Tiglylcarnitine	HC3,LC3
1.35	N/A	183.0291	-1.70	C8H8O5	3,4-Dihydroxymandelate	HC11,LC11,LC3
1.35	N/A	177.0217	2.24	C17H18O4Cl2	IAA-94	HC11,HC3,LC11,LC3
1.36	N/A	326.1367	-0.43	C12H26NO7P	[PC ethyl,acety] 1-ethyl-2-acetyl-sn-glycero-3-phosphocholine	HC11,LC11
1.38	N/A	142.1230	-1.15	C8H17NO	valpromide	HC11,HC3,LC11,LC3
1.38	N/A	216.1234	-0.97	C10H19NO4	O-Propanoylcarnitine	HC11,HC3,LC11,LC3
1.38	N/A	111.0446	0.03	C6H8O2	sorbate	HC11,LC11,LC3
1.39	N/A	181.0137	0.05	C8H6O5	Stipitatate	HC11,HC3,LC11,LC3
1.40	N/A	143.0342	-1.85	C6H8O4	2,3-Dimethylmaleate	HC11,HC3,LC11,LC3
1.40	N/A	214.1076	-1.58	C10H17NO4	2-Amino-9,10-epoxy-8-oxodecanoic acid	HC11,HC3,LC11,LC3
1.40	N/A	271.0817	-0.42	C12H16O7	Arbutin	HC11,LC11,LC3
1.41	N/A	129.0552	-0.06	C6H10O3	(S)-3-Methyl-2-oxopentanoic acid	HC11,HC3,LC11,LC3

1.41	N/A	167.0011	-0.68	C7H5N2OCl	Zoxazolamine	HC11,LC11
1.42	N/A	293.1496	-1.83	C15H22N2O4	Leu-Tyr	HC11,HC3,LC11,LC3
1.43	N/A	117.0552	-0.17	C5H10O3	5-Hydroxypentanoate	HC11,HC3,LC11,LC3
1.43	N/A	161.0595	2.39	C13H25O7P	DHAP(10:0)	HC11,LC11,LC3
1.45	N/A	125.0003	-0.41	C2H7O4P	2-Hydroxyethylphosphonate	HC11
1.47	N/A	183.0655	-1.22	C9H12O4	3-Methoxy-4-hydroxyphenylethyleneglycol	HC11,HC3,LC11,LC3
1.48	N/A	121.0289	-0.39	C7H6O2	Benzoate	HC11,HC3,LC11,LC3
1.48	N/A	171.0655	-1.34	C8H12O4	[FA dioxo(8:0)] 4,7-dioxo-octanoic acid	HC11,HC3,LC11,LC3
1.48	N/A	285.0968	-2.18	C13H18O7	Salicin	HC11,LC11,LC3
1.55	N/A	184.0969	-2.53	C9H15NO3	Ecgonine	LC11,LC3
1.56	N/A	230.1388	-1.81	C11H21NO4	O-Butanoylcarnitine	HC11,LC11,LC3
1.57	N/A	317.0594	2.81	C28H28O17	Acacetin 7-glucuronosyl-(1->2)-glucuronide	HC11,LC11,LC3
1.58	N/A	185.0446	-2.29	C8H10O5	3-hydroxy-3-carboxy-4,5-cyclopropylhex-5-enoate	HC11
1.59	N/A	223.0238	0.09	C8H16O2Se	6-seleno-octanoate	HC11,LC11
1.59	N/A	201.0395	-2.22	C8H10O6	cis-(homo)2aconitate	HC11,HC3,LC11,LC3
1.61	N/A	229.0708	1.94	C17H28N6O5S2	Ala-Met-Cys-His	HC11,HC3,LC11,LC3
1.62	N/A	358.1976	-0.68	C16H29N3O6	Leu-Leu-Asp	HC11,HC3,LC11,LC3
1.67	N/A	159.0656	-1.21	C7H12O4	[FA (7:0/2:0)] Heptanedioic acid	HC11,HC3,LC11,LC3
1.67	N/A	144.0661	-0.17	C6H11NO3	[FA oxo,amino(6:0)] 3-oxo-5S-amino-hexanoic acid	HC11,LC11
1.68	N/A	189.0759	-2.27	C8H14O5	(R)-3-((R)-3-Hydroxybutanoyloxy)butanoate	HC11,HC3,LC11,LC3
1.69	N/A	97.0292	2.75	C5H6O2	[FA (5:2)] 2,4-pentadienoic acid	HC11,LC11
1.69	N/A	263.0920	0.11	C14H16O5	1,-Acetoxyeugenol acetate	HC11,LC11,LC3
1.71	N/A	209.0455	2.08	C10H10O5	5-Hydroxyferulate	HC11,LC11,LC3
1.74	N/A	491.1247	-2.99	C17H24N4O13	Glu-Asp-Asp-Asp	LC11
1.76	N/A	258.0980	0.90	C11H17NO6	Proacacipetalin	HC11,LC11
1.80	N/A	141.0914	-1.16	C8H14O2	[FA (8:1)] 2Z-octenoic acid	HC11,HC3,LC11,LC3
1.81	N/A	243.0866	2.40	C19H32N6O5S2	Ala-Met-Met-His	HC11,HC3,LC11,LC3
1.83	N/A	179.0342	-1.53	C9H8O4	3-(4-Hydroxyphenyl)pyruvate	HC11,LC11,LC3
1.85	N/A	249.0220	-0.75	C12H10O4S	4,4,-Sulfonyldiphenol	HC11,LC11
1.86	N/A	185.0083	-1.67	C7H6O6	Maleylpyruvate	HC11,LC11
1.86	N/A	113.0351	-0.70	C4H6N2O2	5,6-Dihydrouracil	LC11

1.87	N/A	277.0557	-1.11	C10H14O9	hexane-6-ol-1,3,4,6-tetracarboxylate	HC11,LC11
1.91	N/A	174.0399	-2.03	C6H9NO5	N-Acetyl-L-aspartate	HC11,LC11,LC3
1.95	N/A	291.0870	0.36	C15H16O6	[PR] (-)-Picrotoxinin	HC11,LC11,LC3
1.97	N/A	362.0831	-1.33	C12H17N3O10	Asp-Asp-Asp	LC11
2.00	N/A	175.0239	-2.29	C6H8O6	Ascorbate	HC11,LC11,LC3
2.02	N/A	155.1071	-0.99	C9H16O2	[FA hydroxy(9:1)] 4-hydroxy-2-nonenal	HC11,HC3,LC11,LC3
2.03	N/A	247.0566	-0.36	C8H12N2O7	Asp-Asp	LC11,LC3
2.03	N/A	295.0815	-0.89	C14H16O7	dehypoxanthine futalosine	HC11,LC11
2.10	N/A	131.0457	0.03	C4H8N2O3	L-Asparagine	LC11,LC3
2.17	N/A	275.0916	-1.50	C15H16O5	Lactucin	HC11,LC11,LC3
2.17	N/A	187.0240	-1.41	C7H8O6	(Z)-But-2-ene-1,2,3-tricarboxylate	HC11,LC11,LC3
2.28	N/A	193.0498	-1.57	C10H10O4	Ferulate	HC11,LC11,LC3
2.28	N/A	203.0917	-1.36	C9H16O5	Diethyl (2R,3R)-2-methyl-3-hydroxysuccinate	LC11,LC3
2.29	N/A	189.0575	1.71	C12H24N6O4S2	Cys-Cys-Arg	HC11,HC3,LC11,LC3
2.31	N/A	427.1755	-0.43	C24H28O7	[Fv] Heteroflavanone B	HC11,LC11,LC3
2.32	N/A	230.1028	-0.48	C10H17NO5	Suberylglycine	HC11,LC11
2.36	N/A	115.0030	-1.62	C4H4O4	Fumarate	HC11,HC3,LC11,LC3
2.37	N/A	519.2444	-2.20	C25H36N4O8	Glu-Leu-Pro-Tyr	HC11,HC3,LC3
2.38	N/A	251.0556	-0.01	C24H24O12	Dalpatein 7-O-glucoside	HC11,LC11
2.45	N/A	208.0607	-1.48	C10H11NO4	4-Hydroxyphenylacetylglycine	HC11
2.45	N/A	372.2133	-0.55	C17H31N3O6	Glu-Ile-Ile	HC11,HC3,LC11,LC3
2.45	N/A	473.0976	-2.39	C18H26N4O5S3	Cys-Phe-Cys-Cys	HC11,HC3,LC3
2.46	N/A	111.0809	-1.28	C7H12O	[FA (7:1)] 2-heptenal	HC11,HC3,LC11,LC3
2.48	N/A	411.1070	-2.59	C22H20O8	Podophyllotoxone	HC11,HC3,LC11,LC3
2.49	N/A	489.1227	0.20	C17H26N6O7S2	Glu-Cys-Cys-His	HC11,LC11
2.49	N/A	126.9044	-1.01	HI	hydrogen iodide	HC11,HC3,LC11,LC3
2.49	N/A	121.0289	-0.57	C7H6O2	Benzoate	HC11,HC3,LC11,LC3
2.50	N/A	375.1293	0.40	C16H24O10	Loganate	HC11,HC3,LC11,LC3
2.51	N/A	173.0811	-1.62	C8H14O4	Suberic acid	HC11,HC3,LC11,LC3
2.51	N/A	439.2200	1.56	C20H32N4O7	Asp-Leu-Pro-Pro	HC11,LC11
2.57	N/A	129.0551	-0.86	C6H10O3	(S)-3-Methyl-2-oxopentanoic acid	HC11,LC11,LC3

2.59	N/A	309.06	-2.65	C8H15N4O7P	5-phosphoribosyl-4,5-aminoimidazole	HC11,LC11
2.59	N/A	213.08	-2.38	C20H28O10	Furcatin	HC11,HC3,LC11,LC3
2.62	N/A	118.94	-1.80	H3O9P3	Trimetaphosphate	HC11,LC11,LC3
2.67	N/A	294.11	1.93	C13H17N3O5	Asn-Tyr	HC11
2.71	N/A	145.09	-2.28	C7H14O3	[FA hydroxy(7:0)] 2-hydroxy-heptanoic acid	HC11,HC3,LC3
2.72	N/A	158.08	-1.75	C7H13NO3	5-Acetamidopentanoate	HC11,LC11
2.74	N/A	130.09	-0.86	C6H13NO2	L-Leucine	HC11,HC3,LC11,LC3
2.75	N/A	175.08	-1.31	C11H12O2	Ethyl cinnamate	HC11,LC11
2.77	N/A	299.11	-0.63	C14H20O7	Salidroside	HC11,LC11
2.77	N/A	258.13	-0.82	C12H21NO5	N-(3-oxooctanoyl)-L-homoserine	HC11,HC3,LC11,LC3
2.79	N/A	121.07	1.33	C8H10O	Phenylethyl alcohol	HC11,LC11,LC3
2.79	N/A	416.15	-0.26	C14H23N7O8	Asn-Asn-Asn-Gly	HC11,LC11
2.79	N/A	302.16	-0.21	C14H25NO6	Pimelylcarnitine	HC11,LC11
2.79	N/A	142.12	-1.48	C8H17NO	valpromide	HC11,LC11
2.82	N/A	336.12	1.92	C15H19N3O6	Phe-Asp-Gly	HC11,LC11
2.83	N/A	216.12	-0.22	C10H19NO4	O-Propanoylcarnitine	HC11,LC11
2.83	N/A	345.17	0.63	C20H26O5	Gibberellin A24	LC11
2.88	N/A	214.11	-1.80	C10H17NO4	2-Amino-9,10-epoxy-8-oxodecanoic acid	HC11,LC11
3.00	N/A	212.09	-0.04	C10H15NO4	[FA oxo(6:0)] N-(3-oxo-hexanoyl)-homoserine lactone	HC11,LC11
3.06	N/A	129.02	-0.33	C5H6O4	Mesaconate	HC11,LC11,LC3
3.06	N/A	183.14	-1.60	C11H20O2	[FA (11:1)] 10-undecenoic acid	HC11
3.09	N/A	277.07	-1.87	C28H28O12	Epicatechin 5-O-beta-D-glucopyranoside-3-benzoate	HC11,LC3
3.11	N/A	217.11	-2.22	C10H18O5	3-Hydroxysebacicacid	HC11,HC3,LC11,LC3
3.12	N/A	141.09	-0.65	C8H14O2	[FA (8:1)] 2Z-octenoic acid	HC11,HC3,LC11,LC3
3.14	N/A	330.20	0.36	C15H29N3O5	Leu-Leu-Ser	HC11,HC3,LC11,LC3
3.14	N/A	139.11	-1.75	C9H16O	[FA (9:1)] 3-nonenal	HC11,LC11,LC3
3.15	N/A	257.04	2.59	C7H15O8P	α-(2,6-anhydro-3-deoxy-D-arabino-heptulopyranosid)onate 7-phosphonate	HC11,LC11,LC3
3.16	N/A	132.03	-0.58	C4H7NO4	L-Aspartate	HC11,HC3,LC3
3.18	N/A	143.11	-1.71	C8H16O2	[FA (8:0)] octanoic acid	HC11,LC11,LC3
3.18	N/A	131.05	-0.18	C4H8N2O3	L-Asparagine	HC11,HC3,LC11,LC3

3.25	N/A	295.08	-0.60	C14H16O7	dehypoxanthine futalosine	HC11,LC11
3.26	N/A	187.06	-1.93	C8H12O5	2-oxosuberate	HC11,HC3,LC11,LC3
3.28	N/A	490.19	-0.94	C17H29N7O10	Arg-Asp-Asp-Ser	HC11,LC11
3.28	N/A	225.08	-1.23	C11H14O5	Genipin	HC11,LC11
3.33	N/A	307.12	0.51	C16H20O6	14-Dihydroxycornestin	HC11,LC11,LC3
3.34	N/A	137.10	-0.79	C9H14O	Nona-2,6-dienal	HC11,HC3,LC11,LC3
3.34	N/A	139.04	-1.50	C7H8O3	2,3,5-Trihydroxytoluene	HC11,HC3,LC11,LC3
3.36	N/A	271.10	0.41	C16H16O4	[Fv hydroxy,methox] 2,,6,-Dihydroxy-4,-methoxydihydrochalcone	HC11,LC11
3.37	N/A	439.19	-1.46	C18H28N6O7	Ala-Val-Asp-His	HC11,LC11
3.38	N/A	459.24	-2.19	C20H36N4O8	Asp-Leu-Leu-Thr	HC11,LC11,LC3
3.38	N/A	359.13	-2.35	C16H24O9	7-Deoxyloganate	HC11,LC11,LC3
3.40	N/A	157.05	-1.05	C7H10O4	2-Isopropylmaleate	HC11,HC3,LC11,LC3
3.45	N/A	344.22	-2.01	C16H31N3O5	Leu-Leu-Thr	HC11,LC11,LC3
3.46	N/A	227.09	-1.31	C11H16O5	[FA hydroxy(11:2/11:2)] 2R,9R-dihydroxy-3S,4S,7S,8S-diepoxy-5E,10-undecadien-1-ol	HC11,HC3,LC11
3.48	N/A	546.26	-0.52	C26H37N5O8	Glu-Leu-Thr-Trp	HC11,HC3,LC11,LC3
3.48	N/A	141.06	-1.12	C7H10O3	4-Oxocyclohexanecarboxylate	HC11,LC11,LC3
3.50	N/A	195.14	-1.88	C12H20O2	(1S,2R,4S)-(-)-Bornyl acetate	HC11
3.55	N/A	230.14	-0.80	C11H21NO4	O-Butanoylcarnitine	LC11
3.65	N/A	220.06	-0.76	C11H11NO4	6-Hydroxyindolelactate	HC11,LC11
3.74	N/A	133.01	-1.55	C4H6O5	(S)-Malate	HC3,LC11,LC3
3.87	N/A	327.14	-1.72	C16H24O7	[FA dioxo,hydroxy(4:0/2:0)] 9,15-dioxo-11R-hydroxy-2,3,4,5-tetranor-prostan-1,20-dioic acid	HC11,HC3,LC11,LC3
3.87	N/A	375.14	-2.46	C20H24O7	Ailanthone	HC11,LC11
3.88	N/A	469.12	-2.82	C16H30N4O6S3	Cys-Met-Met-Ser	HC11,LC11,LC3
3.91	N/A	421.07	-2.13	C12H23O14P	alpha,alpha,-Trehalose 6-phosphate	HC11,LC11,LC3
3.92	N/A	146.05	-1.21	C5H9NO4	L-Glutamate	HC11,HC3,LC11,LC3
3.96	N/A	305.10	-1.12	C16H18O6	Cimifugin	HC11,LC11,LC3
3.96	N/A	334.18	-1.76	C17H25N3O4	Leu-Phe-Gly	HC11,HC3
3.97	N/A	293.10	-2.64	C15H18O6	Tutin	HC11,LC11,LC3

3.98	N/A	167.05	-2.26	C7H8N2O3	2,3-Diaminosalicylic acid	HC11,HC3,LC11,LC3
3.99	N/A	263.09	-0.85	C14H16O5	1,-Acetoxyeugenol acetate	HC11,HC3,LC11,LC3
4.01	N/A	150.99	-2.23	C4H8O2S2	Oxidized dithiothreitol	HC11,LC3
4.01	N/A	204.03	-2.92	C10H7NO4	Xanthurenic acid	HC11,LC11
4.02	N/A	213.02	2.74	C5H11O7P	2-Deoxy-D-ribose 5-phosphate	HC11,HC3,LC3
4.02	N/A	239.03	-1.82	C14H8O4	Alizarin	HC11
4.04	N/A	364.19	-1.25	C18H27N3O5	Leu-Ala-Tyr	HC11,HC3,LC11,LC3
4.07	N/A	135.08	-1.26	C9H12O	4-Propylphenol	HC11,HC3,LC11,LC3
4.08	N/A	125.10	-0.74	C8H14O	Sulcatone	HC11,HC3,LC11,LC3
4.13	N/A	467.21	-2.60	C27H32O7	[Fv] Exiguaflavanone E	HC11,HC3
4.19	N/A	254.04	0.01	C9H10N5O2Cl	Imidacloprid	HC11,LC11
4.22	N/A	397.16	1.57	C19H22N6O4	N-Benzoyl-D-arginine-4-nitroanilide	HC11,LC11
4.25	N/A	378.20	-0.71	C19H29N3O5	Leu-Phe-Thr	HC11,HC3,LC11,LC3
4.33	N/A	469.21	2.87	C19H30N6O8	Asp-Leu-Ser-His	HC11,HC3,LC11,LC3
4.37	N/A	159.10	-1.60	C8H16O3	Ethyl (R)-3-hydroxyhexanoate	HC11,HC3,LC11,LC3
4.38	N/A	431.23	-2.13	C22H32N4O5	Ala-Phe-Val-Pro	HC11,HC3,LC11,LC3
4.41	N/A	149.10	-1.71	C10H14O	[PR] Perillyl aldehyde	HC11,LC11
4.53	N/A	212.09	-1.00	C10H15NO4	[FA oxo(6:0)] N-(3-oxo-hexanoyl)-homoserine lactone	LC11
4.54	N/A	493.24	-1.05	C22H34N6O7	Ala-Lys-Asn-Tyr	HC11,HC3,LC11,LC3
4.57	N/A	383.05	-0.93	C18H12N2O8	4-(2-carboxylato-5,6-dihydroxy-1H-indol-4-yl)-5,6-dihydroxy-1H-indole-2-carboxylate	HC11,LC11,LC3
4.60	N/A	188.03	-1.51	C10H7NO3	Kynurenate	HC11,LC11
4.68	N/A	289.11	-0.18	C16H18O5	[PK] 5-O-Methylvisamminol	HC11,LC11,LC3
4.74	N/A	342.24	-2.18	C17H33N3O4	Leu-Leu-Val	HC11,HC3,LC11
4.74	N/A	221.08	-0.23	C12H14O4	[FA (12:4/2:0)] 2E,4E,8E,10E-Dodecatetraenedioic acid	HC11,HC3,LC11
4.81	N/A	529.24	-0.50	C20H34N8O9	Gln-Gln-Gln-Gln	HC11,LC11,LC3
4.83	N/A	191.11	-2.85	C12H16O2	[FA (12:4)] 2E,4E,8Z,10E-dodecatetraenoic acid	HC11,LC11
4.98	N/A	121.03	-0.60	C7H6O2	Benzoate	HC11,HC3,LC11,LC3
5.00	N/A	277.11	-0.90	C15H18O5	Artecanin	HC11,LC11,LC3
5.01	N/A	639.12	1.33	C27H28O18	Quercetin 3-glucoside-7-glucuronide	HC11,LC11,LC3
5.02	N/A	222.08	2.88	C18H30N4O7S	Asp-Leu-Cys-Pro	HC11

5.12	N/A	200.16	-1.41	C11H23NO2	[FA amino(11:0)] 11-amino-undecanoic acid	HC3,LC11
5.14	N/A	247.10	-2.65	C14H16O4	Prenyl caffeate	HC11,HC3,LC11,LC3
5.21	N/A	237.08	-1.99	C12H14O5	3-4-5-Trimethoxycinnamicacid	HC11,LC11,LC3
5.25	N/A	258.13	2.28	C12H21NO5	N-(3-oxooctanoyl)-L-homoserine	LC11
5.26	N/A	523.22	-2.19	C26H36O11	Mascaroside	HC11,HC3,LC11,LC3
5.27	N/A	272.19	-0.81	C14H27NO4	Heptanoylcarnitine	HC3,LC11,LC3
5.27	N/A	382.15	-2.62	C14H21N7O6	Asn-Asn-His	HC11,LC11
5.28	N/A	158.08	-2.91	C7H13NO3	5-Acetamidopentanoate	HC11,LC11
5.29	N/A	427.17	-0.39	C20H24N6O5	Trp-Ser-His	HC11,LC11
5.29	N/A	376.22	-1.52	C20H31N3O4	Leu-Phe-Val	HC11,HC3,LC11,LC3
5.30	N/A	144.04	-2.59	C9H7NO	3-Methyleneoxindole	HC11,LC11,LC3
5.34	N/A	502.19	-0.78	C18H29N7O10	Asn-Asp-Gln-Gln	HC11,LC11
5.34	N/A	130.09	-2.68	C6H13NO2	L-Leucine	HC11,LC11
5.37	N/A	425.18	-1.59	C17H26N6O7	Asp-Val-Gly-His	HC11,LC11,LC3
5.37	N/A	537.27	0.33	C24H38N6O8	Gln-Lys-Thr-Tyr	HC11,HC3,LC11
5.52	N/A	525.26	-2.02	C25H34N8O5	His-Lys-Trp-Gly	HC11,LC11,LC3
5.53	N/A	407.17	0.14	C22H24N4O4	Phe-Trp-Gly	HC11,LC11,LC3
5.55	N/A	235.10	0.31	C13H16O4	3-Dimethylallyl-4-hydroxymandelic acid	HC11,LC11,LC3
5.57	N/A	219.07	-2.64	C12H12O4	2,6-Dioxo-6-phenylhexanoate	LC11,LC3
5.59	N/A	90.99	1.96	C2H4O2S	Thioglycolate	HC11,LC3
5.61	N/A	629.22	-2.45	C34H38N4O4S2	heme c	HC11
5.61	N/A	205.01	-0.76	C10H6O5	flaviolin	HC11
5.62	N/A	457.20	-2.62	C18H30N6O8	Asn-Thr-Gln-Pro	HC11,LC11,LC3
5.62	N/A	255.03	-2.54	C14H8O5	Purpurin	HC11,LC11,LC3
5.63	N/A	291.12	-0.79	C32H40O10	[PR] Pubescenol	HC3,LC11,LC3
5.63	N/A	469.21	-0.28	C24H30N4O6	Ala-Phe-Ala-Tyr	HC11,HC3,LC11,LC3
5.65	N/A	285.08	-2.03	C16H14O5	[Fv] Licodione 2,-methyl ether	HC11,LC11,LC3
5.65	N/A	455.19	-0.29	C18H28N6O8	Asp-Val-Ser-His	HC11,LC11,LC3
5.66	N/A	447.20	-2.69	C24H32O8	[ST (2:0)] estra-1,3,5(10)-triene-3,17alpha-diol 3-D-glucuronide	HC11,LC11,LC3
5.66	N/A	337.09	-1.33	C16H18O8	p-Coumaroyl quinic acid	HC11,LC11

5.68	N/A	419.19	-0.24	C20H28N4O6	Ala-Ala-Pro-Tyr	HC11,LC11
5.68	N/A	243.03	-0.53	C13H8O5	Gentisein	HC11
5.69	N/A	150.99	-1.92	C4H8O2S2	Oxidized dithiothreitol	HC11
5.69	N/A	249.02	-0.73	C12H10O4S	4,4,-Sulfonyldiphenol	HC11,HC3,LC11,LC3
5.69	N/A	225.07	1.26	C25H24O8	Urdamycinone B	HC11,HC3,LC3
5.70	N/A	319.12	-0.57	C17H20O6	PR-toxin	HC11,LC11,LC3
5.72	N/A	471.22	0.20	C19H32N6O8	Asn-Lys-Asp-Pro	HC11,LC11,LC3
5.72	N/A	175.08	-0.92	C11H12O2	Ethyl cinnamate	HC11,HC3,LC11,LC3
5.72	N/A	337.17	2.78	C18H26O6	Pinolidoxin	HC11,LC11,LC3
5.73	N/A	393.15	-2.60	C20H26O8	Glaucarubolone	HC11,LC11,LC3
5.74	N/A	439.20	-2.91	C22H32O9	10-Deacetyl-2-debenzoylbaccatin III	HC11,HC3,LC11,LC3
5.79	N/A	281.05	1.57	C16H10O5	[Fv Methyl,hydrox] 4,5-Methylenedioxy-6-hydroxyaurone	HC11
5.80	N/A	119.02	-0.04	C4H8O2S	3-(Methylthio)propionic acid	HC11,LC3
5.82	N/A	265.15	-2.07	C12H26O4S	sodium dodecyl sulfate	HC11,HC3,LC11,LC3
5.84	N/A	309.08	-0.38	C18H14O5	6-Deoxyjacareubin	HC11,LC11
5.89	N/A	516.26	0.66	C24H35N7O6	Ala-Lys-Trp-Asn	HC11,LC11
5.93	N/A	374.21	-0.13	C20H29N3O4	Leu-Phe-Pro	HC11,HC3,LC11,LC3
5.94	N/A	237.15	-1.99	C14H22O3	[FA oxo(5:1/5:0/4:0)] (1R,2R)-3-oxo-2-(2,Z-pentenyl)-cyclopentanebutanoic acid	HC11
5.98	N/A	201.11	-2.11	C10H18O4	[FA (10:0/2:0)] Decanedioic acid	HC11,HC3,LC11
6.05	N/A	475.20	-2.48	C25H32O9	[ST methoxy,hydroxy(3:0)] 2-methoxy,3-hydroxy-estra-1,3,5(10)-trien-17-one 3-D-glucuronide	HC11,LC11,LC3
6.07	N/A	349.08	-1.98	C11H18N4O7S	Asn-Asp-Cys	HC11,HC3,LC11,LC3
6.14	N/A	525.23	-0.90	C23H30N10O5	His-Pro-His-His	HC11,HC3,LC11
6.16	N/A	422.17	-1.78	C17H29NO11	Neolinustatin	HC11,LC11,LC3
6.17	N/A	257.14	-0.92	C26H44O10	L-Olivosyl-oleandolide	HC11,HC3,LC11,LC3
6.19	N/A	327.14	0.99	C16H24O7	[FA dioxo,hydroxy(4:0/2:0)] 9,15-dioxo-11R-hydroxy-2,3,4,5-tetranor-prostan-1,20-dioic acid	HC11,HC3,LC11,LC3
6.22	N/A	473.16	-0.93	C18H30N6O5S2	Cys-Leu-Cys-His	HC11,HC3,LC11,LC3
6.22	N/A	498.21	0.21	C23H29N7O6	N-Acetyl-O-demethylpuromycin	HC11,LC11
6.22	N/A	157.12	0.47	C9H18O2	Nonanoic acid	HC3,LC3

6.23	N/A	540.24	-2.20	C27H35N5O7	Asn-Phe-Val-Tyr	HC11,LC3
6.25	N/A	359.17	-0.83	C17H28O8	1-O-(8R-hydroxy-8-methyl-3Z,9-decadienoyl)-beta-D-glucopyranose	HC11,HC3,LC11,LC3
6.25	N/A	141.05	-1.48	C7H10O3	4-Oxocyclohexanecarboxylate	HC11,LC11,LC3
6.29	N/A	337.13	2.73	C17H22O7	1-Peroxyferolide	HC11,LC11
6.33	N/A	540.26	-2.97	C26H35N7O6	Arg-Phe-Gly-Tyr	HC11,LC11,LC3
6.34	N/A	223.08	-1.91	C15H12O2	Flavanone	HC11
6.37	N/A	537.26	0.42	C26H34N8O5	His-Phe-Val-His	HC11,LC11
6.38	N/A	500.27	-2.26	C18H35N11O6	Arg-Asn-Gly-Arg	HC11,LC11
6.38	N/A	255.07	-0.60	C15H12O4	Isoliquiritigenin	HC11,LC11,LC3
6.39	N/A	443.24	2.66	C17H32N8O6	Ala-Ala-Gln-Arg	LC11
6.44	N/A	249.11	1.05	C14H18O4	ubiquinone-1	HC11,LC11,LC3
6.48	N/A	292.10	-0.16	C10H19N3O5S	Met-Gly-Ser	HC11,LC11,LC3
6.49	N/A	501.28	0.82	C20H38N8O7	Arg-Leu-Gln-Ser	HC11,LC11
6.49	N/A	431.19	-1.59	C21H28N4O6	Leu-Trp-Asp	HC11,LC11,LC3
6.50	N/A	560.31	-0.12	C24H39N11O5	Arg-Leu-His-His	HC11,LC3
6.50	N/A	473.25	-0.43	C23H34N6O5	Ala-Lys-Trp-Ala	HC11,LC11
6.53	N/A	439.29	-2.08	C22H40N4O5	Ile-Leu-Val-Pro	HC11,HC3,LC11,LC3
6.55	N/A	499.26	-0.97	C20H36N8O7	Arg-Thr-Gln-Pro	HC11,LC11,LC3
6.59	N/A	149.06	-2.41	C9H10O2	Phenylpropanoate	HC11,LC11,LC3
6.64	N/A	173.12	-2.55	C9H18O3	[FA hydroxy(9:0)] 2-hydroxy-nonanoic acid	HC11,HC3,LC11,LC3
6.68	N/A	417.23	2.53	C20H30N6O4	Phe-Pro-Arg	HC11,LC11,LC3
6.71	N/A	273.13	2.22	C23H36N10O6	Gln-Lys-His-His	HC11,LC11,LC3
6.71	N/A	338.14	2.66	C15H21N3O6	Ala-Ser-Tyr	HC11,LC11,LC3
6.72	N/A	253.05	-0.40	C7H14N2O6S	5-L-Glutamyl-taurine	HC11
6.73	N/A	130.09	-0.97	C6H13NO2	L-Leucine	HC11,LC11,LC3
6.77	N/A	518.23	0.71	C24H33N5O8	Asp-Leu-Trp-Ser	HC11,HC3,LC11,LC3
6.81	N/A	217.12	-2.63	C14H18O2	[FA (14:5)] 5,7,9,11,13-tetradecapentaenoic acid	HC11,LC11,LC3
6.84	N/A	373.17	-0.35	C14H26N6O4S	Cys-Pro-Arg	HC11,LC11
6.86	N/A	150.99	-1.60	C4H8O2S2	Oxidized dithiothreitol	HC11,LC3

7.03	N/A	323.09	0.96	C19H16O5	[Fv Dimethoxy(9:1)] 5,6-Dimethoxy-[2,,,3,,:7,8]furanoflavanone	HC11,LC11,LC3
7.05	N/A	271.13	-2.75	C17H20O3	2,2-Dimethyl-8-prenylchromene 6-carboxylic acid	HC11,LC11
7.05	N/A	227.13	-1.80	C12H20O4	Traumatic acid	HC11,HC3,LC11,LC3
7.05	N/A	405.19	-1.09	C22H30O7	Isomontanolide	HC11,LC11,LC3
7.08	N/A	481.30	2.99	C27H46O5S	26-hydroxycholesterol 3-sulfate	HC11
7.11	N/A	508.20	2.60	C18H35N7O6S2	Arg-Met-Thr-Cys	HC11,LC11
7.13	N/A	408.18	2.36	C19H27N3O7	Leu-Asp-Tyr	HC11,LC11
7.16	N/A	239.08	1.28	C9H13N6Cl	Cyanazine	HC11,HC3,LC11,LC3
7.19	N/A	266.11	-0.85	C21H30N10O7	Asn-Gln-His-His	HC11,HC3,LC11,LC3
7.20	N/A	611.29	-1.72	C29H40N8O7	Arg-Phe-Gln-Tyr	HC11,LC11,LC3
7.21	N/A	352.18	0.25	C18H27NO6	Rosmarinine	HC11
7.21	N/A	356.12	-0.91	C13H19N5O7	Asp-Ser-His	HC11
7.27	N/A	355.12	0.18	C20H20O6	Kievitone	HC11,LC11,LC3
7.32	N/A	283.11	-0.27	C23H36N8O7S	Arg-Cys-Gln-Tyr	HC11,HC3,LC11,LC3
7.36	N/A	195.08	-2.57	C14H12O	cis-Stilbene oxide	HC11
7.38	N/A	713.42	-2.15	C41H63O8P	PA(18:4(6Z,9Z,12Z,15Z)/20:5(5Z,8Z,11Z,14Z,17Z))	HC11
7.48	N/A	573.27	1.46	C27H38N6O8	Glu-Leu-Trp-Gln	HC11,LC11,LC3
7.49	N/A	305.14	0.15	C17H22O5	Matricin	HC11,LC11,LC3
7.54	N/A	217.16	-1.83	C15H22O	Solavetivone	HC11
7.54	N/A	558.31	-0.81	C21H41N11O7	Arg-Thr-Gln-Arg	HC11
7.55	N/A	267.16	-1.77	C15H24O4	dihydroartemisinic acid hydroperoxide	HC11,LC11,LC3
7.62	N/A	215.13	-1.37	C11H20O4	[FA (11:0/2:0)] Undecanedioic acid	HC11,HC3,LC11
7.64	N/A	594.29	0.84	C27H37N11O5	Arg-Phe-His-His	HC11,HC3,LC11,LC3
7.66	N/A	587.32	2.08	C40H44O4	synechoxanthin	HC11,LC11
7.67	N/A	296.19	-0.31	C16H27NO4	N-(3-Oxododecanoyl)homoserine lactone	HC11,HC3,LC3
7.68	N/A	377.16	-0.86	C20H26O7	Chaparrinone	HC11,LC11,LC3
7.70	N/A	261.11	-2.05	C15H18O4	[PR] Parthenin	HC11,HC3,LC11,LC3
7.71	N/A	188.07	-2.52	C11H11NO2	3-Indolepropionicacid	HC11,HC3,LC3
7.79	N/A	355.09	2.04	C16H15F3N2O4	Bay-K-8644	HC11,LC11,LC3
7.89	N/A	369.06	-2.29	C16H20O6P2	BPH-674	HC11,HC3,LC11,LC3

7.90	N/A	505.28	-1.79	C24H38N6O6	Asn-Lys-Phe-Val	HC11,LC11,LC3
7.91	N/A	423.03	-2.76	C12H18N4O7P2S	Thiamin diphosphate	HC11,HC3,LC11,LC3
7.91	N/A	263.13	-0.06	C15H20O4	[PR trihydroxy(2:0)] (-)-1alpha,7beta,12-trihydroxy-2,9-illudadien-8-one	HC11,LC11,LC3
7.91	N/A	379.19	-2.07	C17H33O7P	PA(14:1(9Z)/0:0)	HC11,LC11,LC3
7.92	N/A	317.14	-0.81	C18H22O5	Zearalenone	HC11,LC11,LC3
7.96	N/A	421.16	-2.09	C25H26O6	[Fv] Flemingin C	HC11,LC3
7.97	N/A	210.05	-0.38	C8H10N5Cl	m-Chlorophenylbiguanide	HC11,LC3
8.01	N/A	637.21	-2.73	C30H38O15	[Fv Hydroxy,trimethoxy(9:1)] 4,-Hydroxy-5,7,2,-trimethoxyflavanone 4,-rhamnosyl-(1->6)-glucoside	HC11
8.01	N/A	629.35	-1.49	C39H50O7	[PR] Peridinin	HC11
8.02	N/A	347.13	2.30	C22H20O4	Erybraedin E	HC11,HC3,LC11,LC3
8.02	N/A	579.25	-1.63	C24H36N8O9	Arg-Asp-Gln-Tyr	HC11,LC3
8.04	N/A	437.25	2.57	C20H34N6O5	Ala-Leu-Val-His	HC11,LC11,LC3
8.05	N/A	240.07	-1.72	C14H11NO3	N-Benzoylanthranilate	HC11
8.10	N/A	283.14	0.02	C29H44O11	Sarmentoloside	HC11,HC3,LC11,LC3
8.12	N/A	309.17	-2.94	C17H26O5	Botrydial	HC11,LC11,LC3
8.15	N/A	349.17	-0.29	C19H26O6	Alatolide	HC11,LC11,LC3
8.17	N/A	539.34	-2.91	C23H44N10O5	Arg-Leu-Pro-Arg	HC11,LC11,LC3
8.20	N/A	368.11	1.81	C20H19NO6	6-Hydroxyprotopine	HC11,LC11
8.21	N/A	307.13	1.77	C20H20O3	[Fv] Isocordoin	HC11,HC3,LC11,LC3
8.27	N/A	522.22	2.67	C19H37N7O6S2	Arg-Met-Met-Ser	HC11,LC11,LC3
8.28	N/A	228.16	-0.73	C12H23NO3	N-Decanoylglycine	HC11,LC11,LC3
8.29	N/A	333.13	-2.80	C36H44O12	Agrimol C	HC11,LC11,LC3
8.30	N/A	502.24	2.16	C23H33N7O6	Asn-Lys-Trp-Gly	HC11,LC3
8.30	N/A	343.13	-0.03	C18H20N2O5	Tyr-Tyr	HC11,LC11,LC3
8.31	N/A	321.11	-2.50	C12H22N2O6S	N-((R)-Pantothenoyl)-L-cysteine	HC11,LC11,LC3
8.35	N/A	369.17	-0.74	C19H30O5S	[ST hydrox] 3alpha-hydroxy-5alpha-androstan-17-one 3-sulfate	HC11,LC11,LC3
8.36	N/A	253.05	0.71	C7H14N2O6S	5-L-Glutamyl-taurine	HC11
8.37	N/A	347.24	-1.89	C18H36O6	[FA hydroxy(4:0/18:0)] 9,10,12,13-tetrahydroxy-octadecanoic acid	HC11

8.49	N/A	291.12	0.32	C32H40O10	[PR] Pubescenol	HC11,LC11,LC3
8.50	N/A	275.13	-1.44	C16H20O4	Scorpioidin	HC11,HC3,LC11,LC3
8.52	N/A	329.16	-2.61	C16H26O7	8-Epiiridodial glucoside	HC11,LC11,LC3
8.56	N/A	429.20	-0.59	C23H30N2O6	Cinegalline	HC11,HC3,LC11,LC3
8.61	N/A	259.10	0.05	C15H16O4	[PR] Hemigossypol	HC11,LC11,LC3
8.61	N/A	189.09	-2.91	C12H14O2	[FA (12:5)] 3,5,7,9,11-dodecapentaenoic acid	HC11,LC11
8.61	N/A	267.14	-2.78	C12H20N4O3	His-Leu	HC11,HC3,LC11,LC3
8.61	N/A	265.15	-1.23	C12H26O4S	sodium dodecyl sulfate	HC11,HC3,LC11,LC3
8.63	N/A	411.24	-1.32	C22H36O7	Grayanotoxin I	HC11,HC3,LC11
8.65	N/A	223.04	-2.19	C14H8O3	Hydroxyanthraquinone	HC11
8.65	N/A	376.19	-1.67	C19H27N3O5	Val-Pro-Tyr	HC11,LC11,LC3
8.70	N/A	373.17	0.26	C21H26O6	Phantomolin	HC11,LC11,LC3
8.76	N/A	269.17	-1.74	C15H26O4	(10S)-Juvenile hormone III acid diol	HC11,LC11,LC3
8.79	N/A	519.29	-2.30	C25H40N6O6	Asn-Leu-Lys-Phe	HC11,HC3,LC11,LC3
8.81	N/A	265.13	-0.66	C12H18N4O3	L-lysine-p-nitroanilide	HC11
8.84	N/A	395.28	1.52	C23H40O5	[FA trihydroxy(3:2/2:0)] isopropyl 9S,11R,15S-trihydroxy-5Z,13E-prostadienoate	HC11
8.87	N/A	469.17	-1.55	C17H30N2O13	D-Galactosyl-3-(N-acetyl-beta-D-galactosaminyl)-L-serine	HC11,LC11,LC3
8.93	N/A	382.13	2.44	C21H21NO6	(+)-Adlumine	HC11,HC3,LC3
9.05	N/A	323.18	-2.79	C18H28O5	[FA oxo,hydroxy(18:3)] 12-oxo-14,18-dihydroxy-9Z,13E,15Z-octadecatrienoic acid	HC11,LC3
9.06	N/A	263.08	-1.08	C16H12N2O2	Perlolyrine	HC11
9.07	N/A	395.24	-1.25	C22H36O6	[FA methyl(5:1/5:2/8:0)] methyl 8-[3,5-epidioxy-2-(3-hydroperoxy-1-pentenyl)-cyclopentyl]-octanoate	HC11,HC3,LC11
9.08	N/A	300.22	-0.94	C16H31NO4	2-6dimethylheptanoylcarnitine	LC11,LC3
9.10	N/A	365.19	-0.79	C21H28F2O3	6alpha,9-Difluoro-11beta-hydroxypregn-4-ene-3,20-dione	HC11,LC11,LC3
9.13	N/A	563.32	2.97	C23H40N12O5	Arg-Pro-Arg-His	HC11,HC3,LC11,LC3
9.16	N/A	229.14	-1.08	C12H22O4	Dodecanedioic acid	HC11,HC3,LC11
9.17	N/A	335.15	1.36	C18H24O6	omega-Carboxy-trinor-leukotriene B4	HC11,HC3,LC11,LC3
9.17	N/A	308.16	2.24	C15H23N3O4	Lys-Tyr	HC11,LC11
9.21	N/A	403.20	-1.34	C20H28N4O5	Leu-Trp-Ser	HC11,LC11,LC3

9.21	N/A	233.07	0.93	C29H24O6	[Fv] Dichamanetin	HC11
9.27	N/A	409.16	-0.16	C18H35O2I	[FA (18:0)] 3-iodo-octadecanoic acid	HC11,HC3,LC3
9.28	N/A	200.16	-1.83	C11H23NO2	[FA amino(11:0)] 11-amino-undecanoic acid	HC3,LC11,LC3
9.30	N/A	389.16	-2.10	C21H26O7	Erioflorin acetate	HC11,LC11,LC3
9.31	N/A	407.21	-1.12	C22H32O7	Cascarillin	HC11,HC3,LC11,LC3
9.39	N/A	454.28	2.43	C20H37N7O5	Ala-Leu-Pro-Arg	HC11,LC3
9.39	N/A	655.33	-0.16	C30H44N10O7	Arg-Tyr-Tyr-Arg	HC11,LC11,LC3
9.42	N/A	350.15	0.04	C20H21N3O3	Phe-Trp	HC11,LC11,LC3
9.44	N/A	277.14	0.65	C16H22O4	2-Ethylhexyl phthalate	HC11,HC3,LC11,LC3
9.46	N/A	261.07	-0.02	C16H10N2O2	indigo	HC11,HC3,LC11,LC3
9.49	N/A	441.19	2.88	C18H30N6O5S	Ala-Leu-Cys-His	HC11,HC3,LC11,LC3
9.52	N/A	650.34	2.39	C25H43N14O7	[PK] Capreomycin	HC11,HC3,LC3
9.53	N/A	389.20	-1.12	C22H30O6	Neoquassin	HC11,HC3,LC11,LC3
9.59	N/A	311.17	1.08	C33H52O11	Spongipregnoloside A	HC11,HC3,LC11,LC3
9.61	N/A	396.15	1.32	C22H23NO6	Aureothin	HC11,HC3,LC11,LC3
9.61	N/A	359.19	-0.95	C21H28O5	Cortisone	HC11,LC11,LC3
9.61	N/A	328.21	1.17	C17H31NO5	6-Keto-decanoylcarnitine	HC11,LC11,LC3
9.66	N/A	533.31	-2.19	C26H42N6O6	Gln-Leu-Lys-Phe	HC11,LC11,LC3
9.67	N/A	228.20	-1.43	C13H27NO2	2S-Amino-tridecanoic acid	HC11,LC11,LC3
9.70	N/A	350.03	-1.31	C11H17N3O5Se	5-methylaminomethyl-2-selenouridine	HC3,LC11
9.71	N/A	279.07	-1.52	C17H12O4	Neodunol	HC11,LC11
9.74	N/A	296.19	-1.86	C16H27NO4	N-(3-Oxododecanoyl)homoserine lactone	HC11,HC3,LC3
9.77	N/A	363.18	0.40	C20H28O6	Gibberellin A44 diacid	HC11,LC11,LC3
9.77	N/A	242.18	-1.50	C13H25NO3	N-Undecanoylglycine	HC11,HC3,LC11
9.81	N/A	439.15	0.77	C18H24N4O9	Asp-Gly-Ser-Tyr	HC11,LC11,LC3
9.85	N/A	361.17	-0.49	C13H26N6O4S	Met-Gly-Arg	HC11,HC3,LC11,LC3
9.87	N/A	234.06	1.56	C7H13N3O4S	Asn-Cys	HC11
9.91	N/A	490.23	-1.72	C18H33N7O9	Arg-Thr-Thr-Asp	HC3,LC11,LC3
9.91	N/A	439.23	0.09	C19H32N6O6	Ala-Leu-Thr-His	HC11,HC3,LC11,LC3
9.95	N/A	256.19	0.27	C14H27NO3	N-Lauroylglycine	LC11,LC3

9.96	N/A	185.06	1.09	C18H29BrO3	(12Z,15S,18S)-15-hydroxy-18-bromo-12,16,17-octadecatrienoic acid	HC11,HC3,LC11,LC3
10.04	N/A	311.13	0.40	C19H20O4	[Fv] Desmosdumotin C	HC11,LC11
10.04	N/A	459.27	-1.67	C23H36N6O4	Lys-Lys-Trp	HC11,HC3,LC11
10.06	N/A	477.21	-1.92	C21H30N6O7	Ala-Phe-Asn-Gln	HC11,HC3,LC11
10.10	N/A	715.51	-2.19	C43H73O6P	2,3-Bis-O-(geranylgeranyl)glycerol 1-phosphate	LC11
10.15	N/A	351.14	2.39	C18H25O5P	Estra-1,3,5(10)-triene-3,17beta-diol 3-phosphate	HC11,HC3,LC11,LC3
10.16	N/A	486.25	2.36	C23H33N7O5	His-Lys-Phe-Gly	HC11,HC3,LC11,LC3
10.18	N/A	798.53	-1.14	C43H78NO10P	PS(15:1(9Z)/22:2(13Z,16Z))	LC11
10.20	N/A	367.21	0.48	C20H32O6	Prostaglandin G2	HC3,LC11
10.21	N/A	443.10	-2.55	C22H20O10	Granaticin	HC11,HC3,LC11,LC3
10.22	N/A	457.18	-0.75	C19H30N4O7S	Asp-Met-Pro-Pro	HC11,HC3,LC11,LC3
10.23	N/A	411.24	-1.27	C22H36O7	Grayanotoxin I	HC11,HC3,LC11
10.24	N/A	590.32	-0.47	C25H41N11O6	Arg-Phe-Asn-Arg	HC11,HC3,LC11,LC3
10.28	N/A	396.25	-2.19	C44H75O10P	PG(38:6)	HC11,HC3,LC11
10.28	N/A	423.16	-2.54	C16H28N2O11	Chitobiose	HC11,HC3,LC11,LC3
10.29	N/A	673.36	-2.31	C34H46N10O5	Arg-Lys-Trp-Trp	HC11,HC3,LC3
10.30	N/A	465.25	-2.21	C25H38O8	Androsterone glucuronide	HC11,HC3,LC11
10.30	N/A	449.22	2.92	C20H30N6O6	His-Thr-Pro-Pro	HC11,HC3,LC11
10.32	N/A	246.06	-1.59	C16H9NO2	3-Nitrofluoranthene	HC11
10.34	N/A	558.27	0.07	C20H37N11O8	Arg-Asn-Asp-Arg	HC11,HC3,LC11,LC3
10.34	N/A	579.03	-1.38	C15H22N2O18P2	UDP-glucuronate	HC11
10.34	N/A	437.16	-1.43	C25H26O7	[Fv hydroxy,hydroxy,methyl,dimethyl(4:2/9:1)] 5,4-Dihydroxy-8-(1-hydroxy-2,3-epoxy-3-methylbutyl)-6,,,6,,-dimethylpyrano[2,,,3,,:7,6]flavanone	HC11,HC3,LC11,LC3
10.38	N/A	385.18	0.97	C15H26N6O6	Asp-Pro-Arg	HC11,LC11,LC3
10.38	N/A	329.23	-0.78	C18H34O5	[FA trihydroxy(18:1)] 9S,12S,13S-trihydroxy-10E-octadecenoic acid	HC11,HC3,LC11,LC3
10.40	N/A	221.15	-1.83	C14H22O2	Rishitin	HC11,LC11,LC3
10.40	N/A	557.27	0.90	C24H47O12P	PI(15:0/0:0)	HC11,HC3,LC11
10.45	N/A	422.17	1.01	C17H29NO11	Neolinustatin	HC11,HC3,LC11,LC3

10.47	N/A	588.34	1.58	C23H47N11O5S	Arg-Lys-Met-Arg	HC11,LC3
10.47	N/A	500.32	-2.06	C22H43N7O6	Arg-Leu-Leu-Thr	HC11
10.50	N/A	496.20	0.96	C20H35NO13	Validamycin A	HC11,HC3,LC3
10.56	N/A	507.26	2.47	C23H36N6O7	Ala-Lys-Gln-Tyr	HC11,HC3,LC11,LC3
10.58	N/A	761.32	2.48	C44H42N8O5	Trp-Trp-Trp-Trp	HC11,LC3
10.59	N/A	506.27	-1.22	C27H41NO8	Deltaline	HC11,HC3,LC3
10.60	N/A	391.21	0.44	C22H32O6	Isodomedin	HC11,HC3,LC11
10.61	N/A	415.27	-1.91	C18H36N6O5	Ala-Lys-Lys-Ala	HC11,HC3,LC3
10.64	N/A	243.16	-0.83	C13H24O4	[FA (13:0/2:0)] Tridecanedioic acid	HC11,HC3,LC11
10.64	N/A	394.20	0.45	C19H29N3O6	Leu-Thr-Tyr	LC11
10.67	N/A	496.27	-0.74	C22H44NO9P	[PS (16:0)] 1-hexadecanoyl-sn-glycero-3-phosphoserine	HC3
10.73	N/A	606.34	-1.81	C32H45N7O5	Lys-Lys-Phe-Trp	HC11,HC3,LC11
10.75	N/A	488.26	2.03	C23H35N7O5	Ala-Phe-Pro-Arg	HC11,HC3,LC11,LC3
10.80	N/A	650.34	1.41	C25H43N14O7	[PK] Capreomycin	HC3,LC11,LC3
10.80	N/A	399.27	-2.54	C18H36N6O4	Leu-Leu-Arg	HC11,HC3,LC11
10.80	N/A	463.23	-2.54	C25H36O8	[ST hydrox] 17beta-hydroxyandrost-4-en-3-one 3-D-glucuronide	HC11,HC3,LC11,LC3
10.81	N/A	550.30	2.34	C25H41N7O7	Arg-Leu-Thr-Tyr	HC11,HC3,LC11,LC3
10.83	N/A	259.02	0.63	C6H13O9P	D-Glucose 6-phosphate	HC11,HC3,LC11
10.86	N/A	425.20	-0.41	C19H30N4O7	Asp-Val-Pro-Pro	HC11,HC3,LC11
10.87	N/A	405.25	-2.65	C21H34N4O4	Leu-Lys-Phe	HC11,LC11
10.88	N/A	381.23	1.76	C17H30N6O4	Lys-Val-His	HC11,HC3,LC11,LC3
10.88	N/A	467.18	-0.61	C20H28N4O9	Ala-Thr-Asp-Tyr	HC11,HC3,LC11,LC3
10.90	N/A	267.03	-1.96	C15H8O5	Coumestrol	HC11
10.91	N/A	373.22	1.54	C15H30N6O5	Leu-Ser-Arg	HC11,LC11,LC3
10.92	N/A	549.27	-0.46	C29H42O10	Adonitoxin	HC11,HC3,LC11,LC3
10.92	N/A	604.33	-1.35	C26H43N11O6	Arg-Phe-Gln-Arg	HC11,HC3,LC11,LC3
10.94	N/A	530.27	0.92	C25H37N7O6	Ala-Lys-Trp-Gln	HC11,HC3,LC11,LC3
10.95	N/A	437.25	-1.26	C24H38O7	[PR trihydroxy(2:0)] (-)-8alpha,15-diacetoxy-4beta,9alpha,14beta-trihydroxy-3(16)-fusicoccene	HC11,LC11
10.96	N/A	277.14	1.37	C16H22O4	2-Ethylhexyl phthalate	HC11,HC3,LC11

11.02	N/A	439.12	1.05	C20H24O11	Ginkgolide C	HC11,LC3
11.03	N/A	441.14	-2.36	C18H26N4O7S	Ala-Cys-Ser-Tyr	HC11,HC3,LC11,LC3
11.06	N/A	270.21	-0.70	C15H29NO3	Tridecanoylglycine	HC11,HC3,LC11
11.07	N/A	495.09	-1.61	C16H26N4O8P2S	2-Methyl-1-hydroxypropyl-ThPP	HC11,HC3,LC11,LC3
11.09	N/A	460.15	0.11	C17H27N5O8S	Asp-Cys-Gln-Pro	HC11,HC3,LC11,LC3
11.11	N/A	465.18	1.67	C24H26N4O6	Phe-Trp-Asp	HC11,HC3,LC11
11.12	N/A	425.26	2.48	C23H38O7	[FA hydroxy,oxo(2:0)] 9S,15S-dihydroxy-11-oxo-5Z,13E-prostadienoic acid 2-glyceryl ester	HC11,HC3,LC11,LC3
11.15	N/A	510.22	0.79	C22H33N5O9	Asp-Lys-Ser-Tyr	HC11,LC11
11.16	N/A	511.15	-1.53	C21H28N4O9S	Glu-Phe-Asp-Cys	HC11,HC3,LC11,LC3
11.19	N/A	154.06	0.92	C6H9N3O2	L-Histidine	HC11,HC3,LC11,LC3
11.21	N/A	313.24	-1.67	C18H34O4	[FA hydroxy(18:1)] 9,10-dihydroxy-12Z-octadecenoic acid	HC11,HC3,LC11
11.25	N/A	423.27	-1.90	C24H40O6	[ST hydroxy(4:0)] 1beta,3alpha,7alpha,12alpha-Tetrahydroxy-5beta-cholan-24-oic Acid	HC11,HC3,LC11
11.28	N/A	659.33	-0.92	C32H52O14	CapsianosideI	HC11,HC3,LC11,LC3
11.29	N/A	576.33	-1.26	C28H52NO9P	PS(22:2(13Z,16Z)/0:0)	HC11,HC3,LC3
11.29	N/A	517.31	-0.08	C26H42N6O5	Lys-Lys-Phe-Pro	HC11,HC3,LC11
11.32	N/A	375.18	-1.19	C21H28O6	Rhipocephalin	HC3,LC11,LC3
11.33	N/A	860.54	-1.43	C48H80NO10P	PS(42:7)	HC3,LC11
11.36	N/A	456.22	1.73	C18H31N7O7	Ala-Asp-Pro-Arg	HC11,HC3,LC11,LC3
11.38	N/A	363.22	1.54	C21H32O5	Urocortisone	HC11,HC3,LC11,LC3
11.41	N/A	471.26	-0.13	C22H40N4O5S	Ile-Leu-Met-Pro	HC11,HC3,LC11,LC3
11.49	N/A	171.01	-1.87	C3H9O6P	sn-Glycerol 3-phosphate	HC11,HC3,LC11
11.50	N/A	521.27	-2.31	C28H42O9	14-O-(beta-D-glucopyranosyl)-7S,14R-dihydroxy-7,9,13,17-tetramethyl-2E,4E,8E,10E,12E,16E-octadecahexaenoic acid	HC11,HC3,LC11,LC3
11.53	N/A	285.21	-1.98	C16H30O4	[FA hydroxy,oxo(16:0)] 9-hydroxy-16-oxo-hexadecanoic acid	HC11,HC3,LC11,LC3
11.54	N/A	531.22	1.10	C24H32N6O8	Asn-Trp-Val-Asp	LC11
11.55	N/A	493.24	-1.35	C22H34N6O7	Ala-Lys-Asn-Tyr	HC11,HC3,LC11
11.56	N/A	395.24	-0.23	C22H36O6	[FA methyl(5:1/5:2/8:0)] methyl 8-[3,5-epidioxy-2-(3-hydroperoxy-1-pentenyl)-cyclopentyl]-octanoate	HC11,LC11
11.56	N/A	248.10	0.39	C20H30N6O9	Asp-Leu-Asp-His	HC11,HC3,LC3
11.56	N/A	331.25	-2.76	C18H36O5	[FA trihydroxy(18:0)] 9,10,18-trihydroxy-octadecanoic acid	HC11,HC3,LC11

11.58	N/A	305.12	-0.21	C20H18O3	[Fv] Lonchocarpin	HC11,HC3,LC11,LC3
11.59	N/A	356.24	-0.42	C19H35NO5	2,3-dioctanoylglyceramide	HC11,HC3,LC11
11.59	N/A	532.29	-1.68	C22H43N7O6S	Gln-Lys-Lys-Met	HC11,HC3,LC11,LC3
11.62	N/A	445.22	1.08	C21H30N6O5	Lys-Trp-Asn	HC11,HC3,LC11,LC3
11.66	N/A	568.31	2.29	C30H43N5O6	Ile-Lys-Phe-Tyr	HC11,HC3,LC3
11.67	N/A	525.25	2.19	C26H34N6O6	Gln-Trp-Pro-Pro	HC11,LC11
11.71	N/A	271.19	-1.19	C15H28O4	[FA methyl(14:0/2:0)] 3-methyl-tetradecanedioic acid	HC11,HC3,LC11,LC3
11.72	N/A	433.26	2.70	C21H34N6O4	Leu-Phe-Arg	HC11,HC3,LC11
11.72	N/A	595.25	-0.23	C29H36N6O8	Gln-Thr-Trp-Tyr	HC11,HC3,LC11,LC3
11.73	N/A	419.24	1.45	C20H32N6O4	Phe-Val-Arg	HC11,HC3,LC11
11.75	N/A	563.25	-1.56	C24H49ClO8S2	14-chlorotetracosane-1,15-disulfate	HC11,LC11
11.76	N/A	295.13	-1.40	C19H20O3	4-Prenylresveratrol	HC11,HC3,LC11,LC3
11.76	N/A	235.04	1.67	C7H12N2O5S	Asp-Cys	HC11
11.80	N/A	215.16	-1.93	C12H24O3	12-Hydroxydodecanoic acid	HC3,LC11,LC3
11.80	N/A	507.21	1.87	C21H32N8O5S	Cys-Leu-His-His	HC11,HC3,LC11
11.82	N/A	449.25	-0.09	C25H38O7	Laserpitin	HC11,LC11
11.85	N/A	285.15	-2.85	C18H22O3	[ST hydroxy(3:0)] 3,16alpha-dihydroxy-estra-1,3,5(10)-trien-17-one	HC11
11.86	N/A	531.23	2.40	C29H32N4O6	Phe-Phe-Gly-Tyr	HC11,LC11
11.86	N/A	534.23	2.81	C23H33N7O8	Asn-Phe-Gln-Gln	HC11,HC3,LC11
11.88	N/A	547.23	-0.01	C20H36N8O8S	Arg-Met-Asp-Gln	HC11,LC11
11.90	N/A	157.12	-1.15	C9H18O2	Nonanoic acid	HC11,LC11
11.91	N/A	257.17	-1.37	C14H26O4	[FA (14:0/2:0)] Tetradecanedioic acid	HC11,HC3
11.93	N/A	503.26	2.60	C24H36N6O6	Ala-Lys-Thr-Trp	HC11,HC3,LC11
11.95	N/A	529.19	2.78	C25H30N4O9	Ala-Asp-Tyr-Tyr	HC11,HC3,LC11
11.95	N/A	450.30	-2.39	C22H46NO6P	[PC (14:1)] 1-(1E-tetradecenyl)-sn-glycero-3-phosphocholine	HC11
12.01	N/A	332.12	-2.00	C32H42O15	3,4,7-Trihydroxy-5-methoxy-8-prenylflavan 4-O-(beta-D-xylopyranosyl-(1->6)-beta-D-glucopyranoside)	HC11,HC3,LC11,LC3
12.05	N/A	515.08	-2.78	C16H26N2O13P2	dTDP-beta-L-rhodinose	HC11,HC3,LC11,LC3
12.14	N/A	456.29	0.76	C20H39N7O5	Ala-Leu-Val-Arg	HC11,HC3,LC3
12.14	N/A	557.28	-0.89	C32H38N4O5	Phe-Phe-Phe-Val	HC11,HC3,LC11,LC3

12.18	N/A	522.22	1.24	C23H33N5O9	Asn-Leu-Asp-Tyr	HC3,LC11,LC3
12.18	N/A	497.19	-1.24	C25H35O8Cl	[FA methyl,oxo,hydroxy(3:0/3:0)] methyl 5S,6S-diacetoxy-9-oxo-10-chloro-12R-hydroxy-7E,10E,14Z-prostatrienoate-cyclo[8,12R]	HC11,HC3,LC11,LC3
12.18	N/A	475.23	0.18	C26H36O8	all-trans-Retinoyl-beta-glucuronide	HC11,HC3,LC11,LC3
12.19	N/A	284.22	-2.21	C16H31NO3	Myristoylglycine	HC11,HC3,LC11,LC3
12.22	N/A	371.24	-1.65	C20H36O6	8,8a-Deoxyoleandolide	HC11,HC3,LC11,LC3
12.23	N/A	559.30	-2.96	C28H49O9P	PG(22:4(7Z,10Z,13Z,16Z)/0:0)	HC11,HC3,LC11,LC3
12.24	N/A	407.22	-2.31	C19H37O7P	Palmitoylglycerone phosphate	HC11
12.25	N/A	501.20	-0.34	C20H34N6O5S2	Cys-Leu-Met-His	HC11,HC3,LC11,LC3
12.25	N/A	524.22	2.29	C26H31N5O7	Ala-Trp-Ser-Tyr	HC11,HC3,LC11,LC3
12.28	N/A	538.23	1.58	C22H33N7O9	Arg-Asp-Ser-Tyr	HC11,HC3,LC11,LC3
12.28	N/A	399.22	-1.38	C24H32O5	[ST Triox] 3,7,12-Trioxo-5beta-chol-1-en-24-oic Acid	HC11,HC3,LC11,LC3
12.29	N/A	481.22	-1.40	C21H34N6O5S	His-Met-Val-Pro	HC11,HC3,LC11,LC3
12.30	N/A	429.20	0.03	C21H35O7P	PA(18:4(6Z,9Z,12Z,15Z)/0:0)	HC11,HC3,LC11,LC3
12.30	N/A	521.22	0.64	C26H30N6O6	Asn-Phe-Trp-Gly	HC11,HC3,LC11,LC3
12.32	N/A	527.20	2.32	C20H32N8O7S	Asn-Met-Gln-His	HC11,HC3,LC11
12.33	N/A	737.45	-2.82	C40H66O12	3-O-(Rhaa1-4Glcb)-26-O-(Glcb)-(25R)-spirost-5-en-3beta-ol	HC11,HC3,LC11,LC3
12.35	N/A	569.28	0.88	C25H46O14	2-O-(beta-D-galactopyranosyl-(1->6)-beta-D-galactopyranosyl) 2S,3R-dihydroxytridecanoic acid	HC11,HC3,LC11,LC3
12.36	N/A	488.22	-1.18	C20H35N5O7S	Asp-Lys-Met-Pro	HC11,HC3,LC11,LC3
12.39	N/A	359.19	-0.11	C21H28O5	Cortisone	HC11,HC3,LC11,LC3
12.40	N/A	365.23	-1.57	C21H34O5	Urocortisol	HC11,HC3,LC11
12.41	N/A	479.18	2.64	C19H28N8O5S	His-Met-Gly-His	HC11,HC3,LC11,LC3
12.43	N/A	649.32	1.10	C34H50O12	Thapsigargin	HC11,HC3,LC11
12.47	N/A	478.28	-0.20	C26H41NO7	Delcorine	HC11,HC3,LC11,LC3
12.47	N/A	584.23	-2.33	C28H35N5O9	Glu-Phe-Gln-Tyr	HC11,HC3,LC3
12.53	N/A	270.21	-0.24	C15H29NO3	Tridecanoylglycine	HC11,HC3,LC11,LC3
12.55	N/A	571.27	-0.27	C25H36N10O6	Arg-Phe-Asn-His	HC11,HC3,LC11,LC3
12.56	N/A	325.14	1.97	C20H22O4	[Fv Trihydrox] 2,,4,,6,-Trihydroxy-3,-prenyldihydrochalcone	HC11
12.60	N/A	607.25	0.31	C30H36N6O8	Glu-Phe-Trp-Gln	HC11,LC11,LC3

12.63	N/A	649.37	2.56	C62H112N2O26	Ganglioside GM2 (d18:0/12:0)	HC11,HC3,LC11,LC3
12.63	N/A	475.27	-2.00	C27H40O7	Cyclic-3,20-bis(1,2-ethanediyl acetal)-11alpha-(acetyloxy)-5alpha,6alpha-epoxypregnane-3,20-dione	HC11,LC11
12.64	N/A	410.23	-0.55	C18H38NO7P	[PC acetyl(8:2)] 1-octyl-2-acetyl-sn-glycero-3-phosphocholine	HC3,LC11,LC3
12.64	N/A	558.30	-0.75	C27H41N7O6	Asn-Leu-Lys-Trp	HC11,HC3,LC11,LC3
12.66	N/A	412.23	0.47	C17H31N7O5	Ala-Ala-Pro-Arg	HC3,LC11,LC3
12.67	N/A	433.28	0.60	C26H42O3S	[ST hydrox] (5Z,7E)-(1R,3R)-1,25-dihydroxy-9,10-seco-3-thia-5,7,10(19)-cholestatriene 3-oxide	HC11,HC3,LC11,LC3
12.67	N/A	328.25	-0.92	C18H35NO4	4-8dimethylnonanoylcarnitine	HC3,LC3
12.68	N/A	493.22	0.71	C29H34O7	Microlenin	HC11,HC3,LC11
12.70	N/A	549.27	-2.31	C25H38N6O8	Glu-Lys-Phe-Gln	HC11,HC3,LC11,LC3
12.76	N/A	345.17	-0.06	C20H26O5	Gibberellin A24	HC11,HC3,LC11,LC3
12.76	N/A	513.21	0.08	C24H30N6O7	Asp-Phe-Pro-His	HC11,HC3,LC3
12.78	N/A	467.18	1.53	C20H28N4O9	Ala-Thr-Asp-Tyr	HC11,HC3,LC11
12.80	N/A	371.16	0.98	C15H24N4O7	Ala-Ala-Asp-Pro	HC11,HC3,LC11,LC3
12.83	N/A	457.28	-1.72	C20H38N6O6	Ala-Leu-Lys-Gln	HC11,HC3,LC11,LC3
12.85	N/A	563.33	0.37	C26H44N8O6	Arg-Lys-Val-Tyr	HC11,HC3,LC11,LC3
12.92	N/A	482.31	2.79	C22H41N7O5	Arg-Leu-Val-Pro	HC11,HC3,LC11
12.93	N/A	585.27	1.09	C33H38N4O6	(3Z)-Phycocyanobilin	HC11,HC3,LC11,LC3
12.93	N/A	431.22	-1.52	C21H37O7P	PA(18:3(6Z,9Z,12Z)/0:0)	HC11,LC11,LC3
12.97	N/A	439.17	0.26	C19H28N4O6S	Met-Gln-Tyr	HC11,HC3,LC11,LC3
12.98	N/A	383.24	1.02	C17H32N6O4	Leu-Pro-Arg	HC11,HC3,LC3
12.99	N/A	331.23	-1.07	C21H32O3	17alpha,20alpha-Dihydroxypregn-4-en-3-one	HC11,HC3,LC11,LC3
12.99	N/A	499.17	-0.29	C18H28N8O7S	Asn-Cys-Gln-His	HC11,LC3
13.00	N/A	481.28	-0.44	C22H38N6O6	His-Leu-Leu-Thr	HC3,LC11,LC3
13.01	N/A	555.22	-1.01	C26H32N6O8	Asn-Phe-Asn-Tyr	HC11,HC3,LC11,LC3
13.01	N/A	514.27	-1.24	C20H37N9O7	Arg-Leu-Asn-Asn	HC11,HC3,LC11
13.01	N/A	249.06	2.48	C8H14N2O5S	gamma-L-Glutamyl-L-cysteine	HC11
13.06	N/A	577.31	-1.89	C26H42N8O7	Arg-Leu-Gln-Tyr	HC11,HC3,LC11,LC3
13.08	N/A	527.23	-0.15	C27H36N4O5S	Cys-Leu-Phe-Phe	HC11,HC3,LC11,LC3
13.10	N/A	413.17	-1.33	C15H26N8O4S	Cys-Arg-His	HC11,HC3,LC11,LC3

13.11	N/A	229.18	-1.60	C13H26O3	[FA hydroxy(13:0)] 2-hydroxy-tridecanoic acid	HC11,HC3,LC11,LC3
13.11	N/A	377.18	-0.72	C24H26O4	Albafuran A	HC11,HC3,LC11,LC3
13.12	N/A	411.16	-2.59	C21H26F2O6	Fluocinolone	HC11,HC3,LC11,LC3
13.14	N/A	500.21	2.44	C18H31N9O6S	Arg-Cys-Ser-His	HC11,HC3,LC11,LC3
13.16	N/A	313.24	-1.22	C18H34O4	[FA hydroxy(18:1)] 9,10-dihydroxy-12Z-octadecenoic acid	HC11,HC3,LC11,LC3
13.18	N/A	523.33	-1.70	C24H44N8O5	His-Leu-Lys-Lys	HC11,HC3,LC11,LC3
13.18	N/A	381.22	-0.21	C42H69O10P	PG(14:1(9Z)/22:6(4Z,7Z,10Z,13Z,16Z,19Z))	HC11,HC3,LC11,LC3
13.19	N/A	475.14	-1.46	C16H24N6O11	Asn-Asn-Asp-Asp	HC11
13.20	N/A	285.21	-0.78	C16H30O4	[FA hydroxy,oxo(16:0)] 9-hydroxy-16-oxo-hexadecanoic acid	HC11,HC3,LC11,LC3
13.22	N/A	436.23	-1.01	C19H31N7O5	His-Lys-Gly-Pro	HC11,HC3,LC11,LC3
13.22	N/A	271.19	-1.17	C15H28O4	[FA methyl(14:0/2:0)] 3-methyl-tetradecanedioic acid	HC11,HC3,LC11,LC3
13.23	N/A	487.19	-2.85	C17H28N8O9	Asn-Asn-Asn-Gln	HC11,HC3,LC11,LC3
13.24	N/A	441.16	0.04	C18H26N4O9	Asp-Asp-Pro-Pro	HC11,HC3,LC11,LC3
13.26	N/A	298.24	-1.50	C17H33NO3	Pentadecanoylglycine	HC11,HC3,LC11,LC3
13.32	N/A	561.27	0.64	C26H38N6O8	Glu-Lys-Thr-Trp	HC11,HC3,LC11,LC3
13.33	N/A	227.20	-1.36	C14H28O2	Tetradecanoic acid	HC11,HC3,LC11,LC3
13.34	N/A	377.18	-1.18	C18H26N4O5	Phe-Val-Asn	HC11,LC11,LC3
13.36	N/A	429.23	1.23	C21H30N6O4	Lys-Phe-His	HC11,LC11
13.37	N/A	585.29	-2.50	C26H38N10O6	Arg-Phe-Gln-His	HC11,LC11,LC3
13.37	N/A	417.14	-2.57	C14H22N6O9	Asn-Asn-Asp-Gly	HC11
13.37	N/A	502.23	-0.42	C21H37N5O7S	Glu-Lys-Met-Pro	HC11,HC3,LC11,LC3
13.39	N/A	537.31	-2.36	C30H42N4O5	Ile-Leu-Phe-Phe	HC11,HC3,LC3
13.39	N/A	506.23	1.62	C20H37N5O8S	Glu-Lys-Met-Thr	HC11,HC3,LC11,LC3
13.46	N/A	495.23	-1.60	C25H32N6O5	His-Phe-Pro-Pro	HC11,LC11
13.46	N/A	672.34	-2.64	C36H51NO11	Veratridine	HC11,HC3
13.47	N/A	283.19	-1.18	C16H28O4	(10S)-Juvenile hormone III diol	HC11,HC3,LC11,LC3
13.49	N/A	421.26	-1.29	C24H38O6	[ST Trihydroxy,ox] 3alpha,7beta,12alpha-Trihydroxy-6-oxo-5alpha-cholan-24-oic Acid	HC11,LC11
13.52	N/A	460.28	1.56	C20H39N5O7	Ile-Lys-Thr-Thr	HC11,LC3
13.55	N/A	482.24	1.04	C20H33N7O7	Arg-Asp-Pro-Pro	HC11,LC11
13.56	N/A	349.05	1.69	C9H19O12P	nonulose 9-phosphate	HC11,HC3,LC11,LC3

13.57	N/A	493.19	-1.92	C18H34N6O6S2	Asn-Lys-Met-Cys	HC11,HC3,LC11
13.71	N/A	477.20	-1.93	C22H30N4O8	Asp-Phe-Thr-Pro	HC11,LC11
13.71	N/A	384.27	-1.08	C21H39NO5	3-Hydroxy-cis-5-tetradecenoylcarnitine	HC11,LC11,LC3
13.72	N/A	355.21	-1.96	C19H32O6	[FA methyl(18:2)] methyl 9-hydroperoxy-10,12-epidioxy-13,15-octadecadienoate	HC11,HC3,LC11,LC3
13.79	N/A	817.50	0.41	C46H75O10P	PG(40:8)	HC11,HC3,LC11,LC3
13.83	N/A	441.13	2.93	C22H22N2O8	12-Dehydrotetracycline	HC11,HC3,LC11,LC3
13.84	N/A	461.21	-2.91	C16H30N8O8	Arg-Asn-Ser-Ser	HC11,HC3,LC11,LC3
13.85	N/A	461.19	0.37	C16H30N8O6S	Ala-Asn-Cys-Arg	HC11,HC3,LC11
13.85	N/A	447.13	-0.52	C22H24O10	[Fv] Homobutein 4-glucoside	HC11,LC11,LC3
13.86	N/A	446.17	-0.27	C17H29N5O7S	Asn-Met-Pro-Ser	HC11,LC3
13.87	N/A	288.10	0.40	C11H19N3O4S	Ala-Cys-Pro	HC11,HC3,LC11,LC3
13.87	N/A	481.15	0.34	C27H28FN2OClS	SSR 125543	HC11,HC3,LC11,LC3
13.91	N/A	515.12	-2.75	C25H24O12	Formononetin 7-O-glucoside-6,,-O-malonate	HC11,HC3,LC3
13.93	N/A	553.29	-0.97	C25H46O13	2-O-(beta-D-galactopyranosyl-(1->6)-beta-D-galactopyranosyl) 2S-hydroxytridecanoic acid	HC11,HC3,LC11,LC3
13.95	N/A	121.00	-0.46	C3H6O3S	3-Mercaptolactate	HC11,LC11,LC3
13.95	N/A	475.19	-0.56	C21H28N6O7	Ala-Ser-Tyr-His	HC11,LC11,LC3
13.95	N/A	401.18	0.45	C15H26N6O7	Gln-Gln-Gln	HC11,HC3,LC11,LC3
14.04	N/A	710.32	-0.06	C63H106O35	Capsicoside A	HC11,HC3,LC11,LC3
14.06	N/A	442.24	0.28	C18H33N7O6	Ala-Thr-Pro-Arg	HC11,HC3,LC11,LC3
14.07	N/A	573.22	0.40	C28H38N4O5S2	Met-Met-Phe-Phe	HC11,HC3,LC11,LC3
14.07	N/A	328.25	-0.69	C18H35NO4	4-8dimethylnonanoylcarnitine	HC3,LC11,LC3
14.10	N/A	256.23	-0.58	C15H31NO2	Decanoylcholine	LC11,LC3
14.14	N/A	499.33	1.96	C23H44N6O6	Gln-Leu-Leu-Lys	HC11
14.15	N/A	464.22	1.74	C26H31N3O5	Codonocarpine	HC11,HC3,LC11,LC3
14.16	N/A	418.18	1.04	C16H29N5O6S	Ala-Leu-Asn-Cys	HC11,HC3,LC11,LC3
14.18	N/A	480.27	0.51	C22H44NO8P	[PC (10:0/4:2)] 1-decanoyl-2-butyryl-sn-glycero-3-phosphocholine	HC3,LC11,LC3
14.18	N/A	492.30	-1.71	C27H43NO7	Zygadenine	HC11,HC3,LC11
14.19	N/A	614.27	-1.63	C32H37N7O6	Gln-Trp-Trp-Pro	HC11,HC3,LC11,LC3

14.21	N/A	377.20	-2.93	C37H56N8O9	Nostocyclopeptide A1	HC11,LC11,LC3
14.22	N/A	136.99	-0.82	C3H6O4S	3-sulfopropanal	HC11,LC11,LC3
14.22	N/A	491.19	-0.12	C18H32N6O8S	Asn-Met-Thr-Gln	HC11,LC11,LC3
14.22	N/A	611.26	0.52	C33H36N6O6	Asn-Phe-Phe-Trp	HC11,HC3,LC11
14.22	N/A	391.18	0.51	C21H28O7	LCuiestenin	HC11,HC3,LC11
14.23	N/A	407.28	-0.26	C24H40O5	Cholate	HC11,HC3,LC11
14.26	N/A	529.24	0.75	C20H34N8O9	Gln-Gln-Gln-Gln	HC11,HC3,LC11
14.26	N/A	512.22	1.53	C19H31N9O8	Arg-Asp-Ser-His	HC3,LC11,LC3
14.28	N/A	530.26	0.88	C26H37N5O7	Asp-Leu-Trp-Val	HC11,HC3,LC11,LC3
14.30	N/A	312.25	-0.97	C18H35NO3	[FA (16:0)] N-hexadecanoyl-glycine	HC11,HC3,LC3
14.31	N/A	457.17	-0.93	C22H26N4O7	Asn-Tyr-Tyr	HC11,HC3,LC11
14.31	N/A	481.28	0.46	C30H42O3S	[ST (3:0/3:0)] (7E,16Z)-(1S,3R)-23-thia-24,24b-clycobenzyl-19-nor-24a,24b-dihomo-9,10-seco-5,7,16-cholestatriene-1,3,25-triol	HC11,HC3,LC11,LC3
14.35	N/A	490.28	0.47	C23H37N7O5	Ala-Phe-Val-Arg	HC11,HC3,LC11,LC3
14.35	N/A	315.25	-1.33	C18H36O4	[FA hydroxy(18:0)] 9,10-dihydroxy-octadecanoic acid	HC11,HC3,LC11,LC3
14.41	N/A	285.21	-1.08	C16H30O4	[FA hydroxy,oxo(16:0)] 9-hydroxy-16-oxo-hexadecanoic acid	HC11,HC3,LC11,LC3
14.41	N/A	563.33	0.97	C26H44N8O6	Arg-Lys-Val-Tyr	HC11,HC3,LC11,LC3
14.44	N/A	448.27	1.33	C21H35N7O4	Lys-Phe-Arg	HC11,HC3,LC11
14.45	N/A	689.42	2.45	C39H63O8P	PA(14:1(9Z)/22:6(4Z,7Z,10Z,13Z,16Z,19Z))	HC11,HC3,LC11,LC3
14.46	N/A	461.25	2.73	C22H34N6O5	His-Leu-Pro-Pro	HC11,HC3,LC11,LC3
14.47	N/A	228.20	-1.66	C13H27NO2	2S-Amino-tridecanoic acid	LC11
14.47	N/A	431.24	-0.94	C25H36O6	Glycinoeclepin A	HC11
14.49	N/A	599.32	-1.23	C30H44N6O7	Lys-Lys-Tyr-Tyr	HC11,HC3,LC11,LC3
14.50	N/A	496.26	0.86	C20H35N9O6	Arg-Val-Ser-His	HC11,HC3,LC11,LC3
14.51	N/A	545.24	-1.65	C25H34N6O8	Asn-Leu-Trp-Asp	HC11,HC3,LC11
14.52	N/A	529.22	1.31	C27H34N2O9	3-alpha(S)-Strictosidine	HC11,LC11
14.53	N/A	511.18	-2.91	C22H32N4O8S	Asp-Leu-Cys-Tyr	HC11,HC3,LC11,LC3
14.55	N/A	317.18	0.07	C19H26O4	6beta-Hydroxy-D-homo-17a-oxaandrost-4-ene-3,17-dione	HC11
14.55	N/A	533.24	2.05	C24H34N6O8	Asp-Lys-Trp-Ser	HC11,HC3,LC11
14.55	N/A	562.26	2.52	C25H37N7O8	Glu-Pro-Tyr-Arg	HC11,HC3,LC11,LC3

14.58	N/A	516.22	2.01	C23H31N7O7	Ala-Gln-Tyr-His	HC11,LC11,LC3
14.58	N/A	323.03	-0.49	C9H13N2O9P	UMP	HC11,HC3,LC11,LC3
14.60	N/A	455.24	0.50	C18H32N8O6	Ala-Asn-Pro-Arg	HC11,HC3,LC11,LC3
14.61	N/A	459.20	2.56	C16H28N8O8	Arg-Asn-Asp-Gly	HC11,HC3,LC11,LC3
14.62	N/A	512.26	0.74	C20H35N9O7	Arg-Asn-Gln-Pro	HC11,HC3,LC11,LC3
14.62	N/A	329.13	-2.87	C20H23O2Cl	17alpha-Chloroethynylestradiol	HC11,HC3,LC3
14.62	N/A	243.20	-1.56	C14H28O3	2S-Hydroxytetradecanoic acid	HC11,HC3,LC11
14.63	N/A	302.12	-0.35	C12H21N3O4S	Met-Gly-Pro	HC11,HC3,LC11,LC3
14.64	N/A	355.25	-2.64	C20H36O5	Prostaglandin F1alpha	HC11,HC3,LC11,LC3
14.68	N/A	489.21	-1.12	C17H30N8O9	Arg-Asn-Asp-Ser	HC11,HC3,LC11
14.68	N/A	349.16	1.69	C20H27O3Cl	9-Chloro-17beta-hydroxy-17-methylandrost-4-ene-3,11-dione	HC11,LC11,LC3
14.69	N/A	527.23	-2.01	C22H36N6O7S	Glu-Leu-Met-His	HC11,HC3,LC11,LC3
14.71	N/A	851.04	2.61	C20H28N10O20P4	Guanosinetetraphosphateadenosine	HC11,HC3,LC11,LC3
14.71	N/A	537.31	0.13	C30H42N4O5	Ile-Leu-Phe-Phe	HC11,HC3,LC11,LC3
14.72	N/A	588.28	-0.16	C27H39N7O8	Glu-Lys-Trp-Gln	HC11,HC3,LC3
14.74	N/A	449.25	-1.43	C25H38O7	Laserpitin	HC11,HC3,LC11
14.75	N/A	313.18	-1.91	C20H26O3	momilactone A	HC11
14.76	N/A	519.15	-2.17	C25H28O12	[Fv hydroxy(2:0)] 4,2,-Dihydroxy-4,,6,-diacetoxydihydrochalcone 2,-O-glucoside	HC11,HC3,LC11,LC3
14.77	N/A	427.17	1.53	C20H24N6O5	Trp-Ser-His	HC11,HC3,LC11,LC3
14.77	N/A	554.29	2.98	C23H41N9O5S	Arg-Leu-Met-His	HC11,HC3,LC11
14.80	N/A	438.24	-2.57	C44H80O13P2	PGP(18:1(11Z)/20:3(8Z,11Z,14Z))	HC3,LC11,LC3
14.83	N/A	544.25	-2.59	C25H35N7O7	Asn-Leu-Trp-Asn	HC11,HC3,LC3
14.85	N/A	467.24	-0.58	C19H32N8O6	Ala-Lys-Asn-His	HC11,HC3,LC11
14.85	N/A	557.34	2.82	C40H46O2	χ,χ-caroten-18-oate	HC11,HC3,LC3
14.85	N/A	437.25	0.37	C20H34N6O5	Ala-Leu-Val-His	HC11,HC3,LC11
14.88	N/A	479.24	-0.25	C29H36O6	[PR] (-)-Jolkinol A	HC11,HC3,LC11,LC3
14.89	N/A	489.21	-0.59	C22H30N6O7	Ala-Thr-Trp-Asn	HC11,HC3,LC11,LC3
14.93	N/A	185.15	-2.34	C11H22O2	[FA (11:0)] undecanoic acid	HC11,LC11
14.94	N/A	303.20	-0.44	C19H28O3	19-Hydroxytestosterone	HC11
14.96	N/A	233.15	-0.93	C15H22O2	[PR] 1,13-Dihydroxy-herbertene	HC11,HC3,LC11,LC3

14.98	N/A	517.15	-1.05	C19H30N6O7S2	Glu-Met-Cys-His	HC11,HC3,LC11,LC3
14.99	N/A	567.31	-0.30	C22H40N12O6	Arg-Thr-Arg-His	HC11,HC3,LC11
15.00	N/A	550.26	1.85	C23H37N9O5S	His-Lys-Met-His	HC11,HC3,LC3
15.00	N/A	515.22	-1.71	C19H32N8O9	Asn-Gln-Gln-Gln	HC11,HC3,LC11,LC3
15.03	N/A	526.24	-0.78	C20H33N9O8	Arg-Thr-Asp-His	HC11,HC3,LC11,LC3
15.04	N/A	649.37	2.63	C62H112N2O26	Ganglioside GM2 (d18:0/12:0)	HC11,HC3,LC11
15.05	N/A	528.24	2.51	C20H35N9O6S	Arg-Met-Ser-His	HC11,HC3,LC11,LC3
15.05	N/A	560.31	2.21	C24H39N11O5	Arg-Leu-His-His	HC11,HC3,LC11,LC3
15.07	N/A	525.28	1.66	C22H38N8O7	Asp-Lys-Lys-His	HC11,HC3,LC11,LC3
15.08	N/A	482.29	0.35	C22H46NO8P	PS(O-16:0/0:0)	HC11,HC3,LC11,LC3
15.08	N/A	511.31	1.70	C28H48O6S	24-epicathasterone-22-O-sulfate	HC3,LC11,LC3
15.08	N/A	326.27	-0.52	C19H37NO3	Margaroylglycine	HC11,HC3,LC3
15.10	N/A	510.27	-1.05	C27H37N5O5	Ala-Lys-Phe-Phe	HC11,LC11
15.11	N/A	587.29	-1.13	C33H40N4O6	D-Urobilin	HC11,HC3,LC11,LC3
15.15	N/A	435.15	2.34	C25H24O7	[Fv Hydroxy,dimethoxy,hydroxy,methoxy(9:1)] 5-Hydroxy-7,8-dimethoxy-6-(2-hydroxy-5-methoxybenzyl)flavanone	HC11,HC3,LC11
15.15	N/A	551.31	0.12	C23H40N10O6	Arg-Leu-Gln-His	HC11,HC3,LC11,LC3
15.15	N/A	399.17	2.69	C17H28N4O5S	Met-Gly-Pro-Pro	HC11,HC3,LC11,LC3
15.16	N/A	627.28	1.49	C28H40N10O5S	Arg-Met-Trp-His	HC11,HC3,LC11,LC3
15.18	N/A	535.29	1.85	C29H44O9	Rhodexin A	HC11,HC3,LC11
15.20	N/A	593.30	1.84	C28H38N10O5	Arg-Trp-Pro-His	HC11,HC3,LC11,LC3
15.20	N/A	585.26	0.49	C30H34N8O5	His-Phe-Phe-His	HC11,HC3,LC3
15.23	N/A	583.26	2.46	C33H36N4O6	Bilirubin	HC11,HC3,LC11,LC3
15.23	N/A	537.20	2.51	C23H34N6O5S2	Cys-Lys-Trp-Cys	HC11,HC3,LC11,LC3
15.24	N/A	375.25	2.79	C23H36O4	3-Acetyl-5alpha-androstane-3beta,17beta-diol 3-acetate	HC11,HC3,LC11,LC3
15.25	N/A	715.33	1.32	C40H53O8SNa	[PR] 4-Ketonostoxanthin 3-sulfate	HC11,HC3,LC11,LC3
15.29	N/A	625.28	-0.27	C34H38N6O6	Gln-Phe-Phe-Trp	HC11,HC3,LC11,LC3
15.33	N/A	453.24	0.67	C21H34N4O7	Glu-Leu-Pro-Pro	HC11,HC3,LC11,LC3
15.33	N/A	327.20	-2.87	C21H28O3	9,11alpha-Epoxypregn-4-ene-3,20-dione	HC11,HC3,LC11,LC3
15.37	N/A	126.90	-1.11	HI	hydrogen iodide	HC11,HC3,LC11,LC3
15.37	N/A	464.30	-1.89	C26H43NO6	Glycocholate	HC11,HC3,LC11,LC3

15.39	N/A	308.20	-0.06	C36H58O8	soyasapogenol-E-3-O-beta-glucoside	HC11,HC3,LC11,LC3
15.39	N/A	535.20	2.95	C23H32N6O7S	Asn-Met-Trp-Ser	HC11,HC3,LC3
15.39	N/A	659.31	1.97	C36H44N4O8	Coproporphyrinogen III	HC11,HC3,LC11,LC3
15.41	N/A	611.33	-0.49	C30H44N8O6	Arg-Lys-Phe-Tyr	HC11,HC3,LC11
15.42	N/A	376.25	-1.61	C22H35NO4	Karakoline	HC11
15.43	N/A	317.21	0.56	C20H30O3	Leukotriene A4	HC11,HC3,LC11,LC3
15.43	N/A	446.26	0.55	C19H37N5O7	Ile-Lys-Thr-Ser	HC11
15.45	N/A	494.29	-2.78	C23H46NO8P	[PC (7:0/8:0)] 1-heptanoyl-2-octanoyl-sn-glycero-3-phosphocholine	HC11,HC3,LC11,LC3
15.45	N/A	572.30	-2.71	C25H39N11O5	Arg-Trp-Gly-Arg	HC3,LC3
15.46	N/A	523.15	0.29	C19H32N4O9S2	Glu-Met-Met-Asp	HC11
15.47	N/A	480.27	-1.02	C22H44NO8P	[PC (10:0/4:2)] 1-decanoyl-2-butyryl-sn-glycero-3-phosphocholine	HC11,HC3,LC11,LC3
15.47	N/A	500.28	0.80	C25H44NO7P	[PE (20:4)] 1-(5Z,8Z,11Z,14Z-eicosatetraenoyl)-sn-glycero-3-phosphoethanolamine	HC11,HC3,LC11,LC3
15.47	N/A	496.29	-2.26	C22H39N7O6	His-Leu-Lys-Thr	HC11,HC3,LC11,LC3
15.49	N/A	600.33	1.23	C28H43N9O6	Arg-Leu-Trp-Gln	HC11,HC3,LC3
15.49	N/A	615.34	0.31	C28H44N10O6	Arg-Lys-Trp-Gln	HC11,HC3,LC11,LC3
15.51	N/A	409.22	2.61	C18H30N6O5	Ala-Leu-Ala-His	HC11,HC3,LC11
15.53	N/A	379.19	1.99	C17H33O7P	PA(14:1(9Z)/0:0)	HC11,HC3,LC3
15.53	N/A	363.25	-1.58	C22H36O4	[FA oxo,hydroxy,dimethyl(2:0)] 9-oxo-15R-hydroxy-16,16-dimethyl-5Z,13E-prostadienoic acid	HC11,LC3
15.53	N/A	492.22	0.02	C18H35N7O7S	Arg-Met-Thr-Ser	HC11,HC3,LC11,LC3
15.56	N/A	401.29	1.61	C18H38N6O4	Lys-Lys-Lys	HC11,HC3,LC3
15.56	N/A	257.21	-0.82	C15H30O3	[FA hydroxy(15:0)] 2-hydroxy-pentadecanoic acid	HC11,HC3,LC11,LC3
15.57	N/A	399.22	-2.80	C24H32O5	[ST Triox] 3,7,12-Trioxo-5beta-chol-1-en-24-oic Acid	HC11,HC3,LC11,LC3
15.57	N/A	421.26	0.25	C24H38O6	[ST Trihydroxy,ox] 3alpha,7beta,12alpha-Trihydroxy-6-oxo-5alpha-cholan-24-oic Acid	HC11,LC11
15.59	N/A	511.34	2.90	C32H48O5	[PR (3:0)] (+)-22-acetoxy-3alpha-hydorxy-lanosta-7,9(11),24-trien-26-oic acid	HC11,HC3,LC3
15.61	N/A	515.27	2.04	C24H36N8O5	Arg-Trp-Val-Gly	HC11,HC3,LC11,LC3
15.63	N/A	381.22	-2.71	C42H69O10P	PG(14:1(9Z)/22:6(4Z,7Z,10Z,13Z,16Z,19Z))	HC11,HC3,LC11,LC3

15.64	N/A	580.31	1.36	C22H39N13O6	Arg-Asn-Arg-His	HC11,HC3,LC11
15.65	N/A	510.28	-2.96	C23H46NO9P	PS(17:0/0:0)	HC11,HC3,LC11,LC3
15.69	N/A	630.29	-2.49	C33H45NO11	Mesaconitine	HC11,HC3,LC11,LC3
15.74	N/A	488.18	1.07	C19H31N5O8S	Asp-Met-Gln-Pro	HC11,HC3,LC11,LC3
15.75	N/A	357.26	-1.05	C20H38O5	[FA hydroxy(18:0)] 9,13-dihydroxy-10-ethoxy-11-octadecenoic acid	HC11,LC11,LC3
15.80	N/A	481.28	1.15	C30H42O3S	[ST (3:0/3:0)] (7E,16Z)-(1S,3R)-23-thia-24,24b-clycobenzyl-19-nor-24a,24b-dihomo-9,10-seco-5,7,16-cholestatriene-1,3,25-triol	HC11,HC3,LC11
15.84	N/A	345.21	1.23	C22H26N4	(-)-Chimonanthine	HC11,HC3,LC11,LC3
15.86	N/A	297.24	0.53	C18H34O3	2-Oxooctadecanoic acid	HC11,HC3,LC11,LC3
15.86	N/A	501.18	-0.35	C19H34O15	Isoglobotriaose	HC11,HC3,LC11,LC3
15.89	N/A	472.24	-1.82	C20H35N5O8	Asn-Leu-Leu-Asp	HC11,HC3,LC11,LC3
15.90	N/A	336.23	-1.65	C20H33O4	6,7-dihydro-12-epi-LTB4	HC11
15.92	N/A	523.20	0.44	C22H32N6O7S	Asn-Met-Phe-Asn	HC11,HC3,LC11,LC3
15.98	N/A	391.21	-0.34	C18H28N6O4	Phe-Ala-Arg	HC11,HC3,LC11,LC3
16.01	N/A	589.28	0.81	C36H38N4O4	DimethylprotoporphyrinIXdimethylester	HC11,HC3,LC11,LC3
16.05	N/A	359.22	1.11	C22H32O4	[FA hydroxy(22:0/6:0)] 10R,17S-dihydroxy-docosa-4Z,7Z,11E,13E,15Z,19Z-hexaenoic acid	HC11,HC3,LC11
16.05	N/A	649.37	2.67	C62H112N2O26	Ganglioside GM2 (d18:0/12:0)	HC3,LC11
16.09	N/A	269.21	-1.32	C16H30O3	[FA oxo(16:0)] 3-oxo-hexadecanoic acid	HC11,HC3,LC11
16.11	N/A	265.15	-1.60	C12H26O4S	sodium dodecyl sulfate	HC11,HC3,LC11,LC3
16.19	N/A	529.31	1.44	C22H42N8O7	Arg-Leu-Lys-Asp	HC11,HC3,LC11,LC3
16.20	N/A	449.22	-2.73	C25H30N4O4	Phe-Trp-Val	HC3,LC11,LC3
16.23	N/A	412.31	-0.80	C23H43NO5	3-Hydroxy-9-hexadecenoylcarnitine	HC11,HC3,LC11
16.23	N/A	199.17	-2.04	C12H24O2	Dodecanoic acid	HC11,LC11,LC3
16.25	N/A	105.00	1.77	C3H6O2S	S-Methylthioglycolate	LC11
16.25	N/A	508.30	-0.96	C24H48NO8P	[PC acetyl(14:0)] 1-tetradecanoyl-2-acetyl-sn-glycero-3-phosphocholine	HC11,HC3,LC11,LC3
16.26	N/A	587.29	-0.70	C33H40N4O6	D-Urobilin	HC11,HC3,LC11,LC3
16.27	N/A	405.16	0.62	C19H26N4O4S	Met-Trp-Ala	HC11,HC3,LC11,LC3

16.27	N/A	423.27	-0.78	C24H40O6	[ST hydroxy(4:0)] 1beta,3alpha,7alpha,12alpha-Tetrahydroxy-5beta-cholan-24-oic Acid	HC11,HC3,LC11
16.28	N/A	537.27	2.03	C24H38N6O8	Gln-Lys-Thr-Tyr	HC11,HC3,LC11,LC3
16.36	N/A	494.24	-2.71	C26H33N5O5	Lys-Trp-Tyr	HC11,HC3,LC11,LC3
16.41	N/A	538.21	-0.98	C22H33N7O7S	Arg-Phe-Asp-Cys	HC11,LC3
16.42	N/A	788.98	-1.59	C18H26N4O23P4	P1,P4-Bis(5,-uridyl) tetraphosphate	HC11,HC3,LC11,LC3
16.42	N/A	547.16	-2.34	C30H28O10	[Fv] 3,4-Dihydroxyrottlerin	HC11
16.43	N/A	512.36	-1.32	C24H47N7O5	Arg-Leu-Leu-Leu	HC11,LC3
16.47	N/A	643.34	1.86	C28H44N12O6	Arg-Trp-Gln-Arg	HC11,HC3,LC3
16.50	N/A	489.21	2.92	C17H30N8O9	Arg-Asn-Asp-Ser	HC11,HC3,LC11,LC3
16.52	N/A	524.30	-0.41	C24H48NO9P	[PS (18:0)] 1-octadecanoyl-sn-glycero-3-phosphoserine	HC11,HC3,LC11,LC3
16.56	N/A	499.27	-1.86	C25H36N6O5	Ala-Lys-Trp-Pro	HC11,HC3,LC11,LC3
16.57	N/A	548.33	-2.73	C27H52NO8P	[PC (16:0/3:0)] 1-hexadecanoyl-2-(2E-propionyl)-sn-glycero-3-phosphocholine	HC11,LC11,LC3
16.60	N/A	455.22	0.86	C25H32N2O6	Vindoline	HC11,HC3,LC11,LC3
16.61	N/A	673.43	0.38	C35H58N6O7	N-Acetyl-leu-leu-leu-leu-tyr-amide	HC11,LC3
16.61	N/A	373.20	-2.60	C22H30O5	11beta-20-Dihydroxy-3-oxopregn-4-en-21-oicacid	HC11,HC3,LC11,LC3
16.64	N/A	443.23	1.35	C20H36N4O5S	Cys-Leu-Leu-Pro	HC11,HC3,LC11,LC3
16.68	N/A	464.28	-1.37	C22H44NO7P	[PC (14:1)] 1-(9Z-tetradecenoyl)-sn-glycero-3-phosphocholine	HC11,HC3,LC11,LC3
16.68	N/A	717.45	2.61	C41H67O8P	PA(38:7)	HC11,LC11
16.70	N/A	341.23	-2.15	C19H34O5	[FA methyl,hydroxy,oxo(18:0)] methyl 9,12-dihydroxy-13-oxo-10-octadecenoate	HC11,HC3,LC11
16.70	N/A	524.24	-2.80	C28H35N3O7	Virginiamycin M1	HC11,HC3,LC11,LC3
16.70	N/A	313.24	-1.09	C18H34O4	[FA hydroxy(18:1)] 9,10-dihydroxy-12Z-octadecenoic acid	HC11,HC3,LC11,LC3
16.71	N/A	498.26	-0.27	C25H42NO7P	LysoPE(0:0/20:5(5Z,8Z,11Z,14Z,17Z))	HC11,HC3,LC11,LC3
16.71	N/A	535.25	0.27	C23H36N8O5S	His-Leu-Met-His	HC11,HC3,LC11,LC3
16.77	N/A	540.28	-0.90	C24H47NO10S	Psychosine sulfate	HC11,HC3,LC11
16.79	N/A	679.44	-0.80	C35H69O8PS	[GP (14:0/14:0)] 1,2-ditetradecanoyl-sn-glycero-3-phosphosulfocholine	HC11,HC3,LC11,LC3
16.79	N/A	387.25	-1.99	C24H36O4	[ST hydroxy(3:0)] (5Z,7E)-(1S,3R)-1,3-dihydroxy-9,10-seco-5,7,10(19)-cholatrien-24-oic acid	HC11,HC3,LC11
16.89	N/A	437.18	1.21	C23H26N4O5	Phe-Trp-Ser	HC11,HC3,LC11,LC3

16.89	N/A	435.18	-1.75	C16H32N6O4S2	Met-Met-Arg	HC11,HC3,LC11,LC3
16.94	N/A	461.25	0.49	C22H34N6O5	His-Leu-Pro-Pro	HC11,HC3,LC11
16.96	N/A	499.30	-0.42	C21H40N8O6	Arg-Leu-Val-Asn	HC11,HC3,LC11
16.97	N/A	698.40	-2.80	C36H62NO10P	PS(12:0/18:4(6Z,9Z,12Z,15Z))	HC11,HC3,LC3
16.97	N/A	341.27	-2.21	C20H38O4	[FA (20:0/2:0)] Eicosanedioic acid	HC11,HC3,LC11,LC3
16.97	N/A	510.32	-1.65	C24H50NO8P	PS(O-18:0/0:0)	HC11,HC3,LC11,LC3
16.99	N/A	459.25	-0.18	C23H41O7P	PA(20:3(8Z,11Z,14Z)/0:0)	HC11,HC3
17.04	N/A	404.22	2.47	C22H27N7O	Cypridina luciferin	HC11
17.06	N/A	395.18	0.30	C18H28N4O4S	Lys-Phe-Cys	HC11
17.06	N/A	645.35	2.30	C34H46N8O5	Lys-Lys-Trp-Trp	HC11,HC3,LC11,LC3
17.07	N/A	449.29	0.96	C22H38N6O4	Chaksine	HC11,HC3,LC11
17.08	N/A	431.15	-2.19	C15H24N6O9	Ala-Asn-Asn-Asp	HC11,HC3,LC11,LC3
17.09	N/A	447.18	-1.03	C16H28N6O9	Asn-Thr-Gln-Ser	HC11
17.09	N/A	551.31	2.20	C23H40N10O6	Arg-Leu-Gln-His	HC11,HC3,LC11,LC3
17.14	N/A	297.24	-1.55	C18H34O3	2-Oxooctadecanoic acid	HC11,HC3,LC11,LC3
17.14	N/A	299.26	-1.23	C18H36O3	[FA hydroxy(18:0)] 2S-hydroxy-octadecanoic acid	HC11,HC3,LC11,LC3
17.14	N/A	377.26	-2.42	C42H77O9P	PG(O-18:0/18:4(6Z,9Z,12Z,15Z))	HC3,LC11
17.14	N/A	333.22	-1.23	C18H35ClO3	9-chloro-10-hydroxy-octadecanoic acid	HC11,HC3,LC11
17.17	N/A	472.24	-2.74	C20H35N5O8	Asn-Leu-Leu-Asp	HC11,HC3,LC11,LC3
17.17	N/A	373.19	-0.86	C16H30N4O4S	Lys-Met-Pro	HC11,HC3,LC11
17.18	N/A	271.23	-0.87	C16H32O3	16-hydroxypalmitate	HC11,HC3,LC11,LC3
17.18	N/A	283.23	-0.95	C17H32O3	[FA methoxy(16:1)] 2-methoxy-5Z-hexadecenoic acid	HC11,HC3,LC11,LC3
17.22	N/A	595.34	2.79	C30H44N8O5	Arg-Lys-Phe-Phe	HC11,HC3,LC11,LC3
17.23	N/A	491.30	0.64	C24H40N6O5	Ala-Lys-Lys-Phe	HC11,HC3,LC11,LC3
17.23	N/A	396.22	0.52	C17H36NO7P	[PC (9:0)] 1-nonanoyl-sn-glycero-3-phosphocholine	HC11
17.25	N/A	653.30	0.61	C29H46N6O9S	aeruginosin 98-b	HC11,HC3,LC3
17.28	N/A	272.19	0.02	C14H27NO4	Heptanoylcarnitine	HC11,HC3,LC11,LC3
17.38	N/A	520.33	-1.74	C29H47NO7	Mycinamicin VII	HC11,HC3,LC11,LC3
17.38	N/A	523.26	-0.83	C27H38F6O3	[ST (6:0/3:0)] (5Z,7E)-(1S,3R)-26,26,26,27,27,27-hexafluoro-9,10-seco-5,7,10(19)-cholestatriene-1,3,24-triol	HC11,HC3
17.38	N/A	505.23	1.15	C24H34N4O8	Asp-Leu-Pro-Tyr	HC11,HC3,LC11,LC3

17.40	N/A	485.25	0.14	C19H34N8O7	Arg-Gln-Pro-Ser	HC11,HC3,LC11,LC3
17.41	N/A	507.27	-2.41	C24H45O9P	1-18:2-lysophosphatidylglycerol	HC11,HC3,LC11
17.46	N/A	435.25	-1.94	C21H41O7P	LPA(0:0/18:1(9Z))	HC11,HC3,LC11,LC3
17.46	N/A	467.20	1.25	C21H32N4O6S	Ala-Leu-Cys-Tyr	HC11,HC3,LC11,LC3
17.46	N/A	437.25	-0.70	C20H34N6O5	Ala-Leu-Val-His	HC11,HC3,LC11,LC3
17.49	N/A	594.36	-0.32	C24H45N13O5	Arg-Lys-Arg-His	HC11,HC3,LC11
17.50	N/A	579.34	-0.42	C28H52O12	alpha,alpha,-Trehalose 6-palmitate	HC11,HC3,LC11,LC3
17.55	N/A	337.20	-0.99	C20H28F2O2	4,4-Difluoro-17beta-hydroxy-17alpha-methyl-androst-5-en-3-one	HC11,HC3,LC11,LC3
17.55	N/A	503.17	-1.20	C18H28N6O11	Asp-Asp-Gln-Gln	HC11,HC3,LC11
17.59	N/A	299.22	-0.28	C17H32O4	[FA methyl(16:0/2:0)] 2-methyl-hexadecanedioic acid	HC11,HC3,LC11,LC3
17.62	N/A	461.27	-2.14	C23H43O7P	PA(20:2(11Z,14Z)/0:0)	HC11,HC3,LC11
17.65	N/A	524.27	-2.66	C24H39N5O8	Desmosine	HC11,HC3,LC11,LC3
17.66	N/A	538.31	0.09	C25H50NO9P	PS(19:0/0:0)	HC11,HC3,LC11,LC3
17.67	N/A	467.30	2.03	C22H40N6O5	Lys-Lys-Pro-Pro	HC11,HC3,LC11,LC3
17.75	N/A	409.18	2.24	C20H30N2O5S	Benfuracarb	HC11,HC3,LC11,LC3
17.81	N/A	485.27	0.80	C25H43O7P	PA(20:4(5Z,8Z,11Z,14Z)e/2:0)	HC11,HC3,LC11,LC3
17.82	N/A	407.20	-1.21	C18H28N6O5	Ala-Tyr-Arg	HC11,HC3,LC11,LC3
17.84	N/A	635.39	-0.35	C32H61O10P	PG(12:0/14:1(9Z))	HC11,HC3,LC3
17.94	N/A	715.43	0.47	C41H65O8P	PA(38:8)	HC11,HC3,LC11,LC3
17.94	N/A	445.27	2.48	C20H38N4O7	Ile-Leu-Thr-Thr	HC11
17.95	N/A	403.14	0.65	C21H24O8	[Fv methoxy(6:0/9:1)] 5,6,7,3,,4,,5,-Hexamethoxyflavanone	HC11,HC3,LC11,LC3
17.97	N/A	336.23	0.32	C20H33O4	6,7-dihydro-12-epi-LTB4	HC3,LC11
17.98	N/A	463.31	0.05	C27H44O6	Ecdysone	HC11,LC11,LC3
18.01	N/A	552.32	-1.52	C30H43N5O5	Ile-Lys-Phe-Phe	HC11,HC3,LC11
18.02	N/A	469.31	0.52	C22H42N6O5	Lys-Lys-Val-Pro	HC11,LC11,LC3
18.14	N/A	285.24	-0.82	C17H34O3	2-Methoxyhexadecanoic acid	HC3,LC11,LC3
18.14	N/A	311.22	-2.31	C18H32O4	[FA (18:2)] 9S-hydroperoxy-10E,12Z-octadecadienoic acid	HC3,LC11,LC3
18.15	N/A	435.15	2.25	C25H24O7	[Fv Hydroxy,dimethoxy,hydroxy,methoxy(9:1)] 5-Hydroxy-7,8-dimethoxy-6-(2-hydroxy-5-methoxybenzyl)flavanone	HC11,HC3,LC11,LC3
18.15	N/A	297.24	-1.65	C18H34O3	2-Oxooctadecanoic acid	HC11,HC3,LC11,LC3

18.16	N/A	265.15	-1.50	C12H26O4S	sodium dodecyl sulfate	HC11,HC3,LC11,LC3
18.18	N/A	284.19	-0.73	C15H27NO4	2-Octenoylcarnitine	HC11,HC3,LC11
18.18	N/A	377.27	0.01	C23H38O4	2-Arachidonoylglycerol	LC11,LC3
18.18	N/A	391.21	2.23	C22H32O6	Isodomedin	HC11,HC3,LC11,LC3
18.18	N/A	445.22	1.37	C21H30N6O5	Lys-Trp-Asn	HC11,HC3,LC11,LC3
18.19	N/A	121.03	0.56	C7H6O2	Benzoate	HC11,HC3,LC11,LC3
18.19	N/A	285.12	-2.58	C28H36N4O9	Asp-Leu-Tyr-Tyr	HC11,HC3,LC11,LC3
18.20	N/A	549.20	-2.79	C27H34O12	Eucommin A	HC11,HC3,LC11,LC3
18.21	N/A	138.02	-1.36	C6H5NO3	6-Hydroxynicotinate	HC11,HC3,LC11,LC3
18.21	N/A	255.20	-0.81	C15H28O3	[FA oxo(15:0)] 4-oxo-pentadecanoic acid	HC11,HC3,LC11,LC3
18.21	N/A	299.26	-1.11	C18H36O3	[FA hydroxy(18:0)] 2S-hydroxy-octadecanoic acid	HC11,HC3,LC11,LC3
18.22	N/A	105.00	0.13	C3H6O2S	S-Methylthioglycolate	HC11,HC3,LC11,LC3
18.23	N/A	374.25	-0.18	C23H35O4	9,-Carboxy-gama-chromanol	HC11,HC3,LC11,LC3
18.27	N/A	257.21	-1.13	C15H30O3	[FA hydroxy(15:0)] 2-hydroxy-pentadecanoic acid	HC11,HC3,LC11,LC3
18.29	N/A	119.04	-0.53	C5H4N4	Purine	HC11,HC3,LC11,LC3
18.29	N/A	367.24	-2.33	C42H73O8P	PA(17:1(9Z)/22:4(7Z,10Z,13Z,16Z))	HC11,HC3,LC11,LC3
18.40	N/A	621.35	-0.19	C32H46N8O5	NAc-FnorLRF-amide	HC3,LC3
18.40	N/A	505.32	-1.99	C29H46O7	2-deoxy-20-hydroxy-5alpha-ecdysone 3-acetate	HC11,HC3,LC11,LC3
18.51	N/A	356.28	-0.91	C20H39NO4	N-palmitoyl threonine	HC3,LC11,LC3
18.58	N/A	325.20	-2.77	C18H30O5	[FA trihydroxy(2:0)] 9S,11R,15S-trihydroxy-2,3-dinor-5Z,13E-prostadienoic acid-cyclo[8S,12R]	HC11,HC3,LC11,LC3
18.63	N/A	227.20	-1.38	C14H28O2	Tetradecanoic acid	HC11,HC3,LC11,LC3
18.66	N/A	345.16	1.75	C14H26N4O4S	Lys-Cys-Pro	HC11,LC11
18.72	N/A	412.31	-1.14	C23H43NO5	3-Hydroxy-9-hexadecenoylcarnitine	HC11,HC3,LC11
18.79	N/A	432.25	2.48	C50H75O10P	PG(22:6(4Z,7Z,10Z,13Z,16Z,19Z)/22:6(4Z,7Z,10Z,13Z,16Z,19Z))	HC11,HC3,LC11,LC3
18.90	N/A	489.19	-2.34	C18H30N6O10	Asn-Lys-Asp-Asp	HC11
18.90	N/A	399.23	2.63	C18H32N4O6	Ala-Leu-Thr-Pro	HC11,HC3,LC11,LC3
18.97	N/A	453.32	-1.33	C26H46O6	27-Norcholestanehexol	HC11,HC3,LC11
18.99	N/A	519.24	-0.75	C24H36N6O5S	Lys-Met-Trp-Gly	HC11,HC3,LC11,LC3
19.02	N/A	413.21	-1.20	C22H39BrO2	6-bromo-docosa-5E,9Z-dienoic acid	HC11,HC3,LC11
19.04	N/A	361.21	2.28	C20H30N2O4	Calpeptin	HC11,HC3,LC11,LC3

19.07	N/A	613.31	0.70	C31H51O10P	[PR] Dodecaprenyl phosphate-galacturonic acid	HC11,LC11,LC3
19.19	N/A	377.18	0.08	C18H26N4O5	Phe-Val-Asn	HC11,HC3,LC11,LC3
19.21	N/A	529.33	0.62	C35H46O4	[Fv] Neolinderatin	HC11,HC3,LC11,LC3
19.30	N/A	496.29	-1.44	C22H39N7O6	His-Leu-Lys-Thr	HC11,HC3,LC11,LC3
19.47	N/A	336.23	0.40	C20H33O4	6,7-dihydro-12-epi-LTB4	LC3
19.70	N/A	299.26	-1.49	C18H36O3	[FA hydroxy(18:0)] 2S-hydroxy-octadecanoic acid	HC11,HC3,LC11,LC3
19.80	N/A	779.49	1.02	C43H73O10P	PG(15:0/22:6(4Z,7Z,10Z,13Z,16Z,19Z))	HC11,HC3,LC11,LC3
19.87	N/A	392.26	-1.90	C19H40NO5P	C19 Sphingosine-1-phosphate	HC11,HC3,LC11,LC3
19.88	N/A	553.32	1.57	C26H51O10P	[PG (10:0)] 1,2-didecanoyl-sn-glycero-3-phospho-(1,-sn-glycerol)	HC11,HC3,LC11
19.99	N/A	253.22	-1.94	C16H30O2	(9Z)-Hexadecenoic acid	HC11,LC11
20.02	N/A	565.28	-0.50	C34H38N4O4	MesoporphyrinIX	LC11,LC3
20.07	N/A	693.44	0.24	C35H66O13	methyl 13-sophorosyloxydocosanoate	HC11,HC3,LC11,LC3
20.20	N/A	552.32	-1.86	C30H43N5O5	Ile-Lys-Phe-Phe	HC11,HC3,LC11
20.42	N/A	489.30	-1.50	C25H47O7P	PA(22:2(13Z,16Z)/0:0)	HC11,HC3,LC11
20.53	N/A	523.29	0.86	C29H40N4O5	Ile-Phe-Phe-Val	HC11,HC3,LC11,LC3
20.58	N/A	391.28	-2.56	C24H40O4	[ST hydrox] 3alpha,7alpha-Dihydroxy-5beta-cholan-24-oic Acid	HC11,HC3,LC11,LC3
20.81	N/A	255.23	-1.52	C16H32O2	Hexadecanoic acid	HC11,HC3,LC11,LC3
20.83	N/A	476.31	-1.31	C24H48NO6P	[PC (16:2)] 1-(9E,10E-hexadecadienyl)-sn-glycero-3-phosphocholine	HC11,LC11,LC3
20.87	N/A	440.34	-2.66	C25H47NO5	3-Hydroxy-11Z-octadecenoylcarnitine	HC11,LC11,LC3
20.91	N/A	367.24	-2.27	C42H73O8P	PA(17:1(9Z)/22:4(7Z,10Z,13Z,16Z))	HC11,HC3,LC11,LC3
21.03	N/A	581.35	-2.84	C58H104N2O21	Ganglioside GM3 (d18:1/9Z-18:1)	HC3,LC11
21.07	N/A	312.25	-1.66	C18H35NO3	[FA (16:0)] N-hexadecanoyl-glycine	HC3,LC3
21.10	N/A	895.57	-2.91	C49H85O12P	PI(O-18:0/22:6(4Z,7Z,10Z,13Z,16Z,19Z))	HC11,HC3,LC11,LC3
21.11	N/A	635.40	-0.52	C29H52N10O6	Argiotoxin 636	HC11,HC3,LC11,LC3
21.16	N/A	116.93	-0.30	CrH2O4	chromate	HC11,HC3,LC11,LC3
21.29	N/A	535.34	-2.82	C27H53O8P	[GP (12:0/12:0)] 1,2-didodecanoyl-sn-glycero-3-phosphate	HC3,LC11,LC3
21.44	N/A	687.40	-1.66	C39H61O8P	PA(18:4(6Z,9Z,12Z,15Z)/18:4(6Z,9Z,12Z,15Z))	HC11,HC3,LC11,LC3
21.50	N/A	445.22	1.14	C21H30N6O5	Lys-Trp-Asn	HC11,HC3,LC11,LC3

21.71	N/A	269.25	-1.78	C17H34O2	[FA (17:0)] heptadecanoic acid	HC11,HC3,LC11,LC3
21.90	N/A	528.32	-0.18	C28H43N5O5	Ile-Leu-Trp-Val	HC11,HC3,LC11
22.74	N/A	283.26	-1.24	C18H36O2	Octadecanoic acid	HC11,HC3,LC11,LC3
22.79	N/A	606.34	-1.66	C32H45N7O5	Lys-Lys-Phe-Trp	HC11,HC3,LC11,LC3
22.96	N/A	576.37	-0.51	C29H56NO8P	[PC (16:0)] 1-hexadecanoyl-2-(4E-valeryl)-sn-glycero-3-phosphocholine	HC11,HC3,LC11,LC3
23.06	N/A	392.29	2.54	C22H39N3O3	N-palmitoyl histidine	HC11,HC3,LC11,LC3
23.43	N/A	536.34	1.67	C26H52NO8P	[PC acetyl(16:0)] 2-acetyl-3-hexadecanoyl-sn-glycero-1-phosphocholine	HC11,HC3,LC11,LC3
25.20	N/A	623.35	2.94	C33H52O11	Spongipregnoloside A	HC11,LC11,LC3
25.59	N/A	255.20	-1.49	C15H28O3	[FA oxo(15:0)] 4-oxo-pentadecanoic acid	HC11,HC3,LC11,LC3
25.70	N/A	119.04	-0.41	C5H4N4	Purine	HC11,HC3,LC11,LC3
26.68	N/A	661.40	0.56	C37H58O10	cimicifoetiside A	HC11,HC3,LC11,LC3
30.32	N/A	119.04	-0.45	C5H4N4	Purine	HC11,HC3,LC11,LC3
30.65	N/A	445.22	0.90	C21H30N6O5	Lys-Trp-Asn	HC11,LC11
30.72	N/A	112.01	0.53	C3H3N3O2	Azomycin	HC11,HC3,LC11,LC3
30.74	N/A	943.71	1.15	C53H100O13	DAT(16:0/23:0(2Me[S],4Me[S]))	HC3,LC11
30.76	N/A	435.10	-1.73	C13H21N6O9P	(L-Seryl)adenylate	HC11,HC3,LC11,LC3
30.77	N/A	265.15	-0.87	C12H26O4S	sodium dodecyl sulfate	HC11,HC3,LC11,LC3
30.77	N/A	157.00	-1.92	C6H6O3S	benzenesulfonate	HC11,HC3
30.81	N/A	126.90	-1.09	HI	hydrogen iodide	HC11,HC3,LC11,LC3
30.82	N/A	382.13	2.29	C21H21NO6	(+)-Adlumine	HC11,HC3,LC11,LC3
30.83	N/A	396.15	1.33	C22H23NO6	Aureothin	HC11,HC3,LC11,LC3
30.92	N/A	367.24	2.30	C37H73N2O10P	[PS (17:0/14:0)] 1-heptadecanoyl-2-(9Z-tetradecenoyl)-sn-glycero-3-phosphoserine (Ammmonium salt)	HC11,HC3,LC11,LC3
30.92	N/A	183.00	-1.11	C6H4N2O5	2,4-Dinitrophenol	HC11,LC11
30.92	N/A	116.93	-0.15	CrH2O4	chromate	HC11,HC3,LC11
30.92	N/A	213.01	2.67	C5H14N2O2Se	selenalysine	HC11,HC3,LC11,LC3
30.93	N/A	111.02	0.62	C4H4N2O2	Uracil	HC3,LC11
30.93	N/A	138.02	-0.21	C6H5NO3	6-Hydroxynicotinate	HC11,HC3,LC11,LC3
30.93	N/A	121.03	0.14	C7H6O2	Benzoate	HC11,HC3,LC11

30.94	N/A	173.08	-0.45	C8H14O4	Suberic acid	HC11,HC3,LC3
30.94	N/A	651.29	1.00	C36H40N6O6	Trp-Trp-Val-Tyr	HC11
30.94	N/A	255.23	-1.39	C16H32O2	Hexadecanoic acid	HC11,HC3,LC3
30.94	N/A	547.34	1.62	C26H44N8O5	Arg-Lys-Phe-Val	HC11
30.94	N/A	409.22	-0.33	C22H34O7	Nigakihemiacetal A	HC3,LC3
30.94	N/A	159.07	-1.12	C7H12O4	[FA (7:0/2:0)] Heptanedioic acid	HC11,HC3,LC11,LC3
30.95	N/A	533.24	0.04	C24H34N6O8	Asp-Lys-Trp-Ser	HC11,LC11,LC3
30.97	N/A	267.07	-1.20	C9H16O9	2(alpha-D-Mannosyl)-D-glycerate	HC11,HC3,LC11,LC3
30.98	N/A	283.26	-0.11	C18H36O2	Octadecanoic acid	HC11,LC11,LC3
30.98	N/A	96.96	1.75	H2O4S	Sulfate	HC11,HC3,LC3
30.99	N/A	138.97	-0.60	C2H4O5S	Sulfoacetate	HC11,HC3,LC11,LC3
31.00	N/A	129.05	-1.61	C6H10O3	(S)-3-Methyl-2-oxopentanoic acid	HC11,HC3,LC11,LC3
31.03	N/A	128.03	-0.34	C5H7NO3	L-1-Pyrroline-3-hydroxy-5-carboxylate	HC11,HC3,LC11
31.03	N/A	715.54	-2.57	C51H72O2	menaquinone-8	HC3
31.06	N/A	498.30	-1.49	C22H41N7O6	Gln-Lys-Lys-Pro	HC11,LC3
31.07	N/A	527.33	0.28	C23H44N8O6	Arg-Leu-Leu-Gln	HC11,HC3,LC3
31.07	N/A	742.50	1.99	C40H74NO9P	PS(O-16:0/18:3(9Z,12Z,15Z))	HC11,HC3,LC11,LC3
31.12	N/A	112.94	-0.42	H2O3S2	H2S2O3	HC11,HC3,LC11,LC3
31.14	N/A	145.06	-1.28	C5H10N2O3	L-Glutamine	HC11,HC3,LC11,LC3
31.19	N/A	237.06	-1.25	C8H14O8	3-Deoxy-D-manno-octulosonate	HC11,HC3,LC11,LC3
31.19	N/A	131.08	-1.04	C5H12N2O2	L-Ornithine	HC3,LC11,LC3
31.19	N/A	146.05	-1.31	C5H9NO4	L-Glutamate	HC11,HC3,LC11,LC3
31.20	N/A	218.10	-1.32	C9H17NO5	Pantothenate	HC11,HC3,LC11
31.20	N/A	284.26	-0.73	C17H35NO2	[SP (17:0)] heptadecasphing-4-enine	HC11
31.24	N/A	171.01	-1.66	C3H9O6P	sn-Glycerol 3-phosphate	HC11,HC3,LC11
31.24	N/A	219.05	-2.68	C8H12O7	dihomocitrate	HC11,HC3,LC11,LC3
31.28	N/A	321.05	-1.42	C10H15N2O8P	dTMP	HC11,HC3,LC11,LC3
31.29	N/A	131.05	-0.48	C4H8N2O3	L-Asparagine	HC11,HC3,LC11,LC3
31.30	N/A	323.03	-0.96	C9H13N2O9P	UMP	HC11,HC3,LC11
31.30	N/A	132.03	-0.97	C4H7NO4	L-Aspartate	HC11,LC11,LC3
31.32	N/A	159.08	-1.66	C6H12N2O3	D-Alanyl-D-alanine	HC11,HC3,LC11,LC3

31.42	N/A	362.08	-1.00	C12H17N3O10	Asp-Asp-Asp	HC11,LC11,LC3
31.60	N/A	477.11	-1.38	C16H22N4O13	Asp-Asp-Asp-Asp	HC11,HC3,LC11
33.28	N/A	132.03	-0.81	C4H7NO4	L-Aspartate	HC11,HC3,LC11,LC3
33.32	N/A	131.05	-0.76	C4H8N2O3	L-Asparagine	HC11,HC3,LC11,LC3
33.50	N/A	154.06	-1.91	C6H9N3O2	L-Histidine	HC11,HC3,LC11,LC3

B						
Retention time (minutes)	Predicted RT (minutes)	m/z	mass error ppm	Formula	Putatively identified metabolite (Level 2[1])	Samples
Positive ion mode - ZIC-HILIC column						
3.58	2.15	145.1228	-0.29	C8H16O2	3R-Methylheptanoic acid	HC11,HC3,LC11,LC3
3.75	2.39	315.1235	0.81	C18H18O5	[Fv Hydroxy,methox] 2'-Hydroxy-3,4,5-methoxychalcone	HC11,HC3,LC11,LC3
4.52	3.08	363.1969	-0.76	C21H27FO4	17,21-Epoxy-9-fluoro-11beta-hydroxypregn-4-ene-3,20-dione	HC11,HC3
4.52	3.02	335.1656	-0.58	C19H23FO4	9-Fluoro-16alpha-hydroxyandrost-4-ene-3,11,17-trione	HC11,HC3,LC11
4.68	4.41	295.1332	-0.65	C19H18O3	(2-Butylbenzofuran-3-yl)(4-hydroxyphenyl)ketone	HC11,HC3,LC11,LC3
4.71	4.36	251.1439	1.36	C18H18O	(R)-4'-Deoxyindenestrol	HC11
4.90	4.31	343.1548	0.85	C20H22O5	Phaseollidin hydrate	HC11,HC3,LC11,LC3
5.05	4.19	149.0605	1.66	C9H8O2	trans-Cinnamate	HC11,HC3,LC11,LC3
5.13	4.95	391.2853	1.29	C24H38O4	[ST hydrox] 3alpha,12alpha-Dihydroxy-5beta-chol-6-en-24-oic Acid	HC11,HC3,LC11,LC3
5.17	5.33	419.3162	0.19	C26H42O4	[ST (3:0)] (5Z,7E)-(1S,3R,24R)-22-oxa-9,10-seco-5,7,10(19)-cholestatriene-1,3,24-triol	HC11,HC3,LC11,LC3
5.22	5.45	705.5828	0.97	C47H76O4	[ST (20:4)] cholest-5-en-3beta-yl (15S-hydroperoxy-5Z,8Z,12E,14Z-eicosatetraenoate)	HC11,HC3,LC11,LC3
5.47	5.47	205.0607	0.19	C19H20O10	Khellol glucoside	HC11,LC11,LC3
5.52	5.52	500.2263	1.20	C23H29N7O6	N-Acetyl-O-demethylpuromycin	LC3
5.53	5.56	901.5014	2.02	C46H78O13P2	PGP(18:3(9Z,12Z,15Z)/22:4(7Z,10Z,13Z,16Z))	HC11
5.56	4.11	886.4902	1.48	C55H66MgN4O5	geranylgeranyl-chlorophyll a	HC11
5.57	5.15	419.2556	-1.61	C21H39O6P	CPA(18:1(11Z)/0:0)	HC11
5.58	5.65	627.3731	-2.15	C33H54O11	Cotylenin F	HC11
5.58	5.16	842.5714	1.73	C49H80NO8P	PE(44:9)	HC11

5.58	5.66	899.6011	-0.23	C49H87O12P	PI(O-20:0/20:5(5Z,8Z,11Z,14Z,17Z))	HC11
5.58	5.66	945.6435	0.35	C51H93O13P	PI(42:3)	HC11
5.58	5.66	925.6150	-2.10	C51H89O12P	PI(O-20:0/22:6(4Z,7Z,10Z,13Z,16Z,19Z))	HC11
5.58	6.13	217.1800	-1.41	C12H24O3	12-Hydroxydodecanoic acid	HC11
5.60	5.69	455.3375	0.53	C26H46O6	27-Norcholestanehexol	HC11
5.60	5.70	427.2110	-2.50	C25H30O6	[Fv hydroxy(5:0/2:0)] 2',4',6',3,4-Pentahydroxy- 3',5-diprenyldihydrochalcone	HC11
5.60	5.70	895.5690	-1.11	C49H83O12P	PI(P-18:0/22:6(4Z,7Z,10Z,13Z,16Z,19Z))	HC11
5.60	6.20	997.7262	-2.33	C67H96O6	TG(64:17)	HC11
5.61	5.71	897.5855	-0.21	C49H85O12P	PI(O-18:0/22:6(4Z,7Z,10Z,13Z,16Z,19Z))	HC11
5.61	5.71	973.6739	-0.58	C53H97O13P	PI(44:3)	HC11
5.61	6.47	395.2213	-2.22	C25H30O4	Bixin	HC11
5.61	5.33	167.0345	0.67	C8H6O4	Phthalate	HC11,HC3,LC11,LC3
5.62	5.23	794.4336	1.19	C41H63NO14	Protoveratrine A	HC11
5.62	6.70	353.3413	-1.87	C23H44O2	[FA (23:1)] 22-tricosenoic acid	HC11,HC3,LC11,LC3
5.62	4.24	444.3679	-2.16	C25H49NO5	12-Hydroxy-12-octadecanoylcarnitine	LC11
5.62	6.24	437.3990	-1.04	C28H52O3	[FA oxo(28:0)] 19-oxo-22Z-octacosenoic acid	HC3,LC11,LC3
5.62	6.24	425.2901	-0.47	C24H40O6	[ST hydroxy(4:0)] 1beta,3alpha,7alpha,12alpha-Tetrahydroxy-5beta-cholan-24-oic Acid	HC11,HC3,LC11,LC3
5.62	3.74	456.4042	-2.43	C27H53NO4	Arachidylcarnitine	HC3,LC11,LC3
5.62	6.24	529.2916	-2.70	C27H45O8P	2-deoxyecdysone 22-phosphate	HC11

5.63	6.29	387.2165	-1.55	C23H30O5	[FA oxo,hydroxy(2:0)] 9-oxo-11R,15S-dihydroxy-17-phenyl-18,19,20-trinor-5Z,13E-prostadienoic acid	HC11
5.63	6.65	299.2580	-1.92	C18H34O3	2-Oxooctadecanoic acid	HC11,HC3,LC11,LC3
5.63	5.63	427.3034	0.45	C20H38N6O4	Leupeptin	HC11,HC3,LC3
5.63	6.66	371.3164	0.88	C22H42O4	2-monooleoylglycerol	HC11,HC3,LC11,LC3
5.63	6.26	453.3568	-2.69	C27H48O5	[ST (5:0)] 5beta-Cholestane-3alpha,7alpha,12alpha,25,26-pentol	HC3,LC11,LC3
5.64	4.27	416.3372	-0.95	C23H45NO5	3-Hydroxyhexadecanoylcarnitine	LC11
5.64	6.27	409.3681	-0.14	C26H48O3	[FA oxo(26:0)] 17-oxo-20Z-hexacosenoic acid	HC3,LC11,LC3
5.64	6.64	325.2737	-1.80	C20H36O3	[FA hydroxy(20:2)] 11R-hydroxy-12E,14Z-eicosadienoic acid	HC11,HC3,LC11,LC3
5.64	6.75	395.3883	-1.57	C26H50O2	[FA (26:0)] 9-hexacosenoic acid	HC3,LC11,LC3
5.64	6.29	437.3620	-2.39	C27H48O4	[ST (4:0)] 5beta-Cholestane-3alpha,7alpha,12alpha,26-tetrol	HC11,HC3,LC11
5.64	6.71	311.2953	1.16	C20H38O2	[FA (20:0)] 11Z-eicosenoic acid	HC11,HC3,LC11,LC3
5.65	6.14	337.1654	1.02	C18H24O6	omega-Carboxy-trinor-leukotriene B4	HC11
5.65	4.29	706.3999	-2.13	C34H59NO14	[SP] Fumonisin B2	HC11
5.65	5.80	859.6409	-2.17	C48H91O10P	PG(42:2)	HC11
5.65	6.35	267.1729	1.51	C12H27O4P	Tributyl phosphate	HC11,HC3,LC11,LC3
5.65	5.65	351.3265	0.74	C23H42O2	20:2(5Z,9Z)(11Me,15Me,19Me)	HC11,HC3,LC11,LC3
5.65	6.31	463.3778	-1.99	C29H50O4	[ST hydroxy(3:2/3:0/3:0)] (5Z,7E)-(1R,2R,3R)-3-(hydroxy-propyl)-19-nor-9,10-seco-5,7-cholestadien-1,3,25-triol	HC3,LC11
5.66	5.66	917.6823	0.60	C42H44N4O16Fe	siroheme	HC11
5.66	6.31	379.3222	2.51	C24H42O3	[ST (3:0/3:0)] (7E)-(1R,3R)-9,11,21-trinor-9,10-seco-5,7-cholestadien-1,3,25-triol	HC11,HC3,LC11,LC3
5.66	6.37	400.3430	0.95	C23H45NO4	[FA] O-Palmitoyl-R-carnitine	HC11,HC3,LC11

5.66	6.73	309.2792	-0.43	C20H36O2	Icosadienoic acid	HC11,HC3,LC11,LC3
5.66	5.82	743.5578	-1.75	C42H79O8P	PA(17:0/22:2(13Z,16Z))	HC11
5.66	5.14	223.0646	2.48	C8H14O5S	2-(3'-methylthio)propylmalate	HC3,LC11,LC3
5.66	6.67	282.2798	0.59	C18H35NO	[FA (18:1)] 9Z-octadecenamide	HC11,HC3,LC11,LC3
5.67	6.78	341.3049	-1.80	C21H40O3	[FA oxo(21:0)] 2-oxo-heneicosanoic acid	HC3,LC11,LC3
5.67	5.81	388.3058	-1.23	C21H41NO5	2-Hydroxymyristoylcarnitine	HC3,LC11,LC3
5.67	5.35	470.2521	0.45	C20H40NO9P	PS(14:0/0:0)	HC11
5.68	6.33	327.0791	1.40	C18H15O4P	Triphenyl phosphate	HC11,LC11
5.68	6.81	398.3628	-1.39	C24H47NO3	Behenoylglycine	HC3,LC11
5.68	6.75	341.2687	-1.43	C20H36O4	[FA (20:2)] 15S-hydroperoxy-11Z,13E-eicosadienoic acid	HC11,HC3,LC11,LC3
5.68	6.79	374.3414	-2.26	C25H43NO	[FA (5:2/20:4)] N-pentyl-5Z,8Z,11Z,14Z-eicosatetraenoyl amine	HC11,HC3
5.68	6.66	269.2481	0.32	C17H32O2	omega-Cyclohexylundecanoic acid	HC11,HC3,LC11,LC3
5.68	6.76	327.2896	-0.89	C20H38O3	2-Oxophytanate	HC3,LC11,LC3
5.68	6.72	281.2484	1.14	C18H32O2	Linoleate	HC11,HC3,LC11,LC3
5.68	6.59	257.2119	1.08	C15H28O3	[FA oxo(15:0)] 4-oxo-pentadecanoic acid	HC3,LC11,LC3
5.69	6.82	325.3097	-2.76	C21H40O2	[FA (3:2/18:0)] 2-propyl-9Z-octadecenoic Acid	HC3,LC11,LC3
5.69	6.38	358.2963	1.67	C20H39NO4	N-palmitoyl threonine	HC3,LC11,LC3
5.69	5.52	278.2488	1.56	C18H31NO	[SP (4:0)] 1-deoxy-sphinga-6Z,9Z,12Z,15Z-tetraenine	HC11,LC11
5.69	6.75	305.2487	2.20	C20H32O2	[FA (20:4)] 5Z,8Z,11Z,14Z-eicosatetraenoic acid	HC11,HC3,LC11
5.69	5.89	411.3831	-1.66	C26H50O3	tween 40	LC11
5.69	6.49	279.1597	0.27	C16H22O4	2-Ethylhexyl phthalate	HC11,HC3,LC11,LC3
5.69	6.80	360.3258	-2.13	C24H41NO	[FA (4:2/20:4)] N-butyl-5Z,8Z,11Z,14Z-eicosatetraenoyl amine	HC11,HC3

5.70	5.89	419.2791	-1.45	C25H38O5	3alpha,12alpha-Dihydroxy-5beta-pregnan-20-one diacetate	LC11
5.70	5.58	370.3312	-2.33	C22H43NO3	[SP amino,tetramethyl(4:0/18:0/3:0)] 2S-amino-5,9,13,17-tetramethyl-8E,16-octadecadiene-1,3R,14-triol	HC3,LC11,LC3
5.70	6.67	253.2161	-2.48	C16H28O2	[FA (16:2)] 9,12-hexadecadienoic acid	HC11,HC3,LC11,LC3
5.70	6.40	409.2584	-1.46	C23H36O6	[FA methyl,oxo,hydroxy(2:0)] methyl 9-oxo-11R-hydroxy-15R-acetoxy-5Z,13E-prostadienoate	HC3,LC11,LC3
5.70	6.69	297.2423	-2.23	C18H32O3	[FA hydroxy(18:2)] 9S-hydroxy-10E,12Z-octadecadienoic acid	HC3,LC11
5.70	5.99	304.1696	-1.57	C21H21NO	alpha-Ethyl-alpha,beta-diphenyl-2-pyridineethanol	HC11
5.71	6.03	223.0971	0.24	C12H14O4	[FA (12:4/2:0)] 2E,4E,8E,10E-Dodecatetraenedioic acid	HC11,LC11
5.71	5.92	414.2780	2.40	C26H37O4	11'-Carboxy-alpha-tocotrienol	LC11,LC3
5.71	5.92	313.1448	2.83	C19H20O4	[Fv] Desmosdumotin C	HC11,HC3,LC11,LC3
5.71	6.42	449.3096	1.56	C27H44O3S	[ST (2:0/2:0)] (7E)-(3S,6R)-6,19-epithio-9,10-seco-5(10),7-cholestadien-3-ol S,S-dioxide	HC11,LC11
5.71	6.79	332.2949	-1.18	C22H37NO	[FA ethyl(20:4)] N-ethyl-5Z,8Z,11Z,14Z-eicosatetraenoyl amine	HC11
5.71	5.92	683.4650	-0.29	C38H67O8P	PA(13:0/22:4(7Z,10Z,13Z,16Z))	HC11
5.71	6.84	323.2959	2.91	C21H38O2	[FA methyl(20:2)] 19-methyl-5E,9E-eicosadienoic acid	HC3,LC11,LC3
5.71	5.92	433.2585	-1.12	C25H36O6	Glycinoeclepin A	HC11,HC3,LC11,LC3
5.71	6.74	271.2268	-1.86	C16H30O3	[FA oxo(16:0)] 3-oxo-hexadecanoic acid	HC3,LC11,LC3
5.71	5.43	419.2446	-0.30	C25H30N4O2	Naphthyl dipeptide	HC11,HC3,LC11,LC3
5.71	6.11	298.3110	0.05	C19H39NO	Tridemorph	HC11,HC3,LC11,LC3
5.71	6.80	284.2953	0.01	C18H37NO	Octadecanamide	HC11,HC3,LC11,LC3
5.71	6.68	259.2277	1.53	C15H30O3	[FA hydroxy(15:0)] 2-hydroxy-pentadecanoic acid	HC3,LC11,LC3
5.71	5.93	434.2646	-2.05	C23H35N3O5	N-Formyl-norleucyl-leucyl-phenylalanyl-methylester	HC11,HC3,LC3

5.72	6.49	375.2900	0.28	C24H38O3	3-Oxo-5beta-cholanate	HC3,LC11
5.72	5.94	149.0240	0.98	C16H8O6	Medicagol	HC11,HC3,LC11,LC3
5.72	6.29	355.2458	2.68	C21H32F2O2	3,3-Difluoro-5alpha-androstan-17beta-yl acetate	HC11,LC11
5.72	6.73	397.2583	-1.79	C22H36O6	[FA methyl(5:1/5:2/8:0)] methyl 8-[3,5-epidioxy-2-(3-hydroperoxy-1-pentenyl)-cyclopentyl]-octanoate	HC11,HC3,LC3
5.72	6.75	256.2643	1.06	C16H33NO	Palmiticamide	HC11,HC3,LC11,LC3
5.72	6.65	243.1961	0.61	C14H26O3	[FA oxo(14:0)] 3-oxo-tetradecanoic acid	HC3,LC11,LC3
5.72	5.38	381.2630	-2.95	C22H36O5	[ST trihydrox] 24-dinor-3alpha,7alpha,12alpha-trihydroxy-5beta-cholan-22-oic acid	HC11,HC3,LC11,LC3
5.73	6.81	328.2851	-0.19	C19H37NO3	Margaroylglycine	HC3,LC11,LC3
5.73	6.46	275.1865	2.52	C14H26O5	3-Hydroxytetradecanedioicacid	HC11
5.73	6.28	339.2690	0.57	C24H34O	3-(2,4-Cyclopentadien-1-ylidene)-5alpha-androstan-17beta-ol	HC11
5.73	6.68	361.2383	1.30	C22H32O4	[FA hydroxy(22:0/6:0)] 10R,17S-dihydroxy-docosa-4Z,7Z,11E,13E,15Z,19Z-hexaenoic acid	HC11,HC3,LC11,LC3
5.74	5.97	290.2343	-2.91	C35H62O6	Giganin	HC3,LC11,LC3
5.74	6.76	299.2220	-0.67	C17H30O4	[FA (17:1/2:0)] 8E-Heptadecenedioic acid	LC11
5.74	6.47	127.1488	1.35	C9H18	1-Nonene	HC11,HC3,LC11,LC3
5.74	6.13	370.2967	2.73	C21H39NO4	cis-5-Tetradecenoylcarnitine	HC11,HC3,LC11,LC3
5.74	5.98	569.3815	-0.58	C28H57O9P	PG(22:0/0:0)	HC11,HC3,LC11,LC3
5.74	6.88	346.3102	-2.22	C23H39NO	[FA (3:2/20:4)] N-propyl-5Z,8Z,11Z,14Z-eicosatetraenoyl amine	HC11,HC3
5.75	5.35	359.1858	-0.06	C21H26O5	Prednisone	HC11,HC3,LC11
5.75	6.71	227.2008	-1.14	C14H26O2	(9Z)-Tetradecenoic acid	HC11,HC3,LC11,LC3
5.75	5.06	255.1956	-1.74	C15H26O3	3-hydroxy-15-dihydrolubimin	HC11,HC3,LC11,LC3
5.75	6.70	228.2329	0.64	C14H29NO	myristic amide	HC11,HC3,LC11,LC3

5.75	6.73	295.2271	-0.79	C18H30O3	[FA oxo(5:1/5:0/8:0)] (1S,2S)-3-oxo-2-(2'Z-pentenyl)-cyclopentaneoctanoic acid	HC11,HC3,LC11,LC3
5.75	6.79	265.2167	-0.22	C17H28O2	[FA (17:3)] 8Z,11Z,14Z-heptadecatrienoic acid	HC11,HC3,LC11,LC3
5.75	6.01	627.3492	-2.67	C29H55O12P	PI(20:1(11Z)/0:0)	HC3
5.76	6.77	399.2507	-1.22	C18H39O7P	Tris(butoxyethyl)phosphate	HC3,LC11,LC3
5.76	6.69	335.2213	-2.72	C20H30O4	Prostaglandin A2	HC11,HC3,LC11,LC3
5.76	6.76	359.2226	1.12	C22H30O4	Cannabidiolic acid	HC11,HC3,LC11,LC3
5.76	6.81	319.2267	-1.85	C20H30O3	Leukotriene A4	HC3,LC11,LC3
5.76	6.68	213.1852	-1.32	C13H24O2	[FA methyl(12:1)] 2-methyl-2-dodecenoic acid	HC11,HC3,LC11,LC3
5.76	6.07	205.0859	-2.69	C12H12O3	3-Butylidene-7-hydroxyphthalide	HC11,HC3,LC11,LC3
5.76	6.77	241.2166	-0.52	C15H28O2	[FA dimethyl(13:0)] 2,5-dimethyl-2E-tridecenoic acid	HC11,HC3,LC11,LC3
5.77	6.71	349.2164	-0.95	C24H28O2	[PR] Bixindial/ Bixin aldehyde	HC11
5.77	5.77	632.3073	0.39	C33H45NO11	Mesaconitine	HC3
5.77	6.20	271.1910	0.50	C15H26O4	(10S)-Juvenile hormone III acid diol	HC3,LC11,LC3
5.77	6.54	280.2638	-0.63	C18H33NO	Linoleamide	HC11,HC3,LC11,LC3
5.78	5.29	311.1865	2.03	C17H26O5	Botrydial	HC3,LC11
5.78	6.37	285.2069	1.04	C16H28O4	(10S)-Juvenile hormone III diol	LC3
5.78	6.14	259.1334	0.17	C16H18O3	Heritonin	HC11,HC3,LC11,LC3
5.78	6.57	468.3106	-1.71	C29H41NO4	[FA (20:4)] N-(5Z,8Z,11Z,14Z-eicosatetraenoyl)-tyrosine	HC11
5.78	5.57	894.6224	0.05	C50H88NO10P	PS(44:5)	HC11
5.79	6.60	295.1904	-1.61	C17H26O4	[6]-Gingerol	HC3,LC11,LC3

5.79	6.26	339.1814	1.93	C18H26O6	Pinolidoxin	HC11,HC3,LC11,LC3
5.79	5.79	646.3219	-1.33	C34H47NO11	Aconitine	HC3
5.79	6.58	214.2170	-0.22	C13H27NO	Tridecanamide	HC11,HC3,LC11,LC3
5.79	6.09	655.4721	2.82	C37H67O7P	PA(O-16:0/18:4(6Z,9Z,12Z,15Z))	HC11
5.79	5.70	289.1811	2.71	C18H24O3	Estriol	HC3,LC11,LC3
5.80	5.80	779.3534	-1.29	C30H54N10O10S2	Bis(glutathionyl)spermine disulfide	HC3,LC11,LC3
5.80	6.56	299.1279	-1.41	C18H18O4	[Fv] Aurentiacin	HC3,LC11,LC3
5.80	6.09	492.2769	-2.96	C27H41NO5S	Epothilone D	HC11,HC3,LC11,LC3
5.80	5.96	321.2073	2.27	C19H28O4	Testolate	LC11,LC3
5.80	6.42	220.11	-1.94	C16H13N	N-Phenyl-1-naphthylamine	HC11,HC3,LC11,LC3
5.80	6.80	273.21	0.60	C15H28O4	[FA methyl(14:0/2:0)] 3-methyl-tetradecanedioic acid	HC11,HC3,LC11,LC3
5.81	6.67	215.16	-1.25	C12H22O3	3-Oxododecanoic acid	HC11
5.81	6.14	277.14	-1.84	C16H20O4	Scorpioidin	HC11,HC3,LC11,LC3
5.81	6.06	137.13	-2.21	C10H16	[PR] (-)-Limonene	HC11,HC3,LC11,LC3
5.81	4.71	253.18	-2.24	C15H24O3	[PR] (+)-2-Sterpurene-6,12,15-triol	HC11,HC3,LC11,LC3
5.81	6.63	177.16	-2.74	C13H20	3,5,7-Trimethyl-2E,4E,6E,8E-decatetraene	HC11,HC3,LC11,LC3
5.81	6.44	357.13	-1.99	C20H20O6	Kievitone	HC3,LC11,LC3
5.81	6.46	181.16	-1.95	C12H20O	[FA (12:3)] 3,6,8-dodecatrien-1-ol	HC11,HC3,LC11,LC3
5.82	6.26	339.21	2.55	C20H28F2O2	4,4-Difluoro-17beta-hydroxy-17alpha-methyl-androst-5-en-3-one	HC3,LC11,LC3

5.83	4.15	764.56	-0.68	C44H78NO7P	PC(18:4(6Z,9Z,12Z,15Z)/P-18:1(11Z))	HC11
5.83	4.15	810.60	1.43	C46H84NO8P	PC(38:4)	HC11
5.83	5.83	435.24	2.59	C17H34N6O5S	Glutathionylspermidine	LC11,LC3
5.84	5.46	319.19	-0.52	C19H26O4	6beta-Hydroxy-D-homo-17a-oxaandrost-4-ene-3,17-dione	HC11,HC3,LC11,LC3
5.84	6.18	781.58	-2.67	C45H80O10	1-18:1-2-18:2-monogalactosyldiacylglycerol	HC11,LC11
5.84	5.32	135.08	-1.18	C9H10O	Indan-1-ol	HC11,HC3,LC11,LC3
5.84	6.19	837.56	-2.00	C47H81O10P	PG(19:0/22:6(4Z,7Z,10Z,13Z,16Z,19Z))	HC11
5.85	6.02	183.17	-0.61	C12H22O	Geosmin	HC11,HC3,LC11,LC3
5.85	6.20	501.32	-0.37	C30H44O6	11-Deoxocucurbitacin I	HC11,HC3
5.85	6.25	229.09	2.12	C14H12O3	3,4',5-Trihydroxystilbene	HC11,HC3,LC11,LC3
5.85	5.98	221.12	-0.89	C13H16O3	Precocene 2	HC11,HC3,LC11,LC3
5.85	6.70	341.20	-2.25	C18H28O6	11-dehydro-2,3-dinor-TXB2	HC11,LC11,LC3
5.85	5.46	255.12	-1.82	C13H18O5	[FA methyl,hydroxy,oxo(5:2/4:0)] methyl 4-[2-(2-formyl-vinyl)-3-hydroxy-5-oxo-cyclopentyl]-butanoate	HC11,HC3,LC11,LC3
5.85	6.84	201.19	-2.09	C12H24O2	Dodecanoic acid	HC11,HC3,LC11,LC3
5.86	6.21	393.24	-1.56	C19H37O6P	[GP (16:0)] 1-hexadecanoyl-sn-glycero-2,3-cyclic-phosphate	HC11
5.86	6.22	613.34	0.52	C28H53O12P	PI(19:1(9Z)/0:0)	HC3,LC3
5.86	5.17	393.23	2.23	C22H32O6	Isodomedin	HC11,HC3,LC11,LC3
5.86	6.70	217.12	-1.06	C14H16O2	[FA (14:0/2:0)] 10E,12E-tetradecadiene-4,6-diynoic acid	HC11,HC3,LC11,LC3
5.86	6.99	344.28	1.31	C19H37NO4	1,2-dioctanoyl-1-amino-2,3-propanediol	HC3,LC11,LC3
5.88	6.17	243.10	2.74	C15H14O3	Equol	HC11,HC3,LC11,LC3

5.88	5.75	293.17	-1.18	C17H24O4	Trichodermin	HC11,HC3,LC11,LC3
5.88	5.67	297.17	-0.79	C16H24O5	Graphinone	HC11,HC3,LC11,LC3
5.88	5.77	717.52	-1.62	C43H73O6P	2,3-Bis-O-(geranylgeranyl)glycerol 1-phosphate	HC11,HC3,LC3
5.90	3.67	256.12	1.32	C12H17NO5	N-D-Glucosylarylamine	HC11,HC3,LC11,LC3
5.90	6.30	921.68	-1.32	C50H97O12P	PI(P-20:0/21:0)	HC11
5.90	6.16	119.09	0.39	C9H10	alpha-Methylstyrene	HC11,HC3,LC11,LC3
5.91	6.31	459.24	-0.87	C26H34O7	Fumagillin	HC11,HC3,LC11,LC3
5.91	4.27	277.11	-2.42	C15H16O5	Lactucin	HC11,HC3,LC11,LC3
5.91	5.82	413.26	2.08	C23H32N4O3	2,4-diamino-6-ethyl-5,3'-(2-cyclohexylphenoxy)prop-1'-yloxypyrimidine	HC3
5.91	6.87	282.13	1.98	C21H15N	2,3,3-Triphenylacrylonitrile	HC11
5.92	6.59	261.11	-2.45	C15H16O4	[PR] Hemigossypol	HC11,HC3,LC11,LC3
5.92	6.84	241.11	-0.67	C12H16O5	3-carboxy-4-methyl-5-propyl-2-furanpropanoic acid	HC3,LC11,LC3
5.92	5.84	774.57	0.50	C42H80NO9P	PS(O-16:0/20:2(11Z,14Z))	HC11,HC3,LC11,LC3
5.92	5.84	716.52	-2.76	C39H74NO8P	PE(34:2)	HC11,HC3,LC11,LC3
5.92	6.35	677.49	1.77	C47H64O3	chlorobiumquinone	HC11,LC11
5.93	6.58	294.14	1.67	C15H20ClN3O	paclobutrazol	HC11,HC3,LC11,LC3
5.93	6.66	275.13	-2.83	C16H18O4	4,4'-Dihydroxy-3,5-dimethoxydihydrostilbene	HC11,HC3,LC11,LC3
5.94	6.37	457.22	-1.37	C26H32O7	[Fv] Antiarone J	HC3,LC11,LC3
5.94	5.76	219.07	-2.59	C12H10O4	4-Methylumbelliferyl acetate	HC3,LC11,LC3
5.94	5.65	123.08	-0.42	C8H10O	Phenylethyl alcohol	HC11,HC3,LC11,LC3

5.94	6.41	179.11	-2.35	C11H14O2	Eugenol methyl ether	HC11,HC3,LC11,LC3
5.95	5.91	626.35	-1.01	C32H51NO11	Glycochenodeoxycholicacid3-glucuronide	HC11,HC3
5.96	6.43	549.41	-1.77	C33H56O6	Cholesteryl-beta-D-glucoside	HC11,LC11,LC3
5.97	6.44	547.35	-2.92	C28H50O10	3-O-Mycarosylerythronolide B	HC11
5.97	5.30	111.04	-0.81	C6H6O2	p-Benzenediol	HC11,HC3,LC11,LC3
5.97	6.38	327.14	1.44	C16H22O7	[FA hydroxy,dioxo(4:0/2:0)] 11R-hydroxy-9,15-dioxo-2,3,4,5-tetranor-prostan-1,20-dioic acid	HC3,LC11,LC3
5.98	5.72	247.10	-2.62	C14H14O4	Columbianetin	HC11,HC3,LC11,LC3
5.98	6.81	217.14	-1.45	C11H20O4	[FA (11:0/2:0)] Undecanedioic acid	HC11
5.98	3.30	297.13	-1.39	C15H20O6	[PR] Vomitoxin	HC11,HC3,LC11,LC3
5.99	6.97	271.19	2.26	C37H48O3	[ST (3:0/3:0)] (5Z,7E)-(1S,3R)-25,25-diphenyl-26,27-dinor-9,10-seco-5,7,10(19)-cholestatriene-1,3,25-triol	HC3,LC11,LC3
5.99	6.75	266.12	2.11	C17H15NO2	2,3-Bis(p-methoxyphenyl)acrylonitrile	HC11
5.99	6.48	709.52	2.43	C41H73O7P	PA(O-18:0/20:5(5Z,8Z,11Z,14Z,17Z))	HC11
5.99	5.49	101.06	1.74	C5H8O2	Tiglic acid	HC11,HC3,LC11,LC3
5.99	5.79	181.09	-2.37	C10H12O3	Coniferyl alcohol	HC11,HC3,LC11,LC3
5.99	6.02	191.07	-1.70	C11H10O3	7-Ethoxycoumarin	HC11,HC3,LC11,LC3
5.99	6.47	224.11	2.84	C15H13NO	2-Acetamidofluorene	HC11,HC3,LC11,LC3
6.00	6.00	920.33	0.59	C36H54N7O19P	5-methyl-tetrahydrosarcinapterin	HC11,HC3
6.02	5.97	129.09	-1.22	C7H12O2	3-Isopropylbut-3-enoic acid	HC11,HC3,LC11,LC3
6.02	6.36	125.10	-2.32	C8H12O	[FA (8:2)] 2,4-octadienal	HC11,HC3,LC11,LC3

6.02	7.05	561.38	-0.01	C33H52O7	[ST (3:0)] (5Z,7E)-(3S)-9,10-seco-5,7,10(19)-cholestatrien-3-glucosiduronate	HC11,HC3,LC11,LC3
6.03	6.08	111.08	1.07	C7H10O	[FA (7:2)] 2,4-heptadienal	HC11,HC3,LC11,LC3
6.03	6.54	288.29	-2.97	C17H37NO2	[SP (17:0)] heptadecasphinganine	HC11,HC3,LC11,LC3
6.04	6.41	123.04	0.61	C7H6O2	Benzoate	HC11,HC3,LC11,LC3
6.04	6.04	745.21	1.93	C28H37N6O16P	demethylated 7,8-dihydromethanopterin	HC11,LC3
6.04	7.07	371.24	0.54	C20H34O6	Thromboxane B2	HC11
6.05	6.55	232.13	0.72	C14H17NO2	Leiokinine A	HC11
6.05	7.26	391.18	0.43	C21H27N2O3Cl	Compound II(R/S)	HC3,LC11,LC3
6.05	6.37	305.10	0.44	C16H16O6	[Fv hydroxy,methoxy(4:0)] 3,4,4',alpha-Tetrahydroxy-2'-methoxydihydrochalcone	HC11,HC3,LC11,LC3
6.06	7.11	223.12	0.44	C25H32O7	[FA methyl,oxo(5:0/5:0)] methyl 4R,12S-diacetoxy-9-oxo-5Z,7E,10Z,13Z,17Z-prostapentaenoate-cyclo[8,12]	HC11,HC3,LC11,LC3
6.06	5.62	787.24	-1.78	C39H38N4O14	HydroxypropionicporphyrinIII	HC11,HC3,LC11,LC3
6.07	6.51	347.19	-1.69	C20H26O5	Gibberellin A24	HC11,HC3,LC11,LC3
6.07	6.15	167.07	-1.60	C9H10O3	3-(3-Hydroxy-phenyl)-propanoic acid	HC11,HC3,LC11,LC3
6.08	6.51	240.10	0.18	C15H13NO2	N-Hydroxy-2-acetamidofluorene	HC11
6.08	4.66	782.57	-2.95	C44H80NO8P	PC(36:4)	HC11
6.08	4.66	227.09	-2.21	C11H14O5	Genipin	HC3,LC11,LC3
6.10	6.69	715.53	-1.62	C40H75O8P	[PG (14:0/8:0)] 1-tetradecanyl-2-(8-[3]-ladderane-octanyl)-sn-glycero-3-phospho-(1'-sn-glycerol)	HC11
6.10	6.42	293.20	1.37	C14H28O6	octyl α-D-galactopyranoside	HC11
6.10	6.71	415.18	-1.11	C23H26O7	[Fv Trihydroxy,methoxy,hydroxy,ethyl(9:1)] 5,7,4'-Trihydroxy-3'-methoxy-6-(beta-hydroxyethyl)-8-prenylflavanone	HC3,LC11,LC3
6.11	6.72	360.24	2.66	C41H67O8P	PA(38:7)	HC3,LC11,LC3

6.11	7.25	383.22	-0.56	C17H35O7P	[GP (14:0)] 1-tetradecanoyl-2-sn-glycero-3-phosphate	LC11,LC3
6.11	5.09	87.04	2.26	C4H6O2	Diacetyl	HC11,HC3,LC11,LC3
6.12	6.25	778.56	0.82	C41H80NO10P	PS(13:0/22:0)	HC11
6.13	4.12	220.17	-1.38	C14H21NO	Fabianine	HC11,HC3,LC11,LC3
6.13	6.77	677.51	-2.33	C37H73O8P	PA(34:0)	HC11,HC3,LC11,LC3
6.15	4.80	834.60	-1.84	C48H84NO8P	PC(40:6)	HC11
6.15	6.04	113.06	-0.07	C6H8O2	sorbate	HC11,HC3,LC11,LC3
6.16	6.11	101.10	-0.59	C6H12O	[FA (6:1)] 3Z-hexenol	HC11,HC3,LC11,LC3
6.17	6.17	914.16	-0.20	C30H42N7O18P3S	p-Coumaroyl-CoA	HC11
6.18	5.71	134.07	-2.97	C7H7N3	2-Aminobenzimidazole	HC11,HC3,LC11,LC3
6.18	6.26	169.09	-1.64	C9H12O3	1,3,5-trimethoxybenzene	HC11,HC3,LC11,LC3
6.18	6.86	439.28	-2.66	C21H43O7P	[GP (18:0)] 1-octadecanoyl-2-sn-glycero-3-phosphate	HC3,LC11,LC3
6.19	3.72	230.12	0.74	C14H15NO2	thyronamine	HC11,HC3,LC3
6.20	7.22	199.17	-0.43	C12H22O2	[PR] Citronellyl acetate	HC11,HC3,LC11,LC3
6.20	6.95	297.18	-2.93	C20H24O2	Ethynyl estradiol	LC11,LC3
6.21	4.53	188.16	-1.98	C10H21NO2	[FA amino(10:0)] 10-amino-decanoic acid	HC11,HC3,LC11,LC3
6.21	4.26	274.20	-0.62	C14H27NO4	Heptanoylcarnitine	HC3,LC11,LC3
6.21	6.18	196.08	0.65	C13H9NO	4-(2-Benzofuranyl)pyridine	HC11,HC3,LC3
6.21	6.21	939.34	-1.20	C45H60CoN4O14	cobyrinate	HC11

6.22	6.32	130.12	-2.24	C7H15NO	N-Methylhexanamide	HC11,HC3,LC11,LC3
6.24	7.27	462.34	-1.57	C24H47NO7	Psychosine	HC3,LC3
6.24	4.30	107.07	-0.76	C4H10O3	Diethylene glycol	HC11,HC3,LC11,LC3
6.25	6.25	972.20	-1.11	C33H48N7O19P3S	(3-methylbenzyl)succinyl-CoA	HC11,LC11,LC3
6.26	7.66	279.10	1.12	C12H23O2Br	[FA (12:0)] 11-bromo-dodecanoic acid	LC11,LC3
6.26	5.39	174.09	-0.39	C11H11NO	1,3-Dimethyl-8-isoquinolinol	HC11,HC3,LC11,LC3
6.29	7.03	204.10	0.80	C12H13NO2	Indolebutyric acid	HC11,HC3,LC11,LC3
6.30	6.59	463.11	1.09	C15H29O10P3	Farnesyl triphosphate	LC11
6.30	4.10	260.19	-2.84	C13H25NO4	[FA (6:0)] O-hexanoyl-R-carnitine	LC11,LC3
6.30	7.60	777.55	-0.43	C45H76O10	MGDG(18:2(9Z,12Z)/18:3(9Z,12Z,15Z))	HC11
6.30	6.58	131.07	2.98	C6H10O3	(S)-3-Methyl-2-oxopentanoic acid	HC11,HC3,LC11,LC3
6.32	6.46	237.08	0.42	C12H12O5	5,6,7-Trimethoxycoumarin	HC3,LC11,LC3
6.32	5.67	140.03	0.66	C6H5NO3	6-Hydroxynicotinate	HC3,LC11,LC3
6.33	7.15	712.18	-1.53	C31H35O19	Succinylcyanin	HC11,HC3,LC11,LC3
6.33	6.33	455.25	-1.10	C20H38O11	octyl β-1,6-D-galactofuranosyl-α-D-glucopyranoside	HC11,HC3
6.34	7.69	459.27	0.19	C26H38N2O3S	[FA (20:4)] N-(4-benzenesulfonamide)-5Z,8Z,11Z,14Z-eicosatetraenoyl amine	HC3,LC11,LC3
6.34	5.94	218.08	2.30	C12H11NO3	3-Methylindolepyruvate	HC11,HC3,LC11,LC3
6.36	7.22	769.13	1.82	C35H28O20	Quercetin 3-(2'',6''-digalloylgalactoside)	LC11,LC3
6.36	7.73	819.58	1.51	C44H82O13	alpha,alpha'-Trehalose 6-mycolate	HC11,HC3
6.37	6.93	190.09	2.37	C11H11NO2	3-Indolepropionicacid	HC11,HC3,LC11,LC3
6.38	5.83	115.04	0.10	C5H6O3	2-Hydroxy-2,4-pentadienoate	HC11,HC3,LC11,LC3
6.39	7.27	804.58	1.43	C43H82NO10P	[GP (18:0/18:0)] 1-octadecanoyl-2-(9Z-octadecenoyl)-sn-glycero-3-phosphothreonine	HC11

6.39	6.52	116.11	-0.12	C6H13NO	hexanamide	HC11,HC3,LC11,LC3
6.39	6.59	160.08	0.90	C10H9NO	Indole-3-acetaldehyde	HC11,HC3,LC11,LC3
6.41	7.19	131.11	0.88	C7H14O2	[FA methyl(6:0)] 2-methyl-hexanoic acid	HC11,HC3,LC11,LC3
6.42	7.41	175.13	-2.34	C9H18O3	[FA hydroxy(9:0)] 2-hydroxy-nonanoic acid	HC11,HC3,LC3
6.43	4.17	216.12	-0.13	C10H17NO4	2-Amino-9,10-epoxy-8-oxodecanoic acid	HC3,LC11,LC3
6.45	7.94	269.13	-2.99	C21H16	3-Methylcholanthrene	HC3,LC3
6.45	5.41	564.40	-0.48	C29H58NO7P	[PC (3:0/18:2)] 2-(2E-propionyl)-3-octadecyl-sn-glycero-1-phosphocholine	HC11
6.46	6.46	887.30	-2.70	C42H54FeN8O6S2	Cytochrome C	HC11,HC3,LC3
6.46	7.42	843.61	-2.39	C47H87O10P	PG(19:1(9Z)/22:2(13Z,16Z))	HC11
6.46	5.63	330.23	-1.51	C17H31NO5	6-Keto-decanoylcarnitine	HC3,LC11,LC3
6.46	6.64	129.05	-2.34	C6H8O3	(4E)-2-Oxohexenoic acid	HC11,HC3,LC11,LC3
6.47	7.44	795.56	-2.49	C45H78O11	1-18:2-2-18:2-monogalactosyldiacylglycerol	HC11,HC3
6.49	7.97	439.31	1.12	C25H42O6	[PR] 3beta-(3-methyl-butanoyloxy)-villanovane-13alpha,17-diol	HC11
6.49	6.56	146.06	1.03	C9H7NO	3-Methyleneoxindole	HC11,HC3,LC11,LC3
6.50	6.76	204.09	-1.63	C9H9N5O	(5-Phenyl-1,2,4-triazol-3-yl)urea	HC11
6.51	6.58	139.08	-0.19	C8H10O2	4-Hydroxyphenylethanol	HC11,HC3,LC11,LC3
6.51	4.38	236.14	-2.37	C17H17N	(S)-7,8,13,14-Tetrahydroprotoberberine	HC11,HC3,LC11,LC3
6.51	5.65	144.07	1.83	C6H9NO3	Vinylacetylglycine	HC11,HC3,LC3
6.51	7.53	681.24	2.57	C32H40O16	3-(3-Methylbutyl)tricetin 5-neohesperidoside	HC11,HC3
6.52	6.52	868.29	0.06	C39H49NO21	Anthemis glycoside A	HC11

6.52	4.79	132.07	-2.63	C5H9NO3	N-Acetyl-beta-alanine	HC3
6.52	5.51	178.12	0.72	C11H15NO	Valerianine	HC11,HC3,LC3
6.52	5.54	531.37	0.61	C28H53NO6P	[PC (8:0)] 2-(8-[3]-ladderane-octanyl)-sn-glycero-3-phosphocholine	HC11
6.54	7.58	761.30	1.65	C45H44O11	Kuwanone H	HC11,HC3
6.55	7.60	637.14	-2.58	C28H28O17	Acacetin 7-glucuronosyl-(1->2)-glucuronide	HC11,HC3,LC11,LC3
6.55	5.78	190.12	-0.50	C12H15NO	1-Methyl-4-phenyl-1,2,3,6-tetrahydropyridine N-oxide	HC11,HC3,LC11,LC3
6.57	6.96	176.07	1.31	C10H9NO2	Indole-3-acetate	HC11,HC3,LC11,LC3
6.57	7.15	696.50	1.74	C39H70NO7P	PE(18:4(6Z,9Z,12Z,15Z)/P-16:0)	HC11
6.59	8.19	557.22	1.41	C27H37O10Cl	[FA methyl,oxo,hydroxy(3:0/3:0/3:0)] methyl 5S,6R,7R-triacetoxy-9-oxo-10-chloro-12S-hydroxy-10Z,14Z,17Z-prostatrienoate-cyclo[8R,12S]	HC3,LC11,LC3
6.60	8.20	499.36	-1.85	C35H46O2	[PR] Neurosporaxanthin	HC11
6.60	8.14	383.22	0.83	C24H30O4	[ST Dioxo(3:0)] 3,12-Dioxochola-1,4,9(11)-trien-24-oic Acid	HC3,LC11,LC3
6.61	7.22	856.61	1.37	C47H86NO10P	PS(19:1(9Z)/22:2(13Z,16Z))	HC11
6.62	5.62	186.11	0.25	C9H15NO3	8-keto-7-aminoperlagonate	HC11,HC3,LC11,LC3
6.64	4.02	304.18	2.99	C14H25NO6	Pimelylcarnitine	HC11,HC3,LC11,LC3
6.65	7.80	805.57	-2.49	C47H81O8P	PA(44:6)	HC11,HC3
6.65	8.31	615.33	-0.04	C31H51O10P	[PR] Dodecaprenyl phosphate-galacturonic acid	HC3,LC11,LC3
6.66	7.32	798.56	0.15	C44H80NO9P	PS(O-16:0/22:4(7Z,10Z,13Z,16Z))	HC11
6.66	6.66	930.15	-2.23	C30H42N7O19P3S	Caffeoyl-CoA	HC11
6.68	7.86	829.60	0.01	C46H85O10P	PG(40:3)	HC11
6.68	6.36	402.28	-1.85	C21H39NO6	[SP] Myriocin	HC11,HC3,LC3
6.68	7.36	495.31	2.00	C30H42N2O2S	Thiobinupharidine	HC3,LC3

6.69	8.19	550.39	2.04	C56H111N2O16P	[SP hydroxy,amino(26:0)] N-(hexacosanoyl)-4R-hydroxysphinganine-1-phospho-(1'-[2-amino-2-deoxy-D-glucopyranosyl-alpha1-6-D-myo-inositol])	HC11
6.69	4.16	222.13	0.38	C16H15N	Dizocilpine	HC11,HC3,LC11,LC3
6.70	7.91	240.14	1.15	C30H38O5	[Fv hydroxy(4:0)] 3-Geranyl-4,2',4',6'-tetrahydroxy-5-prenyldihydrochalcone	LC11
6.71	7.92	250.16	-2.48	C23H47O9P	PG(17:0/0:0)	HC3,LC3
6.72	5.51	193.11	-2.06	C8H16O5	D-Mycinose	HC11,HC3,LC11,LC3
6.72	7.94	839.60	1.81	C44H87O12P	PI(O-18:0/17:0)	HC11
6.72	7.94	663.23	-0.04	C32H38O15	Ikarisoside B	HC11
6.72	7.45	842.59	1.18	C46H84NO10P	PS(40:3)	HC11
6.73	8.05	361.22	2.19	C22H29FO3	9-Fluoro-16alpha-methylpregn-4-ene-3,11,20-trione	HC3,LC3
6.73	7.45	694.50	-1.33	C36H72NO9P	PS(O-16:0/14:0)	HC11
6.77	6.53	662.47	2.48	C35H67NO8S	[SP amino,methyl,hydroxy,methyl(14:0/18:0)] 2-carboxy-2-amino-3-O-(13'-methyltetradecanoyl)-4-hydroxy-17-methyloctadec-5-ene-1-sulfonic acid	HC11,HC3,LC11,LC3
6.78	3.85	124.04	1.56	C6H5NO2	Picolinic acid	HC11,HC3,LC11,LC3
6.81	5.82	130.09	-2.13	C6H11NO2	N4-Acetylaminobutanal	HC11,HC3,LC11,LC3
6.81	8.35	205.09	-1.25	C9H16O3S	2-oxo-8-methylthiooctanoate	HC11
6.82	8.15	217.13	-1.49	C25H36O6	Glycinoeclepin A	HC11,HC3,LC11,LC3
6.83	8.15	705.51	-2.08	C38H73O9P	PG(P-16:0/16:1(9Z))	HC11
6.83	8.16	933.68	-0.99	C51H97O12P	PI(O-20:0/22:2(13Z,16Z))	HC11
6.86	8.21	835.59	2.81	C48H83O9P	PG(P-20:0/22:6(4Z,7Z,10Z,13Z,16Z,19Z))	HC11
6.86	6.37	176.11	-0.29	C11H13NO	4-trans-(N,N-dimethylamino)cinnamaldehyde	HC11,HC3,LC3

6.89	7.87	188.07	0.23	C11H9NO2	Indoleacrylicacid	HC3,LC11,LC3
6.91	8.31	783.55	-2.61	C44H79O9P	PG(O-18:0/20:5(5Z,8Z,11Z,14Z,17Z))	HC11
6.91	6.91	884.15	1.37	C26H44N7O17P3S2	dimethylsulfoniopropioyl-CoA	LC11,LC3
6.92	6.44	248.08	0.44	C9H13NO7	N-Succinyl-L-glutamate	LC11
6.93	3.87	416.21	-2.09	C17H29N5O7	Asn-Val-Pro-Ser	HC3,LC11,LC3
6.94	7.02	199.06	0.72	C9H10O5	3-(3,4-Dihydroxyphenyl)lactate	HC3,LC11
6.96	3.91	475.13	-1.07	C18H26N4O7S2	Cys-Cys-Ser-Tyr	LC11
6.96	6.24	296.11	-2.69	C14H17NO6	(R)-Prunasin	HC3
6.97	7.08	198.11	-1.97	C10H15NO3	Tenuazonic acid	HC3,LC11,LC3
6.99	6.99	147.07	-0.81	C6H10O4	(S)-2-Aceto-2-hydroxybutanoate	HC11,HC3,LC11,LC3
6.99	7.80	224.09	-1.11	C11H13NO4	Bendiocarb	HC3
7.00	5.23	295.17	-2.44	C15H22N2O4	Leu-Tyr	LC11
7.06	7.40	162.06	0.94	C9H7NO2	4,6-Dihydroxyquinoline	HC11,HC3,LC3
7.07	9.32	323.16	-1.68	C21H22O3	[Fv] Derricin	HC11
7.08	7.11	149.06	0.08	C9H8O2	3-Hydroxy-1-indanone	HC3,LC11,LC3
7.11	4.22	430.27	-2.44	C19H35N5O6	Ala-Leu-Leu-Asn	HC11,HC3,LC11,LC3
7.12	4.22	244.15	-1.07	C12H21NO4	Tiglylcarnitine	HC3,LC11,LC3
7.12	9.24	419.16	-2.54	C23H31BrO2	18-bromo-8E,17E19Z-tricosatrien-4,6-diynoic acid	HC11,HC3,LC3
7.14	8.77	713.28	2.22	C37H44O14	124-1	HC3
7.14	8.28	401.21	-1.03	C22H28N2O5	11-O-Demethyl-17-O-deacetylvindoline	HC11

7.14	8.78	791.58	-0.19	C43H83O10P	PG(15:0/22:1(11Z))	HC11
7.15	8.31	585.32	-1.56	C32H44N2O8	Lappaconitine	HC11
7.19	5.09	372.18	0.53	C21H25NO5	Demecolcine	HC11,HC3
7.21	7.23	227.18	1.80	C12H22N2O2	1,8-Diazacyclotetradecane-2,9-dione	HC11,HC3,LC11,LC3
7.22	4.46	132.10	0.35	C6H13NO2	L-Norleucine	HC11,HC3,LC11,LC3
7.22	4.44	548.29	1.46	C27H41N5O5S	Ile-Met-Trp-Val	HC3,LC3
7.23	8.97	487.19	1.10	C28H26N2O6	Haplodimerine	HC11
7.25	4.49	474.29	-1.53	C21H39N5O7	Gln-Leu-Leu-Thr	HC11,HC3,LC11,LC3
7.28	9.07	777.54	1.32	C45H77O8P	PA(42:6)	HC11
7.30	4.61	508.17	1.95	C18H29N5O10S	Asp-Met-Asp-Gln	LC11,LC3
7.30	4.68	341.10	0.96	C14H16N2O8	Vulgaxanthin-II	LC11
7.33	7.14	183.08	0.87	C6H15O4P	Diisopropyl phosphate	HC11,HC3,LC11,LC3
7.34	8.69	748.53	0.26	C43H74NO7P	[PE (16:1/22:6)] 1-O-(1Z-hexadecenyl)-2-(4Z,7Z,10Z,13Z,16Z,19Z-docosahexaenoyl)-sn-glycero-3-phosphoethanolamine	HC11
7.35	7.35	960.17	1.59	C31H44N7O20P3S	5-Hydroxyferuloyl-CoA	HC11
7.39	4.55	237.10	-1.11	C12H14NO4	Salsoline-1-carboxylate	HC3
7.39	7.39	379.22	0.04	C37H56N8O9	Nostocyclopeptide A1	HC3,LC11,LC3
7.40	6.80	214.02	-2.77	C8H7NO4S	Indoxylsulfate	HC3,LC11,LC3
7.40	8.70	356.12	-2.70	C13H25NO6S2	5-methylthiopentyldesulfoglucosinolate	LC11
7.41	7.31	468.27	-0.97	C21H42NO8P	[PC (6:0/7:0)] 1-hexanoyl-2-heptanoyl-sn-glycero-3-phosphocholine	HC11,HC3
7.41	4.72	226.12	-0.41	C15H15NO	2-[2-(4-Pyridinyl)-1-butenyl]phenol	HC11,HC3,LC11,LC3
7.42	9.56	294.14	-2.12	C15H20ClN3O	paclobutrazol	HC11
7.43	9.36	311.22	2.27	C32H60O11	Butyl 4'-O-hexadecanoyl-neohesperidoside	HC11,LC11

7.45	9.33	273.14	-0.45	C19H16N2	Sempervirine	HC3
7.49	7.47	882.60	-0.06	C52H84NO8P	PC(44:10)	HC11,HC3,LC3
7.50	4.99	410.16	2.23	C18H23N3O8	Glu-Asp-Phe	HC3
7.53	4.90	189.14	-0.77	C12H16N2	N,N-Dimethyltryptamine	HC11,HC3,LC11,LC3
7.54	8.72	249.12	-2.07	C13H16N2O3	6-Hydroxymelatonin	HC3
7.56	10.16	299.16	-2.94	C19H22O3	Ostruthin	HC11,HC3,LC11
7.57	8.87	164.11	2.64	C10H13NO	N-Acetylphenylethylamine	HC11,HC3
7.58	9.65	791.56	0.13	C46H79O8P	[PG (8:0/8:0)] 1-(8-[3]-ladderane-octanyl)-2-(8-[3]-ladderane-octanyl)-sn-glycero-3-phospho-(1'-sn-glycerol)	HC11
7.61	9.07	361.20	-1.14	C21H28O5	Cortisone	HC3
7.66	9.94	343.15	1.35	C20H22O5	[Fv] Brosimacutin C	HC11,HC3,LC11,LC3
7.66	7.77	123.06	2.59	C6H6N2O	Picolinamide	HC3,LC11,LC3
7.66	5.31	402.23	-2.90	C17H31N5O6	Ala-Leu-Ala-Gln	HC3,LC11,LC3
7.66	7.38	197.13	-1.95	C10H16N2O2	Fasoracetam	HC11,HC3,LC11,LC3
7.67	10.50	185.09	1.29	C22H24O5	[Fv] 4'-O-Methylxanthohumol	LC3
7.67	8.49	103.04	1.54	C4H6O3	2-Oxobutanoate	HC11,HC3,LC11,LC3
7.68	8.87	699.27	0.36	C37H38N4O10	PentacarboxylporphyrinI	HC3
7.71	9.91	581.35	-2.81	C28H52O12	alpha,alpha'-Trehalose 6-palmitate	HC3
7.73	5.62	166.09	-1.05	C9H11NO2	D-Phenylalanine	HC11,HC3,LC11,LC3
7.74	10.28	278.12	-0.64	C12H23NO2S2	S-(2-Methylpropanoyl)-dihydrolipoamide	HC3
7.74	5.48	409.24	-1.74	C20H32N4O5	Pro-Val-Pro-Pro	HC3

7.75	10.18	391.29	1.30	C24H38O4	[ST hydrox] 3alpha,12alpha-Dihydroxy-5beta-chol-8(14)-en-24-oic Acid	HC11,HC3,LC11,LC3
7.76	8.76	128.07	1.24	C6H9NO2	delta1-Piperideine-2-carboxylate	HC11,HC3,LC11,LC3
7.76	5.53	446.26	-2.11	C19H35N5O7	Asn-Leu-Leu-Ser	HC3,LC11,LC3
7.77	10.05	491.19	-2.57	C25H30O10	[Fv Hydroxy,dimethoxy(9:1/9:1)] 5-Hydroxy-7,4'-dimethoxy-6,8-di-C-prenylflavanone 5-O-galactoside	HC3
7.78	9.24	211.11	0.46	C10H14N2O3	Aprobarbital	HC3,LC3
7.78	9.45	191.09	2.35	C7H14N2O2S	Aldicarb	HC11,HC3,LC3
7.78	7.16	300.11	-1.87	C13H17NO7	p-aminobenzoate-β-D-glucopyranosyl ester	HC3
7.78	9.66	189.08	2.20	C8H12O5	2-oxosuberate	HC11,HC3,LC11,LC3
7.79	4.85	399.26	-2.67	C19H34N4O5	Ala-Leu-Val-Pro	HC3
7.79	6.44	248.11	-1.32	C10H17NO6	Linamarin	HC3
7.80	8.29	187.06	1.10	C8H10O5	3-hydroxy-3-carboxy-4,5-cyclopropylhex-5-enoate	HC11,HC3,LC3
7.80	10.60	219.09	1.19	C9H14O6	Triacetin	LC3
7.81	8.49	174.09	1.33	C11H11NO	1,3-Dimethyl-8-isoquinolinol	HC11,HC3,LC3
7.83	8.73	179.08	-2.28	C9H10N2O2	Phenacemide	HC11,HC3,LC11,LC3
7.84	10.69	537.23	-1.05	C22H45ClO8S2	13-chloro-docosane-1,14-disulfate	HC11,HC3
7.85	5.70	443.29	-1.45	C21H38N4O6	Ile-Leu-Thr-Pro	HC3
7.86	10.23	709.49	2.67	C37H73O8PS	[GP (14:0/16:0)] 1-tetradecanoyl-2-hexadecanoyl-sn-glycero-3-phosphosulfocholine	HC11
7.87	10.73	309.20	0.99	C16H27F3O2	14,14,14-Trifluoro-11E-tetradecenyl acetate	HC11,LC11
7.87	5.35	336.17	0.04	C15H21N5O4	Isopentenyl adenosine	HC3
7.87	5.75	518.23	-2.58	C23H31N7O7	Gln-Trp-Gln-Gly	HC3,LC11
7.88	9.76	792.55	-0.22	C45H78NO8P	PE(40:6)	HC11

7.88	5.20	127.02	1.17	C2H7O4P	Ethylphosphate	HC11,HC3,LC11,LC3
7.89	9.81	363.20	1.86	C21H27FO4	17,21-Epoxy-9-fluoro-11beta-hydroxypregn-4-ene-3,20-dione	HC11,HC3
7.92	4.98	327.08	2.92	C13H14N2O8	Miraxanthin-II	HC3,LC11
7.93	7.71	206.14	1.83	C9H19NO4	Pantothenol	HC11,HC3,LC11,LC3
7.94	9.03	177.08	-1.64	C7H12O5	(2S)-2-Isopropylmalate	HC3,LC11,LC3
7.94	7.94	292.18	0.73	C27H46N6O8	N1-(3,4-dihydroxybenzoyl)-N8,N'8-citryl-bis(spermidine)	HC11,HC3,LC11,LC3
7.94	10.38	543.13	0.46	C30H22O10	Isochamaejasmin	HC11,HC3,LC11,LC3
7.95	11.18	386.25	-1.62	C24H33O4	9'-Carboxy-alpha-tocotrienol	HC11,HC3
7.96	9.42	599.29	0.91	C34H38N4O6	haematoporphyrin IX	HC3,LC11
7.98	10.14	242.14	1.28	C12H19NO4	N-(3-Oxooctanoyl)homoserine lactone	HC3,LC3
8.02	6.19	120.07	-1.19	C4H9NO3	(-)-erythro-(2R,3R)-dihydroxybutylamide	HC11,HC3
8.02	5.00	212.13	-2.24	C11H17NO3	Mescaline	HC3
8.02	9.55	655.28	0.69	C36H38N4O8	Coproporphyrin I	HC3,LC11
8.03	4.95	191.12	-2.26	C11H14N2O	N-Methylserotonin	HC11,HC3,LC11,LC3
8.04	10.38	396.10	-2.61	C15H17N5O6S	Tribenuron methyl	HC11,HC3
8.06	6.12	445.12	-2.65	C17H24N4O6S2	Cys-Cys-Gly-Tyr	HC11,HC3,LC11,LC3
8.07	8.46	146.08	-0.40	C6H11NO3	4-Acetamidobutanoate	HC3
8.07	4.62	291.12	2.30	C11H18N2O7	N-Succinyl-L-L-2-6-diaminopimelate	HC11,HC3
8.08	8.31	123.01	0.15	C3H6O3S	3-Mercaptolactate	HC11,HC3,LC11
8.08	10.92	254.12	-2.51	C13H19NO2S	Fenothiocarb	HC3,LC11
8.09	11.18	419.32	-0.37	C26H42O4	[ST (3:0)] (5Z,7E)-(1S,3R,20R,24R)-22-oxa-9,10-seco-5,7,10(19)-cholestatriene-1,3,24-triol	HC11,HC3,LC11,LC3
8.10	10.70	545.38	0.32	C40H48O	18-hydroxyrenierapurpurin	HC11,HC3,LC11,LC3

8.10	6.21	607.29	-1.46	C36H38N4O5	Phe-Phe-Phe-Phe	HC11,HC3,LC11
8.11	10.71	411.04	2.79	C17H14O10S	6-Hydroxyluteolin 6,3'-dimethyl ether 7-sulfate	HC11,HC3,LC11
8.11	10.71	545.33	-2.78	C35H44O5	[Fv hydroxy(4:0)] 5,3'-Digeranyl-3,4,2',4'-tetrahydroxychalcone	HC11
8.11	8.71	197.03	-0.74	C6H4N4O4	Xanthine-8-carboxylate	HC11,HC3,LC11,LC3
8.11	8.26	238.16	-0.16	C17H19N	N-Methyl-(R,S)-tetrahydrobenzylisoquinoline	HC11,HC3,LC11,LC3
8.11	6.23	523.22	1.74	C22H30N6O9	Asn-Phe-Asp-Gln	HC11,HC3
8.12	11.61	284.30	0.24	C18H37NO	Octadecanamide	HC11,HC3,LC11,LC3
8.13	8.02	295.12	2.42	C15H18O6	Tutin	HC3,LC3
8.19	8.19	574.15	-1.58	C20H28N7O9PS	Biotinyl-5'-AMP	LC11
8.19	9.74	194.10	1.45	C14H11N	2-Anthramine	HC11,HC3,LC11,LC3
8.21	10.28	149.06	1.23	C9H8O2	4-Hydroxycinnamyl aldehyde	HC11,HC3,LC11,LC3
8.21	8.92	854.57	-1.59	C50H80NO8P	PC(42:10)	HC3,LC11,LC3
8.22	9.95	567.30	0.10	C34H38N4O4	MesoporphyrinIX	HC11,HC3,LC11,LC3
8.24	10.02	211.09	2.58	C13H10N2O	2-Aminoacridone	HC3,LC3
8.28	11.91	390.28	2.96	C24H37O4	9'-Carboxy-alpha-chromanol	HC11
8.31	10.61	450.29	0.11	C21H42N2O6P	[SP] N-(acyl)-sphing-4-enine-1-phosphoethanolamine	HC11
8.32	11.13	409.19	-1.80	C21H28O8	Vernoflexuoside	HC11,HC3,LC11,LC3
8.32	11.45	796.53	2.72	C40H77NO12S	[SP] (3'-sulfo)Galbeta-Cer(d18:1/2-OH-16:0)	HC3,LC11,LC3
8.32	8.55	374.20	-2.57	C21H27NO5	(S)-autumnaline	HC11,HC3,LC11,LC3
8.36	11.22	407.28	1.62	C24H38O5	3alpha,12alpha-Dihydroxy-7-oxo-5beta-cholanate	HC3,LC11,LC3
8.36	6.63	236.08	2.01	C9H9N5O3	6-Succinoaminopurine	HC11,HC3
8.37	11.24	811.57	-0.23	C42H83O12P	PI(O-18:0/15:0)	HC11
8.37	8.92	134.05	1.24	C9H18N2O3S2	S-5-methylthiopentylhydroximoyl-L-cysteine	HC11,HC3,LC11,LC3
8.37	8.37	852.18	2.73	C26H44N7O17P3S	3-Methylbutanoyl-CoA	HC3,LC11,LC3

8.38	8.34	116.03	1.74	C4H5NO3	Maleamate	HC3,LC11,LC3
8.40	9.76	172.10	-0.02	C8H13NO3	N-Butyryl-L-homoserine lactone	HC3,LC11,LC3
8.43	6.86	483.25	0.25	C22H34N4O8	Ile-Thr-Ser-Tyr	HC3
8.44	10.24	111.04	0.16	C6H6O2	Catechol	HC11,HC3,LC11,LC3
8.44	6.89	596.25	0.43	C30H37N5O6S	Met-Trp-Pro-Tyr	HC3,LC3
8.45	5.12	253.12	-1.13	C12H16N2O4	Ala-Tyr	HC3,LC3
8.45	11.09	214.14	-2.95	C11H19NO3	N-Heptanoylhomoserine lactone	HC3,LC3
8.47	6.17	288.11	-1.32	C12H17NO7	Volkenin	HC11,HC3,LC11
8.47	11.95	455.21	-1.10	C19H34O12	3-O-(alpha-L-arabinopyranosyl-(1->6)-beta-D-glucopyranosyl) butyl 3S-hydroxybutanoate	HC3,LC3
8.48	10.43	142.05	-0.83	C6H7NO3	2,5,6-trihydroxy-3-methylpyridine	HC11,HC3,LC11,LC3
8.49	10.45	152.07	-0.03	C8H9NO2	(Z)-4-Hydroxyphenylacetaldehyde-oxime	HC3,LC11,LC3
8.50	5.37	157.06	-2.21	C6H8N2O3	4-Imidazolone-5-propanoate	HC3,LC3
8.50	11.50	581.19	2.09	C27H32O14	Narirutin	HC3
8.52	6.65	182.05	-2.04	C6H7N5S	thioadenine S-methylether	HC3,LC11,LC3
8.52	9.05	113.04	1.57	C4H4N2O2	Uracil	HC11,HC3,LC11,LC3
8.53	10.65	217.03	1.97	C9H6F2O4	(2,6-difluoro-4-hydroxyphenyl)pyruvate	LC3
8.53	11.57	481.33	-1.89	C24H49O7P	PA(21:0/0:0)	HC11,LC11,LC3
8.54	7.08	485.19	1.40	C21H32N4O5S2	Cys-Leu-Phe-Cys	HC3,LC3
8.54	11.55	188.06	-2.14	C19H18O8	Chrysosplenetin	LC11
8.55	5.82	168.07	2.90	C8H9NO3	Isopyridoxal	HC11,HC3,LC11,LC3
8.55	9.60	482.29	-1.24	C22H44NO8P	[PC (10:0/4:2)] 1-decanoyl-2-butyryl-sn-glycero-3-phosphocholine	HC11,HC3,LC11,LC3

8.55	11.10	840.55	-1.88	C49H78NO8P	PE(44:10)	HC3,LC11,LC3
8.55	7.00	226.12	1.05	C15H15NO	2-[2-(4-Pyridinyl)-1-butenyl]phenol	HC11,HC3,LC11,LC3
8.56	10.38	148.10	-0.76	C6H13NO3	N-hydroxyisoleucine	HC3,LC3
8.57	7.15	540.23	0.52	C27H33N5O5S	Met-Phe-Trp-Gly	HC11,HC3,LC3
8.58	10.31	210.08	2.87	C10H11NO4	4-Hydroxyphenylacetylglycine	HC11,HC3,LC11,LC3
8.58	7.15	508.22	-0.49	C21H29N7O8	Asn-Phe-Asn-Asn	HC11,HC3,LC11,LC3
8.58	10.16	774.20	2.47	C36H37O19	Delphinidin 3-O-beta-D-glucoside 5-O-(6-coumaroyl-beta-D-glucoside)	HC11,HC3
8.59	11.67	395.22	-2.15	C18H35O7P	PA(15:1(9Z)/0:0)	HC3,LC3
8.61	11.35	243.09	1.31	C12H10N4O2	Lumichrome	HC3,LC3
8.62	6.61	155.01	2.15	C3H7O5P	Propanoyl phosphate	HC11,LC3
8.67	12.33	405.30	-2.80	C25H40O4	[ST (3:0)] (5Z,7E)-(1S,3R)-21-nor-20-oxa-9,10-seco-5,7,10(19)-cholestatriene-1,3,25-triol	HC11,HC3,LC11,LC3
8.67	10.31	160.10	0.30	C7H13NO3	5-Acetamidopentanoate	HC3,LC11,LC3
8.68	7.36	509.20	-1.91	C21H28N6O9	Asn-Phe-Asn-Asp	HC11,HC3,LC11,LC3
8.69	8.32	173.05	0.77	C7H8O5	3-Dehydroshikimate	HC11,HC3,LC11,LC3
8.69	10.39	160.06	1.84	C6H9NO4	beta-Alanopine	HC3,LC3
8.70	9.99	185.05	2.29	C8H8O5	3,4-Dihydroxymandelate	HC11,LC11,LC3
8.71	11.42	243.05	0.88	C6H13NO7P	[PE] 1-acyl-sn-glycero-3-phosphoethanolamine	HC11,LC11,LC3
8.73	12.23	268.15	0.44	C14H21NO4	Diethofencarb	HC3,LC3
8.74	4.86	222.10	0.69	C8H15NO6	N-Acetyl-D-mannosamine	HC3
8.75	10.34	189.04	2.18	C7H8O6	(Z)-But-2-ene-1,2,3-tricarboxylate	HC3,LC11,LC3
8.77	12.03	473.11	-2.32	C23H20O11	Tetracenomycin C	HC3,LC11,LC3
8.77	6.30	217.15	-2.11	C10H20N2O3	Val-Val	HC3,LC11,LC3
8.78	9.06	124.04	2.31	C6H5NO2	Nicotinate	HC11,HC3,LC11,LC3
8.81	5.19	127.04	1.48	C8H16N2O3S2	Met-Cys	HC3

8.83	8.44	222.13	1.58	C16H15N	Dizocilpine	HC11,LC11,LC3
8.86	11.40	166.05	-1.20	C8H7NO3	Formylanthranilate	HC3,LC11,LC3
8.86	12.67	273.11	-2.49	C16H16O4	[Fv hydroxy,methox] 2',6'-Dihydroxy-4'-methoxydihydrochalcone	HC11,HC3
8.86	7.72	210.09	-1.10	C16H26N4O9	Ala-Val-Asp-Asp	HC11,LC11
8.87	10.59	204.09	1.03	C8H13NO5	N2-Acetyl-L-aminoadipate	HC11,HC3,LC11,LC3
8.89	12.27	595.36	-0.43	C36H50O7	Glycosyl-4,4'-diaponeurosporenoate	HC11,HC3
8.89	5.96	269.08	0.53	C11H12N2O6	Portulacaxanthin III	LC11,LC3
8.90	7.80	208.11	-2.85	C18H30N4O7	Glu-Leu-Gly-Pro	HC11,HC3,LC11,LC3
8.91	10.31	826.54	-2.87	C48H76NO8P	PC(18:4(6Z,9Z,12Z,15Z)/22:6(4Z,7Z,10Z,13Z,16Z,19Z))	HC3,LC11,LC3
8.91	8.06	294.12	1.73	C11H19NO8	N-Acetylmuramate	HC3,LC11,LC3
8.92	7.65	276.11	1.16	C11H17NO7	Cardiospermin	HC11,HC3,LC11,LC3
8.93	10.36	552.37	0.44	C27H54NO8P	[PC (10:0/9:0)] 1-decanoyl-2-nonanoyl-sn-glycero-3-phosphocholine	HC11,HC3,LC3
8.93	7.86	218.10	-0.91	C20H26N4O7	Asp-Phe-Gly-Pro	HC11,HC3,LC11,LC3
8.94	7.88	565.22	2.87	C28H32N6O5S	Ala-Trp-Trp-Cys	HC3,LC3
8.94	7.89	468.17	-0.72	C19H25N5O9	Asn-Asp-Gly-Tyr	HC11,HC3,LC11,LC3
8.95	12.90	475.32	-1.60	C32H42O3	[ST hydroxy,methyl,ethyl(2:0/2:0/2:0)] (5Z,7E)-(1S,3R)-23-[3-(1-hydroxy-1-methylethyl)phenyl]-24-nor-9,10-seco-5,7,10(19)-cholatrien-22-yne-1,3-diol	HC11,HC3,LC11,LC3
8.95	9.76	128.04	2.84	C5H5NO3	2,3,6-Trihydroxypyridine	HC3,LC11,LC3
8.95	7.90	609.21	-1.56	C29H32N6O7S	Asp-Trp-Trp-Cys	HC3,LC3
8.96	12.42	539.21	2.83	C33H30O7	8-trans-[2-(6-Benzoyloxy-4-hydroxy-2-methoxy-3-methylphenyl)ethenyl]-5-methoxyflavan-7-ol	HC3,LC11,LC3
8.99	12.73	322.12	1.72	C13H23NO4S2	S-Glutaryldihydrolipoamide	HC3,LC3

8.99	8.99	560.14	-1.56	C18H31N3O13P2	dTDP-alpha-D-desosamine	HC3,LC11,LC3
9.01	12.06	201.04	2.97	C12H8OS	dibenzothiophene-5-oxide	HC3,LC11,LC3
9.04	12.59	395.04	0.81	C17H14O9S	Kaempferol 7,4'-dimethyl ether 3-O-sulfate	HC11,HC3,LC11,LC3
9.08	12.65	681.51	-1.95	C36H73O9P	PG(O-16:0/14:0)	HC11,LC11
9.08	9.08	872.15	-2.53	C28H40N7O17P3S	S-Benzoate coenzyme A	HC3,LC11,LC3
9.08	12.16	790.60	2.44	C43H84NO9P	PS(O-18:0/19:1(9Z))	HC11
9.09	13.18	639.57	-1.37	C38H74N2O5	[FA (16:0/17:0)] N-(3-(hexadecanoyloxy)-heptadecanoyl)-L-ornithine	HC11
9.09	8.18	258.13	-1.91	C26H34N4O7	Ala-Val-Tyr-Tyr	HC11,HC3,LC11,LC3
9.09	8.18	471.17	1.61	C20H30N4O5S2	Ala-Met-Phe-Cys	HC3,LC3
9.10	12.19	547.15	1.26	C26H26O13	afrormosin-7-O-glucoside-6''-O-malonate	HC11,HC3,LC11,LC3
9.10	8.25	259.09	2.49	C10H14N2O6	Ribothymidine	HC11,HC3,LC11,LC3
9.14	5.93	304.10	1.22	C12H17NO8	Gynocardin	HC3,LC11,LC3
9.15	12.81	795.59	-1.38	C46H83O8P	PA(21:0/22:4(7Z,10Z,13Z,16Z))	HC11
9.15	13.11	644.51	0.88	C36H69NO8	Glucosylceramide (d18:1/12:0)	HC11
9.16	7.89	352.13	2.24	C13H21NO10	N-Acetyl-4-O-acetylneuraminate	HC11,HC3,LC11,LC3
9.16	8.33	234.13	-2.22	C22H34N4O7	Ala-Leu-Thr-Tyr	HC11,HC3,LC11,LC3
9.17	8.93	115.05	2.59	C4H6N2O2	5,6-Dihydrouracil	HC3,LC3
9.17	9.17	853.17	-2.39	C25H43N8O17P3S	L-3-Aminobutyryl-CoA	HC3,LC11,LC3
9.18	12.35	806.59	1.58	C43H84NO10P	[PS (18:0/19:0)] 1-octadecanoyl-2-nonadecanoyl-sn-glycero-3-phosphoserine	HC11
9.19	12.39	776.58	-0.22	C42H82NO9P	PS(O-16:0/20:1(11Z))	HC11
9.20	8.40	552.24	-0.53	C23H33N7O9	Asn-Gln-Gln-Tyr	HC3,LC3
9.20	5.98	216.12	0.04	C10H17NO4	Propenoylcarnitine	HC3,LC11,LC3

9.20	9.87	151.06	1.05	C6H6N4O	1-Methylhypoxanthine	HC3,LC11,LC3
9.22	10.93	550.35	-1.04	C27H52NO8P	[PC (16:0/3:0)] 1-hexadecanoyl-2-(2E-propionyl)-sn-glycero-3-phosphocholine	HC11,LC11,LC3
9.24	8.48	569.21	2.79	C24H32N4O12	Tyr-Glu-Glu-Glu	HC3
9.24	10.99	832.59	1.14	C48H82NO8P	PC(40:7)	HC11
9.24	11.36	174.09	1.66	C11H11NO	1,3-Dimethyl-8-isoquinolinol	HC11,HC3,LC3
9.26	6.80	274.09	-0.23	C12H19NO2S2	Brugine	HC3
9.26	12.52	782.60	-2.70	C45H84NO7P	PE(22:2(13Z,16Z)/P-18:1(11Z))	HC11
9.29	6.43	176.09	2.88	C7H13NO4	N-Carboxyethyl-g-aminobutyricacid	HC3,LC3
9.29	11.08	538.35	2.93	C26H52NO8P	[PC acetyl(16:0)] 2-acetyl-3-hexadecanoyl-sn-glycero-1-phosphocholine	HC11,HC3,LC11,LC3
9.29	7.76	257.06	2.76	C10H12N2O4S	serotonin O-sulfate	HC3,LC3
9.29	8.59	263.11	1.05	C23H32N4O8S	Asp-Met-Pro-Tyr	LC3
9.31	12.61	780.59	-2.87	C45H82NO7P	PE(22:4(7Z,10Z,13Z,16Z)/P-18:0)	HC11
9.31	12.61	402.41	-2.36	C28H51N	3beta,4-Dimethyl-4-aza-5alpha-cholestane	HC11
9.31	8.63	494.19	2.50	C25H27N5O4S	Trp-Trp-Cys	HC11,HC3,LC11,LC3
9.31	12.63	812.52	-1.12	C47H74NO8P	PE(42:10)	HC3,LC11,LC3
9.32	12.64	866.59	2.77	C48H84NO10P	PS(42:5)	HC11
9.33	10.87	169.10	-1.18	C8H12N2O2	Cyclo(deltaAla-L-Val)	HC11,HC3,LC11,LC3
9.34	13.18	489.17	-0.45	C32H24O5	Dracorubin	HC3,LC3
9.35	8.31	125.07	2.22	C6H8N2O	Methylimidazole acetaldehyde	HC11,HC3,LC11,LC3
9.35	9.35	326.38	-0.31	C22H47N	di-n-Undecylamine	HC11,HC3,LC11,LC3
9.36	8.71	612.25	2.38	C30H37N5O7S	Glu-Met-Phe-Trp	HC3
9.36	10.62	255.15	-1.76	C16H18N2O	Amphenone B	HC3,LC3

9.36	9.53	125.04	1.19	C3H9O3P	propylphosphonate	HC3,LC3
9.36	9.80	179.08	-2.05	C9H10N2O2	4-Oxo-4-(3-pyridyl)-butanamide	HC3,LC11,LC3
9.36	9.08	137.05	2.27	C5H4N4O	Hypoxanthine	HC3,LC11,LC3
9.37	10.10	236.14	1.69	C17H17N	(S)-7,8,13,14-Tetrahydroprotoberberine	HC11,HC3,LC11,LC3
9.38	12.77	630.59	-1.07	C40H75N3O2	Juliflorine	HC11,HC3
9.39	13.28	474.41	0.61	C34H51N	N-Methylindolo[3,2-b]-5alpha-cholest-2-ene	HC11
9.41	8.66	237.09	1.39	C11H12N2O4	L-Formylkynurenine	HC3
9.43	11.69	242.07	0.44	C10H11NO6	N-(2,3-Dihydroxybenzoyl)-L-serine	LC11,LC3
9.43	13.41	365.31	0.13	C23H40O3	[ST (3:0)] 24-Nor-5beta-cholane-3alpha,6alpha,23-triol	HC11,HC3,LC3
9.43	8.64	211.11	2.33	C10H14N2O3	2,6-Dihydroxypseudooxynicotine	HC3,LC11,LC3
9.43	12.87	484.30	0.62	C22H46NO8P	PS(O-16:0/0:0)	HC11,HC3,LC11,LC3
9.45	11.40	414.36	-2.20	C24H47NO4	Heptadecanoylcarnitine	HC11,HC3,LC11
9.46	6.56	247.13	0.62	C10H18N2O5	Glu-Val	HC3,LC11,LC3
9.46	12.93	460.39	0.45	C33H49N	1'H-5alpha-Cholest-2-eno[3,2-b]indole	HC11,HC3
9.46	12.35	174.11	-0.50	C8H15NO3	N-Acetyl-L-leucine	LC11
9.47	6.41	217.13	-0.09	C13H16N2O	Tetrahydroharmine	HC3,LC11,LC3
9.47	13.44	723.21	-2.70	C33H38O18	6'''-(3-Hydroxy-3-methylglutaroyl)isoviolanthin	HC11
9.49	11.72	317.14	-1.92	C14H16N6O3	tetrahydropteroate	HC11,HC3,LC3
9.51	13.53	833.63	-0.51	C46H89O10P	PG(40:1)	HC11
9.52	13.05	440.24	-0.49	C26H33NO5	Militarinone B	HC11,HC3,LC11,LC3
9.53	13.55	224.11	-2.58	C15H13NO	2-Acetamidofluorene	HC11,HC3,LC11,LC3
9.54	9.08	623.23	-0.98	C30H34N6O7S	Glu-Trp-Trp-Cys	HC11,HC3,LC11

9.55	12.32	249.09	0.54	C12H12N2O4	5-Hydroxyindoleacetylglycine	LC3
9.55	9.10	263.12	-1.93	C24H36N4O7S	Asp-Leu-Met-Phe	HC11,HC3,LC11,LC3
9.55	13.61	761.64	0.58	C44H89O7P	PA(O-20:0/21:0)	HC11
9.56	9.11	539.18	-2.92	C20H34N4O9S2	Glu-Glu-Met-Met	HC3
9.56	9.02	241.13	2.25	C15H16N2O	Prolyl-2-naphthylamide	HC3,LC11,LC3
9.56	13.62	663.53	-1.83	C37H75O7P	PA(16:0e/18:0)	HC11,LC11
9.56	9.79	200.06	2.98	C8H9NO5	Clavulanic acid	HC3
9.56	13.63	759.63	0.81	C44H87O7P	PA(P-20:0/21:0)	HC11
9.57	8.40	139.09	-0.12	C7H10N2O	4-Hydroxymethylphenylhydrazine	HC3,LC11,LC3
9.58	13.65	733.61	-2.30	C42H85O7P	PA(O-18:0/21:0)	HC11
9.58	13.66	421.27	0.14	C28H36O3	Tingenone	HC11
9.58	13.55	184.11	-0.50	C10H15O3	(3R)-3-Isopropenyl-6-oxoheptanoate	HC3,LC11
9.60	10.77	142.05	0.19	C6H7NO3	6-oxo-1,4,5,6-tetrahydronicotinate	HC3,LC11,LC3
9.61	13.71	731.60	0.56	C42H83O7P	PA(O-20:0/19:1(9Z))	HC11,LC11
9.61	13.85	389.30	-2.92	C25H40O3	[PR (2:0)] (+)-24,25-epoxy-16-scalaren-12alpha,25alpha-diol	HC11
9.62	13.74	719.59	-1.30	C41H83O7P	PA(O-20:0/18:0)	HC11
9.63	13.76	691.56	0.43	C39H79O7P	PA(O-20:0/16:0)	HC11
9.63	13.76	767.59	-2.88	C45H83O7P	PA(O-20:0/22:4(7Z,10Z,13Z,16Z))	HC11
9.65	13.81	825.59	0.22	C43H85O12P	PI(O-20:0/14:0)	HC11,LC11
9.65	13.81	689.55	0.62	C39H77O7P	PA(O-16:0/20:1(11Z))	HC11
9.67	9.67	467.17	-1.09	C21H26N2O10	indole-3-acetyl-glutamate-N-beta-D-glucose	HC11
9.68	13.86	365.14	-1.07	C22H20O5	Alpinumisoflavone dimethyl ether	HC3,LC3

9.68	13.86	453.33	-1.75	C23H49O6P	PA(O-20:0/0:0)	HC11,HC3,LC11,LC3
9.68	12.96	203.06	2.35	C8H10O6	cis-(homo)2aconitate	HC3,LC11,LC3
9.68	9.68	575.22	1.35	C24H30N8O9	Tetrahydrofolyl-[Glu](2)	HC3
9.68	9.68	465.07	2.74	C16H20N2O10S2	4-hydroxy-3-indolylmethyl-glucosinolate	HC11,HC3
9.69	13.87	675.53	1.44	C38H75O7P	PA(O-16:0/19:1(9Z))	HC11
9.69	13.88	701.55	0.65	C40H77O7P	PA(O-20:0/17:2(9Z,12Z))	HC11
9.69	10.63	153.04	0.79	C5H4N4O2	Xanthine	HC11,HC3,LC11,LC3
9.70	13.91	479.08	-2.18	C21H18O13	Isoetin 4'-glucuronide	HC11
9.71	9.41	510.18	-0.69	C21H27N5O10	Asn-Phe-Asp-Asp	HC3
9.71	13.93	749.53	2.35	C40H77O10P	PG(34:1)	HC11
9.72	11.61	134.05	0.21	C9H18N2O3S2	S-5-methylthiopentylhydroximoyl-L-cysteine	HC3,LC11,LC3
9.72	9.72	628.26	1.33	C24H41N3O16	Tri-N-acetylchitotriose	HC11
9.72	13.94	405.13	-2.26	C24H20O6	[Fv] Calomelanol C	LC11
9.72	13.94	709.50	-0.81	C37H73O10P	PG(12:0/19:0)	HC11,HC3
9.72	13.94	440.41	-0.32	C27H53NO3	Pentacosanoylglycine	LC11
9.72	13.95	703.57	2.88	C40H79O7P	PA(O-18:0/19:1(9Z))	HC11
9.73	13.95	933.64	-1.15	C50H93O13P	PI(19:0/22:2(13Z,16Z))	HC11,HC3
9.73	13.96	691.30	-0.37	C35H46O14	Zaragozic acid A	HC11,HC3
9.73	9.46	489.27	-1.66	C25H36N4O6	Ile-Pro-Pro-Tyr	HC3,LC11,LC3
9.76	12.02	524.41	2.78	C27H58NO6P	[PC methyl(18:2)] 1-methyl-2-octadecyl-sn-glycero-3-phosphocholine	HC11
9.78	7.80	150.10	1.14	C8H11N3	benzylguanidine	HC3,LC11,LC3
9.78	8.31	190.07	-0.71	C7H11NO5	L-2-Amino-6-oxoheptanedioate	HC3

9.80	7.87	128.07	2.05	C6H9NO2	2,3,4,5-Tetrahydropyridine-2-carboxylate	HC3,LC11,LC3
9.80	14.10	705.47	0.70	C37H69O10P	PG(13:0/18:2(9Z,12Z))	HC11
9.80	14.10	661.52	-0.03	C37H73O7P	PA(O-20:0/14:1(9Z))	HC11
9.80	13.39	254.07	0.17	C11H11NO6	N-Pyruvoyl-5-methoxy-3-hydroxyanthranilate	HC3
9.81	14.12	397.23	-2.20	C18H37O7P	PA(15:0/0:0)	HC3
9.82	14.14	677.44	-1.39	C35H65O10P	PG(12:0/17:2(9Z,12Z))	HC11
9.82	14.15	703.46	1.73	C37H67O10P	PG(13:0/18:3(6Z,9Z,12Z))	HC11
9.82	14.07	344.28	2.86	C19H37NO4	Dodecanoylcarnitine	HC11,HC3,LC11
9.83	14.15	677.55	1.09	C38H77O7P	PA(O-18:0/17:0)	HC11
9.84	9.67	227.12	-1.29	C21H32N4O7	Ala-Leu-Ser-Tyr	HC3,LC3
9.85	14.21	463.19	2.32	C26H26N2O6	(+)-Plicamine	HC3,LC3
9.85	14.02	286.27	0.17	C17H35NO2	[SP (17:0)] heptadecasphing-4-enine	HC11,HC3,LC11,LC3
9.86	14.22	759.18	2.48	C35H34O19	Monepalin B	HC11,HC3,LC3
9.86	13.72	206.10	2.23	C20H30N2O5S	Benfuracarb	HC11,HC3,LC11,LC3
9.86	9.72	512.23	-1.98	C22H33N5O9	Gln-Thr-Thr-Tyr	LC11
9.87	11.64	374.20	0.45	C21H27NO5	(S)-autumnaline	LC11,LC3
9.88	7.51	240.11	1.70	C9H13N5O3	Dihydrobiopterin	HC3,LC11,LC3
9.88	14.26	797.55	-1.19	C41H81O12P	PI(O-18:0/14:0)	HC11
9.89	14.27	635.50	-1.97	C35H71O7P	PA(O-18:0/14:0)	HC11
9.89	14.01	374.25	1.58	C26H31NO	Androsta-5,16-dieno[17,16-b]quinolin-3beta-ol	HC11
9.89	14.28	645.45	0.48	C35H65O8P	PA(32:2)	HC11,HC3,LC3
9.89	11.73	377.16	0.15	C20H24O7	Ailanthone	HC3
9.90	14.29	232.08	-1.75	C23H26O10	[Fv hydroxy,dimethox] 4,2'-Dihydroxy-4',6'-dimethoxychalcone 4-glucoside	HC3,LC11,LC3
9.90	13.80	745.42	2.64	C70H124N2O31	Ganglioside GM1 (d18:1/14:0)	HC3,LC11,LC3

9.90	14.30	721.50	-1.85	C38H73O10P	PG(32:1)	HC11
9.90	13.90	188.07	0.22	C11H9NO2	Indoleacrylicacid	LC3
9.90	14.31	649.52	-1.37	C43H68O4	3-demethylubiquinol-7	HC11,HC3,LC11,LC3
9.91	13.57	330.26	0.64	C18H35NO4	4-8dimethylnonanoylcarnitine	HC11,HC3,LC11
9.91	14.31	659.50	-1.39	C44H66O4	ubiquinone-7	HC11
9.91	12.44	144.07	2.64	C6H9NO3	Vinylacetylglycine	HC3,LC11,LC3
9.91	9.91	838.17	2.39	C25H42N7O17P3S	2-Methylpropanoyl-CoA	HC3,LC11,LC3
9.93	14.31	379.25	2.84	C22H34O5	Pleuromutilin	HC11,HC3,LC11,LC3
9.93	14.36	679.26	-2.58	C33H42O15	Wanepimedoside A	HC11,HC3,LC11,LC3
9.93	12.24	186.11	-2.23	C9H15NO3	8-keto-7-aminoperlagonate	LC3
9.93	13.87	447.09	2.36	C21H18O11	Baicalin	HC3,LC11,LC3
9.94	14.37	555.29	-1.76	C32H42O8	Acrovestone	HC11
9.94	9.88	551.21	0.06	C27H30N6O5S	Cys-Trp-Trp-Gly	HC3
9.94	9.89	208.11	-2.61	C18H30N4O7	Ala-Leu-Asp-Pro	HC11,HC3,LC11,LC3
9.94	9.94	479.19	2.52	C19H30N2O12	glcNAc-1,6-anhMurNAc	HC3,LC11,LC3
9.94	13.63	272.26	-2.85	C16H33NO2	Undecanoylcholine	HC3,LC3
9.95	14.39	355.19	-2.61	C15H31O7P	PA(12:0/0:0)	HC3,LC11,LC3
9.95	9.88	244.15	-1.28	C12H21NO4	Tiglylcarnitine	HC3,LC11,LC3
9.95	10.11	244.12	-2.57	C12H13N5O	o-topolin	HC3,LC3
9.96	12.43	882.60	2.56	C52H84NO8P	PC(22:4(7Z,10Z,13Z,16Z)/22:6(4Z,7Z,10Z,13Z,16Z,19Z))	HC11
9.97	12.78	204.09	-2.06	C8H13NO5	N2-Acetyl-L-aminoadipate	HC11,HC3,LC11,LC3

9.97	14.43	727.21	-1.42	C32H38O19	Schaftoside 6''-O-glucoside	HC11
9.98	10.19	294.12	2.47	C11H19NO8	N-Acetyl-D-muramoate	HC3,LC11,LC3
9.98	10.50	96.05	2.44	C5H5NO	pyridine-N-oxide	HC3,LC11,LC3
9.99	14.48	617.42	-0.35	C33H61O8P	PA(30:2)	HC11
9.99	14.49	631.47	0.28	C42H62O4	3-heptaprenyl-4,5-dihydroxybenzoate	HC11
10.02	14.03	643.35	1.62	C29H55O13P	Variant-surface-glycoprotein 1,2-didecanoyl-sn-phosphatidylinositol	HC3
10.03	10.03	422.29	-0.17	C24H39NO5	Talatizamine	HC11,HC3,LC11,LC3
10.03	14.06	468.39	-1.46	C30H49N3O	Lucidine B	HC11,HC3,LC3
10.05	12.16	396.20	1.16	C19H29N3O4S	Met-Phe-Val	HC11
10.06	14.11	426.24	-0.32	C29H31NO2	Nafoxidine	HC11,HC3
10.06	10.13	470.17	-2.00	C19H27N5O7S	Ala-Asn-Cys-Tyr	LC3
10.08	11.32	376.22	-1.14	C20H29N3O4	Leu-Phe-Pro	HC11
10.08	12.46	262.22	-1.02	C17H27NO	Cryptophorine	HC11
10.08	13.25	322.22	2.65	C22H27NO	Phenazocine	HC11
10.10	14.54	345.24	-1.33	C22H32O3	[ST hydroxy(3:0)] (5Z,7E)-(1S,3R)-1,3-dihydroxy-9,10-seco-23,24-dinor-5,7,10(19)-cholatrien-22-one	HC11,LC11
10.10	10.10	615.40	-0.21	C32H58N2O7S	CHAPS	HC11
10.11	11.74	225.10	1.77	C9H12N4O3	Temurin	LC3
10.11	14.23	784.49	-0.23	C45H70NO8P	PE(18:4(6Z,9Z,12Z,15Z)/22:6(4Z,7Z,10Z,13Z,16Z,19Z))	HC11,HC3,LC11,LC3
10.12	14.24	593.38	-1.21	C38H48N4O2	(-)-Santiaguine	HC11,HC3,LC11,LC3
10.15	7.75	127.02	2.81	C2H7O4P	2-Hydroxyethylphosphonate	HC11,HC3,LC11,LC3

10.15	11.38	350.21	-2.06	C18H27N3O4	Leu-Phe-Ala	LC11
10.15	10.79	129.03	2.10	C4H4N2O3	Barbiturate	HC3
10.16	14.31	539.40	2.08	C37H50N2O	Methanophenazine	HC11
10.16	10.32	451.20	2.31	C19H26N6O7	Asn-Phe-Asn-Gly	HC11,LC3
10.16	14.32	664.46	2.07	C34H66NO9P	PS(P-16:0/12:0)	HC11,HC3,LC11,LC3
10.16	13.63	258.24	-0.24	C15H31NO2	Decanoylcholine	HC11,HC3,LC11
10.17	12.84	440.31	-1.65	C21H46NO6P	[PC methyl(12:2)] 1-dodecyl-2-methyl-sn-glycero-3-phosphocholine	HC11,HC3,LC11
10.17	9.84	194.12	-1.63	C11H15NO2	3,4-Methylenedioxymethamphetamine	LC11,LC3
10.18	13.85	314.27	-0.19	C18H35NO3	(+)-Prosopinine	HC3,LC11,LC3
10.18	11.54	230.15	0.66	C15H19NO	Pronethalol	HC11,HC3,LC11
10.18	13.58	244.23	-2.52	C14H29NO2	[SP (14:0)] tetradecasphing-4E-enine	HC3,LC11,LC3
10.20	14.23	316.25	0.39	C17H33NO4	[FA (10:0)] O-decanoyl-R-carnitine	HC3,LC11
10.21	5.67	184.17	1.21	C11H21NO	Tecostanine	HC3,LC11,LC3
10.21	10.43	544.35	-0.66	C29H45N5O5	Ile-Leu-Leu-Trp	HC11,HC3,LC3
10.22	10.43	550.21	1.07	C23H31N7O7S	Asn-Trp-Cys-Gln	HC11,LC3
10.22	14.10	183.08	1.73	C6H15O4P	triethyl phosphate	HC11,HC3,LC11,LC3
10.24	9.26	346.18	-1.93	C15H27N3O4S	Met-Val-Pro	HC11
10.24	14.27	371.07	0.23	C16H19O6PS	BPH-700	LC11,LC3
10.26	13.53	302.23	0.20	C16H31NO4	2-6dimethylheptanoylcarnitine	HC3,LC11,LC3
10.27	10.50	360.20	1.73	C16H29N3O4S	Leu-Met-Pro	HC11
10.29	14.58	483.17	-1.20	C27H28FN2OClS	SSR 125543	HC11,LC3
10.29	13.08	482.36	2.47	C24H52NO6P	[PC (16:2)] 1-hexadecyl-sn-glycero-3-phosphocholine	HC3,LC11
10.30	13.59	230.25	1.25	C14H31NO	[SP (14:0)] 1-deoxy-tetradecasphinganine	LC11,LC3
10.33	10.67	529.28	0.79	C26H36N6O6	Asn-Leu-Trp-Pro	HC11,HC3,LC11

10.36	5.99	156.10	-0.19	C8H13NO2	Arecoline	HC3,LC11,LC3
10.37	11.63	348.19	-2.48	C15H29N3O4S	Leu-Leu-Cys	HC11
10.37	9.02	142.09	2.50	C7H11NO2	L-Hypoglycin	HC3,LC11,LC3
10.41	12.41	362.21	1.65	C16H31N3O4S	Leu-Met-Val	HC11,HC3
10.41	6.58	156.08	-1.39	C6H9N3O2	3-(Pyrazol-1-yl)-L-alanine	LC3
10.42	15.06	274.27	-2.98	C16H35NO2	Hexadecasphinganine	HC11,HC3
10.44	14.64	230.21	-0.35	C13H27NO2	[FA amino(13:0)] 13-amino-tridecanoic acid	HC11,HC3,LC11
10.46	14.25	216.20	-1.67	C12H25NO2	[FA amino(12:0)] 12-amino-dodecanoic acid	HC3,LC11,LC3
10.48	14.96	847.23	1.99	C40H38N4O17	MesohydroxyuroporphyrinIII	LC11
10.49	10.97	485.15	-0.03	C20H28N4O6S2	Cys-Cys-Pro-Tyr	HC3,LC11
10.49	11.06	250.16	0.65	C18H19N	cis-N-Methyl-(S)-7,8,13,14-tetrahydroprotoberberine	HC11,HC3,LC11,LC3
10.52	12.40	236.14	1.92	C17H17N	(S)-7,8,13,14-Tetrahydroprotoberberine	HC11,HC3,LC11,LC3
10.52	8.66	208.17	1.79	C13H21NO	Luciduline	HC11,HC3,LC11,LC3
10.54	11.07	534.26	-0.86	C25H35N5O8	Asp-Leu-Thr-Trp	HC11,LC11,LC3
10.54	15.07	716.45	-2.53	C37H66NO10P	PS(13:0/18:3(6Z,9Z,12Z))	HC11
10.54	13.44	322.18	0.90	C21H23NO2	quinidone	HC11
10.55	11.87	222.13	1.87	C16H15N	Dizocilpine	HC11,HC3,LC11,LC3
10.55	14.84	149.06	0.70	C9H8O2	Dihydrocoumarin	HC11,HC3,LC11,LC3
10.56	12.06	242.19	1.10	C17H23N	N-Methyl morphinan	HC11,HC3,LC11,LC3
10.56	15.16	345.25	0.77	C21H32N2O2	16b-Hydroxystanozolol	HC11,HC3
10.58	13.66	244.21	1.81	C17H25N	Phencyclidine	HC11,HC3,LC11,LC3
10.58	13.55	154.05	0.46	C7H7NO3	3-Hydroxyanthranilate	LC11,LC3
10.58	15.16	690.43	-2.60	C35H64NO10P	PS(12:0/17:2(9Z,12Z))	HC11,HC3

10.59	13.32	288.25	-0.81	C16H33NO3	[SP hydroxy,hydroxy,methyl(10:2/2:0)] 6R-(8-hydroxydecyl)-2R-(hydroxymethyl)-piperidin-3R-ol	HC3,LC11,LC3
10.59	13.02	274.20	1.44	C14H27NO4	Heptanoylcarnitine	HC3,LC11,LC3
10.59	15.18	676.42	-1.24	C34H62NO10P	PS(14:1(9Z)/14:1(9Z))	HC11,HC3,LC3
10.62	6.81	362.16	1.04	C14H23N3O8	Leu-Asp-Asp	HC3,LC3
10.62	6.99	170.08	1.59	C8H11NO3	6-Hydroxydopamine	HC3,LC11,LC3
10.63	8.57	338.18	-0.02	C24H21N2	Benzyl viologen	HC3
10.64	14.89	176.07	-0.24	C10H9NO2	5-Hydroxyindoleacetaldehyde	HC3
10.64	15.29	447.25	2.28	C25H30N6O2	MC-207,110	HC11,HC3,LC11,LC3
10.65	9.88	135.09	-1.32	C8H10N2	2-phenylacetamidine	LC11,LC3
10.67	10.67	654.43	-0.07	C34H59N3O9	Enniatin B4	HC11,HC3
10.69	8.20	380.12	2.82	C14H21NO11	4-Deoxy-beta-D-gluc-4-enuronosyl-(1,3)-N-acetyl-D-galactosamine	HC3,LC11,LC3
10.69	15.46	316.28	-2.50	C18H37NO3	[SP hydrox] 6-hydroxysphing-4E-enine	HC11,LC11,LC3
10.70	13.90	384.25	1.28	C17H38NO6P	[PC methyl(8:2)] 1-octyl-2-methyl-sn-glycero-3-phosphocholine	HC11,HC3,LC11,LC3
10.70	9.58	269.08	1.74	C11H12N2O6	Portulacaxanthin III	HC3,LC3
10.70	13.76	319.24	0.22	C19H30N2O2	amo-1618	HC11,HC3,LC11
10.71	15.42	451.29	-1.30	C30H34N4	Usambarine	HC11
10.73	10.73	868.18	2.48	C26H44N7O18P3S	(2S,3S)-3-Hydroxy-2-methylbutanoyl-CoA	HC3,LC11,LC3
10.75	11.50	475.29	0.06	C25H38N4O5	Ile-Phe-Val-Pro	HC11,HC3
10.75	12.54	312.13	-1.53	C12H17N5O5	N2-N2-Dimethylguanosine	LC3
10.76	14.50	392.25	-1.59	C21H33N3O4	Leu-Leu-Phe	HC11,HC3
10.76	13.38	220.17	2.54	C14H21NO	Fabianine	HC11,HC3,LC11,LC3

10.77	13.57	238.16	1.28	C17H19N	N-Methyl-(R,S)-tetrahydrobenzylisoquinoline	HC11,HC3,LC11,LC3
10.77	11.53	457.25	0.52	C21H36N4O5S	Ile-Met-Pro-Pro	HC11
10.77	7.18	279.21	0.92	C16H26N2O2	Lycocernuine	HC11,HC3
10.79	15.57	506.35	1.39	C29H47NO6	Mycinamicin VIII	HC11,HC3,LC11,LC3
10.79	14.37	202.18	-0.57	C11H23NO2	[FA amino(11:0)] 11-amino-undecanoic acid	HC11,HC3,LC11
10.79	11.59	237.12	-2.97	C20H32N4O9	Glu-Leu-Asp-Pro	LC11
10.80	14.31	330.23	0.02	C17H31NO5	6-Keto-decanoylcarnitine	HC11,HC3,LC11
10.80	11.60	445.24	0.80	C18H32N6O7	Ala-Leu-Asn-Gln	HC11
10.81	10.10	196.06	1.44	C9H9NO4	Dopaquinone	LC3
10.82	11.64	264.10	-2.82	C22H30N4O11	Glu-Thr-Asp-Tyr	HC11,LC11
10.84	11.68	475.30	-0.60	C22H42N4O5S	Ile-Leu-Met-Val	HC11,HC3,LC11
10.85	11.38	232.17	2.13	C15H21NO	Metazocine	HC11,HC3,LC11,LC3
10.85	14.52	167.07	0.73	C6H14O3S	hexanesulfonate	HC11,HC3
10.86	12.76	268.13	0.95	C17H17NO2	(-)-Caaverine	HC11,HC3,LC11
10.87	11.73	459.28	0.64	C21H38N4O7	Asp-Leu-Leu-Val	HC11,HC3,LC3
10.88	11.76	443.23	-2.71	C20H34N4O5S	Met-Val-Pro-Pro	HC11
10.88	9.34	368.16	0.60	C14H25NO10	N-Acetyl-6-O-L-fucosyl-D-glucosamine	HC3,LC3
10.88	15.74	232.12	-1.61	C10H17NO5	Suberylglycine	LC3
10.89	15.78	638.40	-2.24	C31H60NO10P	PS(13:0/12:0)	HC11,HC3
10.89	13.65	224.14	1.08	C16H17N	(R,S)-Tetrahydrobenzylisoquinoline	HC11,HC3,LC11,LC3
10.90	15.33	204.18	1.88	C14H21N	N-(Cyclohexylmethyl)-N-methylbenzenamine	HC11,HC3,LC11,LC3
10.91	10.17	305.22	2.48	C18H28N2O2	RU 5135	HC11,HC3,LC11
10.91	10.91	484.05	-2.04	C13H26NO10PS3	Coenzyme M 7-mercaptoheptanoylthreonine-phosphate heterodisulfide	HC11
10.92	14.49	358.27	-1.75	C18H35N3O4	Leu-Leu-Leu	HC11,HC3

10.93	13.41	182.19	-0.66	C12H23N	Dicyclohexylamine	HC11,HC3,LC11,LC3
10.95	11.91	431.23	2.84	C19H34N4O5S	Ala-Leu-Met-Pro	HC11
11.00	15.36	194.10	1.10	C14H11N	2-Anthramine	HC11,HC3,LC11
11.06	6.51	270.12	2.39	C10H15N5O4	formycin A	LC11
11.07	8.58	256.10	-1.46	C9H13N5O4	2-Amino-4-hydroxy-6-(D-erythro-1,2,3-trihydroxypropyl)-7,8- dihydropteridine	LC11
11.10	11.10	721.32	-2.80	C29H48N6O15	1,6-anhydrous-N-Acetylmuramyl-tetrapeptide	LC11,LC3
11.10	11.10	291.21	1.87	C17H26N2O2	3'-Hydroxyropivacaine	HC11,HC3
11.10	14.37	352.17	1.15	C20H21N3O3	Phe-Trp	LC11
11.13	7.12	247.14	0.65	C14H18N2O2	Hypaphorine	HC11,HC3,LC11
11.15	16.10	323.20	1.45	C19H27FO3	12alpha-Fluoro-11beta,17beta-dihydroxyandrost-4-en-3-one	HC3,LC3
11.16	14.76	378.24	2.32	C20H31N3O4	Leu-Phe-Val	HC11,HC3,LC11
11.19	13.92	279.17	-0.40	C15H22N2O3	Leu-Phe	HC11,HC3
11.20	12.18	237.10	1.54	C12H14NO4	Salsoline-1-carboxylate	LC11
11.22	8.44	249.20	2.30	C15H24N2O	Aphylline	HC11,HC3,LC11
11.26	14.55	344.25	-0.98	C17H33N3O4	Leu-Leu-Val	HC3
11.27	15.68	183.09	2.20	C12H10N2	Harman	HC11,HC3
11.27	11.02	209.09	2.64	C11H13N2Cl	Epibatidine	LC11
11.28	15.55	62.93	-1.09	Cu	Cu+	LC11,LC3
11.29	12.57	497.20	-1.69	C20H28N6O9	Asn-Asn-Ser-Tyr	HC3,LC11,LC3
11.29	14.50	374.20	0.83	C21H27NO5	(S)-autumnaline	LC11,LC3
11.30	14.53	137.11	-0.87	C8H12N2	N,N-Dimethyl-1,4-phenylenediamine	HC11,HC3,LC11,LC3
11.31	12.62	253.14	-1.76	C25H36N4O7	Glu-Leu-Phe-Pro	LC11
11.33	14.47	130.16	2.34	C8H19N	Octylamine	HC11,HC3,LC11

11.33	7.01	241.13	0.90	C10H16N4O3	N(alpha)-gamma-L-Glutamylhistamine	HC11,HC3
11.35	11.58	263.14	-2.19	C14H18N2O3	Phe-Pro	HC11
11.35	11.61	276.16	0.28	C16H21NO3	Homatropine	HC3
11.35	10.27	332.16	-1.29	C14H25N3O4S	Leu-Cys-Pro	LC3
11.37	11.83	121.05	-0.16	C5H4N4	Purine	HC11,HC3,LC11,LC3
11.39	15.10	182.05	0.90	C8H7NO4	DIBOA	LC3
11.39	16.41	242.12	1.91	C15H15NO2	N-Methylflindersine	HC11,LC11,LC3
11.40	12.38	250.14	1.86	C14H19NO3	Ruspolinone	HC3
11.41	13.18	268.10	-1.26	C10H13N5O4	Deoxyguanosine	HC3,LC11
11.43	12.49	196.09	2.93	C11H14NCl	SK&F 86466	HC11,HC3
11.48	15.46	936.37	-1.37	C54H72N4O6Zn	Zn-bacteriochlorophyll a	HC3
11.50	11.29	166.12	1.62	C10H15NO	(+)-Pseudoephedrine	HC11,HC3,LC11,LC3
11.52	16.27	180.07	-1.99	C9H9NO3	Hippurate	HC11,HC3
11.53	13.05	441.31	-0.89	C22H40N4O5	Ile-Leu-Val-Pro	HC11,HC3,LC11,LC3
11.53	10.97	385.25	1.64	C18H32N4O5	Ala-Val-Val-Pro	HC3
11.54	12.45	382.14	2.35	C14H23NO11	N-Acetyl-9-O-lactoylneuraminicacid	HC3,LC3
11.54	13.67	342.24	-1.01	C17H31N3O4	Leu-Leu-Pro	HC11,HC3
11.55	13.09	608.28	-2.07	C30H37N7O7	Gln-Phe-Trp-Gln	HC11
11.57	13.13	497.24	1.75	C23H36N4O6S	Ala-Leu-Met-Tyr	LC3
11.59	14.65	176.14	1.79	C12H17N	Nigrifactin	HC11,HC3,LC11,LC3
11.61	11.83	238.14	2.03	C13H19NO3	Gigantine	HC3
11.62	15.15	255.15	1.00	C16H18N2O	Amphenone B	HC11,HC3,LC11
11.62	11.62	485.25	0.94	C18H36N4O11	Kanamycin A	LC3

11.64	13.27	511.26	-2.44	C24H38N4O6S	Cys-Leu-Leu-Tyr	HC11,HC3,LC11,LC3
11.64	15.99	242.21	-2.68	C14H27NO2	[SP (14:0/2:0)] tetradecasphinga-4E,6E-dienine	HC3,LC3
11.66	9.41	174.09	2.51	C11H11NO	Echinorine	HC11,HC3,LC3
11.67	13.34	433.25	2.71	C22H32N4O5	Ala-Phe-Val-Pro	HC11,HC3,LC11
11.68	13.36	463.29	-0.90	C24H38N4O5	Ala-Leu-Leu-Phe	LC3
11.72	11.72	886.18	-1.23	C26H46N7O19P3S	2'-(5''-phosphoribosyl)-3'-dephospho-CoA	LC11,LC3
11.72	13.45	461.24	2.30	C18H32N6O8	Asn-Leu-Gln-Ser	HC11,LC11,LC3
11.73	13.45	479.25	1.71	C23H34N4O7	Asp-Phe-Val-Val	HC11,HC3,LC11
11.78	16.38	227.18	-0.16	C12H22N2O2	1,8-Diazacyclotetradecane-2,9-dione	HC11,HC3,LC11,LC3
11.79	12.89	137.05	1.67	C5H4N4O	allopurinol	LC3
11.81	13.61	469.21	0.12	C24H28N4O6	Thr-Trp-Tyr	HC11,LC11,LC3
11.82	12.01	164.11	2.69	C10H13NO	(R)-2-Methylimino-1-phenylpropan-1-ol	HC11,HC3,LC3
11.83	12.74	174.13	-0.02	C12H15N	1-Methyl-4-phenyl-1,2,3,6-tetrahydropyridine	HC11,HC3,LC11,LC3
11.85	15.65	169.08	-0.57	C11H8N2	beta-Carboline	HC11,HC3,LC11,LC3
11.87	16.41	190.12	1.35	C12H15NO	1-Methyl-4-phenyl-1,2,3,6-tetrahydropyridine N-oxide	HC11,HC3,LC11,LC3
11.89	8.46	234.10	-1.10	C10H11N5O2	Dihydroxycoprostanoicacid	HC3,LC11
11.91	8.81	402.16	-1.97	C15H23N5O8	Asn-Asp-Gly-Pro	HC11,LC11,LC3
11.91	13.82	408.25	-2.58	C21H33N3O5	Leu-leu-tyr	LC11,LC3
11.91	14.57	265.16	2.48	C14H20N2O3	Phe-Val	HC11,HC3,LC11,LC3
11.92	13.85	490.27	0.38	C24H35N5O6	Ala-Leu-Thr-Trp	HC11,HC3,LC11,LC3
11.94	13.02	298.12	1.13	C11H15N5O5	1-methylguanosine	LC11
11.96	13.53	286.18	-0.09	C18H23NO2	Isococculidine	HC11,HC3,LC3

11.96	9.80	116.07	1.05	C5H9NO2	D-Proline	HC11,HC3,LC11,LC3
11.96	13.83	231.17	1.52	C11H22N2O3	Leu-Val	HC11,HC3
11.97	14.13	336.19	1.69	C17H25N3O4	Leu-Phe-Gly	HC11,HC3,LC11,LC3
11.98	14.62	392.22	-0.71	C20H29N3O5	Leu-Pro-Tyr	HC11,HC3,LC11,LC3
11.98	10.54	198.11	0.56	C10H15NO3	L-Metanephrine	HC3
11.99	10.71	362.16	2.61	C14H23N3O8	Glu-Asp-Val	HC3,LC3
12.00	13.35	128.14	1.00	C8H17N	Coniine	HC11,HC3,LC11,LC3
12.00	6.92	138.06	0.71	C7H7NO2	N-Methylnicotinate	HC11,HC3,LC11,LC3
12.00	11.61	208.17	-0.22	C13H21NO	Luciduline	HC11,HC3,LC11,LC3
12.00	7.96	222.10	0.26	C9H11N5O2	a pyrimidodiazepine	HC11,HC3,LC11,LC3
12.01	12.01	69.03	2.65	C4H4O	3-Butyn-1-al	HC3,LC11,LC3
12.01	16.88	204.09	1.40	C8H13NO5	N2-Acetyl-L-aminoadipate	HC11,HC3,LC11,LC3
12.02	16.27	96.05	1.45	C5H5NO	2-Hydroxypyridine	HC3,LC11,LC3
12.02	14.05	574.24	2.68	C27H35N5O7S	Asn-Met-Phe-Tyr	HC11
12.02	14.34	124.04	-0.03	C6H5NO2	Picolinic acid	HC3,LC11,LC3
12.02	14.05	523.28	-1.69	C25H38N4O8	Asp-Leu-Leu-Tyr	HC3,LC11,LC3
12.03	12.03	496.10	0.31	C15H23N5O10P2	Isopnetenyladenosine-5'-diphosphate	HC3,LC3
12.04	15.76	257.02	2.17	C10H9O6P	4-methyl-umbelliferyl phosphate	HC3
12.05	14.10	509.26	1.44	C24H36N4O8	Asp-Leu-Val-Tyr	HC11,HC3,LC11,LC3
12.05	14.11	550.21	-1.55	C24H31N5O10	Glu-Thr-Trp-Asp	HC11
12.06	17.02	145.09	-2.86	C7H12O3	trans-4-Hydroxycyclohexanecarboxylate	HC11,HC3,LC3
12.06	11.28	114.06	2.89	C5H7NO2	(S)-1-Pyrroline-5-carboxylate	HC11,HC3,LC11,LC3
12.08	9.15	406.15	-2.46	C15H23N3O10	Glu-Glu-Glu	LC11,LC3
12.08	14.14	244.15	0.42	C12H21NO4	Tiglylcarnitine	HC11,HC3,LC11,LC3

12.08	12.08	429.17	-2.69	C20H28O10	Furcatin	LC3
12.09	12.09	445.21	0.09	C21H32O10	dihydrophaseic acid 4-O-beta-D-glucoside	LC3
12.09	14.47	246.15	0.79	C15H19NO2	Benzoyltropein	HC11,HC3,LC3
12.10	12.36	166.09	0.19	C9H11NO2	L-Phenylalanine	HC11,HC3,LC3
12.11	14.22	432.21	-1.72	C17H29N5O8	Ala-Leu-Asn-Asp	LC3
12.11	16.73	384.22	0.84	C16H34NO7P	[PC acetyl(6:2)] 1-hexyl-2-acetyl-sn-glycero-3-phosphocholine	HC11,HC3
12.14	14.29	419.23	-2.84	C18H34N4O5S	Ala-Leu-Leu-Cys	HC11,HC3
12.16	14.13	281.10	0.37	C10H20N2O3S2	Met-Met	HC3
12.17	14.33	221.08	-0.67	C18H24N4O9	Asp-Gly-Ser-Tyr	HC11
12.17	15.02	208.13	-0.23	C12H17NO2	3-(Dimethylamino)propyl benzoate	HC11,HC3,LC3
12.18	14.36	495.25	2.92	C23H34N4O8	Asp-Leu-Phe-Thr	HC11,HC3,LC11,LC3
12.19	14.38	462.26	1.01	C19H35N5O8	Gln-Leu-Thr-Thr	HC11,HC3,LC11,LC3
12.20	14.03	328.22	-1.02	C16H29N3O4	Ile-Pro-Val	HC11,HC3,LC11,LC3
12.20	14.41	448.22	-1.71	C21H29N5O6	Ala-Phe-Asn-Pro	LC3
12.21	14.43	540.24	-2.78	C27H33N5O7	Ala-Thr-Trp-Tyr	HC3
12.21	14.30	210.11	0.63	C11H15NO3	Tyr-OEt	HC11,HC3
12.23	14.46	463.25	-2.14	C23H34N4O6	Ala-Leu-Pro-Tyr	HC11,HC3,LC11,LC3
12.24	14.48	437.24	-2.52	C21H32N4O6	Ala-Leu-Ala-Tyr	HC11,HC3,LC11,LC3
12.24	12.24	464.24	0.10	C26H31N4O4	ketoconazol	LC11
12.26	14.52	445.27	-1.86	C20H36N4O7	Asp-Leu-Val-Val	HC11,HC3,LC11,LC3

12.26	9.54	375.22	-2.19	C16H30N4O6	Ala-Leu-Thr-Ala	HC3
12.27	7.72	266.09	-0.74	C10H11N5O4	5'-Dehydroadenosine	HC3,LC11,LC3
12.30	14.60	410.19	-1.39	C19H27N3O7	Leu-Asp-Tyr	HC11,HC3,LC11,LC3
12.31	13.57	346.20	2.17	C15H27N3O6	Leu-Val-Asp	HC11,HC3,LC3
12.31	15.22	166.07	-1.01	C6H7N5O	3-Methylguanine	HC3
12.32	14.09	378.17	0.25	C15H27N3O6S	Leu-Met-Asp	HC11,HC3,LC3
12.32	9.45	215.12	1.51	C13H14N2O	Harmaline	HC11,HC3,LC11,LC3
12.32	12.32	378.07	1.38	C13H18N2O7PS	external aldimine	LC11
12.35	14.19	302.21	-0.19	C14H27N3O4	Leu-Leu-Gly	HC11,HC3,LC11,LC3
12.38	14.91	360.21	1.33	C16H29N3O6	Leu-Leu-Asp	HC11,HC3,LC3
12.38	17.38	148.11	1.37	C10H13N	Actinidine	HC11,HC3,LC3
12.38	14.14	126.13	0.80	C8H15N	gamma-Coniceine	HC11,HC3,LC11,LC3
12.39	12.97	288.19	-1.23	C13H25N3O4	Leu-Val-Gly	HC3
12.39	13.08	279.10	-0.83	C10H18N2O5S	Glu-Met	LC11
12.40	14.79	547.29	2.20	C26H38N6O7	Gln-Leu-Thr-Trp	HC11
12.41	14.81	417.24	2.15	C18H32N4O7	Ala-Leu-Val-Asp	HC11,HC3
12.41	14.14	140.14	-2.26	C9H17N	Pinidine	HC11,HC3,LC11,LC3
12.42	14.65	380.18	-1.16	C18H25N3O6	Phe-Val-Asp	HC11,HC3,LC3
12.42	16.07	133.08	-2.00	C8H8N2	Indoleamine	HC11,HC3,LC11,LC3
12.42	14.85	521.26	-0.11	C25H36N4O8	Glu-Leu-Pro-Tyr	HC11,HC3,LC11,LC3
12.44	12.44	455.25	-0.70	C20H38O11	octyl β-1,6-D-galactofuranosyl-α-D-glucopyranoside	HC11,HC3,LC11,LC3

12.45	11.63	391.20	-0.13	C19H26N4O5	Phe-Gln-Pro	HC11,HC3
12.45	11.45	276.11	-0.30	C12H13N5O3	Ethenodeoxyadenosine	HC11,HC3,LC11,LC3
12.47	14.94	231.13	2.33	C20H36N4O8	Asp-Leu-Leu-Thr	HC11,HC3,LC3
12.48	9.73	217.13	-0.32	C8H16N4O3	N-acetyl-(L)-arginine	HC3,LC11
12.51	14.64	392.18	-0.05	C19H25N3O6	Glu-Phe-Pro	LC3
12.53	14.93	396.21	0.86	C19H29N3O6	Leu-Thr-Tyr	HC11,HC3,LC11,LC3
12.53	15.65	130.05	1.91	C5H7NO3	L-1-Pyrroline-3-hydroxy-5-carboxylate	HC11,HC3,LC3
12.54	15.08	431.25	-0.63	C19H34N4O7	Ala-Leu-Leu-Asp	HC11,HC3,LC11,LC3
12.54	14.11	382.20	0.49	C18H27N3O6	Leu-Ser-Tyr	HC11,HC3,LC3
12.54	12.15	212.16	-2.76	C12H21NO2	Elaeokanine C	HC11,HC3,LC3
12.54	12.92	168.10	1.77	C9H13NO2	3-Methoxytyramine	HC11,HC3
12.56	15.12	212.10	-1.22	C19H26N4O7	Ala-Phe-Ala-Asp	HC11,HC3,LC11
12.56	14.50	382.14	2.54	C14H23NO11	N-Acetyl-9-O-lactoylneuraminicacid	HC3,LC3
12.58	12.58	526.29	1.82	C24H39N5O8	Desmosine	HC11,HC3,LC11
12.59	15.17	470.30	-2.74	C22H39N5O6	Gln-Leu-Leu-Pro	HC11,HC3,LC11,LC3
12.59	13.09	267.13	1.55	C13H18N2O4	Phe-Thr	HC11,HC3,LC11
12.60	15.21	250.13	-2.49	C26H34N4O6	Ala-Phe-Val-Tyr	HC11
12.64	13.97	320.16	-1.58	C13H25N3O4S	Leu-Met-Gly	HC11,HC3,LC11,LC3
12.64	10.71	304.19	1.36	C13H25N3O5	Leu-Thr-Ala	HC3
12.65	12.34	296.14	-1.81	C12H17N5O4	N6,N6-Dimethyladenosine	HC3
12.66	15.31	481.23	2.49	C22H32N4O8	Ala-Leu-Asp-Tyr	HC11,HC3,LC11,LC3

12.67	15.33	401.24	-1.33	C18H32N4O6	Ala-Leu-Thr-Pro	HC11
12.67	12.87	323.06	0.34	C10H15N2O8P	dTMP	HC3
12.67	16.45	392.18	-2.33	C16H29N3O6S	Glu-Ile-Met	HC11,HC3,LC3
12.68	10.73	142.12	-0.19	C8H15NO	Hygrine	HC11,HC3,LC11,LC3
12.69	11.51	215.14	2.15	C10H18N2O3	Val-Pro	HC11,HC3,LC3
12.70	15.41	405.22	2.06	C17H32N4O5S	Ala-Leu-Met-Ala	HC11,HC3,LC11,LC3
12.71	15.43	489.15	-2.03	C19H28N4O7S2	Cys-Thr-Cys-Tyr	HC11,HC3,LC11,LC3
12.72	10.05	184.13	-1.42	C10H17NO2	Acetylpseudotropine	HC11,HC3,LC11,LC3
12.72	15.23	224.16	-1.34	C13H21NO2	Tigloidine	HC3,LC11
12.73	12.93	276.14	-1.27	C12H21NO6	Glutarylcarnitine	HC11,HC3,LC11,LC3
12.73	15.46	403.22	0.16	C17H30N4O7	Ala-Val-Val-Asp	HC11,HC3
12.73	14.51	153.10	1.09	C8H12N2O	4-(hydroxylamino)-N,N-dimethylaniline	HC11,HC3,LC11,LC3
12.74	11.05	170.15	0.54	C10H19NO	Nitramine	HC11,HC3,LC11,LC3
12.74	15.85	281.15	1.53	C14H20N2O4	Val-Tyr	HC11,HC3,LC11,LC3
12.75	12.72	352.15	2.67	C16H21N3O6	Phe-Ala-Asp	HC11,HC3
12.76	18.39	113.02	1.22	C5H4O3	2-Furoate	HC3,LC11
12.77	17.33	106.07	1.91	C7H7N	4-vinylpyridine	HC11,HC3
12.78	9.68	316.19	2.66	C14H25N3O5	Leu-Pro-Ser	HC3,LC3
12.78	15.57	403.26	-0.85	C18H34N4O6	Ala-Leu-Leu-Ser	HC11,HC3,LC3
12.79	14.67	121.05	-0.44	C5H4N4	Purine	HC11,HC3,LC11,LC3
12.79	14.80	396.18	2.04	C18H25N3O7	Val-Asp-Tyr	HC11,HC3
12.79	18.53	335.15	2.46	C14H26N2O3S2	Lipoyllysine	HC3,LC3
12.80	7.26	172.06	1.93	C7H9NO4	AminoDHS	HC3

12.80	17.60	217.13	-1.83	C13H16N2O	Girgensonine	LC11
12.80	11.23	144.10	0.91	C7H13NO2	Stachydrine	HC11,HC3,LC11
12.80	18.08	183.08	0.62	C6H15O4P	Diisopropyl phosphate	HC11,HC3,LC11,LC3
12.82	11.20	146.08	1.30	C6H11NO3	[FA oxo,amino(6:0)] 3-oxo-5S-amino-hexanoic acid	HC11,HC3,LC3
12.83	13.47	218.14	2.57	C10H19NO4	O-Propanoylcarnitine	HC11,HC3,LC11,LC3
12.83	13.89	150.10	1.48	C8H11N3	benzylguanidine	HC3,LC11
12.83	9.83	216.12	0.63	C10H17NO4	4-(1-methyl-5-hydroxy-2-pyrrolidinyl)-3-oxobutanoate methyl ester	HC3,LC11,LC3
12.90	15.16	248.20	0.50	C16H25NO	Lycopodine	HC3
12.91	15.82	401.22	-2.32	C21H28N4O4	Trp-Val-Pro	LC3
12.91	15.83	494.26	0.44	C23H35N5O7	Ala-Leu-Gln-Tyr	HC11,LC3
12.92	15.84	538.25	0.29	C24H35N5O9	Asp-Leu-Gln-Tyr	HC11,HC3,LC11,LC3
12.94	15.87	504.22	2.60	C22H29N7O7	Ala-Trp-Asn-Asn	HC11,HC3,LC11
12.94	15.88	577.23	2.00	C27H36N4O8S	Met-Thr-Tyr-Tyr	LC11,LC3
12.98	15.95	572.22	0.06	C27H33N5O7S	Cys-Thr-Trp-Tyr	HC11,LC11
12.98	8.72	272.16	2.77	C12H21N3O4	Val-Gly-Pro	HC3,LC11,LC3
12.98	12.70	156.14	0.26	C9H17NO	N-Methylpelletierine	HC11,HC3,LC11,LC3
12.99	14.39	214.11	0.66	C10H15NO4	Kainic acid	HC3,LC3
13.01	18.52	754.58	2.18	C43H80NO7P	[PC (15:2/20:4)] 1-pentadecyl-2-(5Z,8Z,11Z,14Z-eicosatetraenoyl)-sn-glycero-3-phosphocholine	HC3
13.04	13.04	642.31	-0.16	C30H47N3O10S	S-(9-hydroxy-PGA2)-glutathione	LC11
13.07	10.43	203.10	1.47	C8H14N2O4	Coprine	HC3,LC3
13.08	8.31	130.12	-2.00	C7H15NO	4-Trimethylammoniobutanal	HC11,HC3,LC11,LC3
13.09	15.03	358.20	2.82	C16H27N3O6	Glu-Ile-Pro	HC11,HC3,LC11,LC3
13.09	18.93	152.07	-0.31	C8H9NO2	(R)-Mandelamide	HC11,HC3,LC11,LC3
13.10	13.92	220.08	-0.17	C8H13NO6	O-Succinyl-L-homoserine	HC11,HC3,LC11,LC3

13.12	11.23	431.18	- 2.08	C17H26N4O9	Glu-Ala-Asp-Pro	HC3
13.12	14.81	283.22	1.56	C19H26N2	(-)-Quebrachamine	HC3,LC11,LC3
13.13	11.30	286.18	- 1.25	C13H23N3O4	Leu-Gly-Pro	HC11,HC3,LC11,LC3
13.13	11.91	154.12	- 0.14	C9H15NO	Pseudopelletierine	HC3,LC3
13.14	15.33	166.12	1.37	C10H15NO	Hordenine	HC11,HC3,LC11,LC3
13.15	13.15	920.22	0.45	C42H45CoN4O16	cobalt-precorrin-2	LC11,LC3
13.15	15.67	290.18	- 1.57	C17H23NO3	L-Hyoscyamine	HC3,LC3
13.18	12.51	144.14	- 1.61	C8H17NO	Butyro-betaine	HC11,HC3,LC11,LC3
13.21	16.41	552.27	0.38	C25H37N5O9	Glu-Leu-Gln-Tyr	HC11,LC3
13.23	15.87	176.09	- 2.24	C7H13NO4	alpha-aminopimelate	HC11,LC11
13.24	8.75	320.18	2.97	C13H25N3O6	Leu-Thr-Ser	HC3,LC3
13.24	16.48	518.24	2.62	C23H31N7O7	Ala-Trp-Asn-Gln	LC11
13.24	16.02	399.11	1.61	C14H22O13	alginate	LC11,LC3
13.25	11.51	535.19	- 1.26	C20H30N4O13	Glu-Glu-Glu-Glu	HC11,LC11
13.26	16.52	449.20	2.14	C21H28N4O7	Ala-Phe-Asp-Pro	HC11,LC11,LC3
13.27	15.24	196.10	2.25	C10H13NO3	L-Tyrosine methyl ester	HC3,LC11,LC3
13.27	12.37	198.11	2.12	C10H15NO3	2-carbomethoxy-3-tropinone	HC3,LC3
13.27	13.85	312.20	- 0.09	C20H25NO2	Spiradine A	HC3,LC3
13.31	17.41	174.13	- 0.43	C12H15N	Norselegiline	HC11,HC3,LC11,LC3
13.32	13.32	736.25	0.51	C34H41NO17	Anthemis glycoside B	HC3,LC3
13.32	15.11	291.12	- 0.70	C11H18N2O7	N-Succinyl-LL-2,6-diaminoheptanedioate	HC11,LC11
13.34	9.44	325.10	0.41	C14H16N2O7	Portulacaxanthin I	LC11
13.34	15.97	206.10	1.90	C8H15NO5	N-Acetyl-D-fucosamine	HC11,HC3,LC3

13.35	13.30	233.15	1.84	C10H20N2O4	Leu-Thr	HC11,HC3,LC3
13.36	10.08	120.07	-0.62	C4H9NO3	L-Allothreonine	HC3,LC3
13.37	11.57	146.12	-1.25	C7H15NO2	3-Dehydroxycarnitine	HC11,HC3,LC11,LC3
13.38	15.18	230.14	2.51	C11H19NO4	Butenylcarnitine	HC3
13.40	19.29	440.28	-2.06	C20H42NO7P	[PC acetyl(10:2)] 1-decyl-2-acetyl-sn-glycero-3-phosphocholine	HC3,LC11,LC3
13.43	13.43	292.18	-0.23	C27H46N6O8	N1-(3,4-dihydroxybenzoyl)-N8,N'8-citryl-bis(spermidine)	HC11,LC11,LC3
13.47	13.47	496.10	-2.83	C15H23N5O10P2	Isopnetenyladenosine-5'-diphosphate	HC3
13.48	14.42	127.02	0.69	C2H7O4P	2-Hydroxyethylphosphonate	HC11,HC3,LC11,LC3
13.49	15.80	370.14	2.36	C16H23N3O5S	Met-Gly-Tyr	HC11
13.52	19.55	878.57	-2.68	C52H80NO8P	[PC (22:6/22:6)] 1,2-di-(4Z,7Z,10Z,13Z,16Z,19Z-docosahexaenoyl)-sn-glycero-3-phosphocholine	HC11
13.53	10.61	343.20	0.14	C15H26N4O5	Leu-Asn-Pro	HC3
13.62	10.84	387.22	1.70	C17H30N4O6	Ala-Leu-Pro-Ser	HC11,HC3,LC11
13.67	7.71	277.14	1.94	C11H20N2O6	N6-(L-1,3-Dicarboxypropyl)-L-lysine	HC11,HC3
13.67	17.34	503.16	-2.01	C20H30N4O7S2	Cys-Met-Ser-Tyr	LC11
13.70	17.39	475.13	-1.16	C18H26N4O7S2	Cys-Cys-Ser-Tyr	HC3,LC11,LC3
13.72	17.52	139.05	0.39	C6H6N2O2	Urocanate	LC11
13.72	17.43	396.21	2.97	C19H29N3O6	Ile-Thr-Tyr	HC3,LC11,LC3
13.76	14.44	324.16	2.98	C15H21N3O5	Ala-Ala-Tyr	HC11,LC11
13.77	14.84	268.13	2.83	C12H17N3O4	Agaritine	LC11
13.81	15.11	132.10	1.13	C6H13NO2	L-Leucine	HC11,HC3,LC11,LC3
13.82	13.82	637.41	-0.34	C29H52N10O6	Argiotoxin 636	HC11
13.85	14.47	259.09	2.06	C10H14N2O6	(1-Ribosylimidazole)-4-acetate	HC3,LC11,LC3
13.86	18.91	318.21	-2.96	C19H27NO3	Protoemetine	HC3,LC3

13.88	11.78	263.09	2.53	C9H14N2O7	Glu-Asp	HC3,LC11,LC3
13.91	12.81	406.15	0.41	C15H23N3O10	Glu-Glu-Glu	LC11
13.95	19.84	160.13	1.22	C8H17NO2	DL-2-Aminooctanoicacid	HC11,HC3,LC11,LC3
13.96	17.92	286.12	-2.53	C28H34N4O9	Glu-Pro-Tyr-Tyr	LC3
14.00	18.01	234.10	-1.62	C19H26N6O8	Asn-Asn-Gly-Tyr	HC11,HC3,LC11,LC3
14.10	18.20	540.25	-1.91	C24H37N5O7S	Asn-Leu-Met-Tyr	HC11,HC3
14.10	18.21	489.15	0.25	C19H28N4O7S2	Cys-Thr-Cys-Tyr	HC11,LC11
14.11	18.22	431.29	-0.77	C20H38N4O6	Ile-Leu-Val-Ser	HC11
14.12	19.80	116.03	1.62	C4H5NO3	Maleamate	LC11
14.12	20.44	386.31	-1.70	C25H39NO2	3beta-(1-Pyrrolidinyl)-5alpha-pregnane-11,20-dione	HC11
14.13	14.13	631.15	-0.81	C20H31N4O17P	CMP-N-glycoloylneuraminate	HC11
14.15	18.30	514.20	0.07	C21H31N5O8S	Asn-Met-Ser-Tyr	HC11,LC11
14.15	20.36	125.07	1.13	C6H8N2O	N-Propanoylimidazole	HC11,HC3,LC11,LC3
14.19	18.38	502.14	-0.40	C19H27N5O7S2	Asn-Cys-Cys-Tyr	LC11
14.20	19.59	360.24	-2.61	C19H29N5O2	Loxtidine	HC11
14.20	14.20	590.15	1.74	C19H33N3O14P2	dTDP-4-dimethylamino-4,6-dideoxy-5-C-methyl-L-mannose	HC11
14.20	17.66	232.15	-2.01	C11H21NO4	O-Butanoylcarnitine	HC3,LC11,LC3
14.22	15.13	274.18	2.59	C12H23N3O4	Leu-Ala-Ala	HC11
14.22	8.47	302.14	2.29	C12H19N3O6	Ala-Asp-Pro	HC3,LC3
14.24	10.05	378.12	1.63	C13H19N3O10	Glu-Asp-Asp	HC3,LC11,LC3
14.24	13.48	507.16	0.42	C18H26N4O13	Asp-Glu-Asp-Glu	HC11,HC3,LC11,LC3
14.24	14.49	318.17	-1.36	C13H23N3O6	Leu-Ala-Asp	LC11

14.25	15.37	273.20	1.16	C17H24N2O	beta-Obscurine	HC11
14.26	8.05	201.16	-1.47	C10H20N2O2	dimethylsuberimidate	HC11,HC3,LC11,LC3
14.28	11.96	392.13	1.69	C14H21N3O10	Glu-Asp-Glu	HC11,LC11
14.29	13.58	521.17	0.59	C19H28N4O13	Asp-Glu-Glu-Glu	LC11
14.31	16.02	162.08	0.93	C6H11NO4	N-Methyl-L-glutamate	HC11,HC3,LC11,LC3
14.35	20.05	279.11	2.84	C11H18O8	Tuliposide A	LC11
14.36	14.36	634.22	0.04	C23H39NO19	3'-Sialyllactose	LC11
14.36	15.54	380.12	1.34	C14H21NO11	3-(4-Deoxy-beta-D-gluc-4-enuronosyl)-N-acetyl-D-glucosamine	HC3,LC11,LC3
14.37	20.15	142.05	1.92	C6H7NO3	Gentianaine	HC3,LC11,LC3
14.38	14.38	901.59	1.77	C56H76N4O6	Pheophytins	HC11
14.39	14.39	903.60	1.96	C56H78N4O6	Bacterio-pheophytins	HC11
14.40	20.07	174.15	1.17	C9H19NO2	[FA amino(9:0)] 9-amino-nonanoic acid	HC11,HC3,LC11,LC3
14.43	18.86	509.21	-0.14	C20H36N4O7S2	Asp-Leu-Met-Met	LC11
14.44	8.33	377.20	1.29	C15H28N4O7	Ala-Leu-Ser-Ser	HC3
14.45	16.37	217.13	2.77	C13H16N2O	Tetrahydroharmine	LC11
14.45	18.90	491.20	1.56	C19H30N4O11	Glu-Leu-Asp-Asp	LC11
14.48	18.96	512.20	1.31	C21H29N5O10	Asn-Thr-Asp-Tyr	LC11
14.53	11.93	175.11	-2.31	C7H14N2O3	N-Acetylornithine	LC11
14.53	19.07	433.19	2.12	C17H28N4O9	Ala-Leu-Asp-Asp	LC11
14.54	8.52	140.08	0.04	C6H9N3O	L-Histidinal	LC11
14.55	14.55	736.25	1.83	C34H41NO17	Anthemis glycoside B	HC11,LC3
14.57	13.26	148.06	0.82	C5H9NO4	L-Glutamate	HC11,HC3,LC11,LC3
14.63	14.18	381.11	-2.53	C12H20N4O8S	S-(N-Hydroxy-N-methylcarbamoyl)glutathione	HC11,LC11,LC3
14.67	9.14	116.11	2.49	C6H13NO	Trimethylaminoacetone	HC11,HC3,LC11,LC3
14.75	8.51	445.22	2.90	C21H28N6O5	Ala-Phe-Ala-His	LC11
14.78	13.96	152.06	1.72	C5H5N5O	Guanine	HC11,HC3,LC11,LC3
14.78	17.21	204.09	1.67	C8H13NO5	2-acetamidoglucal	HC11,HC3,LC11,LC3

14.79	21.41	228.05	1.86	C9H9NO6	5-(2'-Carboxyethyl)-4,6-dihydroxypicolinate	LC11
14.79	18.96	126.13	1.49	C8H15N	gamma-Coniceine	HC11,HC3,LC11,LC3
14.83	8.67	454.25	-1.17	C18H31N9O5	Ala-Ala-Arg-His	LC11
14.84	15.04	143.08	1.67	C6H10N2O2	Ectoine	LC11,LC3
14.84	8.69	468.22	1.71	C19H29N7O7	Asn-Thr-Pro-His	LC11
14.86	19.72	492.24	-1.73	C23H33N5O7	Asn-Val-Pro-Tyr	LC11
14.90	14.90	565.24	0.42	C22H36N4O13	Aerobactin	LC11
14.93	16.76	289.23	0.02	C18H28N2O	Flabellidine	HC3,LC11,LC3
14.93	14.93	454.28	2.91	C24H39NO7	Delcosine	LC11
14.94	8.88	448.23	0.14	C17H33N7O5S	Ala-Met-Ala-Arg	HC11,HC3,LC11,LC3
14.99	8.98	519.33	0.24	C26H42N6O5	Lys-Lys-Phe-Pro	HC3,LC3
15.00	20.00	508.24	0.99	C23H33N5O8	Asn-Leu-Phe-Asp	LC11
15.01	9.14	118.12	2.44	C6H15NO	2-Methylcholine	LC11
15.04	15.09	493.14	1.17	C17H24N4O13	Glu-Asp-Asp-Asp	HC3,LC11,LC3
15.05	9.93	193.17	2.23	C12H20N2	N, N1-bis-(buta-2,3-dienyl)-1,4-diaminobutane	HC11,HC3,LC11,LC3
15.11	15.11	490.18	1.46	C14H30N6O11P	O-1,4-alpha-L-Dihydrostreptosyl-streptidine 6-phosphate	LC11
15.13	17.42	130.09	2.26	C6H11NO2	L-Pipecolate	HC11,HC3,LC11,LC3
15.16	18.89	135.09	2.77	C8H10N2	2-phenylacetamidine	LC11
15.17	9.34	401.18	-0.13	C15H24N6O7	Ala-Ser-Ser-His	LC11
15.22	9.44	446.22	0.26	C17H31N7O5S	Ala-Cys-Pro-Arg	HC11
15.31	12.19	378.12	1.84	C13H19N3O10	Glu-Asp-Asp	HC3,LC11,LC3
15.38	20.32	168.18	1.28	C11H21N	Mecamylamine	HC11,HC3,LC3
15.39	15.77	432.17	0.32	C16H25N5O9	Asn-Asp-Pro-Ser	LC11,LC3
15.40	9.79	432.20	-2.77	C16H29N7O5S	Arg-Cys-Gly-Pro	LC11,LC3
15.52	22.01	123.06	0.65	C6H6N2O	Isonicotineamide	LC11,LC3
15.68	15.68	476.12	-2.96	C15H21N7O9S	5'-O-[N-(L-glutamyl)-sulfamoyl] adenosine	HC11,LC3
15.75	15.54	130.10	2.30	C5H11N3O	4-Guanidinobutanal	LC11,LC3

15.80	20.77	374.20	1.72	C21H27NO5	Hasubanonine	HC11,HC3,LC11,LC3
15.82	14.55	249.11	2.81	C9H16N2O6	Glu-Thr	LC11,LC3
16.00	19.65	204.09	0.91	C8H13NO5	2-acetamidoglucal	HC11,HC3,LC11,LC3
16.01	16.01	484.10	-1.01	C15H21N3O15	Karakin	HC11,HC3,LC11,LC3
16.07	20.49	305.22	1.84	C18H28N2O2	RU 5135	HC11,HC3,LC11,LC3
16.08	22.16	441.31	1.83	C22H40N4O5	Leu-Leu-Val-Pro	HC3,LC11
16.10	15.53	134.05	1.80	C4H7NO4	L-Aspartate	HC11,HC3,LC11,LC3
16.11	22.22	474.24	1.73	C23H31N5O6	Ala-Thr-Trp-Pro	HC3
16.12	21.87	394.24	0.59	C21H27N7O	Oxidized Cypridina luciferin	HC3,LC3
16.12	14.73	198.12	1.42	C9H15N3O2	Hercynine	LC11,LC3
16.15	10.79	377.13	-2.46	C12H21N6O6P	puromycin aminonucleoside 5'-monophosphate	HC3,LC11,LC3
16.15	12.67	191.07	2.32	C6H10N2O5	Asp-Gly	HC11,HC3,LC11,LC3
16.16	22.32	409.21	1.68	C19H28N4O6	Leu-Asn-Tyr	HC11,HC3,LC11,LC3
16.17	22.34	455.32	-1.68	C23H42N4O5	Ile-Leu-Leu-Pro	HC3
16.18	19.24	379.21	0.45	C17H26N6O4	Pentosidine	HC11,HC3,LC11,LC3
16.18	22.36	465.22	1.64	C22H32N4O5S	Ala-Met-Phe-Pro	HC3
16.19	13.68	302.14	2.06	C12H19N3O6	Glu-Gly-Pro	HC3,LC11,LC3
16.25	11.50	487.18	0.13	C18H26N6O10	Asp-Thr-Asp-His	HC3
16.27	15.20	162.08	1.03	C6H11NO4	L-2-Aminoadipate	HC11,HC3,LC11,LC3
16.28	18.05	142.05	2.45	C6H7NO3	2-Aminomuconate semialdehyde	LC11
16.29	20.23	304.10	2.37	C12H17NO8	Gynocardin	LC11
16.31	22.62	477.31	2.31	C25H40N4O5	Ile-Phe-Val-Val	HC3,LC3
16.38	11.77	558.35	2.94	C22H43N11O6	Arg-Leu-Asn-Arg	HC11,HC3,LC11,LC3
16.39	11.78	521.32	2.16	C24H40N8O5	Ala-Lys-Phe-Arg	HC3
16.45	20.66	143.05	1.42	C5H6N2O3	4-Imidazolone-5-acetate	HC3,LC11,LC3
16.49	22.98	231.13	-0.57	C20H36N4O8	Glu-Leu-Leu-Ser	LC3
16.51	14.24	235.09	2.46	C8H14N2O6	Glu-Ser	HC11,HC3,LC11,LC3
16.54	15.29	249.07	2.74	C8H12N2O7	Asp-Asp	HC11,HC3,LC11,LC3

16.59	23.67	316.22	2.55	C15H29N3O4	Leu-Leu-Ala	HC3
16.62	23.24	429.27	2.40	C20H36N4O6	Ile-Leu-Pro-Ser	HC3,LC3
16.64	13.21	364.10	1.50	C12H17N3O10	Asp-Asp-Asp	HC3,LC11
16.71	16.71	58.07	1.58	C3H7N	Cyclopropylamine	HC3,LC3
16.74	20.93	127.02	2.70	C2H7O4P	2-Hydroxyethylphosphonate	HC11,HC3,LC11,LC3
16.75	12.50	402.26	2.38	C15H31N9O4	Ala-Arg-Arg	LC3
16.76	24.18	208.13	0.27	C12H17NO2	3-(Dimethylamino)propyl benzoate	HC3,LC3
16.76	13.31	116.11	2.08	C6H13NO	Trimethylaminoacetone	HC11,HC3,LC11,LC3
16.76	16.15	263.18	-0.25	C15H22N2O2	Argyrolobine	HC3,LC3
16.77	23.53	415.26	-0.68	C19H34N4O6	Ile-Val-Pro-Ser	HC3,LC11,LC3
16.77	9.63	260.05	2.15	C6H14NO8P	alpha-D-Glucosamine 1-phosphate	LC3
16.78	24.07	344.26	-2.35	C22H33NO2	Veatchine	LC3
16.81	23.01	126.13	-1.24	C8H15N	gamma-Coniceine	HC11,HC3,LC3
16.84	23.06	155.01	0.48	C3H7O5P	Propanoyl phosphate	HC11
16.85	20.74	326.21	-0.40	C16H27N3O4	Leu-Pro-Pro	HC11,HC3,LC3
16.91	19.46	279.21	1.76	C16H26N2O2	Lycocernuine	HC3,LC3
16.97	20.86	158.12	1.41	C8H15NO2	Homostachydrine	HC3,LC11,LC3
17.00	14.70	379.13	-0.36	C13H22N4O7S	Met-Asn-Asp	HC3,LC3
17.05	23.14	153.10	2.49	C8H12N2O	4-(hydroxylamino)-N,N-dimethylaniline	HC11,HC3,LC11,LC3
17.05	13.64	201.16	-2.48	C10H20N2O2	dimethylsuberimidate	HC11,HC3,LC11,LC3
17.08	23.71	328.22	1.14	C16H29N3O4	Leu-Val-Pro	HC11,HC3,LC11,LC3
17.30	13.10	377.13	2.27	C12H21N6O6P	puromycin aminonucleoside 5'-monophosphate	LC11
17.35	12.96	265.19	1.59	C15H24N2O2	13-Hydroxylupanine	HC3
17.44	23.61	390.05	0.34	C11H19NO10S2	2-hydroxy-3-butenylglucosinolate	LC11
17.46	21.67	128.07	1.01	C6H9NO2	alpha-(Methylenecyclopropyl)glycine	HC3,LC11,LC3

17.57	25.14	432.24	-2.51	C18H33N5O7	Ala-Leu-Thr-Gln	HC11,HC3,LC11
18.08	16.75	320.15	2.27	C12H21N3O7	Val-Asp-Ser	HC11,HC3,LC3
18.19	18.19	829.28	0.31	C30H52O26	Cellopentaose	HC11
18.23	23.91	127.02	0.70	C2H7O4P	2-Hydroxyethylphosphonate	HC11,HC3,LC11,LC3
18.29	22.22	279.21	1.76	C16H26N2O2	Lycocernuine	HC11,HC3,LC11,LC3
18.51	12.14	196.08	2.56	C6H13NO6	2-Amino-2-deoxy-D-gluconate	HC11,HC3
18.55	25.85	213.02	-1.27	C5H9O7P	P-DPD	HC3
18.61	16.21	608.23	-1.75	C29H33N7O6S	Cys-Trp-Tyr-His	HC3
18.61	22.44	170.15	0.76	C10H19NO	Lupinine	HC11,HC3,LC3
18.67	16.33	501.33	1.89	C20H40N10O5	Ala-Val-Arg-Arg	HC11,HC3,LC11,LC3
18.67	16.35	516.29	-0.68	C25H37N7O5	Arg-Phe-Pro-Pro	HC3
18.67	16.35	468.28	2.05	C18H33N11O4	Arg-Arg-His	HC3
18.68	16.36	444.30	-2.27	C18H37N9O4	Leu-Arg-Arg	HC3,LC3
18.71	18.71	450.29	-1.42	C19H39N5O7	Gentamicin C1a	HC11,HC3
18.72	17.64	325.04	-0.63	C9H13N2O9P	Pseudouridine 5'-phosphate	HC3
18.73	17.60	235.09	2.03	C8H14N2O6	Thr-Asp	HC3,LC11
18.75	17.43	364.10	0.63	C12H17N3O10	Asp-Asp-Asp	HC11,HC3,LC11,LC3
18.80	14.89	307.15	-1.92	C13H18N6O3	Lupinate	HC11,HC3,LC11
18.82	26.30	208.15	1.29	C11H17N3O	Arenaine	HC3
18.87	16.75	474.28	1.07	C23H35N7O4	Leu-Trp-Arg	HC11,HC3,LC11,LC3
19.04	25.71	146.08	0.74	C6H11NO3	6-Amino-2-oxohexanoate	HC3,LC3
19.04	21.57	160.13	-2.35	C8H17NO2	Methacholine	HC3
19.05	21.49	84.08	-2.10	C5H9N	Piperideine	HC3,LC3

19.08	22.11	128.07	1.13	C6H9NO2	Guvacine	HC3,LC3
19.09	19.09	58.07	1.37	C3H7N	Cyclopropylamine	HC11,HC3,LC11
19.10	17.19	592.24	2.40	C29H33N7O5S	Cys-Phe-Trp-His	HC3,LC3
19.12	19.12	473.12	-0.06	C14H26N4O10P2	CMP-N-trimethyl-2-aminoethylphosphonate	HC3
19.12	17.25	454.21	0.98	C20H31N5O5S	Cys-Lys-Phe-Gly	HC3
19.14	14.25	351.10	0.29	C11H18N4O7S	Asn-Asp-Cys	HC11,HC3
19.15	17.84	201.16	-0.66	C10H20N2O2	dimethylsuberimidate	HC3,LC3
19.15	19.15	423.09	0.88	C12H23O14P	alpha,alpha'-Trehalose 6-phosphate	HC3
19.16	19.16	413.03	-2.16	C10H14N4O10P2	dIDP	HC3
19.18	17.35	422.15	1.94	C18H23N5O5S	Cys-Tyr-His	HC3
19.22	23.36	131.12	-1.84	C6H14N2O	ε-aminocaproamide	HC11,HC3,LC3
19.23	25.38	158.12	1.53	C8H15NO2	Homostachydrine	HC3,LC11,LC3
19.28	22.56	217.11	-2.17	C16H28N6O8	Ala-Gln-Gln-Ser	HC11,LC11,LC3
19.42	17.85	261.13	2.28	C24H36N6O5S	Lys-Met-Trp-Gly	LC11,LC3
19.53	25.38	98.98	2.52	H3O4P	Orthophosphate	HC11,LC11,LC3
19.60	18.20	250.11	-1.95	C20H30N6O9	Asp-Leu-Asp-His	LC11
19.73	18.47	429.25	1.66	C18H32N6O6	Ala-Lys-Asn-Pro	HC11,LC11,LC3
19.75	18.50	608.23	-1.11	C29H33N7O6S	Cys-Trp-Tyr-His	LC3
19.82	13.31	322.19	2.02	C15H23N5O3	Phe-Arg	LC3
19.86	26.04	162.08	2.05	C6H11NO4	O-Acetyl-L-homoserine	HC11,LC3
19.96	18.93	420.20	2.39	C15H29N7O5S	Met-Asn-Arg	HC11,LC11
20.05	28.81	390.05	-0.04	C11H19NO10S2	2-hydroxy-3-butenylglucosinolate	LC11,LC3
20.11	28.82	311.21	-1.68	C20H26N2O	Ibogaine	LC3

20.12	20.12	598.12	-2.97	C22H31NO14S2	4-sinapoyloxybutylglucosinolate	LC11
20.20	20.32	364.10	1.32	C12H17N3O10	Asp-Asp-Asp	HC11,LC11,LC3
20.24	23.55	249.07	1.97	C8H12N2O7	L-beta-aspartyl-L-asparticacid	HC11,LC11
20.38	21.86	373.28	-0.38	C18H36N4O4	Leu-Leu-Lys	LC3
20.39	25.79	479.13	-0.39	C16H22N4O13	Asp-Asp-Asp-Asp	LC11
20.40	12.23	331.23	-1.73	C15H30N4O4	Ile-Ala-Lys	LC3
20.41	26.14	160.13	-1.81	C8H17NO2	Propionylcholine	LC3
20.41	14.07	189.09	-1.88	C7H12N2O4	L-glycyl-L-hydroxyproline	LC11
20.44	25.88	432.17	-2.78	C16H25N5O9	Asn-Asp-Pro-Ser	LC3
20.44	24.89	434.17	-0.15	C16H27N5O7S	Asn-Thr-Cys-Pro	LC3
20.49	13.74	319.12	-1.30	C11H18N4O7	Ala-Asn-Asp	LC3
20.49	28.54	146.08	2.13	C6H11NO3	L-2-Aminoadipate 6-semialdehyde	HC11,LC11,LC3
20.50	27.58	128.07	-0.41	C6H9NO2	L-Baikiain	LC3
20.50	20.50	413.03	-1.92	C10H14N4O10P2	dIDP	LC3
20.51	20.51	423.09	2.08	C12H23O14P	alpha,alpha'-Trehalose 6-phosphate	LC3
20.52	20.06	174.15	-1.76	C9H19NO2	Muscarine	LC3
20.55	17.81	351.10	1.38	C11H18N4O7S	Asp-Cys-Gly-Gly	HC11,LC11,LC3
20.63	20.78	201.16	1.36	C10H20N2O2	dimethylsuberimidate	LC11,LC3
21.14	29.78	346.17	1.08	C19H23NO5	Aknadicine	HC11,LC11
21.22	30.14	139.09	1.18	C7H10N2O	ProPAM	HC11,LC11

21.24	28.64	171.01	-1.21	C3H7O6P	D-Glyceraldehyde 3-phosphate	HC3,LC3
21.36	21.36	205.06	0.84	C19H20O10	Khellol glucoside	HC11,LC11,LC3
21.38	29.76	431.05	-1.69	C12H22N4O5S4	Cys-Cys-Cys-Cys	HC3,LC3
21.45	26.91	420.16	0.94	C15H25N5O7S	Asn-Cys-Pro-Ser	LC3
21.46	30.87	155.01	1.61	C3H7O5P	Hydroxyacetone phosphate	HC11
21.52	29.50	183.99	-0.02	C4H9NO2Se	Selenohomocysteine	HC3,LC3
21.57	22.15	422.15	2.55	C18H23N5O5S	Cys-Tyr-His	HC3,LC3
21.67	22.57	273.09	-1.40	C11H16N2O4S	Thienamycin	HC3,LC3
21.92	22.83	433.18	-2.87	C19H24N6O6	Asn-Tyr-His	LC3
22.04	24.00	364.10	1.40	C12H17N3O10	Asp-Asp-Asp	HC11,LC11
22.06	26.33	249.07	2.65	C8H12N2O7	Asp-Asp	LC11,LC3
22.09	12.47	175.14	-0.85	C8H18N2O2	Ne,Ne dimethyllysine	HC11,HC3,LC11,LC3
22.52	24.05	272.11	-1.60	C21H30N6O11	His-Glu-Glu-Glu	HC3,LC11
22.54	30.08	405.14	0.65	C15H24N4O7S	Ala-Asp-Cys-Pro	LC11,LC3
22.64	30.69	362.14	-2.27	C14H23N3O6S	Met-Asp-Pro	HC3,LC3
22.70	31.01	156.15	-1.32	C8H17N3	1-ethyl-3-(3-dimethylaminopropyl)-carbodiimide	HC3,LC3
22.74	25.01	201.16	-1.60	C10H20N2O2	dimethylsuberimidate	HC11,HC3,LC11,LC3
23.02	25.03	456.14	-0.94	C15H29N5O5S3	Cys-Lys-Cys-Cys	LC3
23.14	31.27	246.09	0.21	C17H26N6O11	Asn-Asp-Asp-Gln	HC11,HC3,LC3
23.30	33.64	326.12	-2.53	C14H19N3O4S	Phe-Cys-Gly	LC3
23.54	16.26	317.15	1.18	C12H20N4O6	Asn-Pro-Ser	LC11

23.62	23.62	922.39	-1.09	C37H59N7O20	GlcNAc-1,6-anhMurNAc-L-Ala-γ-D-Glu-DAP-D-Ala	HC3,LC3
23.90	26.81	272.11	-2.59	C21H30N6O11	His-Glu-Glu-Glu	HC3
23.96	26.91	491.21	-1.49	C18H30N6O10	Asn-Lys-Asp-Asp	HC11,HC3,LC11,LC3
24.05	27.11	489.21	2.48	C19H32N6O7S	His-Met-Thr-Thr	HC11,HC3,LC11,LC3
24.09	33.58	362.14	-2.92	C14H23N3O6S	Met-Asp-Pro	HC3,LC3
24.18	33.69	162.08	-0.55	C6H11NO4	L-Glutamate methylester	HC11,HC3,LC11,LC3
24.37	22.63	156.08	1.00	C6H9N3O2	L-Histidine	HC11,LC11
24.44	34.79	184.11	2.24	C8H13N3O2	Nalpha,Nalpha-Dimethyl-L-histidine	LC11
24.51	24.30	180.09	0.50	C6H13NO5	D-Glucosamine	HC3,LC3
24.72	23.37	364.95	-2.34	C8H16N2O4Se2	Selenohomocystine	LC3
25.27	29.55	460.26	-0.20	C17H33N9O6	Glu-Arg-Arg	HC11,HC3,LC11,LC3
25.35	23.54	175.12	2.32	C6H14N4O2	L-Arginine	HC11,LC11,LC3
25.41	36.41	156.15	-1.68	C8H17N3	1-ethyl-3-(3-dimethylaminopropyl)-carbodiimide	HC3,LC11,LC3
25.41	25.41	411.13	0.54	C19H22O10	aloenin	HC11,HC3,LC11,LC3
25.43	35.85	186.04	-2.30	C7H7NO5	2-Amino-3-carboxymuconate semialdehyde	HC3,LC11,LC3
25.44	29.89	519.30	-2.30	C19H38N10O7	Arg-Thr-Ser-Arg	HC11,HC3,LC3
25.46	35.39	366.14	-0.33	C14H23NO10	2-(acetylamino)-1-5-anhydro-2-deoxy-3-O-b-D-galactopyranosyl-D-arabino-Hex-1-enitol	HC11,HC3,LC11,LC3
25.48	33.36	342.16	-0.36	C17H19N5O3	Trp-His	HC3,LC11,LC3
25.69	34.69	132.10	-0.36	C6H13NO2	beta-Alaninebetaine	HC11,HC3,LC11,LC3
25.91	25.91	458.18	-0.92	C20H23N7O6	5,10-Methylenetetrahydrofolate	LC3

26.29	31.57	269.64	-1.58	C25H39N5O6S	Lys-Met-Pro-Tyr	HC3
26.36	19.73	189.16	0.85	C9H20N2O2	N6,N6,N6-Trimethyl-L-lysine	LC11
26.49	21.27	175.14	-0.64	C8H18N2O2	Ne,Ne dimethyllysine	HC11,HC3,LC11,LC3
26.60	35.95	272.16	2.25	C12H21N3O4	Val-Gly-Pro	HC11,LC11
26.60	32.74	201.16	0.49	C10H20N2O2	dimethylsuberimidate	HC3,LC11,LC3
26.62	32.23	430.24	-1.89	C22H31N5O4	Lys-Trp-Pro	HC11,HC3,LC11,LC3
26.62	36.19	198.04	2.87	C5H11NO5S	Tauropine	LC11,LC3
26.77	32.54	431.19	-0.97	C16H26N6O8	His-Thr-Ser-Ser	LC11
26.79	32.29	158.12	-2.20	C8H15NO2	Lentiginosine	HC3,LC11,LC3
26.97	32.94	258.19	0.88	C23H46N8O5	Arg-Leu-Lys-Val	HC3,LC3
27.04	27.04	411.13	2.29	C19H22O10	aloenin	HC3,LC11
27.07	33.73	152.04	-2.25	C4H9NO3S	methiin	HC3,LC3
27.15	39.30	479.13	-1.41	C16H22N4O13	Asp-Asp-Asp-Asp	HC11,HC3,LC11
27.54	17.52	375.20	2.97	C14H26N6O6	Glu-Ala-Arg	HC11,HC3,LC11
27.57	22.94	344.16	1.75	C13H21N5O6	Asn-Asn-Pro	HC11,HC3,LC11
27.66	34.33	202.12	1.90	C16H30N6O6	Leu-Asp-Arg	LC11
28.07	35.14	254.15	1.84	C24H38N6O6	Asn-Lys-Phe-Val	HC11,LC11
28.08	18.32	203.18	-0.70	C10H22N2O2	Spermine dialdehyde	HC11,LC11,LC3
28.19	40.38	431.19	-0.22	C16H26N6O8	Asn-Asn-Pro-Ser	LC11
28.24	35.49	465.10	-0.19	C15H24N6O5S3	Cys-Cys-Cys-His	HC3
28.26	28.26	54.94	-1.82	Mn	Mn2+	HC3

28.34	24.52	330.29	-0.81	C21H35N3	Jamine	HC3,LC11,LC3
28.35	35.69	491.21	0.48	C18H30N6O10	Asn-Lys-Asp-Asp	HC3,LC11
28.35	35.69	413.18	0.98	C16H24N6O7	Ala-Ala-Asp-His	LC11
28.42	35.85	544.24	2.59	C25H33N7O5S	Ala-Met-Trp-His	HC3,LC3
28.48	28.84	169.10	2.32	C8H12N2O2	Pyridoxamine	LC11
28.55	28.55	55.94	1.14	Fe	Fe2+	HC11,HC3,LC11,LC3
28.57	34.15	359.27	2.36	C17H34N4O4	Leu-Lys-Val	HC3,LC3
28.65	36.30	251.18	-2.69	C22H44N8O5	Arg-Lys-Val-Val	HC3,LC3
28.87	36.74	460.26	-1.68	C17H33N9O6	Glu-Arg-Arg	LC11
29.02	37.04	540.32	-1.51	C29H41N5O5	Lys-Phe-Phe-Val	HC3,LC11,LC3
29.08	37.15	430.24	-2.16	C22H31N5O4	Lys-Trp-Pro	HC11,HC3,LC11,LC3
29.13	26.54	175.14	1.37	C8H18N2O2	Ne,Ne dimethyllysine	HC3,LC11,LC3
29.19	37.38	547.30	-0.96	C22H42N8O6S	Arg-Leu-Met-Gln	HC3,LC11
29.19	37.39	532.33	-1.73	C23H45N7O5S	Arg-Leu-Leu-Met	LC11
29.26	41.21	334.14	-1.22	C13H23N3O5S	Met-Pro-Ser	HC3,LC3
29.32	29.32	581.15	2.20	C26H28O15	Carlinoside	LC11,LC3
29.36	22.31	298.15	2.04	C12H19N5O4	Ala-Ala-His	LC11
29.60	38.20	634.31	0.97	C31H39N9O6	Arg-Trp-Trp-Ser	HC3,LC11,LC3
29.64	38.28	603.32	-0.77	C28H42N8O7	Glu-Leu-Trp-Arg	HC11,HC3,LC11,LC3
29.80	38.60	560.33	-1.00	C21H41N11O7	Arg-Thr-Gln-Arg	HC11,LC11,LC3
29.82	42.94	132.10	0.47	C6H13NO2	beta-Alaninebetaine	HC11,HC3,LC11,LC3
29.87	28.50	126.10	1.54	C6H11N3	N-Methylhistamine	HC11
29.98	39.29	129.10	1.71	C6H12N2O	L-Lysine 1,6-lactam	HC11,HC3,LC11,LC3

30.02	42.17	131.12	1.64	C6H14N2O	N-Acetylputrescine	HC11,HC3,LC11,LC3
30.04	39.08	538.35	2.62	C24H43N9O5	Arg-Leu-Leu-His	HC11,HC3,LC11,LC3
30.39	30.39	60.05	2.82	C2H5NO	Aminoacetaldehyde	HC11,HC3,LC11,LC3
30.61	40.22	491.21	-1.53	C18H30N6O10	Asn-Lys-Asp-Asp	HC11,HC3,LC3
30.72	40.97	201.16	-0.24	C10H20N2O2	dimethylsuberimidate	HC11,HC3,LC11,LC3
31.09	31.09	55.94	1.10	Fe	Fe2+	HC3,LC11,LC3
31.42	34.43	174.11	1.66	C8H15NO3	Swainsonine	HC11,HC3,LC11,LC3
32.04	40.99	330.06	-2.88	C10H12N5O6P	2',3'-Cyclic AMP	HC11,HC3,LC11,LC3
32.82	32.82	51.94	2.60	Cr	Cr+2	HC11,HC3
33.71	46.41	268.15	2.57	C22H42N6O7S	Glu-Lys-Lys-Met	HC3
40.50	40.50	55.94	1.87	Fe	Fe2+	HC11,LC11,LC3
42.57	48.00	180.09	2.97	C6H13NO5	D-Galactosamine	HC11,HC3,LC3
43.48	55.24	175.14	1.66	C8H18N2O2	Ne,Ne dimethyllysine	HC11,HC3,LC11,LC3
43.86	43.86	55.94	1.53	Fe	Fe2+	HC11,LC11,LC3
43.98	43.98	250.97	1.09	C6H16O18P4	1D-myo-Inositol 1,3,4,5-tetrakisphosphate	HC11,HC3,LC11,LC3
44.56	63.14	397.19	2.32	C18H28N4O4S	Lys-Phe-Cys	HC11,HC3,LC11,LC3
44.70	44.70	537.17	0.53	C18H32O18	1-4-beta-D-Glucan	HC11,HC3,LC11,LC3
45.53	45.53	700.23	2.81	C29H33N9O12	Pteroyltriglutamicacid	HC11,HC3,LC11,LC3
45.92	45.92	775.25	-1.03	C30H43N6O16P	7,8-Dihydromethanopterin	HC11,HC3,LC11,LC3

1. Sumner LW, Amberg A, Barrett D, et al. Proposed minimum reporting standards for chemical analysis. Metabolomics 2007;3:211-21.

Table S8. Detection of metabolites involved in common metabolic pathways. Pathways are defined as in the KEGG database. The second column shows the combined number of metabolites detected by HPLC/HRMS in positive and negative ion modes and the total number of metabolites listed for that pathway. The right column lists the samples in which the compounds in the respective pathway were preferentially detected. Yellow highlights indicate greater representation in LC samples; green highlights indicate greater representation in HC samples; and "All" indicates that no significant differences in the number of detected metabolites were observed between the LC and HC systems.

Pathway	# metabolites detected / metabolites in pathway	Samples
Tyrosine metabolism	18/74	All
Arginine and proline metabolism	16/80	All
Phenylalanine metabolism	16/64	All
Tropane, piperidine and pyridine alkaloids	16/61	All
Tryptophan metabolism	16/80	All
Lysine degradation	15/39	HC 3 LC 3
Histidine metabolism	11/44	All
Steroid hormone biosynthesis	11/99	HC 11
Nicotinate and nicotinamide metabolism	10/45	All
Phenylpropanoid biosynthesis	10/51	HC 11
Porphyrin and chlorophyll metabolism	10/93	HC 11
Purine metabolism	10/90	All
Pyrimidine metabolism	9/58	All
Alanine, aspartate and glutamate metabolism	8/24	All
Amino sugar and nucleotide sugars	8/79	LC 3
Aminoacyl-tRNA biosynthesis	8/24	All
Butanoate metabolism	8/39	All
1,4-Dichlorobenzene degradation	7/74	All
Cysteine and methionine metabolism	7/54	All
Pantothenate and CoA biosynthesis	7/25	All
Arachidonic acid metabolism	6/74	HC11
Benzoate degradation via hydroxylation	6/66	All
Isoquinoline alkaloid biosynthesis	6/93	All
Methane metabolism	6/58	All
Valine, leucine and isoleucine biosynthesis	6/23	All
Benzoate degradation via CoA ligation	6/57	All

Biosynthesis of unsaturated fatty acids	6/49	All
Glutathione metabolism	6/29	All
Linoleic acid metabolism	6/25	HC11
Lysine biosynthesis	6/26	All
Pyruvate metabolism	6/31	All
Butanoate metabolism	6/39	All
beta-Alanine metabolism	6/31	All
Starch and sucrose metabolism	6/37	All
Stilbenoid, diarylheptanoid and gingerol biosynthesis	5/24	HC11
Taurine and hypotaurine metabolism	5/22	HC11
Diterpenoid biosynthesis	5/69	HC11
Fatty acid biosynthesis	5/10	All
Fluorene degradation	5/36	All
Glycine, serine and threonine metabolism	5/46	All
Biosynthesis of 12-, 14- and 16-membered macrolides	4/75	HC11
C5-Branched dibasic acid metabolism	4/32	All
Flavonoid biosynthesis	4/68	HC11
Glucosinolate biosynthesis	4/72	All
Glycolysis / Gluconeogenesis	4/28	All
Propanoate metabolism	4/36	HC11 LC11
Zeatin biosynthesis	4/36	HC3
gamma-Hexachlorocyclohexane degradation	4/72	All
1- and 2-Methylnaphthalene degradation	4/63	LC11
Biosynthesis of type II polyketide products	4/86	All
Citrate cycle (TCA cycle)	4/16	All
Folate biosynthesis	4/27	All
Glyoxylate and dicarboxylate metabolism	4/53	All
Primary bile acid biosynthesis	4/47	All
Reductive carboxylate cycle (CO2 fixation)	4/41	All
Sulfur metabolism	4/21	All
Cyanoamino acid metabolism	4/25	All
Limonene and pinene degradation	4/59	All
Biphenyl degradation	3/29	All
Carbazole degradation	3/18	All
Insect hormone biosynthesis	3/23	All
Pentose phosphate pathway	3/34	All
Phenylalanine, tyrosine and tryptophan biosynthesis	3/31	All

Ubiquinone and other terpenoid-quinone biosynthesis	3/35	HC11
Ascorbate and aldarate metabolism	3/47	All
Carbon fixation in photosynthetic organisms	3/22	All
Clavulanic acid biosynthesis	3/10	HC3 LC3
Ethylbenzene degradation	3/14	All
Vitamin B6 metabolism	3/32	All
alpha-Linolenic acid metabolism	3/39	All
Secondary bile acid biosynthesis	3/11	HC11
2,4-Dichlorobenzoate degradation	2/29	All
Anthocyanin biosynthesis	2/56	All
Betalain biosynthesis	2/24	HC3 LC3
Biotin metabolism	2/11	HC11 LC11
Fatty acid metabolism	2/39	HC11 LC11
Indole alkaloid biosynthesis	2/47	HC11
Novobiocin biosynthesis	2/23	HC11
Phosphonate and phosphinate metabolism	2/39	HC11 HC3
Riboflavin metabolism	2/19	All
Sesquiterpenoid biosynthesis	2/34	All
Thiamine metabolism	2/20	All
